# Evaluation metrics and validation of presence-only species distribution models based on distributional maps with varying coverage

**DOI:** 10.1038/s41598-020-80062-1

**Published:** 2021-01-15

**Authors:** Kamil Konowalik, Agata Nosol

**Affiliations:** Department of Plant Biology, Institute of Environmental Biology, Wrocław University of Environmental and Life Sciences, Kożuchowska 5b, 51-631 Wroclaw, Poland

**Keywords:** Biogeography, Conservation biology, Ecological modelling

## Abstract

We examine how different datasets, including georeferenced hardcopy maps of different extents and georeferenced herbarium specimens (spanning the range from 100 to 85,000 km^2^) influence ecological niche modeling. We check 13 of the available environmental niche modeling algorithms, using 30 metrics to score their validity and evaluate which are useful for the selection of the best model. The validation is made using an independent dataset comprised of presences and absences collected in a range-wide field survey of Carpathian endemic plant *Leucanthemum rotundifolium* (Compositae). Our analysis of models’ predictive performances indicates that almost all datasets may be used for the construction of a species distributional range. Both very local and very general datasets can produce useful predictions, which may be more detailed than the original ranges. Results also highlight the possibility of using the data from manually georeferenced archival sources in reconstructions aimed at establishing species’ ecological niches. We discuss possible applications of those data and associated problems. For the evaluation of models, we suggest employing AUC, MAE, and Bias. We show an example of how AUC and MAE may be combined to select the model with the best performance.

## Introduction

One of the main tasks of field biologists is the collection and cataloging of specimens. The resulting collections represent invaluable sources of information regarding species occurrence and distribution. Such data are often used in distribution modeling, which tries to reconstruct the potential distribution of a species from known occurrences^1^. Every point on the Earth may be described by a set of environmental values, along with the presence or absence of species. A distribution model may be constructed by comparing environmental conditions with their associated presence/absence of species. The ultimate goal in generating a distribution model is to determine which variables are most influential on species occurrences, and then generate the model that best approximates the real distribution (Species Distribution Modeling, SDM) or describes the niche of the species (Ecological Niche Modeling, ENM)^2^. The final model may be affected by many factors, among which the most important are the employed algorithms and their settings, the availability and quality of presence records, the resolution of variables, and the data describing these variables^3^. ENM commonly describes a broader area than the actual range of the species, since its purpose is to characterize ecological niches, portions of which may be outside the range actually utilized by a species due to dispersion limits, predation, competition or other factors that exclude it from a particular territory^2^. There are several modeling algorithms with varying assumptions used for both SDM and ENM, but the choice of a particular algorithm seems to be influenced by convention, rather than initial testing^4^.

Besides the choice of modeling techniques or variables used, the quality and number of data points is an important concern in proper modeling setup. Several services collect species occurrences and offer distributional data (GBIF, Tropicos, iNaturalist, iDigBio, etc.). These sources possess rich datasets, but also face certain limitations^5–7^. For example, services collecting data through “citizen science” or digitized collections are heavily dependent on factors such as time when a species is observable, species popularity, population density, and region accessibility, resulting in the fact that coverage of many regions of the world is still poor^8–11^. The reliability of the data in such databases also varies, depending on the expertise of the data collectors and whether they provide additional information that enables confirmation by a specialist. This last issue may be avoided by using material stored in museums, where identification of species may be verified. Such museum-cataloged data is invaluable in determining species range^12,13^. Gathering herbarium specimens is an important procedure carried out as an exclusive task or as documentation performed alongside other studies^14^. Many collections store valuable material that either includes coordinates or may be automatically or manually georeferenced^15^, although georeferencing such data may be problematic^16^. The most common issue is very general locality information, which in extreme cases may be comprised only of a country or a mountain range. In addition, ambiguous location names may pose a serious challenge, as the same name may be used for several places within one region^17,18^. Other problems arise from the use of ephemeral stands or localities where conditions have changed since collection (e.g., collecting a specimen in a puddle or along a roadside).

Besides herbarium datasets, other sources of species distribution information are distributional maps and atlases containing either ranges or occurrence points for a given taxon. The task of many historical and contemporary authors has been the production of such maps of distribution, which are often still the main sources of information about some groups. These maps may be especially rich sources of data on species occurring in remote or hardly accessible areas. They are also useful for species that are difficult to properly identify, especially due to high morphological complexity. Another advantage of maps over herbarium specimens or online databases is that georeferencing maps is faster and less error-prone. Moreover, it is inherently more accurate than assigning new coordinates because points have already been validated. However, the quality and resolution of such maps may differ depending on the purpose of the work, e.g., whether it was a floristic inventory aimed at describing all species within an area or a systematic monograph focused on certain taxa. Theoretically, the former map type will yield more points locally, but with some possibility of error, while the latter will yield fewer points locally, but with more general distribution and described more accurately.

In this article, we analyze the interplay between occurrence data and modeling algorithms, and determine whether georeferenced hardcopy maps may be used as a modeling input. Various papers have already compared modeling methods and their settings^5,19,20^. However, most of these have been based on the use of artificial species, a technique which inherently involves various assumptions regarding the target species’ initial niche^21–23^. Here, we use a Carpathian endemic plant, *Leucanthemum rotundifolium*. This species possesses a very clear distributional pattern and characteristic habitat, making it a good choice for a modeling case study. In this study, we use field-collected data gathered throughout the species’ entire distribution for all evaluation purposes. Various previous papers compared evaluation metrics, but typically focused on only a few of them, which were mostly chosen because of usage frequency^24^. More comprehensively, we employed 30 metrics and 13 modeling algorithms easily accessible within the R platform to interpret relationships between them and find the most useful metrics for model evaluation and ground-truthing. The example used in this study will help to improve ecological modeling, which in turn will increase the scientific community’s understanding of the distribution of endemic and mountain plants^25^.

## Materials and methods

### Study species

The focal species of this study was the round-leaved oxeye daisy *Leucanthemum rotundifolium* (Willd.) DC. (Compositae, Anthemideae), a Carpathian sub-endemic plant. Except for one isolated population in the Vranica Mountains (Bosnia and Herzegovina), it occurs exclusively in the montane to subalpine belt of the Carpathian Mountains^26^. The Carpathians, located in Central Europe, are important as a biogeographical connection between the Alps, the Balkan Mountains, and the Scandinavian Mountains^27,28^. They are also one of the main hotspots of European biodiversity, harboring several endemic plants and animals^29^. *L. rotundifolium* is specialist in regards to habitat; it is a montane plant, with no collection ever being made below 525 m, close to the border of the lower montane belt. Typically, it is found in partial shade within *Picea abies*-dominated forests or within swaths of tall herbaceous plants close to mountain streams. It may also grow in full sun, but only in areas with sufficient soil moisture, such as local depressions with ephemeral water flow. In general, its high water requirements confine its occurrences to stream banks or close to any water (lakes, depressions, ditches, temporary streams). The species is less numerous at higher altitudes (subalpine, alpine), but may be found within *Pinus mugo* shrubs or in ditches and local depressions covered with higher perennials (e.g., *Vaccinium* spp.) that provide it with shade or snow accumulation. It avoids dense forests and areas in heavy shadow or with copious organic litter. It occurs throughout the whole Carpathian range, and in some places, is quite common (particularly in the Western Carpathians).

### Localities and georeferencing

**Figure 1:**
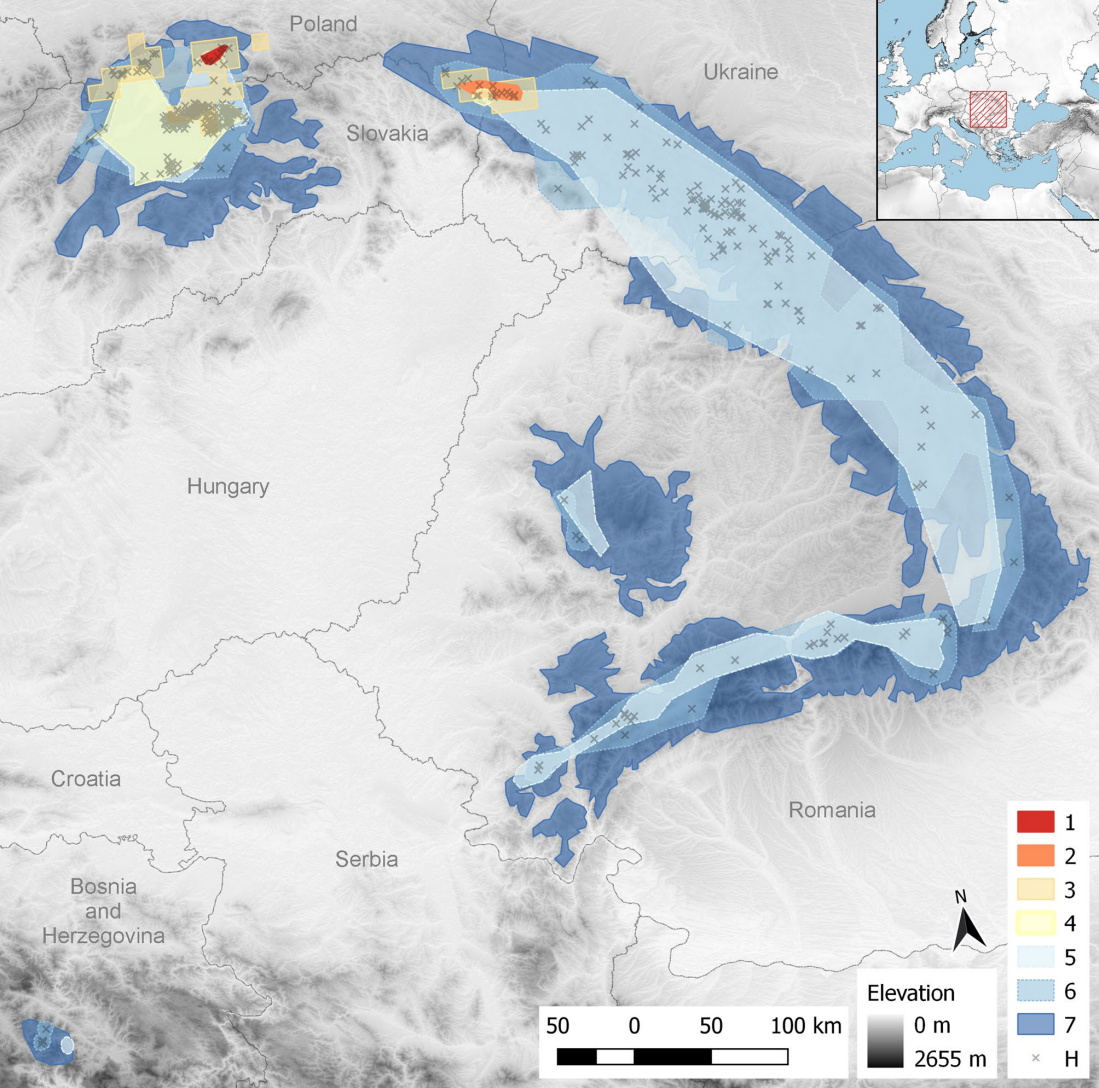
Extents of occurrence datasets used in this study: 1—Gorce Mts^30^, 2—Bieszczady Mts^31^, 3—Atpol quadrats^32^, 4—Slovakian occurrences^26^, 5—Carpathian occurrences^26^, 6—georeferenced herbarium material, 7—distribution in the Carpathians^33^. H (cross)—denotes herbarium localities and displays findings and sampling intensity. All shapes are constructed as convex hulls around points; only datasets 3 and 7 are polygons of species range. The red hatched area in the inset in the upper right corner shows the extent of the map. The underlying image represents the altitude derived from ALOS DEM dataset^34^. The map is projected in ETRS89 (EPSG: 3035).

**Figure 2:**
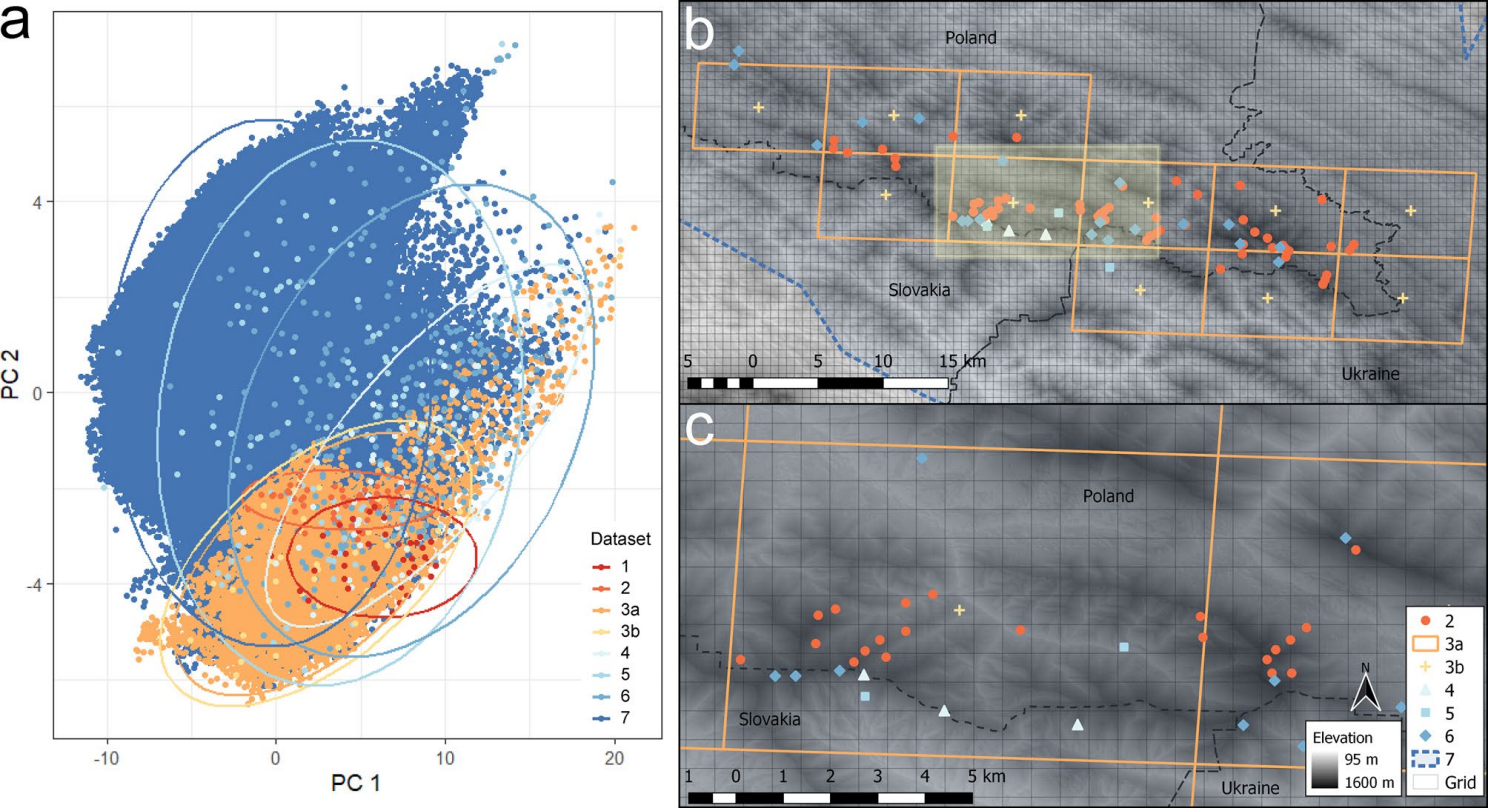
(**a**) Principal Component Analysis (PCA) made using different datasets, (**b**) map of the Bieszczady Mts. (Eastern Carpathians), and (**c**) a portion of the Bieszczady Mts. enlarged to show detailed placement of points. PCA uses as a source specified datasets and illustrates the coverage of environmental variables that corresponds to an environmental space represented by a particular dataset. Circles enclose 95% of the data. Insets (**b**) and (**c**) illustrate varying accuracy of different datasets; the best accuracy is achieved with the regional dataset, with these points aligning well to features of the terrain (2), while the most general dataset, represented by a range (7), falls far outside the recorded occurrences. Numbers correspond to those in Fig. 1: 1—Gorce Mts.^30^, 2—Bieszczady Mts.^31^, 3a—Atpol quadrats^32^, 3b—centroids of Atpol quadrats, 4—Slovakian occurrences^26^, 5—Carpathian occurrences^26^, 6—georeferenced herbarium material, 7—distribution in the Carpathians^33^. Rectangular mesh (Grid) shows the extent of variables in 30 arc seconds. The extent of c is shown by the yellow rectangle on (b). The underlying image in (b) and (c) represents the altitude derived from ALOS DEM dataset^34^ with 30 × 30 m cells.

**Figure 3:**
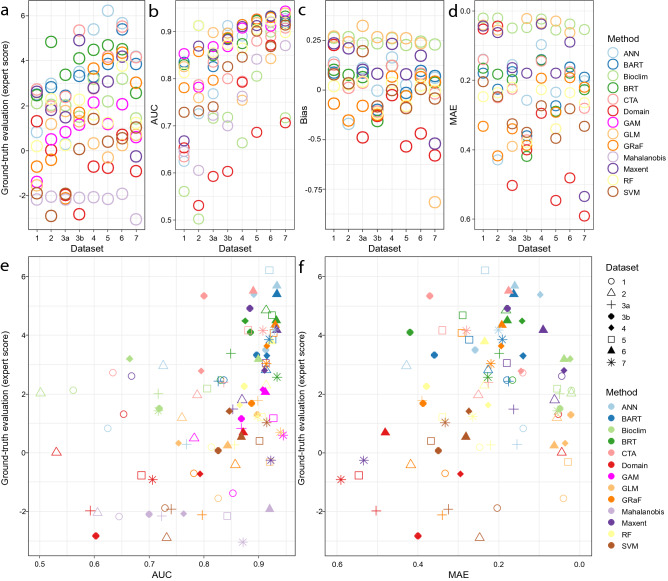
Plots representing different datasets, algorithms, and their evaluation metrics: (**a**) ground-truth evaluation (expert score), (**b**) AUC, (**c**) bias, (**d**) MAE, (**e**) relation between ground-truth evaluation and AUC, (**f**) relation between ground-truth evaluation and MAE. Numbers referring to datasets correspond to those in Fig. 1: 1—Gorce Mts^30^, 2—Bieszczady Mts^31^, 3a—Atpol quadrats^32^, 3b—centroids of Atpol quadrats, 4—Slovakian occurrences^26^, 5—Carpathian Mts occurrences^26^, 6—georeferenced herbarium material, 7—distribution in the Carpathians^33^. Note that the y-axis scale for (**d**) and the x-axis scale for (**f**) are inverted for ease of comparison with other graphs.

To examine the effects of different scales and extents on distribution modeling, several datasets were georeferenced. This was achievable due to the availability of a full range of possible inputs for the focal species, from detailed maps examining a single mountain range to maps illustrating the entire range of the species (Table 1, Fig. 1). Starting from the lowest coverage/highest resolution, the inputs used were the following datasets: distribution in the Gorce Mts (1)^30^, distribution in the Bieszczady Mts (2)^31^, distribution in Poland (3)^32^, distribution in Slovakia (4)^26^, point distribution in the Carpathian Mts (5)^26^, range in the Carpathian Mts (7)^33^. Additionally, we georeferenced herbarium material available in herbaria PRC, PR, BRNU, BRA, SLO, BP, W, KRA, KRAM (6). The georeferenced maps of the Gorce Mountains (1) and the Bieszczady Mountains (2) came from projects documenting the floristic richness of specified areas and cover relatively small regions in the Western and Eastern Carpathian Mountains, respectively. Distribution in Poland^32^ was taken from a national atlas of plant distribution that records the presence of certain species within 10 × 10 km fields. In the present publication, two variants were tested: using all points (at the 30 arc second analysis resolution, see below) within the cell for which distribution was recorded (Figs. 2, 3a) or using only the centroid of the cell (Figs. 2, 3b). Distribution in Slovakia (4) and point distribution for the entire Carpathian range (5) were georeferenced maps taken from^26^. A distributional area in the Carpathians (7) was taken from^33^; this map shows a range based on expert opinion. In this case, the range was drawn so as to enclose areas of the Carpathian Mountains within the minimum and maximum elevation range of recorded species occurrence. The polygons were converted to points by aligning them to the pixel centroids of a raster with resolution of 30 arc seconds, so that each cell covered by a polygon was treated as a point occurrence.

**Table 1:** Details of datasets used in this study showing name, type of data, number of points appearing in source and number adjusted to resolution of variables, approximate area covered by points, RMS error, and source of data. In the case of datasets 3a and 3b, we tested two versions: the original 10 × 10 km quadrats (each containing around 100 points) and the centroids of those quadrats. Datasets 1 to 5 represent scanned and georeferenced hardcopy maps, dataset 6 represents georeferenced specimens, and dataset 7 represents a species range drawn as a polygon.

Number	Dataset	Type of data	Number of points	Number of points adjusted to variables resolution	Approximate area (km^2^)	Total RMS error	Source
1	Gorce Mts	Hardcopy map	49	39	100	0.000196086	Article^30^
2	Bieszczady Mts	Hardcopy map	56	52	353	0.000132417	Article^31^
3a	Poland	Atlas; quadrats 10 × 10 km	Polygon	6435	3579	–	Atlas^32^
3b	Poland	Atlas; centroids of quadrats	36	36	3579	–	Atlas^32^
4	Slovakia	Hardcopy map	89	88	3625	0.00114941	Article^26^
5	Carpathian Mts	Hardcopy map	121	121	39,537	0.0107311	Article^26^
6	Carpathian Mts	Georeferenced specimens	390	364	44,779	–	Herbarium specimens
7	Carpathian Mts	Range	Polygon	137,166	84,619	0.0113535	Article^33^

Hardcopy maps were scanned, and georeferencing was done by selecting characteristic tie points from the maps and aligning them to vector maps of a given area. Depending on the map, tie points were placed at crossroads, rivers, political borders, or other characteristic places. Tie points were added until visually appealing results were achieved (i.e., all river and border lines matched). Afterward, species presence records were digitized and saved as a point dataset. In some cases, the black circles marking presence were large, ellipsoidal, blurred, or strongly overlapping, making it difficult to pinpoint the exact center of the mark. All datasets yielded the minimum number of 13 points that has been recommended to achieve reliable modeling results^35^, but the more local datasets should be regarded as biased or clumped, since the points were usually close to each other. The occurrence points from all datasets were thinned to only a single presence per pixel and aligned to pixel centroids of raster files of environmental predictor variables (Appendix 1).

### Variables

A common approach to modeling is to include only climatic variables; however, our preliminary research showed that the inclusion of more variables gives more specific results, with predictions increasing in accuracy with the addition of variables (data not shown). Thus, for modeling in this study, the following parameters were utilized: 19 bioclimatic variables^36^, 13 soil variables^37^, solar radiation, wind speed, and water vapor pressure^38^. The resolution and extent of all variables were adjusted to match bioclimatic data (30 arc seconds, or approximately 0.6 km^2^ in the study area). In order to preserve focus on algorithms and data sources, environmental variables with the same resolution were used for all analyses. We addressed collinearity, reducing the number of variables by 1) eliminating variables with zero and near-zero variance, 2) removing variables with linear dependencies (both steps were done using Caret 6.0–84 package for R^39^, and 3) running a PCA on all remaining variables. To select the most important PCA derived maps (i.e., PC axes) the Kaiser–Guttman criterion was used; it consists of taking the mean of all eigenvalues and then selecting only PC axes with eigenvalues larger than this mean^40^. Following this criterion, maps based on the first seven principal components were selected (Appendix 2). To emphasize intramountain differences, rather than differences between mountains and lowlands, we restricted the PCA to the area covering only the Carpathians, and then projected its results to the rest of the survey area. The Carpathians region was defined by division map^41^ restricted only to continuous areas above 400 m (approximate lower border of lower montane climatic belt)..

### Modeling settings

Modeling was conducted using the following algorithms available within the Dismo 1.1–4 package^42^: Bioclim^43^, Domain^44^, Mahalanobis^45^, Generalized Linear Models using Gaussian distribution “GLM”, Generalized Additive Model “GAM”^46^, Support Vector Machines “SVM”^47^, Random Forest “RF”^48,49^, Boosted Regression Trees “BRT”^50^, and Maximum Entropy (Maxent v. 3.4.1)^51^. Four additional algorithms were also used: Bayesian additive regression trees “BART”^52^, Gaussian processes with Bayesian priors “GRaF”^53^, Artificial Neural Network “ANN”^54^, and Classification Tree Analysis “CTA”^55^, the last two being executed through the biomod2 3.4.6 package^56^. We used the settings suggested by the vignettes accompanying each package except in the following cases: for Maxent, the maximum iterations were set to 10^4^, the convergence threshold to 10^−5^, and the output to clog-log; for “BART”, the number of tree models was set to 100 (“ntree = 100”) and burnin period to 20% (“nskip = 20”); for “GRaF”^57^ option “opt.l” was set to “TRUE”, which optimized the lengthscale parameters for the model marginal likelihood.

### Background points

For the pan-Carpathian datasets^26,33^ and herbarium specimens, background points were selected from the previously defined region of the Carpathian Mountains restricted to areas above 400 m. For all local datasets (i.e., regional and country-level) the background points were restricted to a 50 km buffer around species occurrences (in this case without elevation threshold). The number of background points varied depending on the algorithm and was set according to suggestions from^58^; the applied settings are presented in Appendix 3. In cases where a smaller number of points was required, they were randomly drawn from the initial set for each replication.

All GIS operations were done in R^59^ and QGIS^60^, except for georeferencing, which was done using ArcGIS v. 10.2 (ESRI, USA).

### Model evaluation

To evaluate the model outputs we used the following metrics available within R: Area under the ROC curve “AUC”, Root Mean Squared Error “RMSE”, Accuracy, Bias, Mean Absolute Error “MAE”, Sum of Squared Errors “SSE” from package Metrics^61^, Expected Calibration Error “ECE”, Maximum Calibration Error “MCE” from package CalibratR^62^, unweighted Kappa statistic, Sensitivity, Specificity, Positive Predictive Value “PPV”, Negative Predictive Value “NPV”, Precision, Recall, F1, Prevalence, Detection Rate, Balanced Accuracy from package Caret^39^, and Boyce Index “CBI” from package Ecospat^63^. Additionally, according to the equations in^24^ we calculated the following: True Skill Statistic “TSS”, Jaccard's Similarity Index, Sørensen's Similarity Index, Overprediction Rate “OPR”, and Underprediction Rate “UPR” (Appendix 4). If binary input was required to calculate an evaluation metric, continuous model outputs were transformed using the maximum specificity and sensitivity threshold.

For evaluation, we used a separate ground-truthing dataset collected using a GPS device (GPSMAP 62 s, Garmin) from 2016–2019. After adjustment to contain only one point per grid cell, this dataset consisted of 90 presences and 4841 absences. This dataset was collected by KK in tandem with a phylogeographical study, and encompassed all mountain subranges within the Carpathian and Vranica Mountains with known occurrences of the focus species. Surveys were made along roads and mountain paths, and effort was made to collect occurrences that minimalize the bias along the environmental variables used in the modelling. The mean distance between the nearest neighboring points across the Carpathians was 17 km but sampling was denser in some regions (especially within Western Carpathians with a mean distance of 2 km). All presence/absence data were collected while walking or driving at a low speed from May to September, which is the flowering period for *L. rotundifolium*. The species’ flowering period is quite long, with the first flowers appearing in late spring, peak flowering occurring in July, and sporadic flowering as late as the end of September. Due to the white ray florets typical of all species of the *Leucanthemum* genus, the flowers are very easy to spot in the field, even from a distance. Additionally, with some experience, it is possible to distinguish *L. rotundifolium* from related species because of its flowers’ lighter shade of white. *L. rotundifolium* also possesses various unique morphological characteristics (orbicular-ovate basal leaves, lanceolate stem leaves) that make it easy to differentiate from other related species. Taken together, these properties provide a higher chance of avoiding imperfect detection and its impact on model evaluation^64^.

One of our central aims was to choose the metrics best suited to assessing models’ performance. To make the pertinent comparisons, we computed an independent validation of presence-only models based on the above-mentioned ground-truth presence/absence data and visual rating (expert score). This was done by KK, who was responsible for collecting the species throughout its distribution and has worked specifically with *Leucanthemum* since 2009. First, a visual assessment for positive prediction in five predefined regions (Western, Eastern, and Southern Carpathians, Apuseni Mountains, Vranica Mountains) was made and each model was assigned a score of 0 for poor and 1 for good predictions (with intermediate scores possible). For example, a score of 1 was assigned if the species was known to occur in the region and the model identified it, and if the model targeted montane ecosystems. A lower score was assigned when the region was known to be inhabited by the species but not predicted by the model as suitable, or if the area was over- or underpredicted. In the same way, overpredictions were assessed within five regions (West, North, East, South of Carpathians, and Pannonian Basin) in which the species was known to be absent, assigning a score of − 1 if the model was extrapolated and a score of 0 if the specificity was high and no areas outside the range of the species were predicted (see Appendix 5 for a locator map of place names referred to in the text). Therefore, considering all regions in the first evaluation, the best model was given five points, while in the second the worst model received minus five points. To differentiate models with the same scoring, we added an additional score calculated as follows. We treated the absences and presences recorded with a GPS device as ground-truth. Therefore, we expected that every model would give a probability of occurrence of 1 at a presence point and a probability of occurrence of 0 at an absence point. We centered and scaled all predictions from the models on a 0–1 scale and recorded the scores that were given to presence points (*x*_*pres*_) and absence points (*x*_*abs*_). We modified the equation for standard deviation to calculate the deviation from the expected score: $1 - \left( {\sqrt {\frac{1}{N}\mathop \sum \limits_{1}^{N} \left( {x_{pres} - 1} \right)^{2} } + \sqrt {\frac{1}{N}\mathop \sum \limits_{1}^{N} \left( {x_{abs} } \right)^{2} } } \right)$. These scores were recorded separately for presences and absences within five predefined regions (Western, Eastern, Southern Carpathians, Apuseni Mts., Vranica Mts.), similarly to the method used for visual assessment. Then all results were added together (expert score for positive prediction, expert score for overprediction, deviation from the expected score) and a higher score assigned to better models.

The strength of the correlation between ground-truth evaluation and other metrics was assessed using Spearman's rank correlation coefficient. To show which suite of metrics agreed with our evaluation and should be used for model verification, multiple linear regression was calculated between ground-truth evaluation and other metrics. To reduce collinearity and eliminate redundant information prior to calculation, all metrics with Spearman's *ρ* above 0.8 were removed, and within pairs, the metric with higher correlation to the result of ground-truth evaluation was retained. After that, all metrics which showed a Variance Inflation Factor above 5 were deleted. We selected a final model based on the minimal Akaike information criterion (AIC) in the best subset method available within R package olsrr^65^.

Finally, a clustering analysis was used to compare the spatial predictions from the models generated from all combinations of occurrence datasets and modeling algorithms, and identify groups of models producing similar results. To enable comparison, model outputs were scaled and standardized. The Euclidean distance was then used to estimate the pairwise dissimilarity between model results, which was used as the input to clustering analysis. To select the most appropriate clustering method and number of clusters, we used Silhouette Width and Dunn Index, available in R package clValid^66^.

## Results

### Georeferencing

Georeferencing of hardcopy maps and herbarium specimens yielded satisfactory results. We obtained 49 to 390 locations for point datasets, and 137,166 for the species range converted to points. After adjusting the occurrences to raster resolution, those numbers were further reduced as shown in Table 1. The variation between different datasets was significant in terms of number, covered environmental space, and exactness (Fig. 2). In general, smaller datasets were locally more accurate, but contained fewer points and characterized a smaller portion of the environmental space (Fig. 2a). In contrast, larger datasets contained more points, but were less specific in smaller regions (see example in Fig. 2b,c). It may be seen from this example that large datasets often miss the exact point where the species occurs; for example, Fig. 2c shows that *L. rotundifolium* according to the local survey (dataset 2) occupies a valley, while datasets 4, 5, and 6 indicate only a summit, and the whole area is depicted by datasets 3 and 7. Naturally, field sampling in mountainous areas is often difficult, and complete penetration of all possible places is usually impossible. Sampling is often restricted by natural obstacles (inaccessible slopes or other terrain features) or by laws which prohibit entry to areas of special interest (strict reserves, animal breeding sites). Also, surveying only tourist routes would potentially bias the results. For datasets originating from floristic surveys (1, 2), the authors published charts showing all paths which were surveyed. In general, the charts of both surveys indicated the effect of the above-mentioned issues, but no strong bias was noted. Datasets 4, 5, and 6 were based on herbarium collections, and though they encompassed the entire distribution area, they were considerably denser in Poland and Slovakia than in Ukraine and Romania. Dataset 7 encompassed too large an area, in some cases extending far beyond any known occurrences.

### Predictions

From the 126 models produced, those that were either too narrow or too wide (under- and overpredicting, respectively) were readily eliminated. However, it was more difficult to rank those that reconstructed a reliable distribution but varied slightly, e.g., a model that performed well in one mountain range but slightly worse in a different area. An interesting result of this study was that many datasets, even local datasets, were able to correctly reconstruct the distribution (Appendix 5). Considering the ground-truth evaluation, two of the best predictions were derived by ANN from occurrences provided from the georeferenced map of the Carpathian Mts. (Carpathians, 5: Expert score 6.22) and georeferenced herbarium specimens (herbarium, 6: Expert score = 5.68). In general, the localized datasets (Gorce Mts., 1: Expert score = average 0.54 ± 1.81; Bieszczady Mts., 2: Expert score = 1.16 ± 2.03) performed worse than the datasets with full coverage (Carpathians, 5: Expert score = 2.14 ± 2.36; herbarium, 6: Expert score = 2.94 ± 2.31). The performance of full-coverage point datasets was also better than that of a broad dataset derived from a map of distributional range (range map, 7: Expert score = 1.43 ± 2.05) or atlas quadrats (atlas quadrats, 3a: Expert score = 0.51 ± 1.90). Medium-sized datasets derived from country inventories also attained good scores (atlas centroids, 3b: Expert score = 1.87 ± 2.38; Slovakian map, 4: Expert score = 2.18 ± 1.99). However, the outputs varied not only according to the dataset, but also according to the model chosen, with some algorithms performing better for one dataset than for another. In general, the algorithms that performed well across different datasets were BRT (Expert score = 3.88 ± 0.89), CTA (Expert score = 3.63 ± 1.26), ANN (Expert score = 3.63 ± 2.05), and BART (Expert score = 3.44 ± 0.9). Those of overall medium performance included Maxent (Expert score = 2.57 ± 1.51), Bioclim (Expert score = 2.21 ± 0.63), GRaF (Expert score = 1.7 ± 2.32), and RF (Expert score = 1.61 ± 1.11). The worst predictions across different datasets were those constructed by GAM (Expert score = 0.89 ± 1.03), GLM (Expert score = 0.46 ± 0.98), SVM (Expert score =  − 0.41 ± 1.5), Domain (Expert score =  − 0.65 ± 1.26), and Mahalanobis (Expert score =  − 2.21 ± 0.33) (Fig. 3).

**Figure 4:**
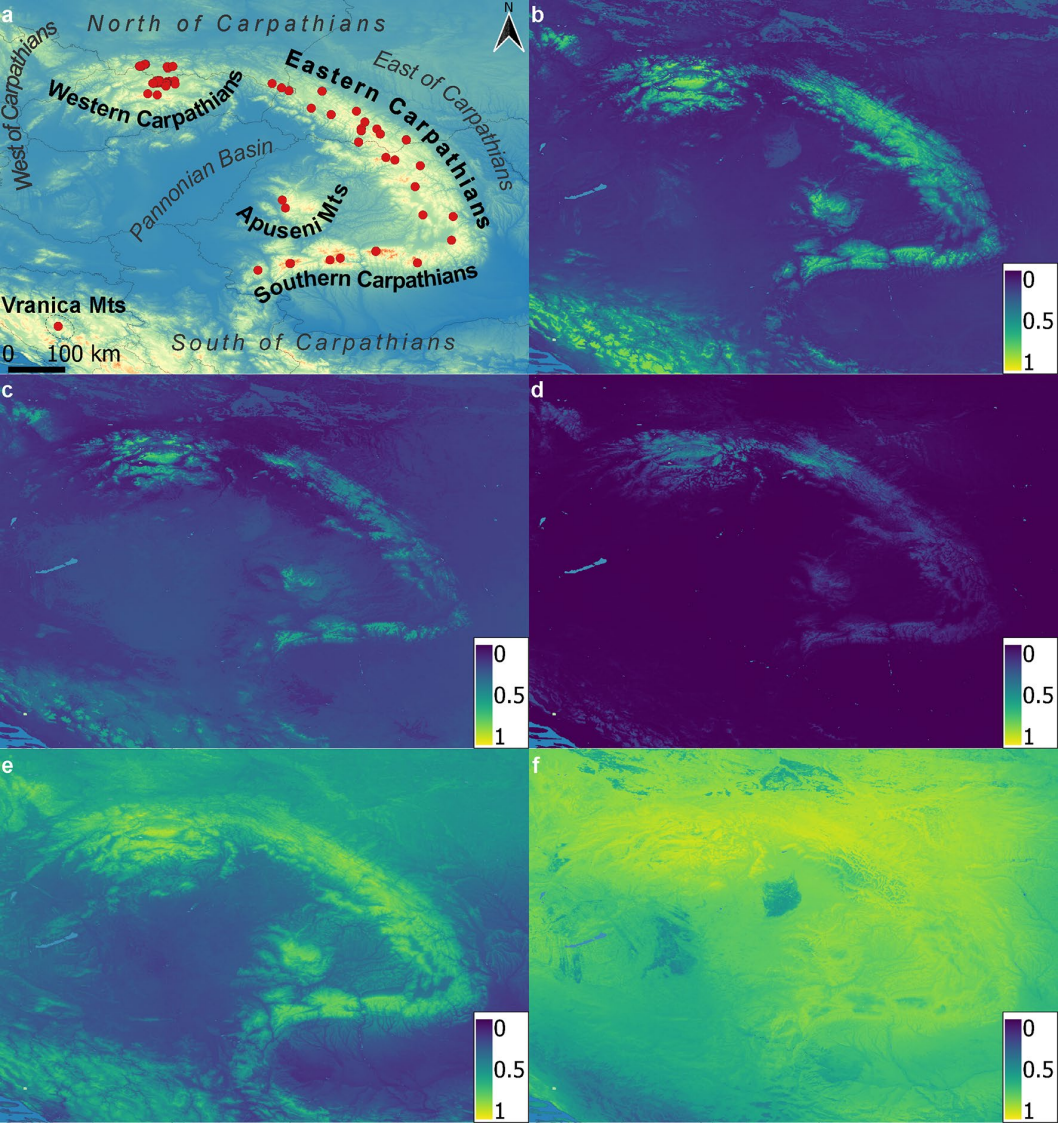
Spatial representation of clustering results; (**a**) regions of the Carpathian Mts. discussed in the text and used for ground-truth evaluation. Red dots show presence locations gathered with GPS device (2016–2019); (**b**) mean of models assigned to cluster A1; (**c**) mean of models assigned to cluster A2; (**d**) mean of models assigned to cluster A3; (**e**) mean of models assigned to cluster B1; (**f**) mean of models assigned to cluster B2. Colors in (**a**) represent altitude while colors in (**b**–**f**) represent inferred habitat suitability. The underlying image in (**a**) represents altitude derived from ALOS DEM dataset^34^.

Hierarchical clustering, which was selected as the most appropriate clustering method, divided the models into two strongly supported groups (A and B). The next-best division was into five groups (A1–A3 and B1–B2, Fig. 4 and Appendix 6). The main influencing factor on grouping was the coverage of the model. As is visible in Fig. 4, cluster A1 grouped models predicting suitable habitat across the entire Carpathian range, and may be regarded as the cluster containing the set of the most probable predictions. Cluster A2 similarly predicts the entire plausible habitat range, but higher suitability was assigned to the northern parts of the Carpathians (influenced by denser sampling). A similar but stronger pattern is visible in cluster A3, in which the southern parts are weakly supported as plausible, while the northern parts are assigned higher suitability. Group B grouped models that overestimate potential distribution; cluster B1 consists of models predicting the Carpathians and adjacent areas, while models belonging to cluster B2 are those strongly overestimating possible distribution in adjacent areas and towards the north. The groups do not follow any particular trend regarding algorithms except in a few cases (e.g., Group B was dominated by the results of Bioclim, Mahalanobis, GAM, and Domain). Clustering according to dataset is slightly more pronounced than clustering according to algorithm, and often the results of particular datasets are grouped (e.g., datasets 4, 6, and 7). The majority of models produced with datasets 3b, 5, 6, and 7 were placed in the preferred cluster (A1). Similarly, the majority of models constructed using ANN, BART, BRT, GRaF, and RF algorithms were placed in this cluster as well.

### Model evaluation

Results obtained from evaluation metrics are presented in Appendix 7. In general, the correlation between different metrics and ground-truth evaluation was weak (Appendix 7, Fig. 3). The three metrics that achieved the highest correlations were AUC (Spearman's *ρ* = 0.51), TSS (Spearman's *ρ* = 0.48), and MAE (Spearman's *ρ* =  − 0.41). Some metrics were highly correlated with each other, e.g., AUC, TSS, NPV, Recall, Detection Rate, and UPR, and MAE, ECE, RMSE, and SSE. The other two closely related groups were 1) metrics associated with false negatives (FN): AUC, Sensitivity, NPV, Recall, Detection rate, Balanced accuracy, TSS, and UPR, and 2) metrics associated with true positives (TP): NPV, Sørensen's Similarity Index, F1, Jaccard's Similarity Index, Kappa, Precision, PPV, Detection rate, Recall, and Sensitivity (Appendix 8). As is apparent from the results, relying on only one metric would be misleading, since individual metrics often tend to select models that perform poorly (i.e., reconstruct distribution only partially) while still meeting the criteria of the evaluation. This was the case for MAE, which consistently placed high value on models produced by BIOCLIM and GLM, which were unacceptable from our perspective because they missed a large portion of the range (Fig. 3d). This issue is also visible in the case of AUC and TSS, which favored models with a larger number of points (Fig. 3b). Thus, the best option when evaluating a model is to utilize an expert opinion assisted by multiple metrics. Such an approach is depicted in Fig. 3e,f, in which the best models (i.e., those that received high ground-truth evaluation and high metric score) are shown in the upper right corner, or in Fig. 6, in which AUC and MAE are used simultaneously to select the best model.

Multiple linear regression analysis was used to test if any metrics significantly predicted ground-truth evaluation ratings of model performance (Table 2). Breusch–Pagan test indicated that the data were homoscedastic. The results of the regression indicated that the four predictors explained 26% of the variance (R^2^ = 0.2604, F(4, 99) = 10.07, *p* = 7.051 × 10^−7^). It was found that AUC significantly predicted ground-truth evaluation score (β = 0.44, *p* < 0.001), as did MAE (β =  − 134.4, *p* < 0.05) and Bias (β = 134.3, *p* < 0.05).

**Table 2:** Results of multiple linear regression; F = 10.07; df (4, 99); R^2^ = 0.2604; *p* = 7.051 × 10^−7^.

Explanatory variable	Estimate (β)	SE	t value	*p* value
(Intercept)	− 1.38 × 10^−15^	0.08	0	1
Accuracy	0.1662	0.09	1.92	0.0577^NS^
AUC	0.4364	0.09	4.886	3.97 × 10^−6^***
Bias	134.3	60.06	2.236	0.0276*
MAE	− 134.4	60.06	− 2.238	0.0275*

Symbols indicate: ***Significant at 0.001; *Significant at 0.05. *NS* not significant.

## Discussion

### Utility of ENMs using different occurrence datasets and modeling algorithms

The central result of this study is that all point datasets achieved acceptable accuracy in at least one model (Fig. 3) and may be used for reconstruction of species distribution, although the coverage of the dataset and applied algorithm strongly affect the model output. The two main uses of ENM produced from historical data such as hardcopy maps or georeferenced herbarium specimens are 1) finding new locations of a species of interest and 2) reconstructing its ecological niche.

**Figure 5:**
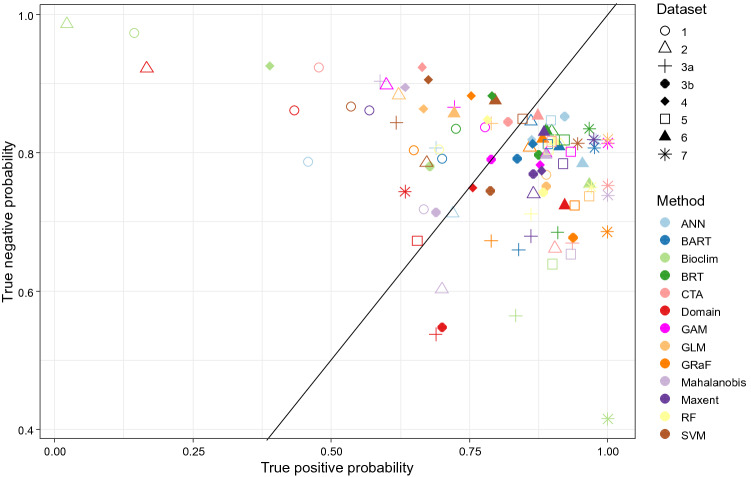
Estimates of true positive (TP) and true negative (TN) classification probability rates for different algorithms and datasets. TP and TN are derived from data collected in the field using a GPS device. The line shows where TP is equal to TN, i.e., the best model correctly classifying TP and TN should be placed in the upper-right corner of the graph.

When the aim is to use environmental niche modeling to find new locations of the species (i.e., maximize true positives, Fig. 5), most of the datasets, even those with limited extent, would be sufficient to pinpoint additional areas where species may be searched for. The same is true for many algorithms, many of which, according to our field survey and ground-truth data, would yield useful results. In the case of small datasets (1 and 2), the algorithms that performed well included BRT and BART, while ANN, CTA, MAXENT, and RF showed good performance in only one of the datasets. Based on this, we can recommend BRT and BART for small datasets, but as may be observed, the outcome is variable, and it is advisable to try other options. It is also interesting that in some cases, algorithms that used limited datasets failed to predict a suitable habitat outside a given mountain range, but performed well within a smaller area, as was the case for GRaF and Maxent algorithms when applied to the Gorce Mts. (1) dataset. When performance across all datasets is considered, BRT, ANN, CTA, and BART were the best performing algorithms. However, it should be noted that even among these algorithms, some performed better on one of the datasets than another; for example, ANN performed better with country-level or general datasets (3b, 4, 5, and 6) than with localized datasets and the range map (1, 2, 3a, and 7).

Another interesting point is that models developed from a full range map (7), which in this case was very broad, were capable of giving a general indication of where a species might be found, with the resulting models having lower spatial generalization and more refined detail than the original range map. Clearly, in this case, the model is reconstructing the distribution of the main habitat types present within the range, but this information may still be helpful for cases in which knowledge of species distribution is very general. Altogether, the results obtained highlight that the outcomes of environmental niche modelling are valuable source of data for biodiversity studies and conservation planning, even for species with incomplete distribution data^67^.

If the aim of using ENM is not only the identification of new locations of a species, but also the reconstruction of its niche, reliance on datasets with small coverage is not recommended. At a minimum, country-level occurrence data that represent a wide spectrum of possible environmental conditions or cover significant portion of a species range should be used. Datasets of greater coverage spanning the whole ecoregion or several countries are recommended. If such dataset is not available, several datasets should be combined to form a more complete final dataset. When available, georeferenced maps (in this study, datasets 1, 2, 3, 4, and 5) could be combined with herbarium localities (here, dataset 6). However, this should be done only if the datasets have good quality or if a rating of accuracy is assigned to each point (the accuracy of georeferenced herbarium specimens especially needs to be considered). Moreover, none of the joined datasets should have coarser resolution than the variables used for modeling^68^, which would exclude, for example, datasets based on atlases. Pooling may present advantages, as relying on only one source of data (especially very local or sparse data) may produce a biased outcome^69^. After combining datasets, they should be examined for autocorrelation and uneven sampling. One solution to these problems is to reduce geographical bias before modeling^10,70,71^. A similar approach is environmental filtering of data, which has been applied even more often and with greater success^5,8,72,73^. Both approaches reduce sampling bias by rarefying occurrences according to a given criteria. Another possible solution is to assign accuracy to data points and then build a model that takes these assignments into account and is capable of excluding questionable sites^74–76^. Other methods that consider spatial relationships exist, but their usage is not as straightforward as those presented here, as they require specifying further assumptions. Nevertheless, as these methods continue to be refined, they may prove to be useful, as demonstrated in some papers, e.g.^77–79^. In attempting to reconstruct the ecological niche of a species, identifying the variables influencing the model is often of interest, as many algorithms offer quantification of variable importance. This is achievable with distributional data covering the entire range of a species, but caution should be used if the dataset is incomplete or biased, as estimation of importance of variables is influenced by sample size and niche characteristics of the species^80^.

### Recommended method for proper evaluation of models

In our case, the studied plant is endemic, with a known distribution and minimal/maximal vertical extent; thus, the predictions were judged by the authors visually and using ground-truth evaluation. However, due to the fact that human-assisted evaluation is subjective and may be misleading, it is very useful to be able to find a metric that will perform this task objectively and automatically. As shown in the results, relying on only one evaluation metric leads to poor results. Therefore, we recommend using and reporting more than one metric, and if possible, checking models against known distributions and knowledge about species requirements. In our case, the metrics that showed the highest agreement with the ground-truth evaluation were AUC, TSS, MAE, Bias, and ECE. When using datasets with small spatial coverage, care must be taken to provide an adequate number of samples for evaluation, as metrics may be dependent on sample size^81^. In our study, it was shown that AUC and TSS are higher in datasets containing more points. This pattern for these two metrics has been reported by previous studies^82^. Nonetheless, some other papers favor the use of TSS over AUC^83,84^, due to calculation assumptions. However, as shown by the results of our study and others, it is apparent that both TSS and AUC depend on sampling density in an approximately linear fashion^82,85–87^. Even though AUC has been criticized before, especially when applied to compare models with different areas or between different species^88–90^, it is probably the most widely-used metric to evaluate ENM, partially because it is a standard output of algorithms such as Maxent. We advocate its continued usage, but seek to raise awareness of its size dependency and the necessity of combining it with other metrics that are computed differently and are not correlated (excluding, for example, TSS or NPV which are highly correlated to AUC). Two other promising metrics are MAE and Bias. They were increased in datasets containing a small number of points, but otherwise would be very helpful in model evaluation and after exclusion of GLM and Bioclim algorithms, would pinpoint almost the same datasets as ground-truth evaluation. Careful examination of metrics is also recommended for ensemble modeling, in which metrics may be used to set the weight of the model during model averaging^91,92^.

**Figure 6:**
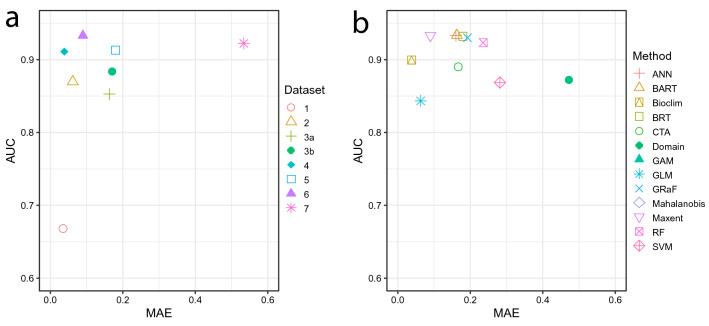
Example shows how AUC and MAE may be used simultaneously to select the best- performing datasets or algorithms: (**a**) selection of the best dataset for use with Maxent algorithm, (**b**) selection of the best algorithm for use with georeferenced herbarium specimens dataset (6). The best-performing dataset or model should appear in the upper left corner, i.e., have a high AUC and low MAE. In this example, the best datasets for Maxent are 4 and 6, while the best algorithm for dataset 6 is Maxent and Bioclim, or alternatively, one of the group consisting of ANN, BART, BRT, and GRaF.

To select the best-performing model or dataset, we propose the method of examining a graph in which the y-axis represents one metric and the x-axis represents another metric. Here, we focus on an example in which AUC and MAE are compared (Fig. 6). In this comparison, we look for the model which achieves the best values for both metrics, i.e., the highest AUC and the lowest MAE. We show an example including only one algorithm (Maxent) in Fig. 6a and one dataset (6) in Fig. 6b. In our example, the datasets achieving the best performance with Maxent are 4 and 6, while the best performing algorithm for dataset 6 is Maxent, followed by members of a group consisting of ANN, BART, BRT, and GRaF.

### Performance of ENMs based on maps versus herbarium data and associated problems

As has been stated, either hardcopy maps or georeferenced herbarium specimens may be used as a source of species occurrences for the modeling algorithm or they can complement each other. Nevertheless, certain issues are associated with both data types, which may limit their application.

Spatial accuracy is one of the main problems that may be encountered when georeferencing hardcopy maps and using them in species distribution modeling. In most cases, the coordinate system is not known, which may affect the placement of occurrences. Georeferencing also depends on the resolution of the underlying map, where the larger spatial exent and smaller illustration will give a less accurate georeferenced image^18^. Moreover, in such maps, where occurrences are marked with dots, the dots usually cover a large area. In the studied example, the scanned map of the entire Carpathian range (5) contained dots which each covered 186 km^2^, while dots on maps with smaller extents were more precise, i.e., the Gorce Mts. (0.24 km^2^) and the Bieszczady Mts. (0.49 km^2^). However, if the point is aligned with the center of the dot, the resulting mismatch in climate, for example, may be so small as to have no effect^17^.

Properly digitized maps may be more accurate than digitized herbarium occurrences because they have already been evaluated by another researcher. However, one disadvantage to maps is that they may contain errors caused by misplacement of points, similar to georeferenced herbarium specimens. Also, unlike herbarium specimens, distributional maps are often the products of regional surveys that do not document all collections as preserved specimens. Thus, it is not always possible to verify the data behind the map. For instance, in many locations surveyed in the Gorce Mts. (1)^30^ where *L. rotundifolium* was noted, we instead found *L. ircutianum* during field surveys; this may not necessarily indicate an erroneous identification, but nonetheless indicates the need for caution when working with such a varied genus as *Leucanthemum*. Additionally, if local adaptation exists within a studied species, using a reduced sample number confined to a small region may result in biased prediction^93^.

Problems associated with georeferencing herbarium specimens are frequent, since the listed locations are imprecise in most cases^18^. Often, only the name of a town or village is given as the place of collection, and sometimes place names are so ambiguous that they refer to an entire district or mountain range. For specimens collected in mountains, the most common collection error is to give only the name of the peak when most collections are actually made en route. This issue was encountered in the present study, as many herbarium collections contained only mountain names, while the typical habitat of the target species is specifically the upper montane-subalpine level. Georeferencing specimens named in this way is difficult; specimens may be placed in the wrong area altogether, or on the correct mountain but at an unsuitable elevation. Even if the specimen is placed on the correct mountain at an appropriate elevation, it is often unclear which side of the mountain it should be placed on, only one of which may be suitable. One possible way of alleviating this problem is to use variables with lower resolution, but even the best resolution of climatic data, which is around 1 km^2^ (30 arc seconds), may be considered coarse for many areas where step summits occur, i.e., there is a high chance of merging higher and lower elevations which may be strictly different (Fig. 2c). Another problem is finding the correct mountain; the Carpathians are inhabited by at least four language groups, and the name of the same mountain may appear several times in different ranges or even in the same range. These naming issues predominately arise from migration and settlement of the Vlach ethnic group, which has resulted in names derived from *muncel, chică*, or *măgură* sometimes reaching more than 30 derivatives throughout the whole range (e.g., Muńcuł (Polish), Minčol (Slovakian), Muncelului (Romanian), Meнчyл (Ukrainian), etc.). Lastly, the name of mountains may change over time, and the same place may be known by different names in the official and unofficial languages of a region. Issues with georeferencing herbarium specimens have been noted before (see^16–18,94^ for potential ways to alleviate them).

## Conclusions

As a result of the presented research, we conclude that one may obtain useful models of sufficient quality by using occurrence data from different portions of a species’ range, but that predictions may differ based on the applied algorithm and coverage of the dataset^20^. When possible, maps should be used as an addition to herbarium specimens, and in certain cases may be used as a standalone source. Emphasis should be placed on utilizing the maximum coverage of a species range, especially in the case of ecological niche reconstruction. After careful examination of several models, it should be possible to reconstruct the potential distribution and find new occurrences of a species, even if previous knowledge about the species range is not available. This highlights the usefulness of data from potentially biased sources, which may hold true for other organisms^73,93,95^. Exploration of different models is useful, as individual algorithms that fail to predict the entire species range may still predict well on a local scale. Finally, the results of this study strongly indicate against the use of a single metric in model evaluation, and supports the use of two or more uncorrelated metrics in assessing modeling outcomes.

## Supplementary Information

Supplementary Legends.

Supplementary Information 2.

Supplementary Information 3.

Supplementary Information 4.

Supplementary Information 5.

Supplementary Information 6.

Supplementary Information 7.

Supplementary Information 8.

Supplementary Information 9.
