# Supplementary material for: Evaluation metrics and validation of presence-only species distribution models based on distributional maps with varying coverage
Source: Sci Rep. 2021 Jan 15;11:1482. doi: 10.1038/s41598-020-80062-1 (PMC7811024; doi:10.1038/s41598-020-80062-1)
Supplement: Supplementary file 1 — Supplementary Legends. [file 41598_2020_80062_MOESM1_ESM.docx]

**Title**: Evaluation metrics and validation of presence-only species distribution models based on distributional maps with varying coverage

**Authors**: Kamil Konowalik^1*^, Agata Nosol^1^

^1^Department of Plant Biology, Institute of Biology, Wroclaw University of Environmental and Life Sciences, Kożuchowska 5b, 51-631 Wrocław, Poland

* author for correspondence: e-mail: [kamil.konowalik@upwr.edu.pl](mailto:kamil.konowalik@upwr.edu.pl)

**Appendix 1** Data used for modeling. The data points are thinned and aligned to cells of environmental rasters with a resolution of 30 arc seconds. The first column indicates the name of the dataset, and the second shows the assigned number that was used in the article.

**Appendix 2** PCA loadings for original variables. Maps derived from the seven principal components (shaded area) were used as input for modeling algorithms.

**Appendix 3** Modeling settings with specifications about the number of background points and repetitions.

**Appendix 4** List of applied evaluation metrics with an indication of source R package or equation.

**Appendix 5** Individual results from modeling algorithms. The first plate illustrates the area of the Carpathian Mts with main subdivisions and two other massifs where L. rotundifolium is occurring (the Apuseni and Vranica Mts). It specifies the regions used for expert evaluation (for details see the materials and methods). The underlying image represents the altitude derived from the ALOS DEM dataset. The following plates illustrate the results of modeling with different datasets and models. The name of the dataset and applied modeling algorithm appear in the lower-left corner. Numbers referring to datasets are compatible with Fig. 1 and Tab. 1. Algorithms shortcuts are explained in the material and methods.

**Appendix 6** Dendrogram of the hierarchical clustering aimed to group model predictions based on their similarity (using Euclidean distances and Ward method). The clustering divides the tree into two main groups: A and B. The next possible solution is a division into five groups: three within cluster A (A1, A2, A3) and two within cluster B (B1, B2). Numbers referring to datasets are compatible with Fig. 1 and Tab. 1. Algorithms shortcuts are explained in the material and methods.

**Appendix 7** A summary containing evaluation results for each dataset and algorithm. The table presents: predicted suitable area, ground-truth evaluation (expert score), and metrics used for evaluation. Correlation between ground-truth evaluation is presented at the bottom of the table.

**Appendix 8** Correlation matrix (Spearman rho) of evaluation metrics. Metrics that attained significant correlation (above 0.8 or below -0.8) are shown in red bold font and should not be used together for evaluation purposes. Caution should be taken regarding also other metrics with a correlation above 0.5 and below -0.5 (bold black font).
