# Supplementary material for: Evaluation metrics and validation of presence-only species distribution models based on distributional maps with varying coverage
Source: Sci Rep. 2021 Jan 15;11:1482. doi: 10.1038/s41598-020-80062-1 (PMC7811024; doi:10.1038/s41598-020-80062-1)
Supplement: Supplementary file 5 — Supplementary Information 5. [file 41598_2020_80062_MOESM5_ESM.docx]

**Appendix 4** List of applied evaluation metrics with an indication of source R package or equation.

| Name | Shortcut | R package or equation |
| --- | --- | --- |
| Accuracy | - | Metrics |
| Area under the ROC curve | AUC | Metrics |
| Balanced Accuracy | - | Caret |
| Bias | - | Metrics |
| Boyce Index | CBI | Ecospat |
| Detection Rate | - | Caret |
| Expected Calibration Error | ECE | CalibratR |
| Expert evaluation | - | $\sum_{region 1}^{region n} 1-\left( \sqrt{\frac{1}{N}\sum_{1}^{N} \left( x_{pres}-1 \right)^{2}}+\sqrt{\frac{1}{N}\sum_{1}^{N} \left( x_{abs} \right)^{2}} \right)$ |
| F1 | - | Caret |
| Jaccard's Similarity Index | Jaccard | $\frac{TP}{FN+TP+FP}$ |
| Maximum Calibration Error | MCE | CalibratR |
| Mean Absolute Error | MAE | Metrics |
| Negative Predictive Value | NPV | Caret |
| Overprediction Rate | OPR | $\frac{FP}{TP+FP}$ |
| Positive Predictive Value | PPV | Caret |
| Precision | - | Caret |
| Prevalence | - | Caret |
| Recall | - | Caret |
| Root Mean Squared Error | RMSE | Metrics |
| Sensitivity | Sens | Caret |
| Sørensen's Similarity Index | Sørensen | $\frac{2TP}{FN+2TP+FP}$ |
| Specificity | Spec | Caret |
| Sum of Squared Errors | SSE | Metrics |
| True Skill Statistic | TSS | $Sens+Spec-1$ |
| Underprediction Rate | UPR | $\frac{FN}{TP+FN}=1-Sens$ |
| Unweighted Kappa statistic | Kappa | Caret |

*TP – True Positives, FN – False Negatives, FP – False Positives
