# Supplementary material for: Evaluation metrics and validation of presence-only species distribution models based on distributional maps with varying coverage
Source: Sci Rep. 2021 Jan 15;11:1482. doi: 10.1038/s41598-020-80062-1 (PMC7811024; doi:10.1038/s41598-020-80062-1)
Supplement: Supplementary file 6 — Supplementary Information 6. [file 41598_2020_80062_MOESM6_ESM.pdf]

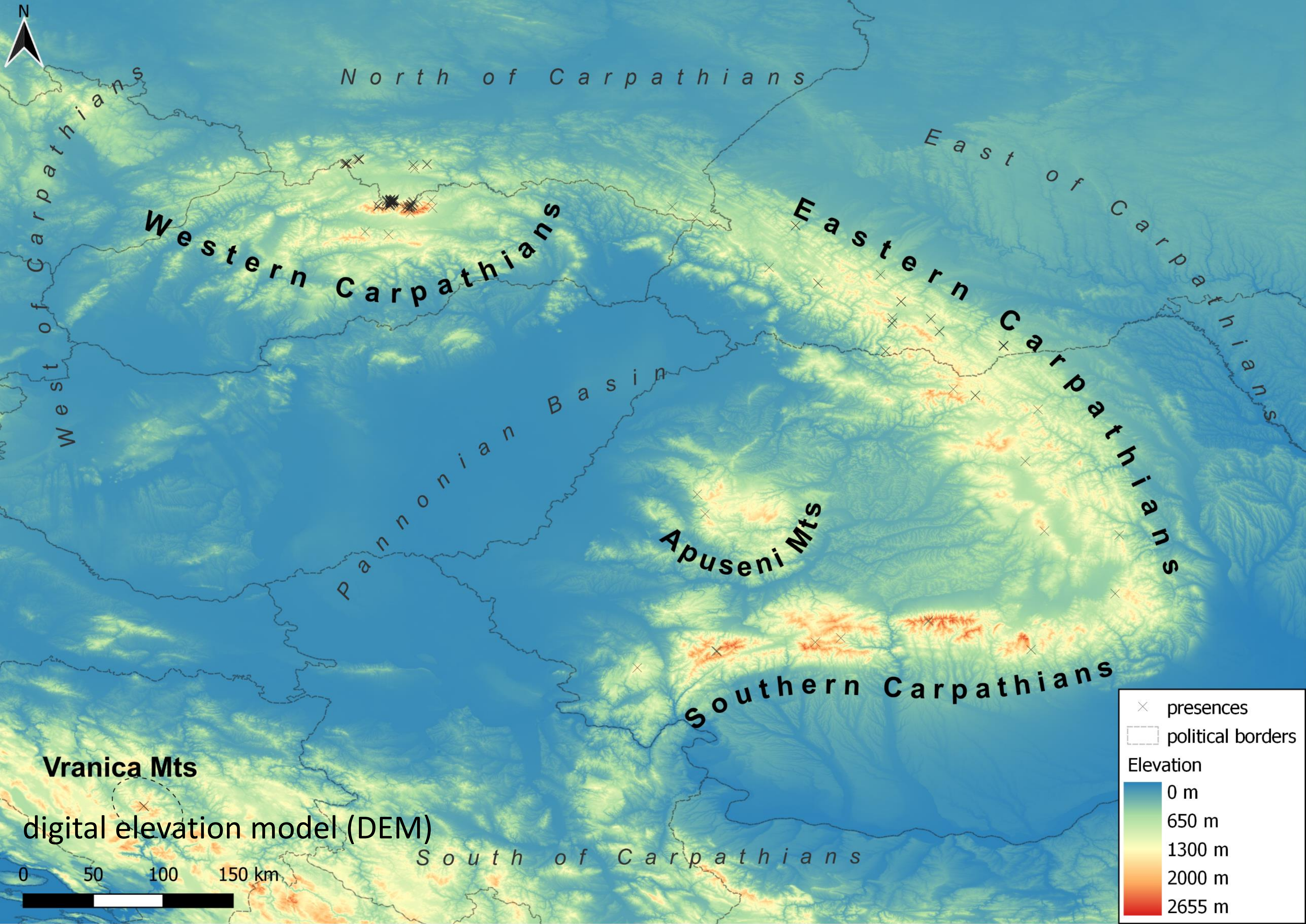
N
North of Carpathians
West of Carpathians
Western Carpathians
East of Carpathians
Eastern Carpathians
Pannonian Basin
Apuseni Mts
Southern Carpathians
Vranica Mts
digital elevation model (DEM)
South of Carpathians
0
50
100
150 km
presences
political borders
Elevation
0 m
650 m
1300 m
2000 m
2655 m

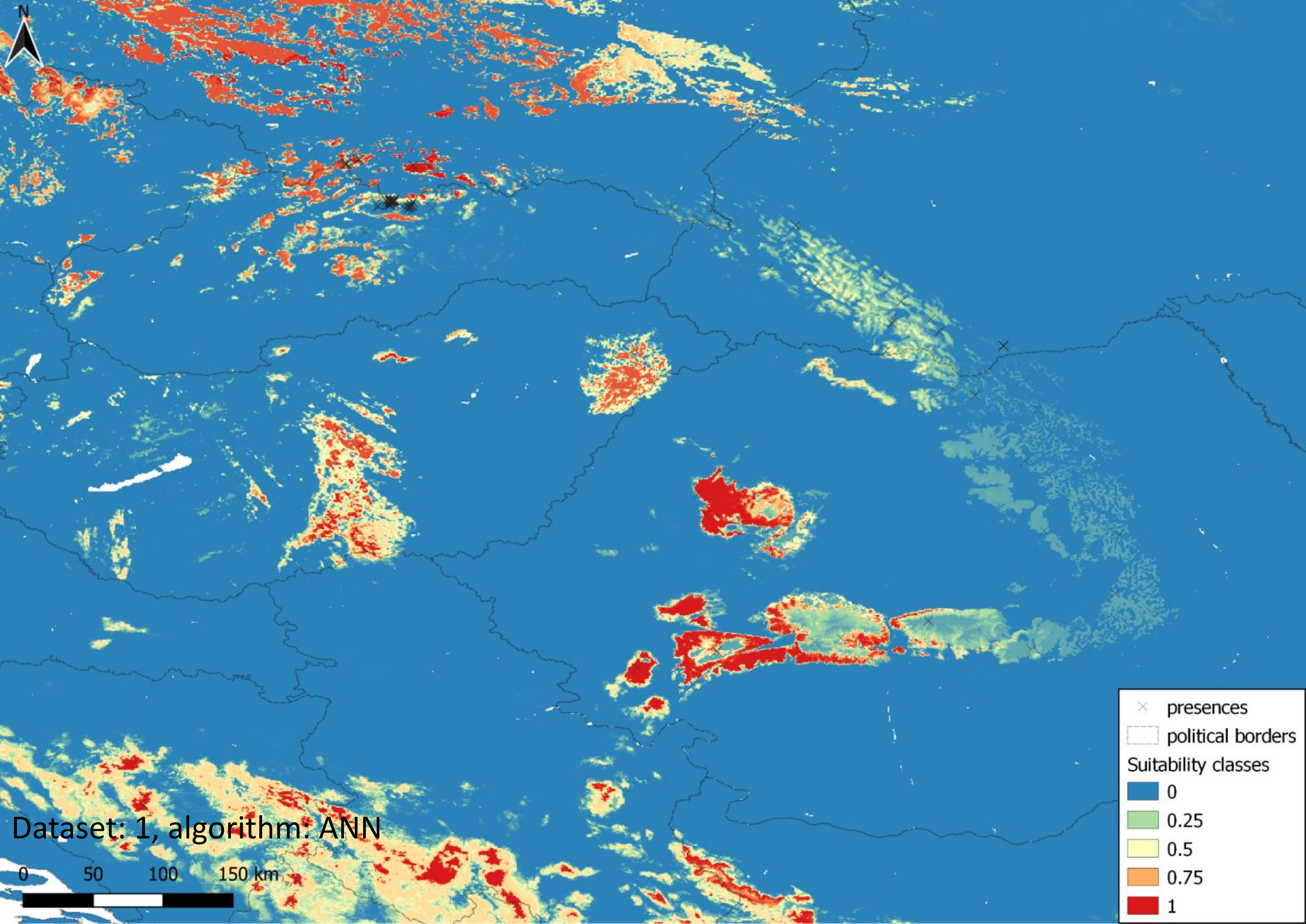

N
presences
political borders
Suitability classes
0
0.25
0.5
0.75
1
Dataset: 1, algorithm: ANN
0
50
100
150 km

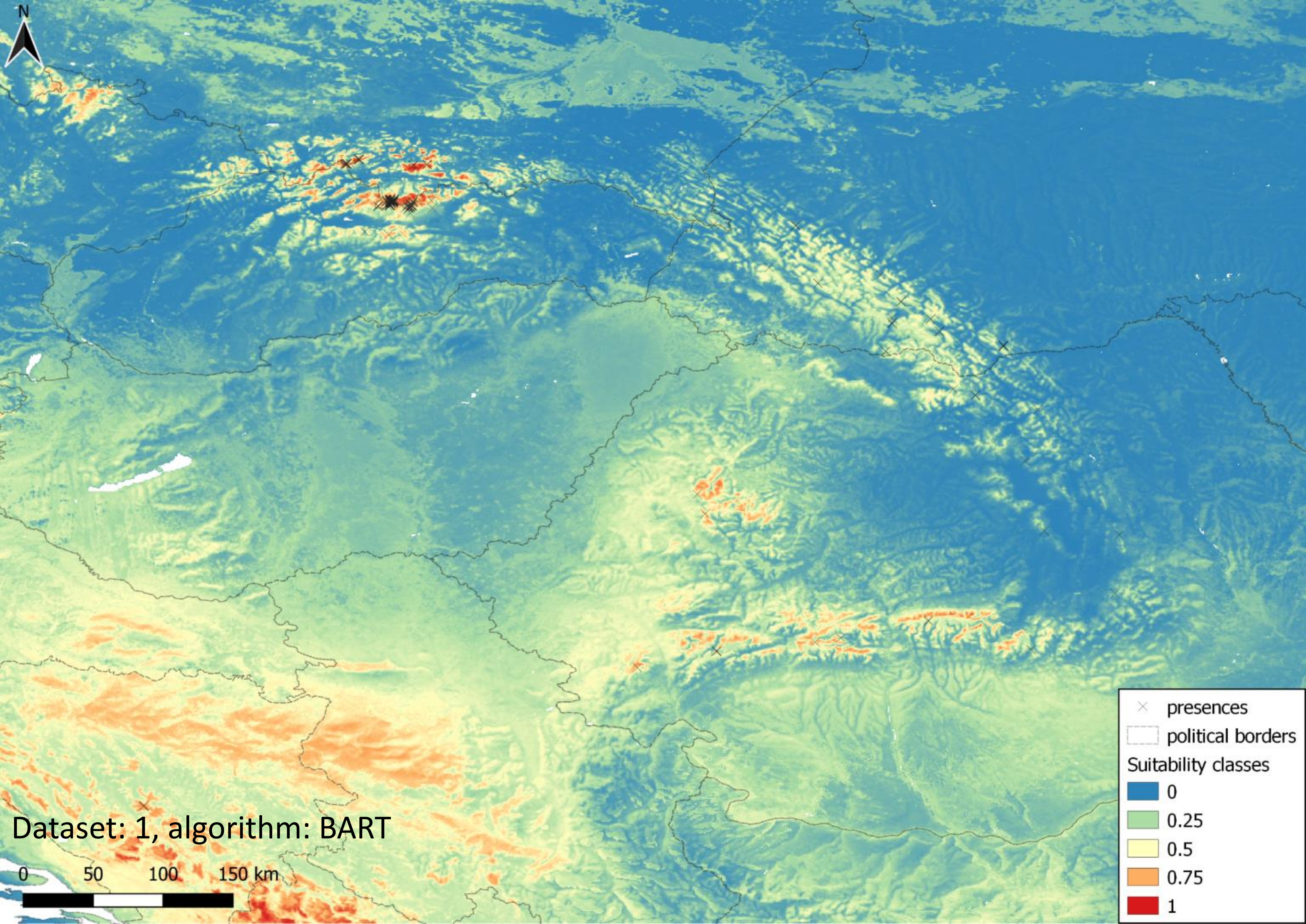
N
Dataset: 1, algorithm: BART
0
50
100
150 km
presences
political borders
Suitability classes
0
0.25
0.5
0.75
1

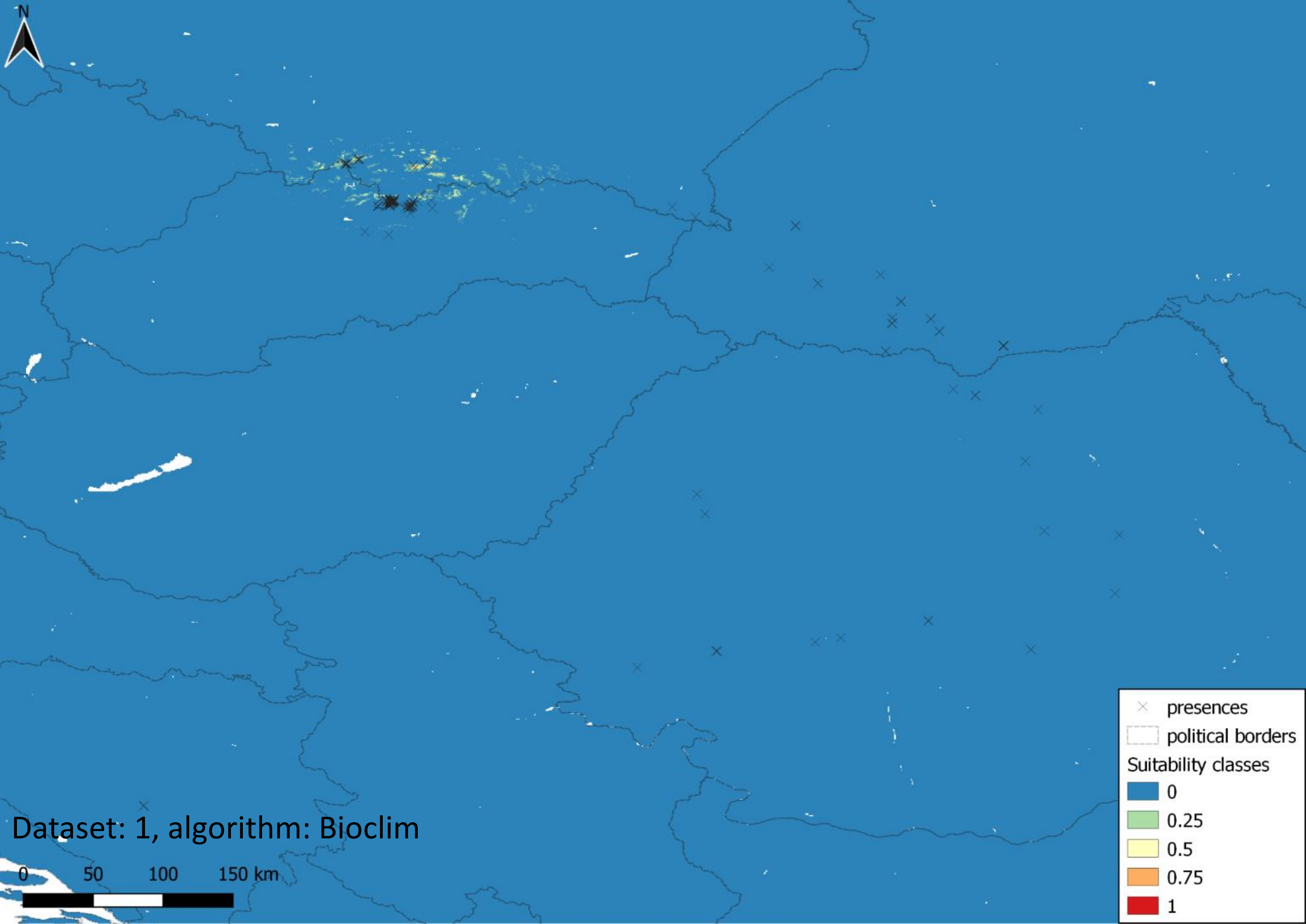
N
presences
political borders
Suitability classes
0
0.25
0.5
0.75
1
Dataset: 1, algorithm: Bioclim
0
50
100
150 km

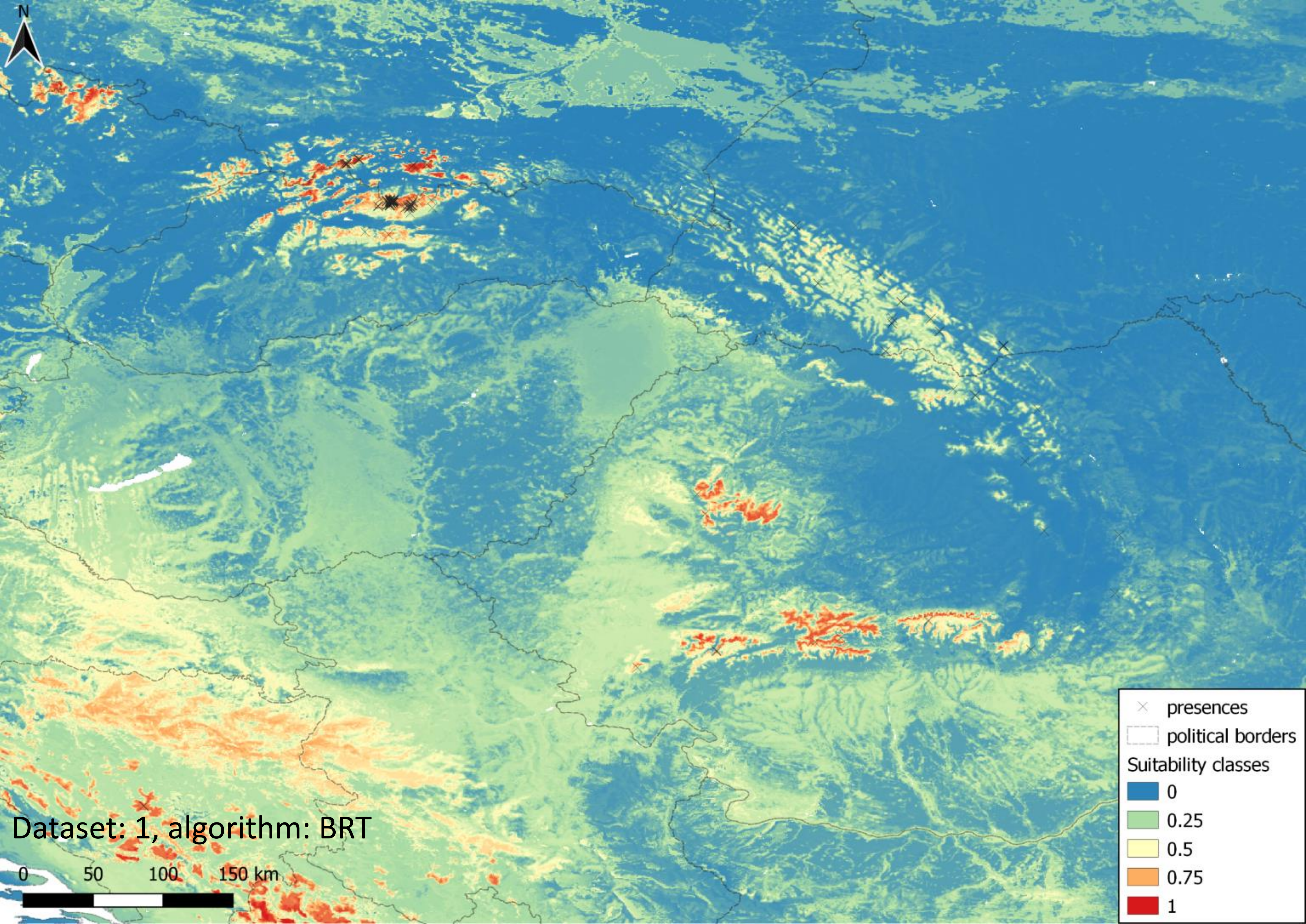
N
Dataset: 1, algorithm: BRT
0
50
100
150 km
presences
political borders
Suitability classes
0
0.25
0.5
0.75
1

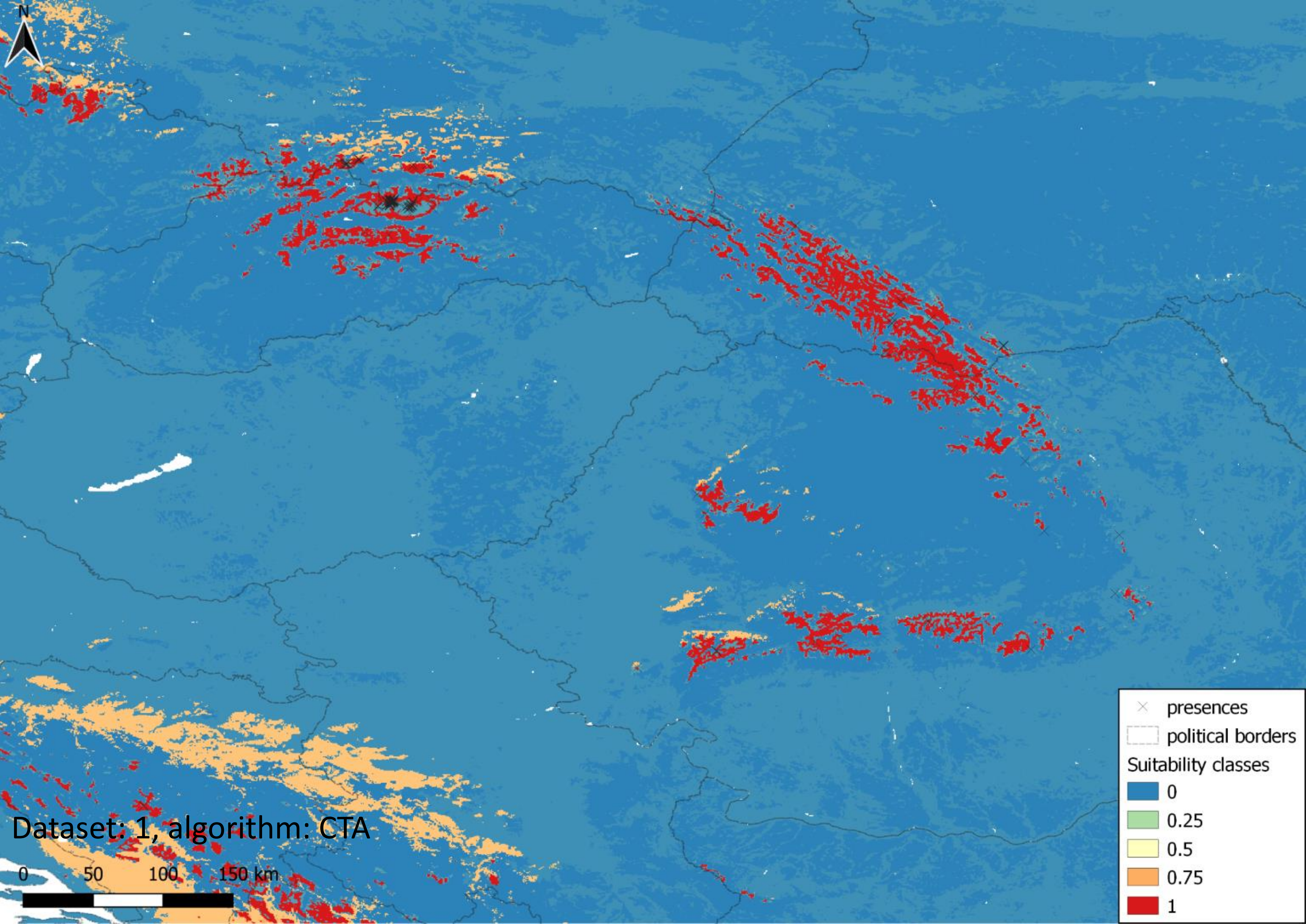
N
presences
political borders
Suitability classes
0
0.25
0.5
0.75
1
Dataset: 1, algorithm: CTA
0
50
100
150 km

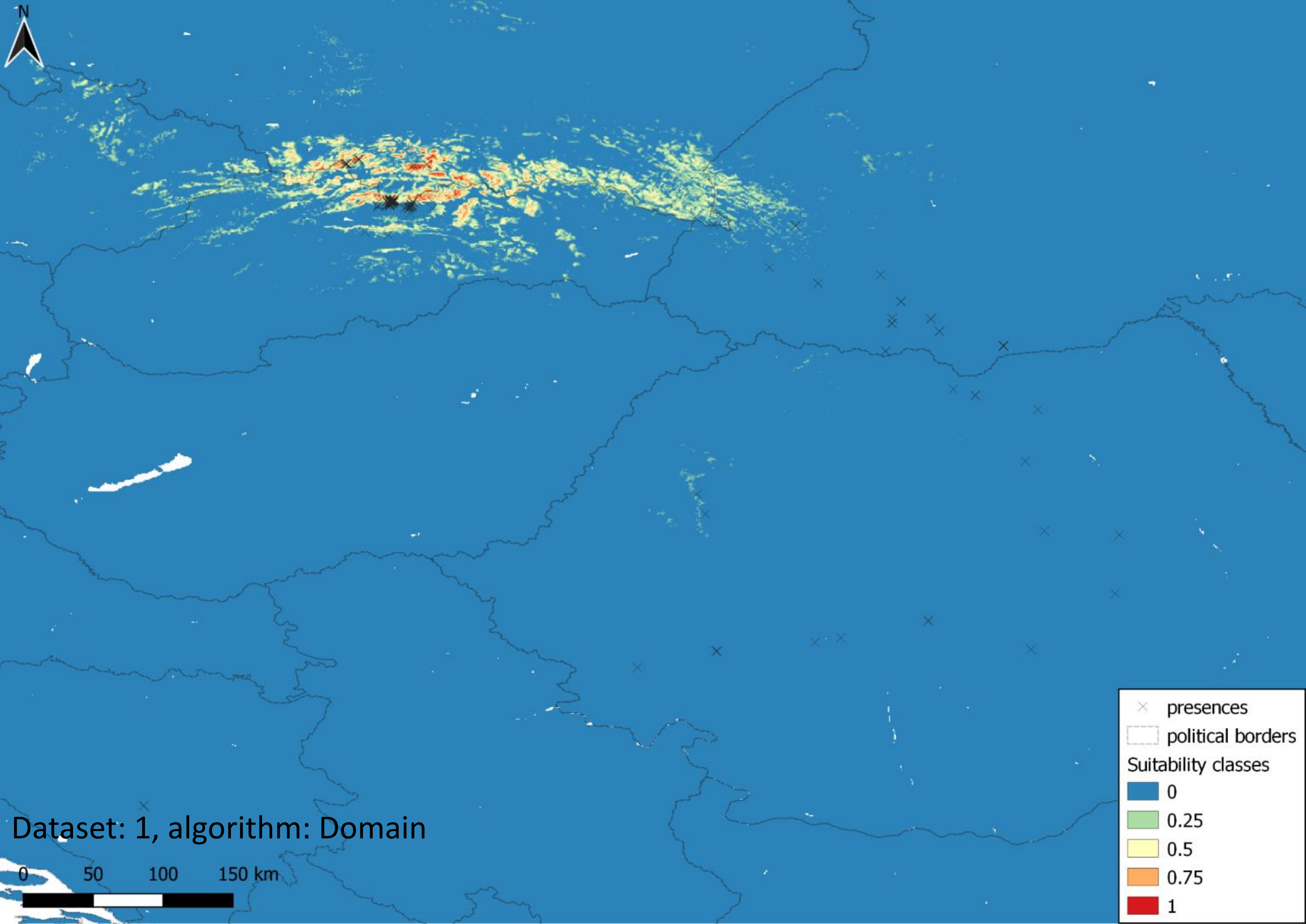
N
presences
political borders
Suitability classes
0
0.25
0.5
0.75
1
Dataset: 1, algorithm: Domain
0
50
100
150 km

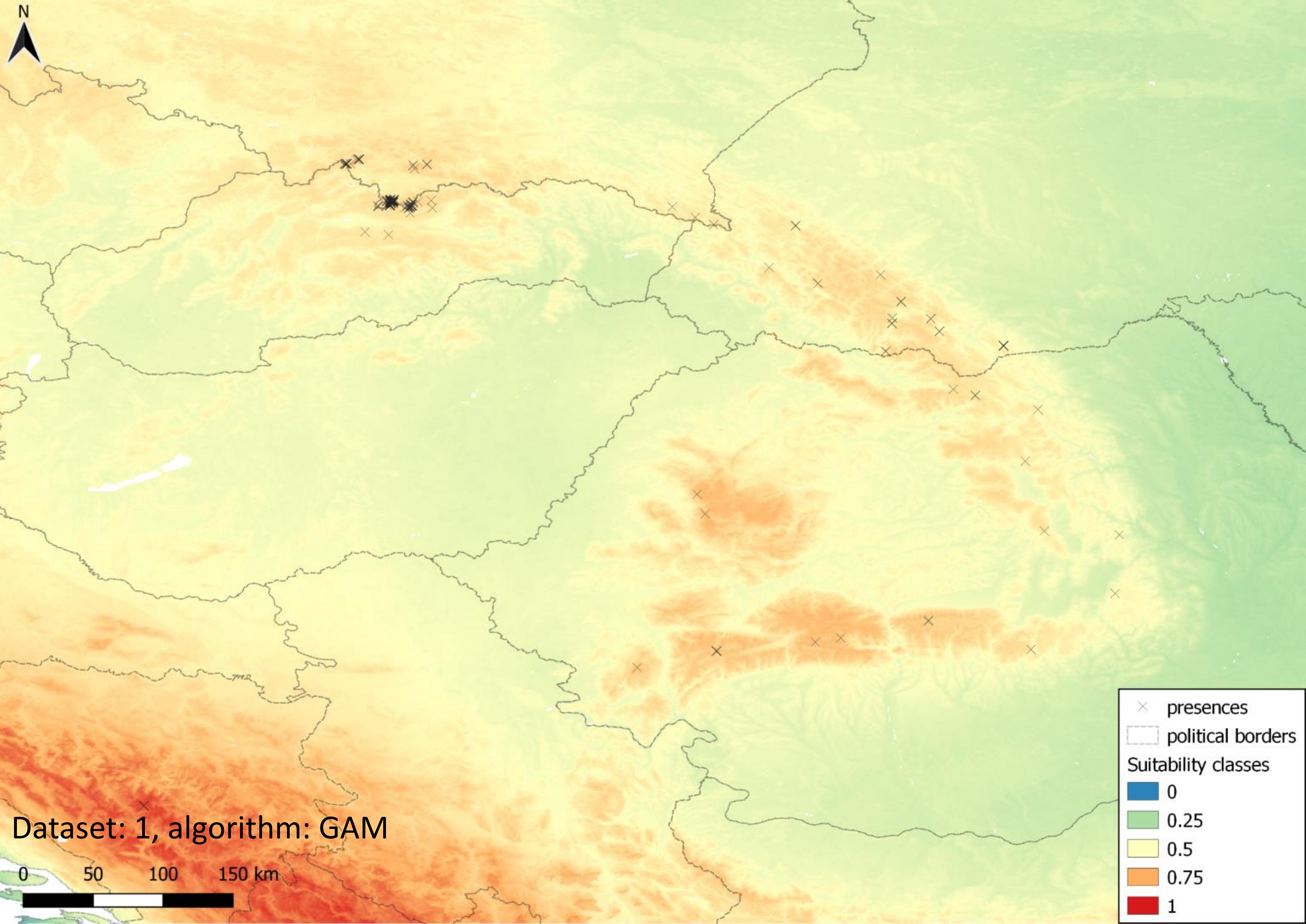
N
presences
political borders
Suitability classes
0
0.25
0.5
0.75
1
Dataset: 1, algorithm: GAM
0
50
100
150 km

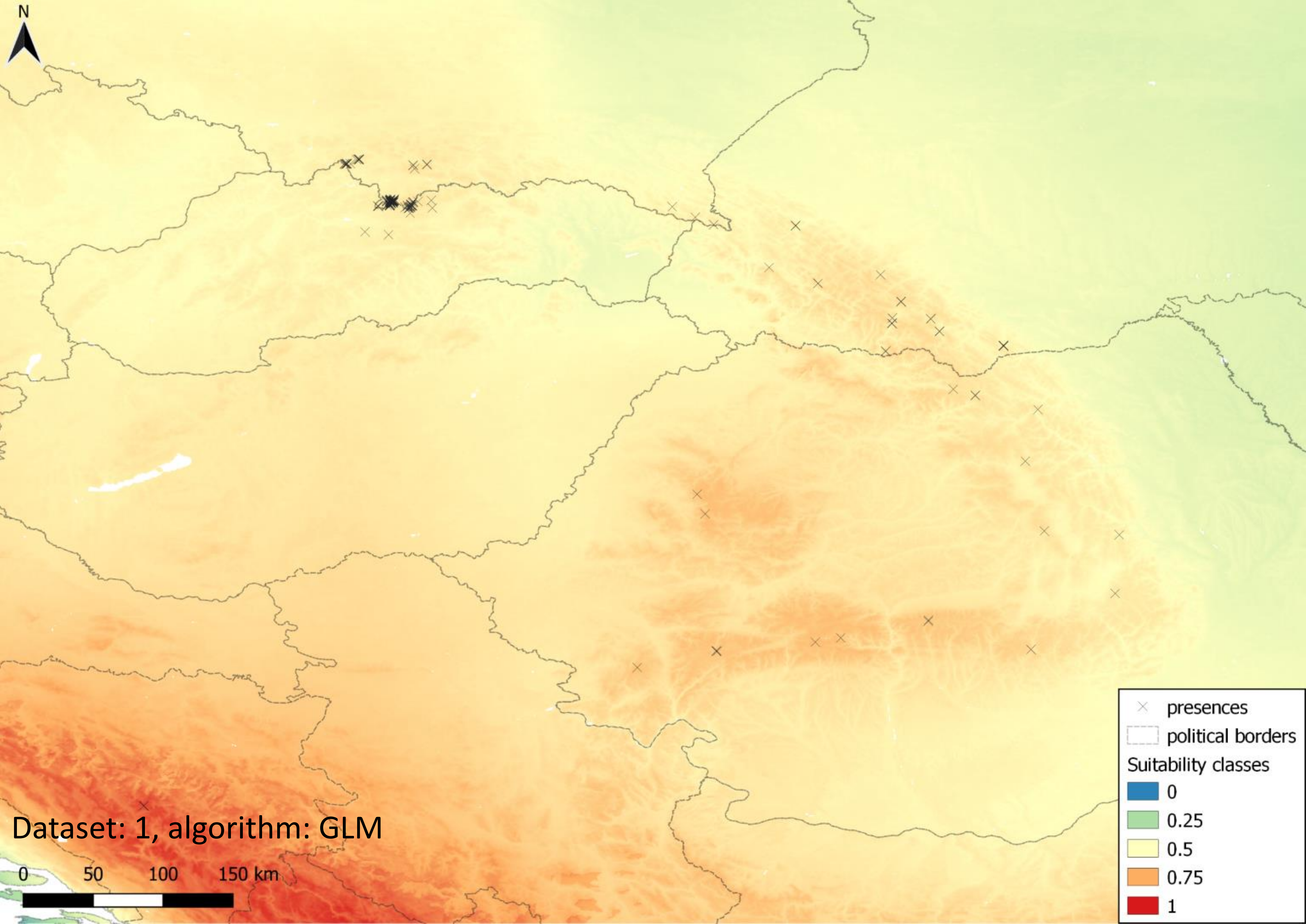
N
presences
political borders
Suitability classes
0
0.25
0.5
0.75
1
Dataset: 1, algorithm: GLM
0
50
100
150 km

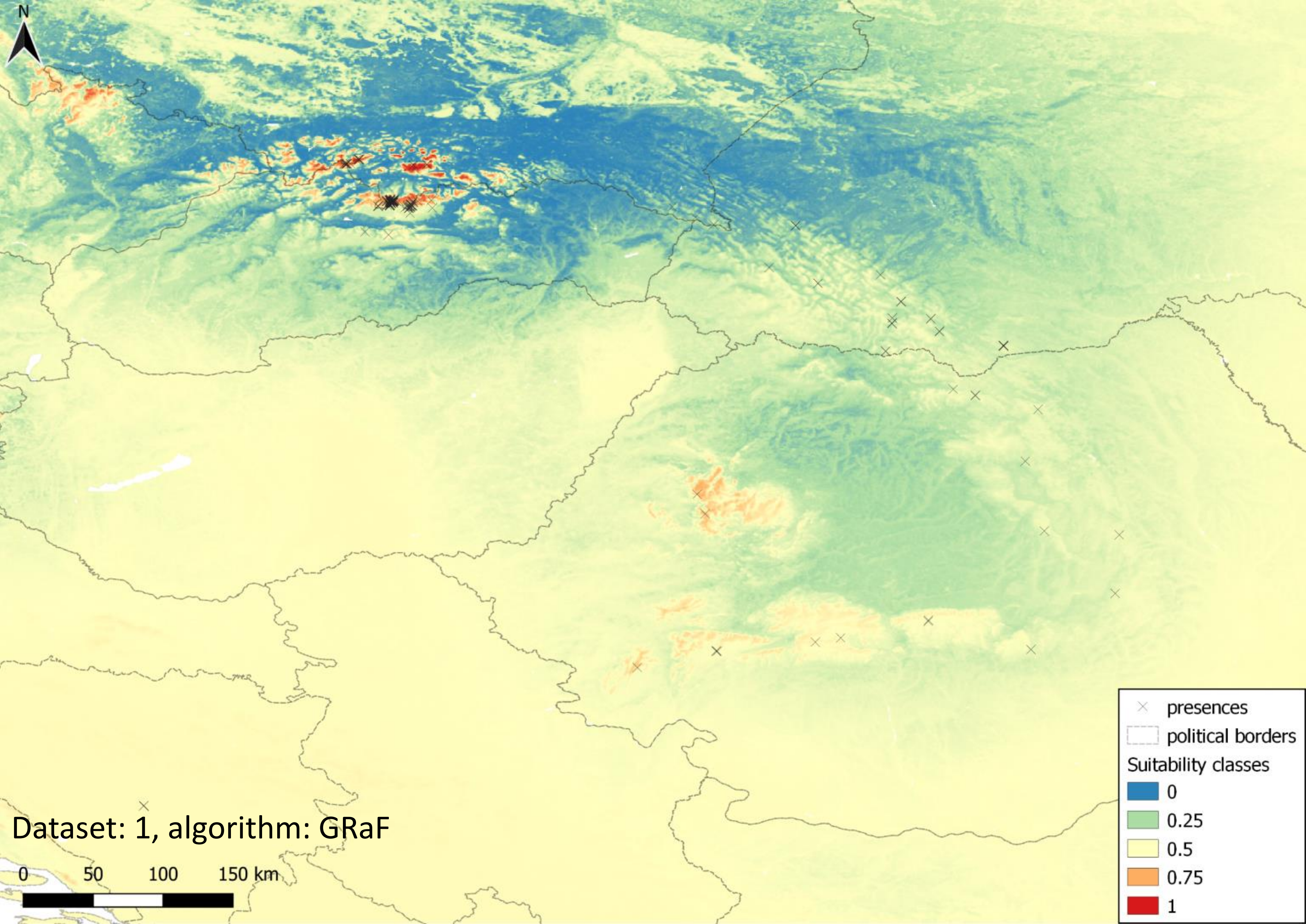
N
presences
political borders
Suitability classes
0
0.25
0.5
0.75
1
Dataset: 1, algorithm: GRaF
0
50
100
150 km

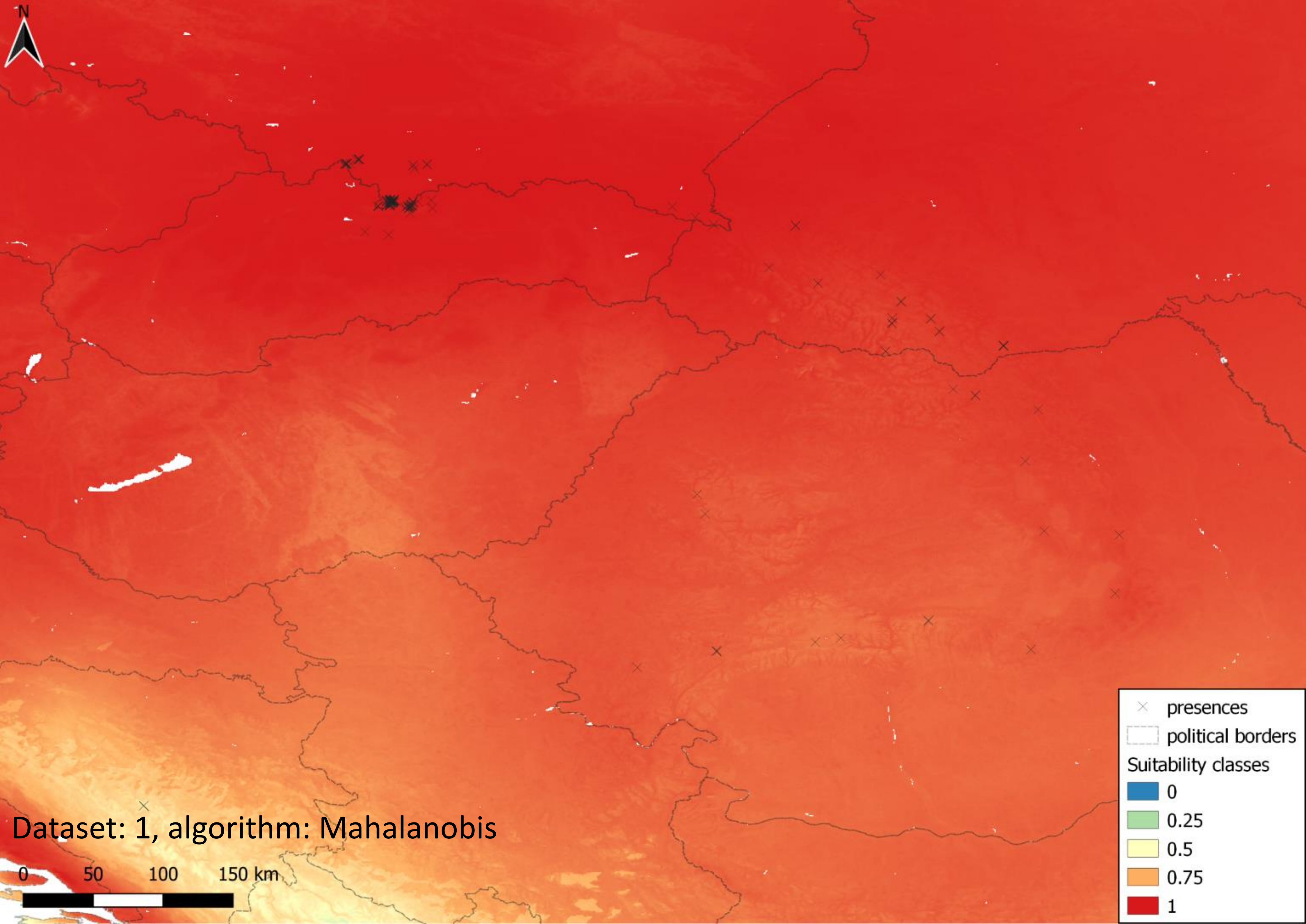
N
presences
political borders
Suitability classes
0
0.25
0.5
0.75
1
Dataset: 1, algorithm: Mahalanobis
0
50
100
150 km

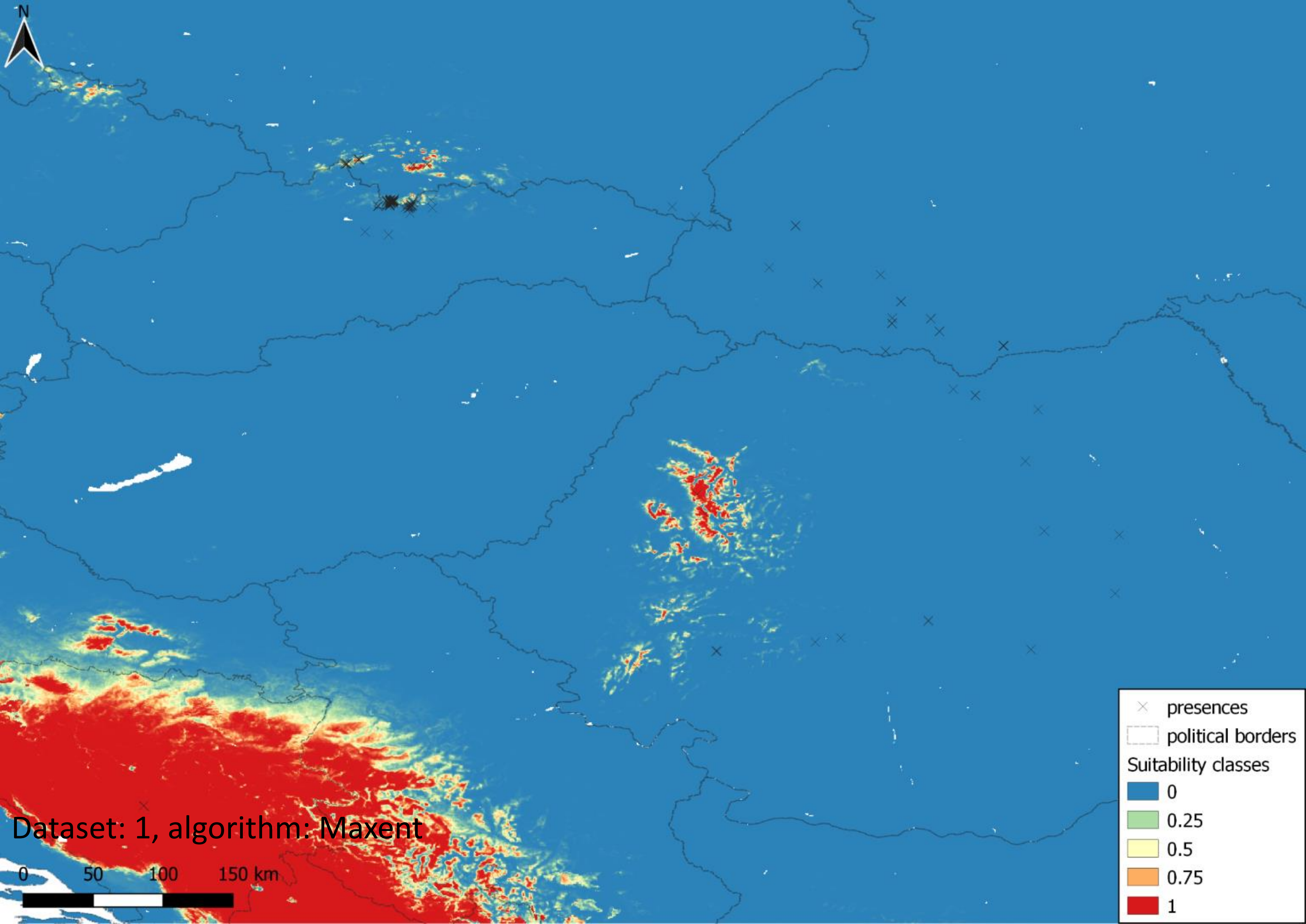
N
Dataset: 1, algorithm: Maxent
0
50
100
150 km
presences
political borders
Suitability classes
0
0.25
0.5
0.75
1

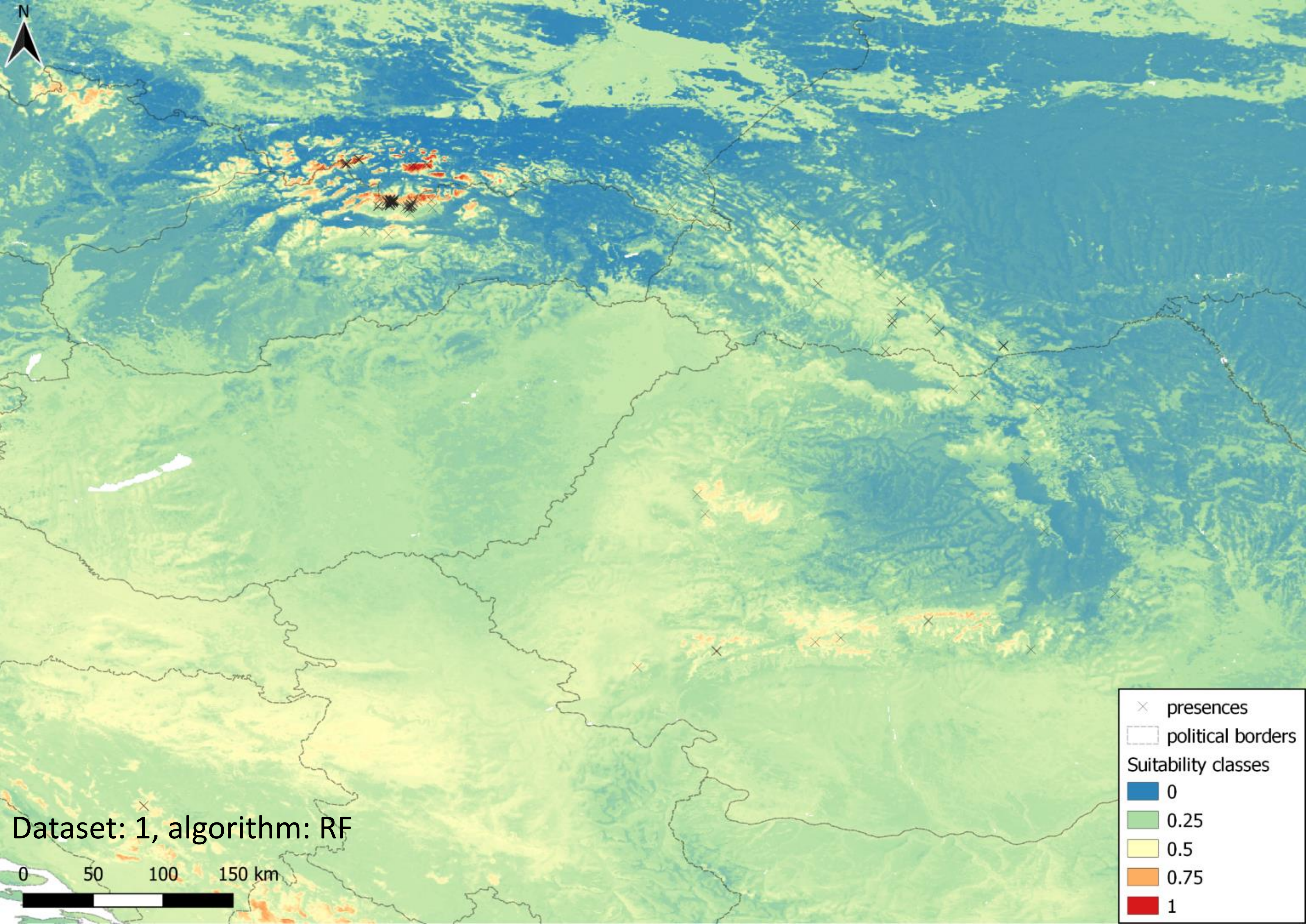
N
Dataset: 1, algorithm: RF
0
50
100
150 km
presences
political borders
Suitability classes
0
0.25
0.5
0.75
1

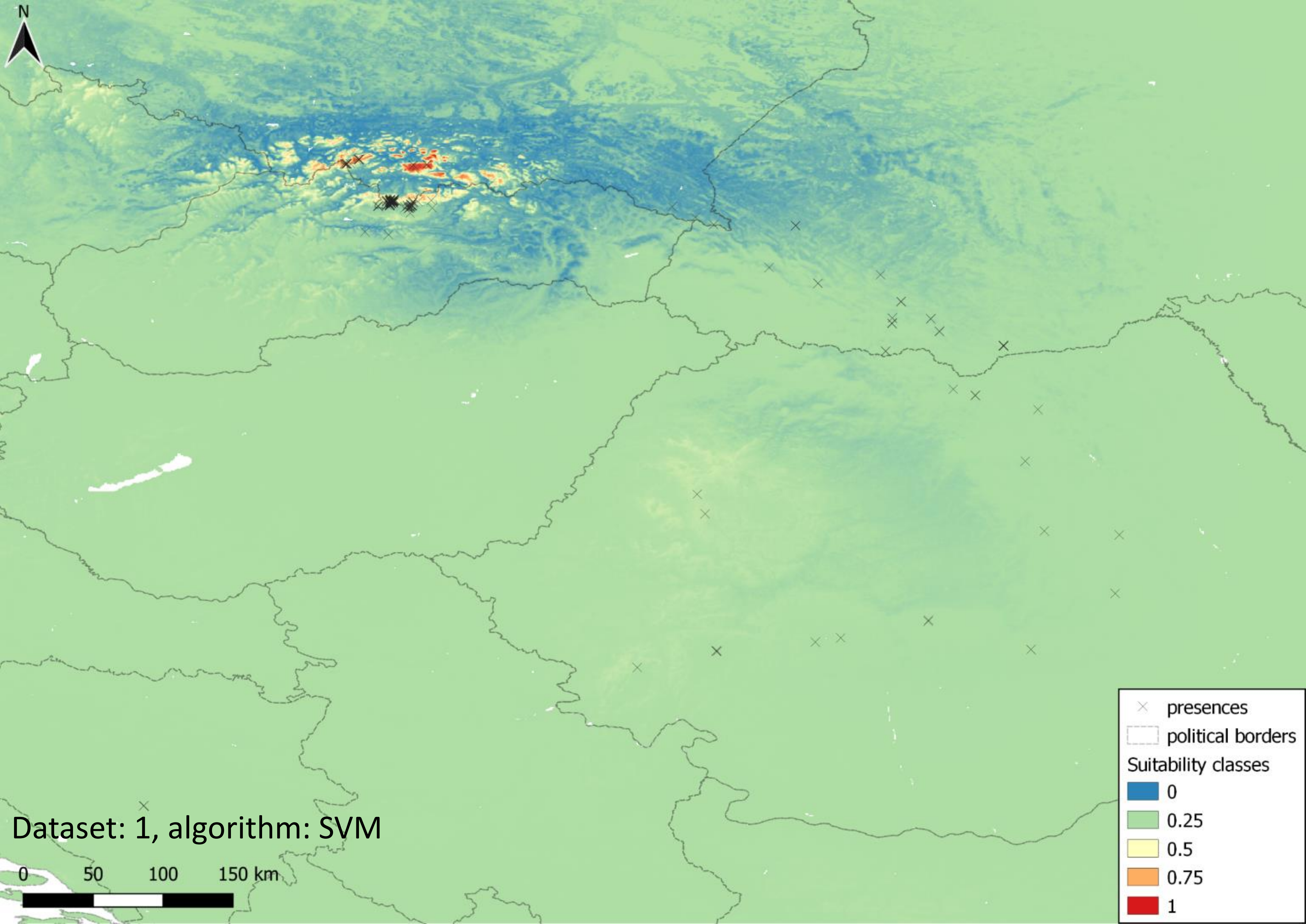
N
Dataset: 1, algorithm: SVM
0
50
100
150 km
presences
political borders
Suitability classes
0
0.25
0.5
0.75
1

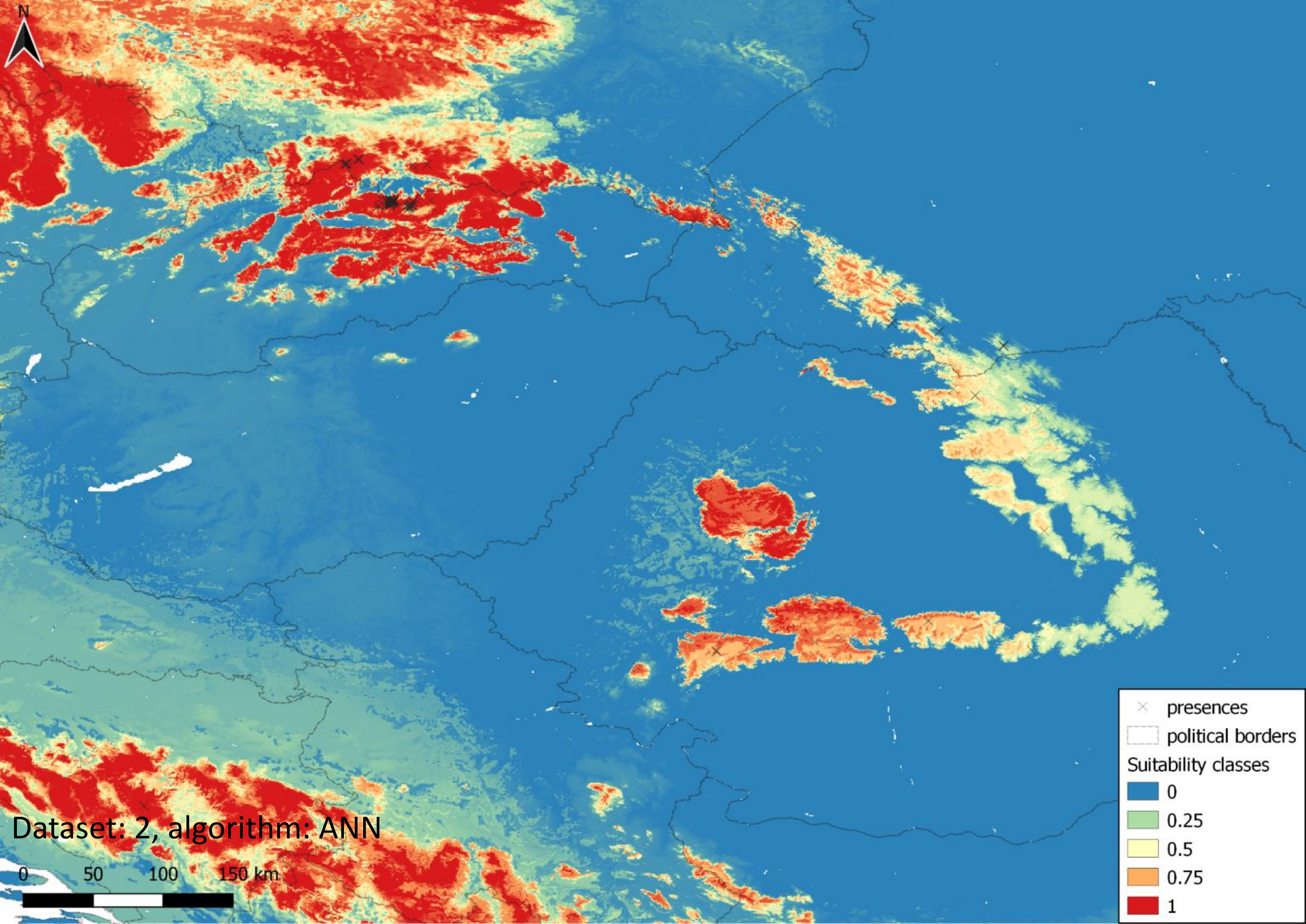
N
presences
political borders
Suitability classes
0
0.25
0.5
0.75
1
Dataset: 2, algorithm: ANN
0
50
100
150 km

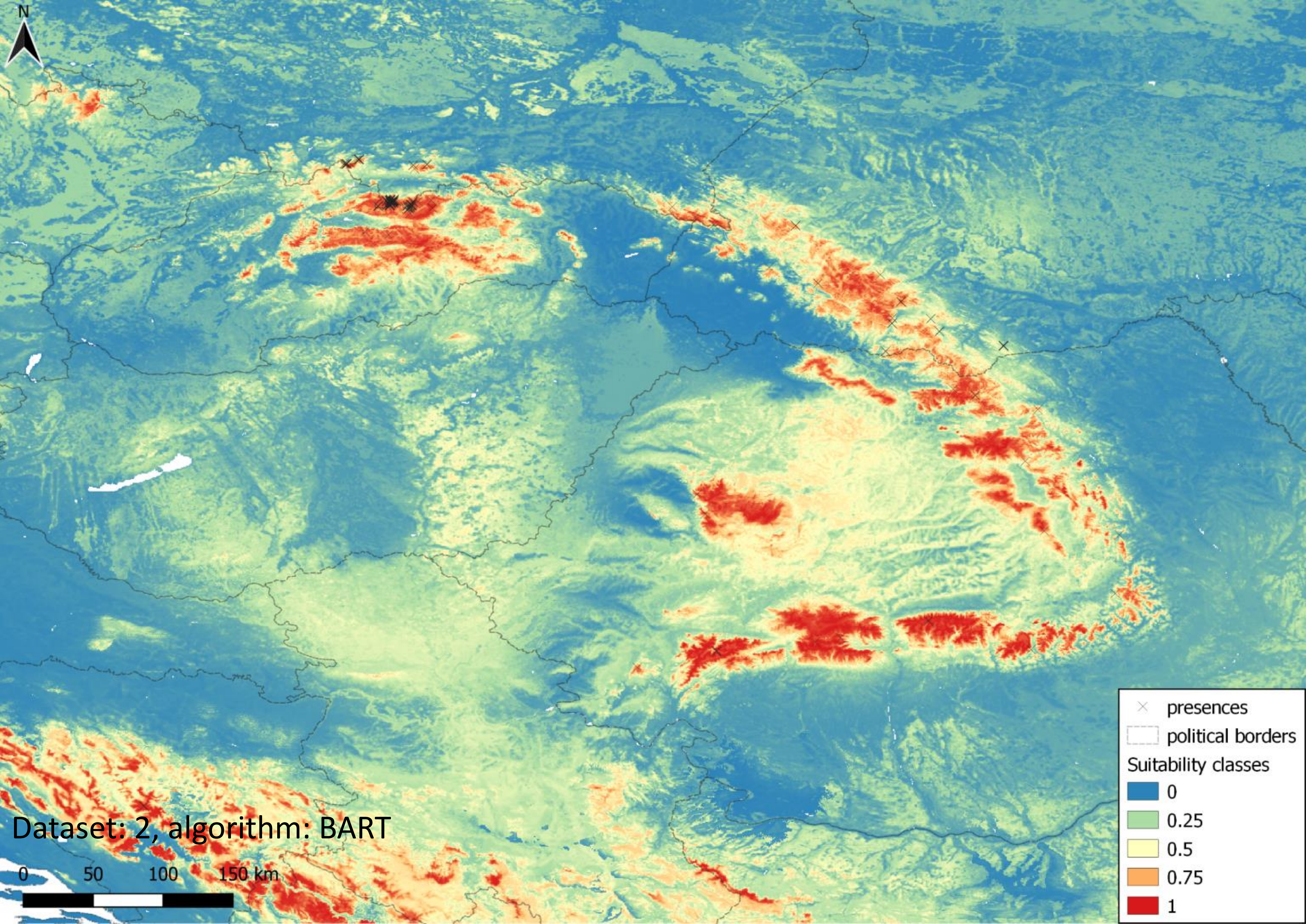

N
presences
political borders
Suitability classes
0
0.25
0.5
0.75
1
Dataset: 2, algorithm: BART
0
50
100
150 km

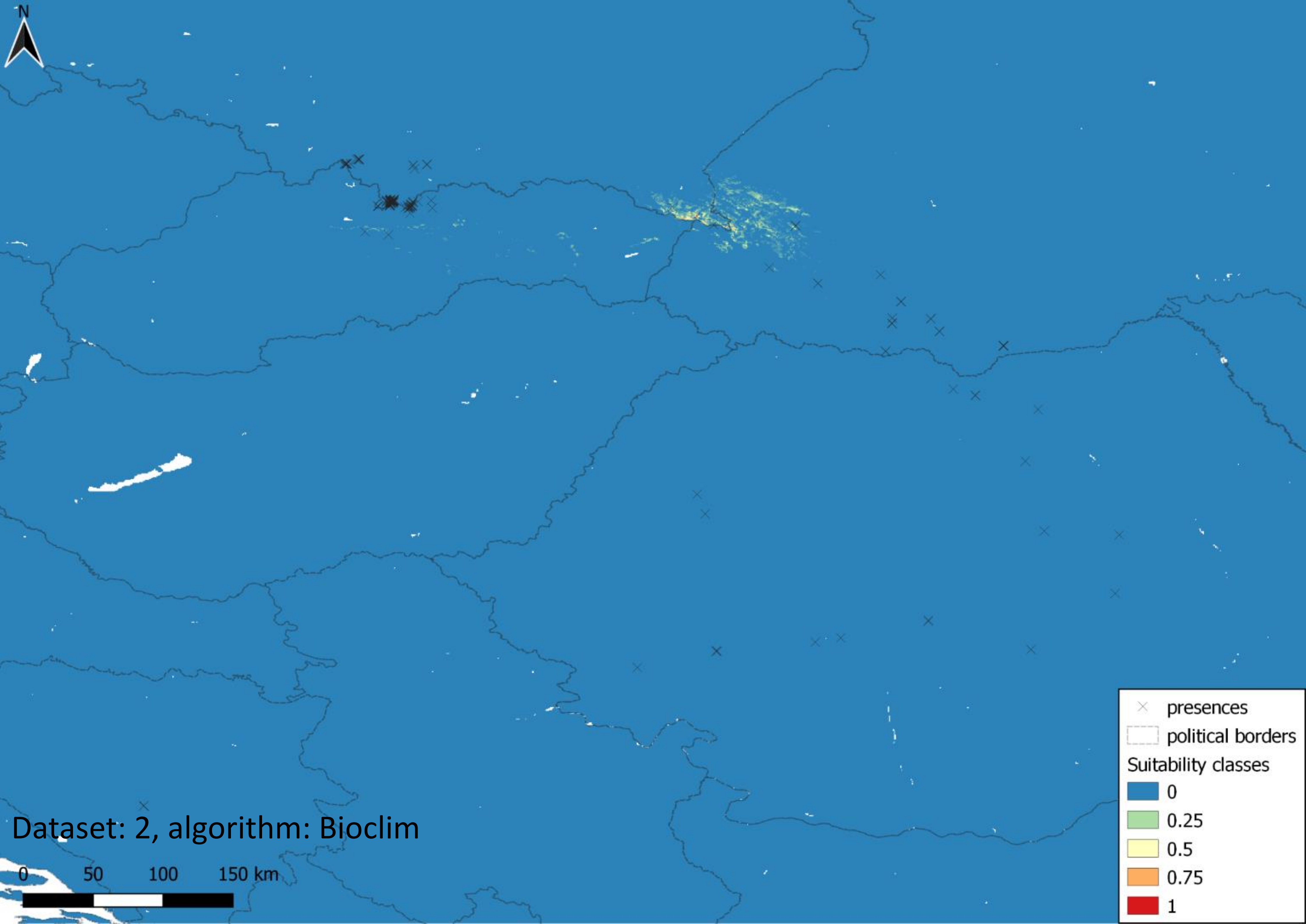
N
presences
political borders
Suitability classes
0
0.25
0.5
0.75
1
Dataset: 2, algorithm: Bioclim
0
50
100
150 km

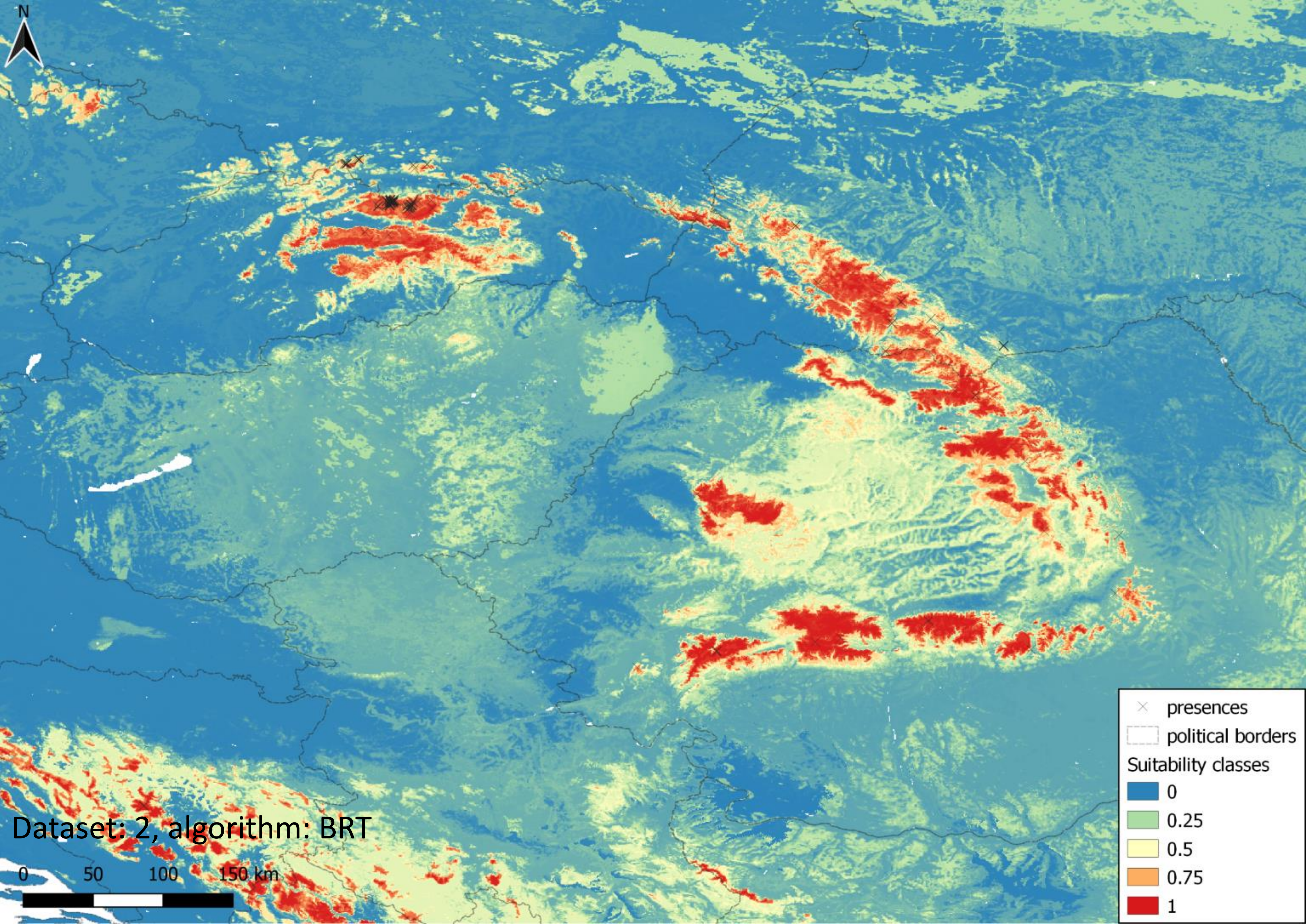
N
Dataset: 2, algorithm: BRT
0
50
100
150 km
presences
political borders
Suitability classes
0
0.25
0.5
0.75
1

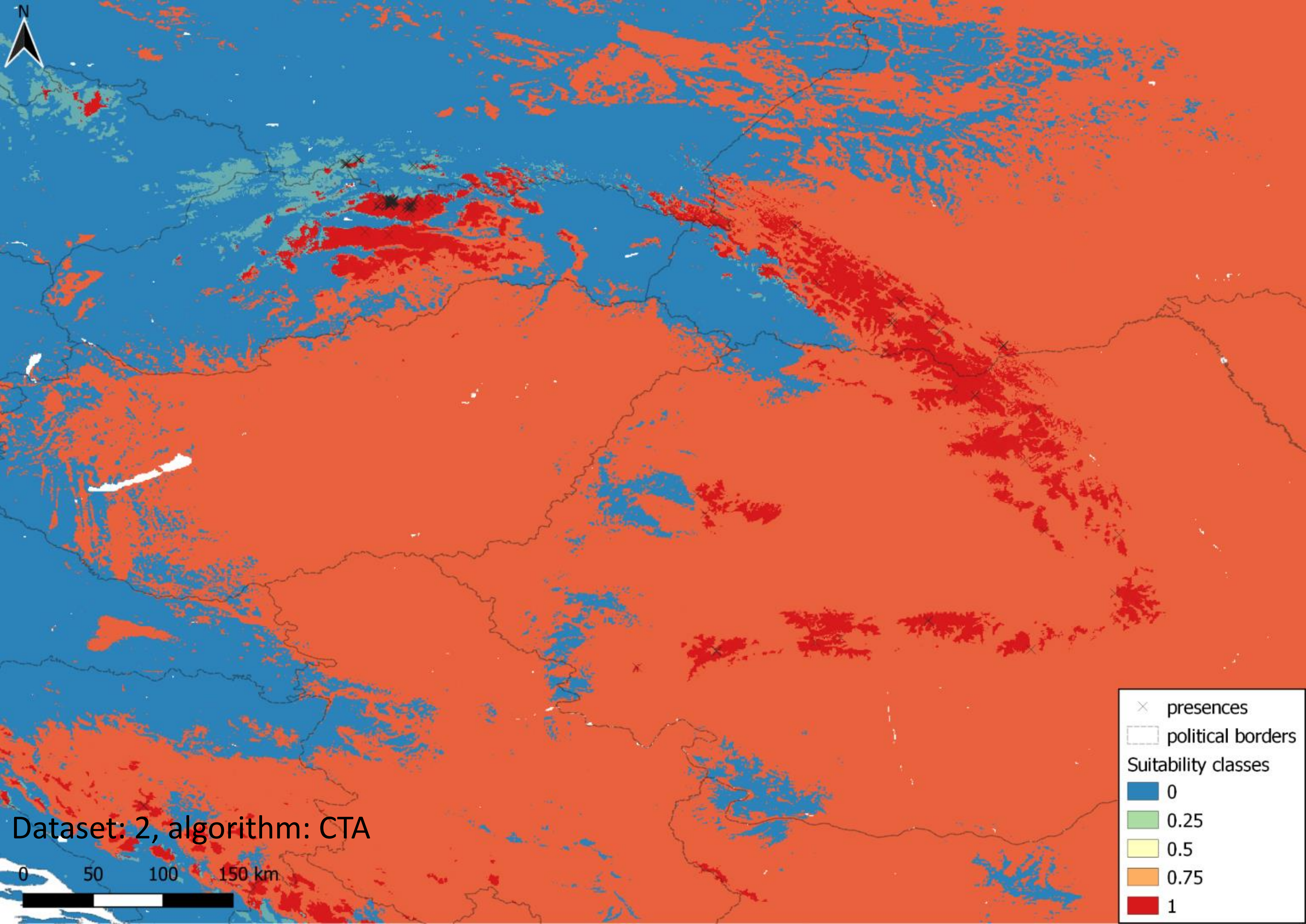
N
presences
political borders
Suitability classes
0
0.25
0.5
0.75
1
Dataset: 2, algorithm: CTA
0
50
100
150 km

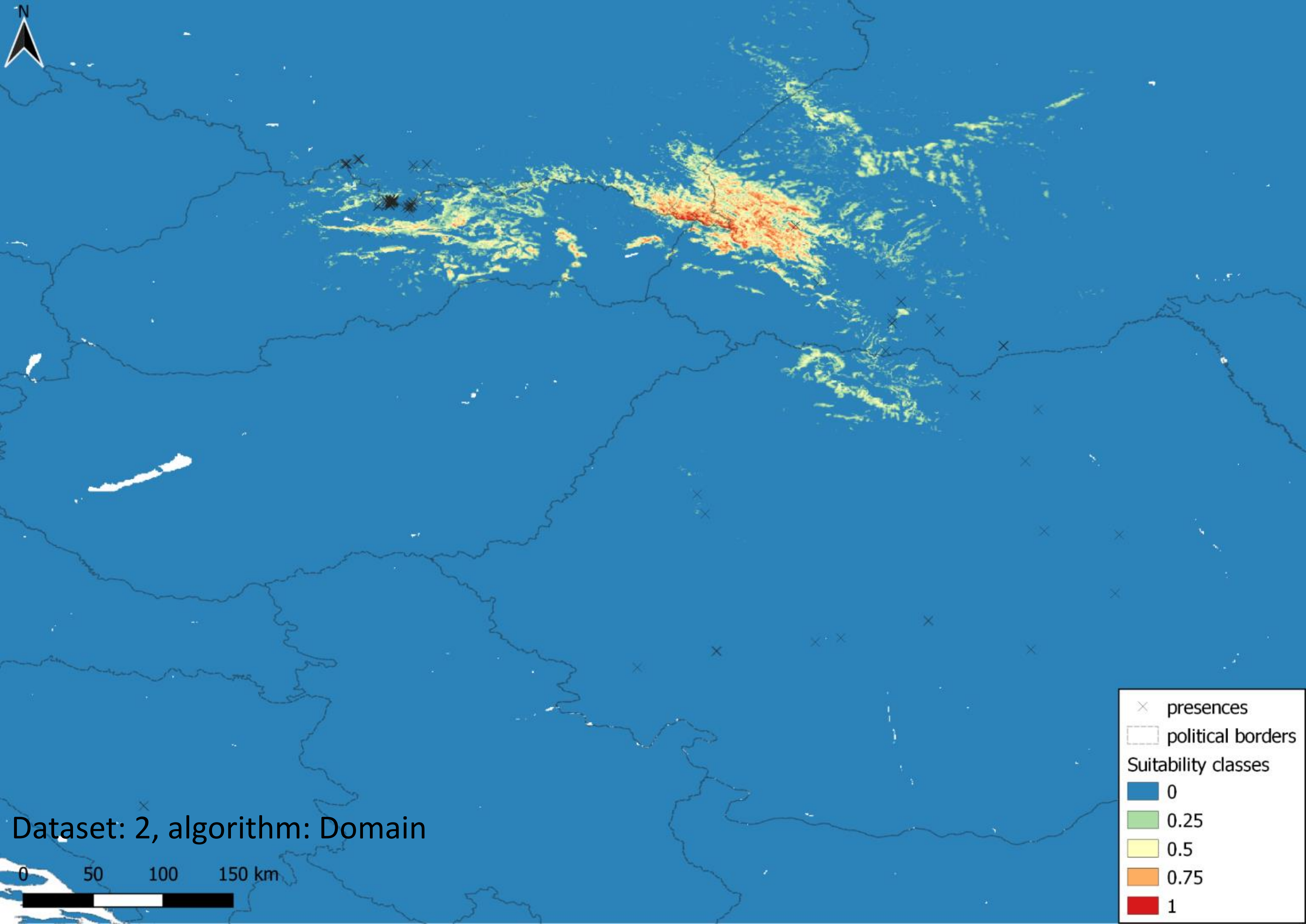
N
presences
political borders
Suitability classes
0
0.25
0.5
0.75
1
Dataset: 2, algorithm: Domain
0
50
100
150 km

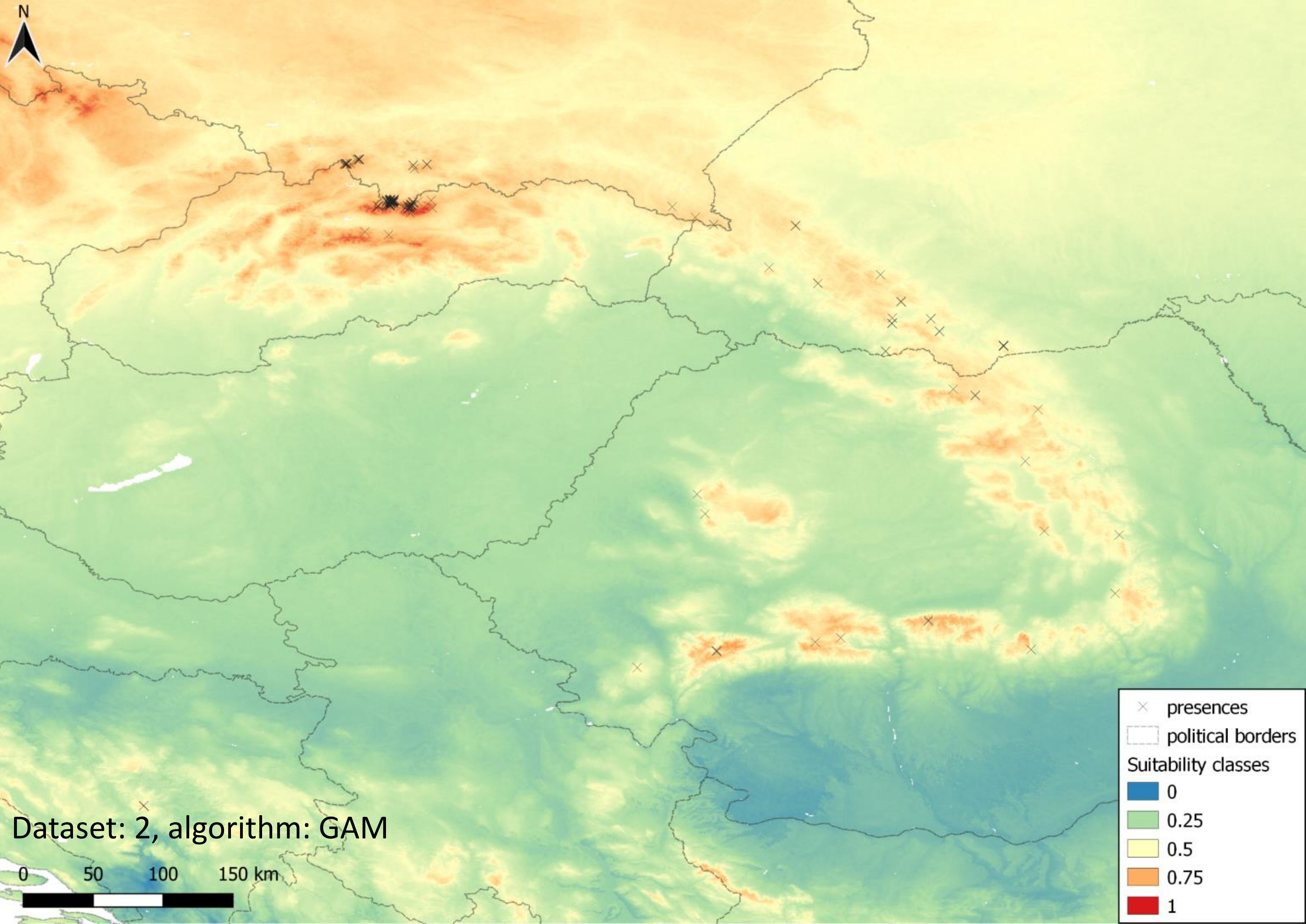
N
Dataset: 2, algorithm: GAM
0
50
100
150 km
presences
political borders
Suitability classes
0
0.25
0.5
0.75
1

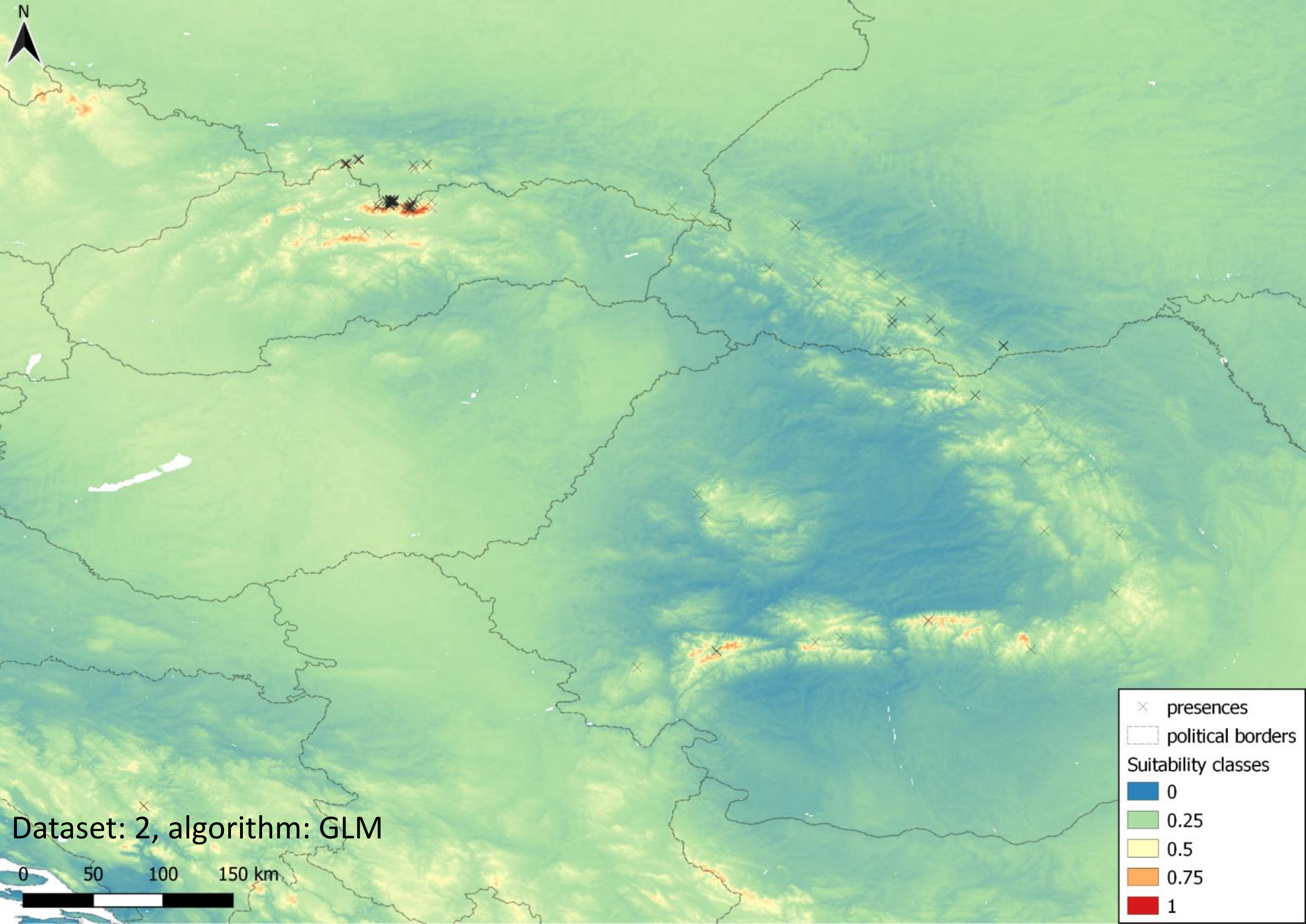
N
presences
political borders
Suitability classes
0
0.25
0.5
0.75
1
Dataset: 2, algorithm: GLM
0
50
100
150 km

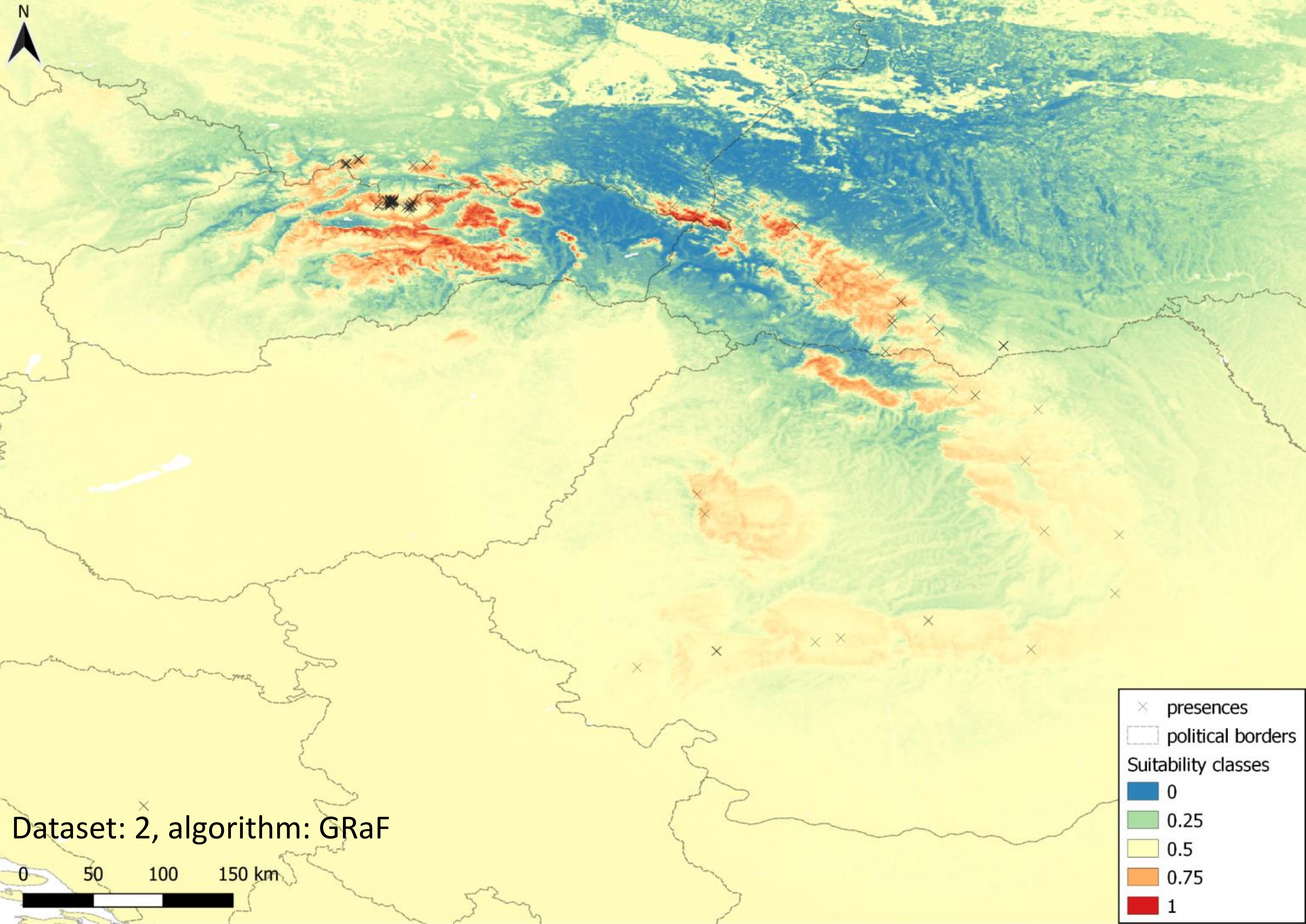
N
presences
political borders
Suitability classes
0
0.25
0.5
0.75
1
Dataset: 2, algorithm: GRaF
0
50
100
150 km

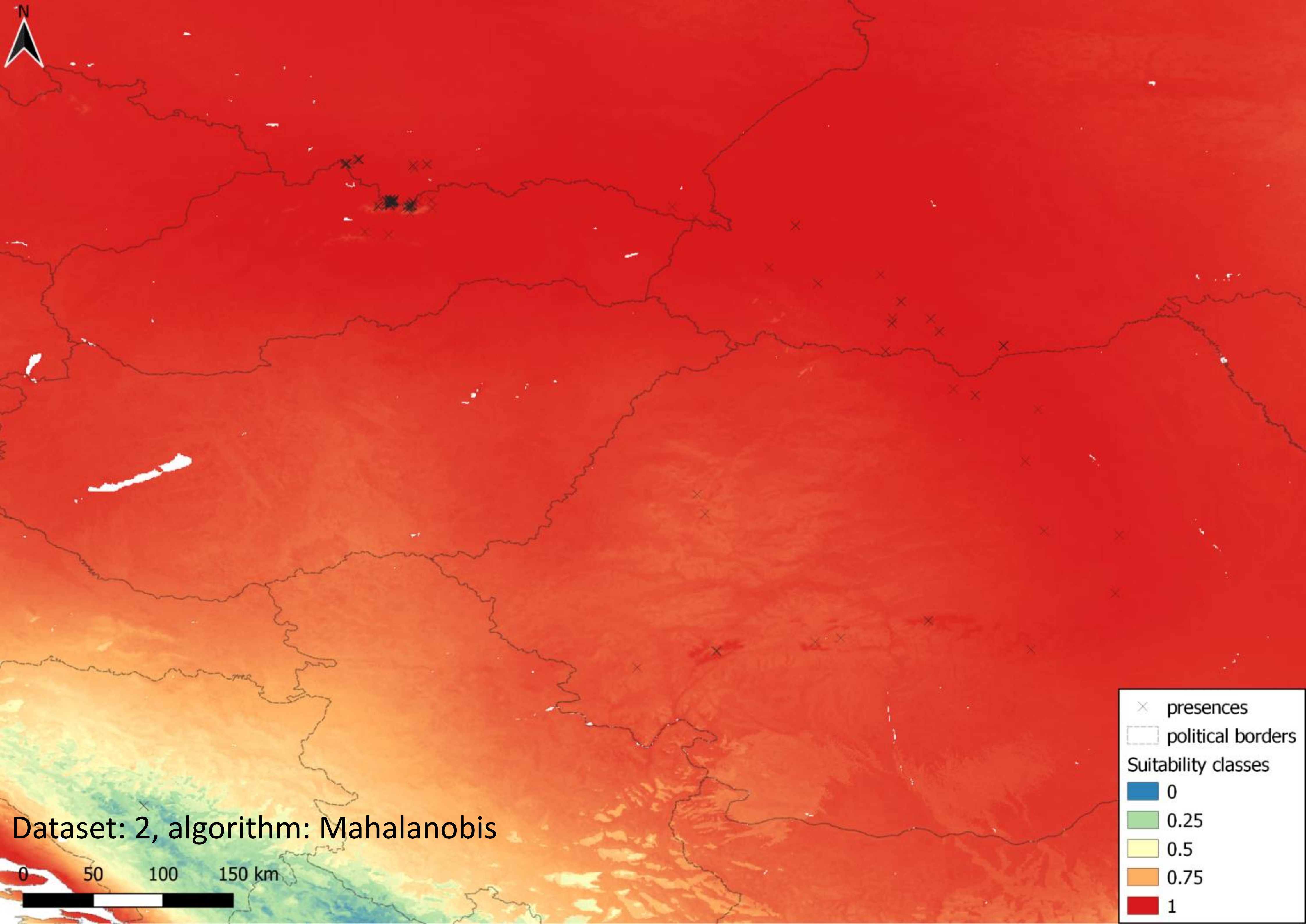
N
presences
political borders
Suitability classes
0
0.25
0.5
0.75
1
Dataset: 2, algorithm: Mahalanobis
0
50
100
150 km

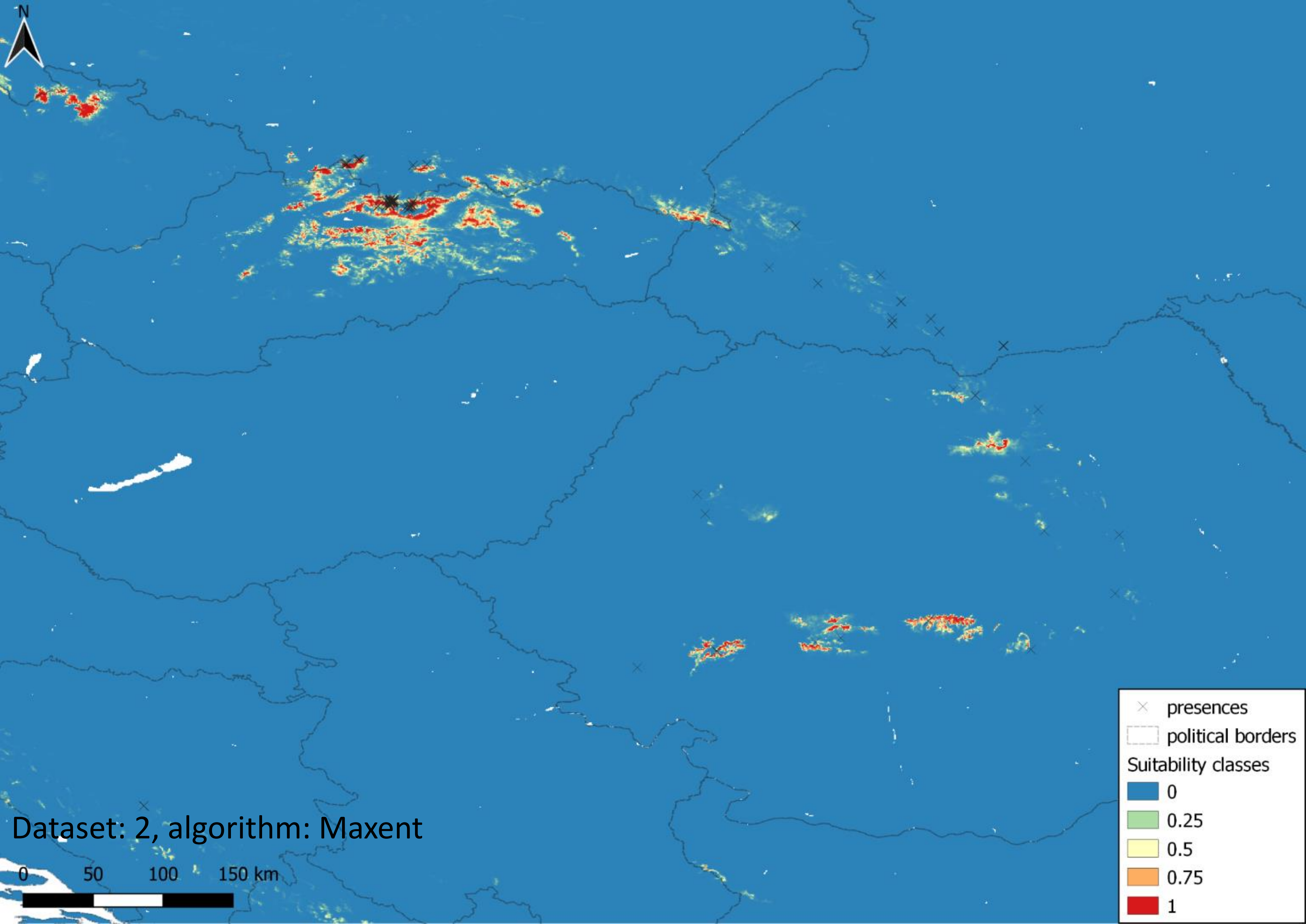
N
presences
political borders
Suitability classes
0
0.25
0.5
0.75
1
Dataset: 2, algorithm: Maxent
0
50
100
150 km

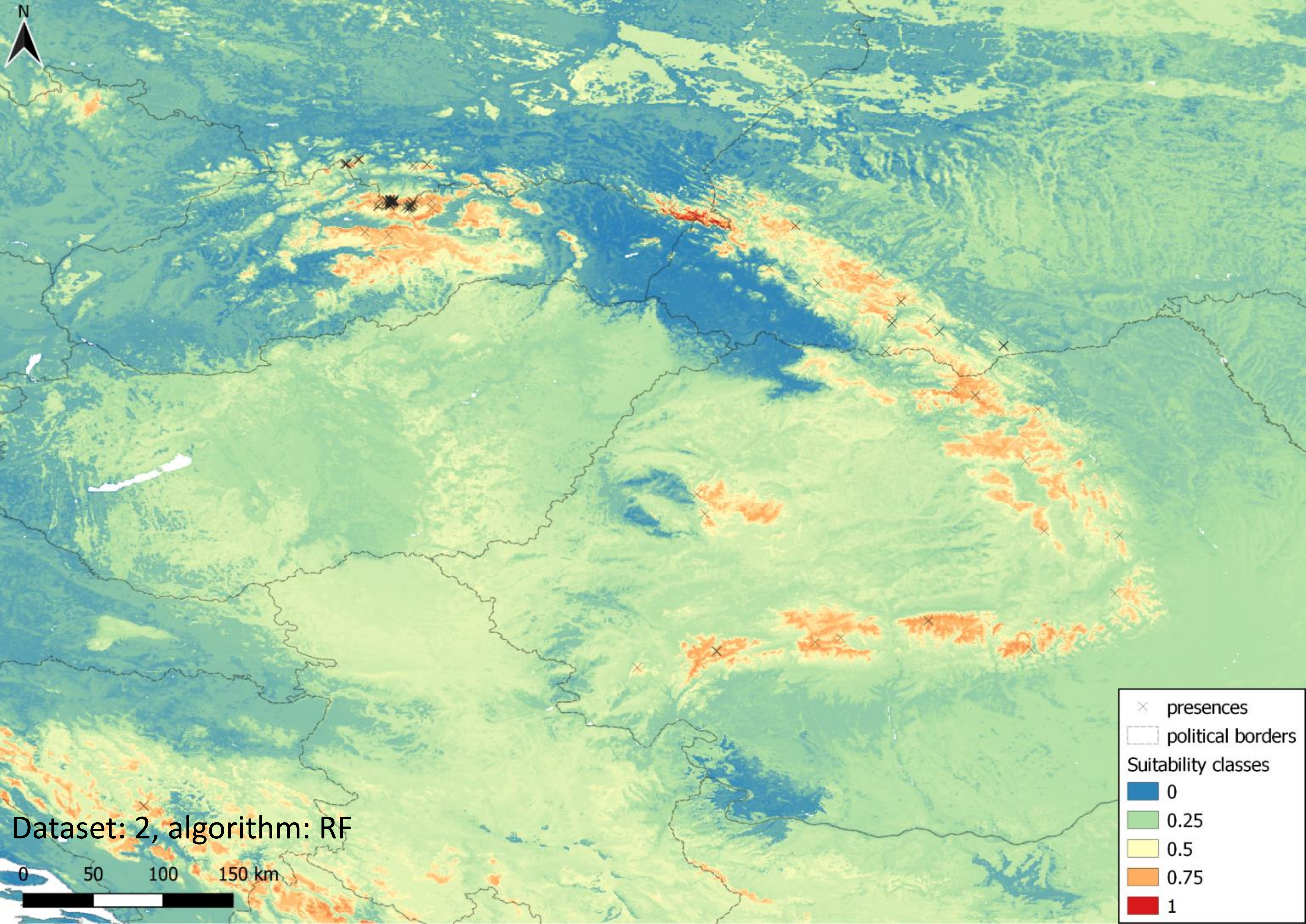
N
Dataset: 2, algorithm: RF
0 50 100 150 km
presences
political borders
Suitability classes
0
0.25
0.5
0.75
1

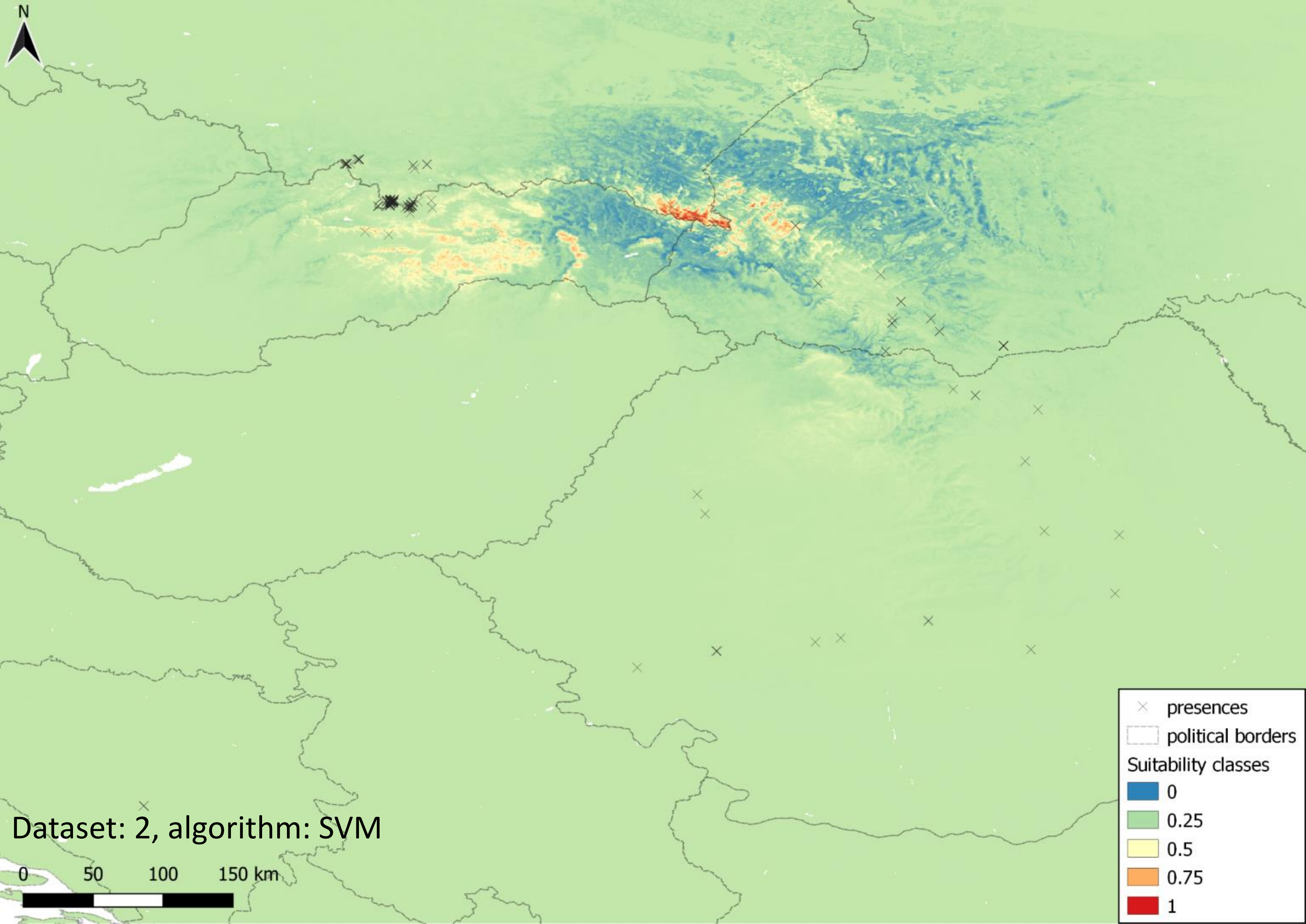
N
Dataset: 2, algorithm: SVM
0
50
100
150 km
presences
political borders
Suitability classes
0
0.25
0.5
0.75
1

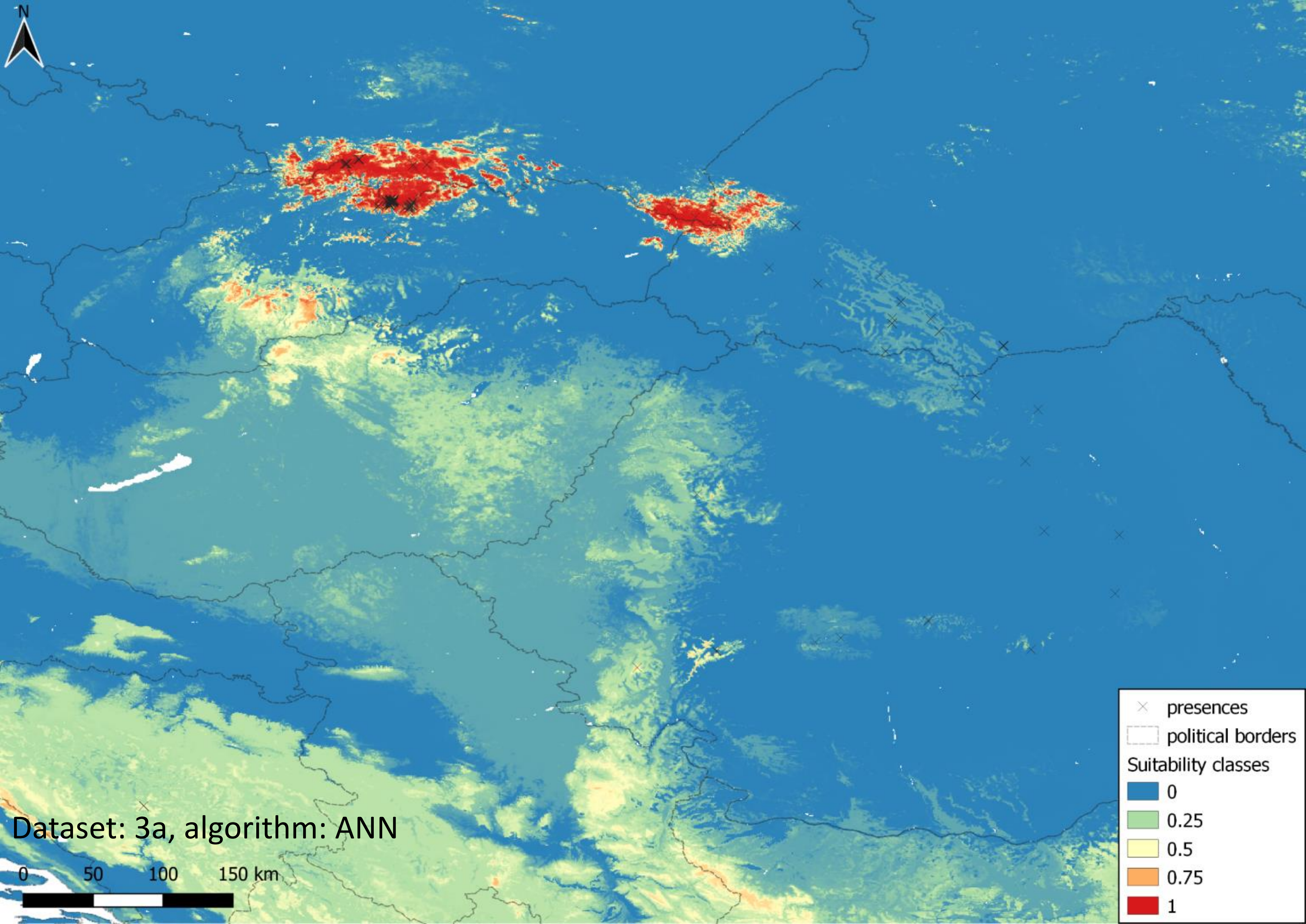
N
presences
political borders
Suitability classes
0
0.25
0.5
0.75
1
Dataset: 3a, algorithm: ANN
0
50
100
150 km

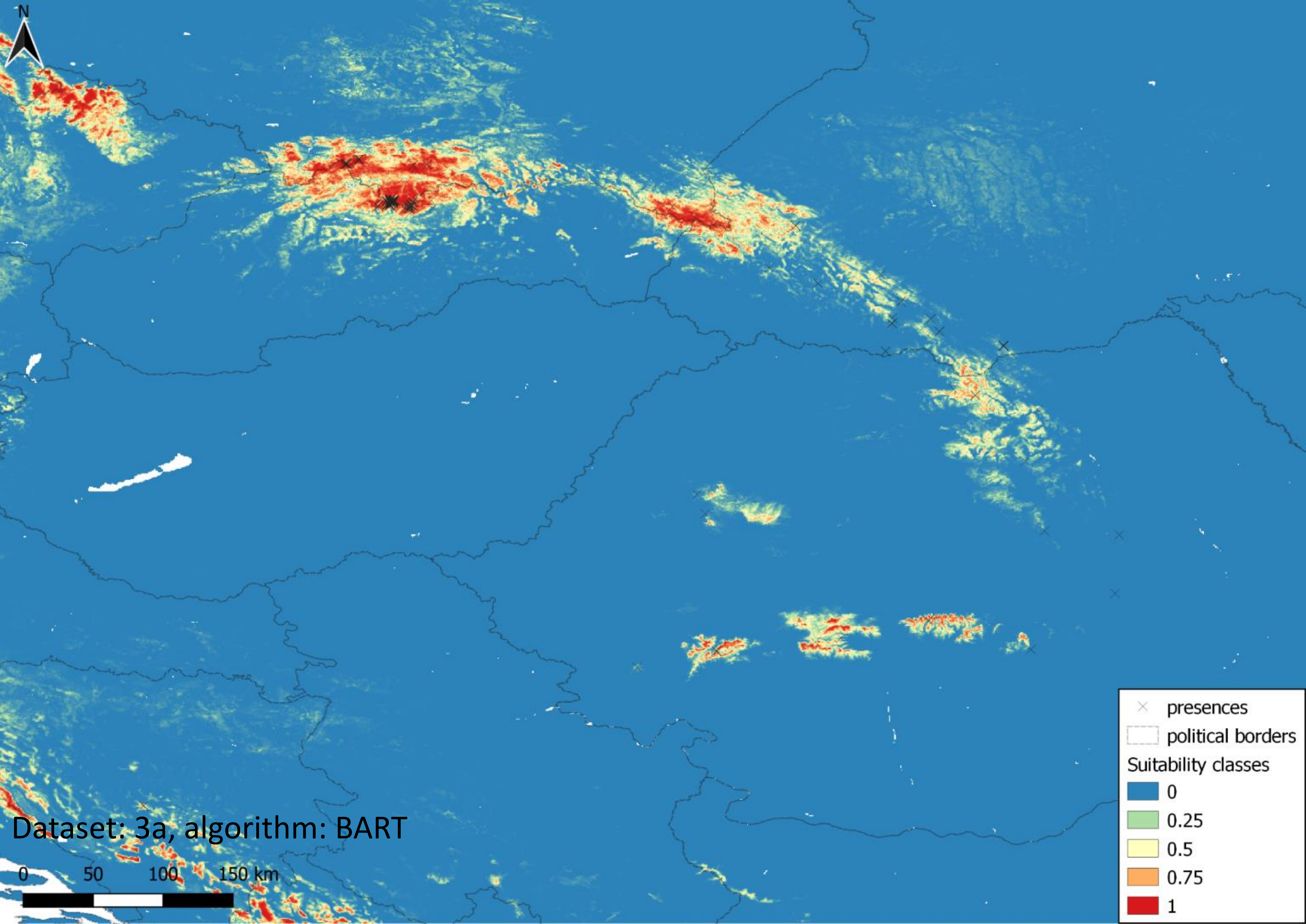
N
presences
political borders
Suitability classes
0
0.25
0.5
0.75
1
Dataset: 3a, algorithm: BART
0
50
100
150 km

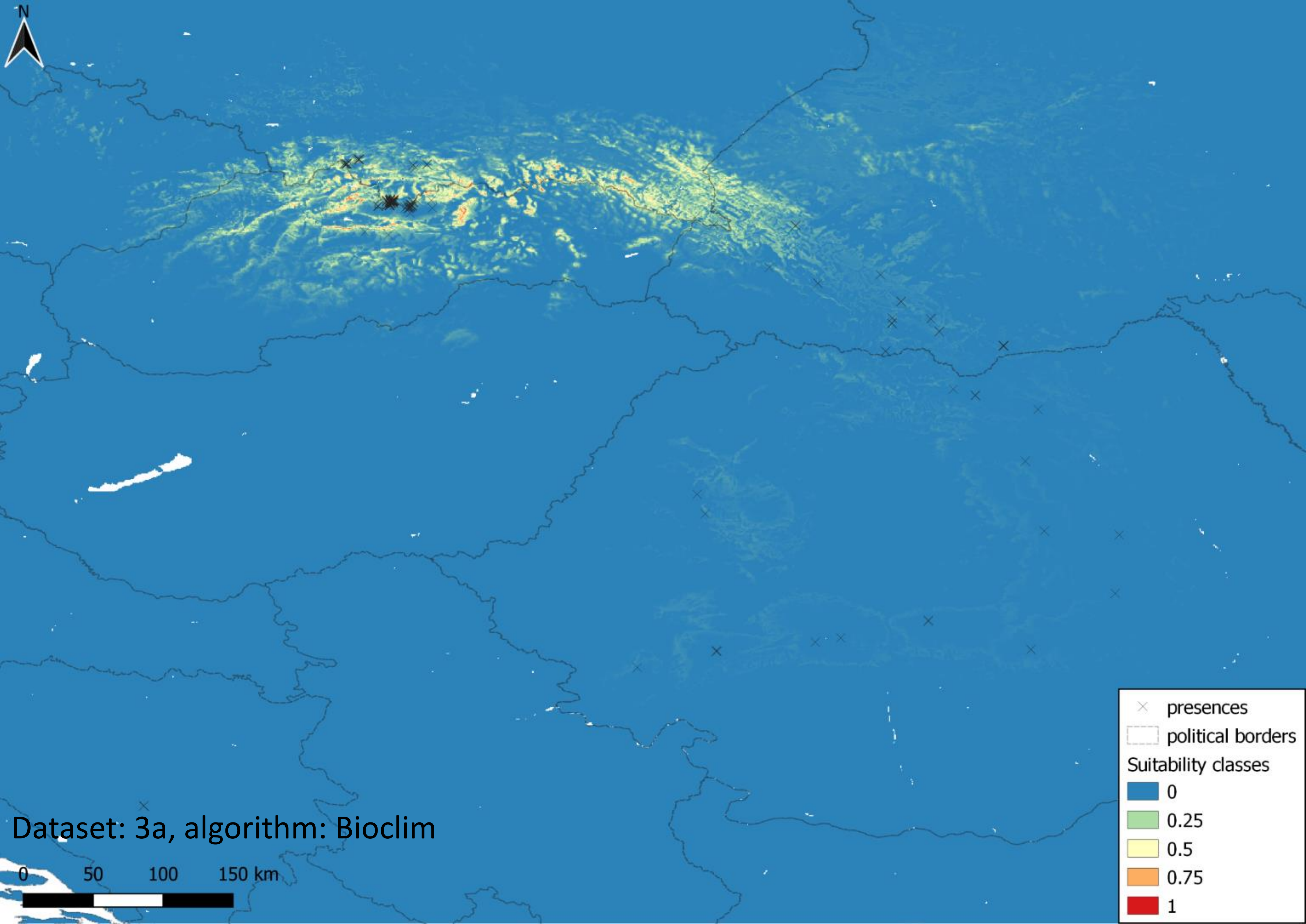
N
presences
political borders
Suitability classes
0
0.25
0.5
0.75
1
Dataset: 3a, algorithm: Bioclim
0
50
100
150 km

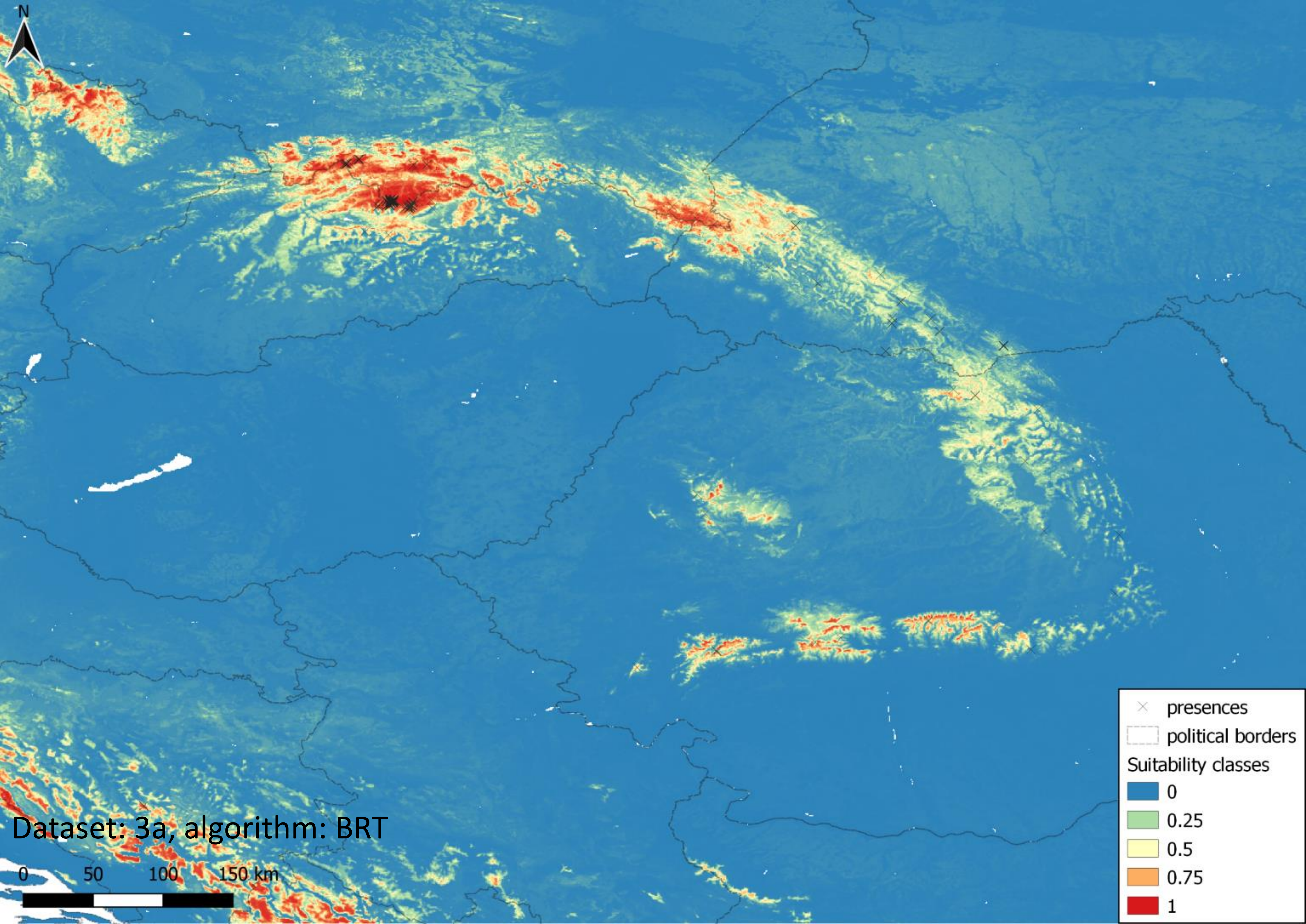
N
presences
political borders
Suitability classes
0
0.25
0.5
0.75
1
Dataset: 3a, algorithm: BRT
0
50
100
150 km

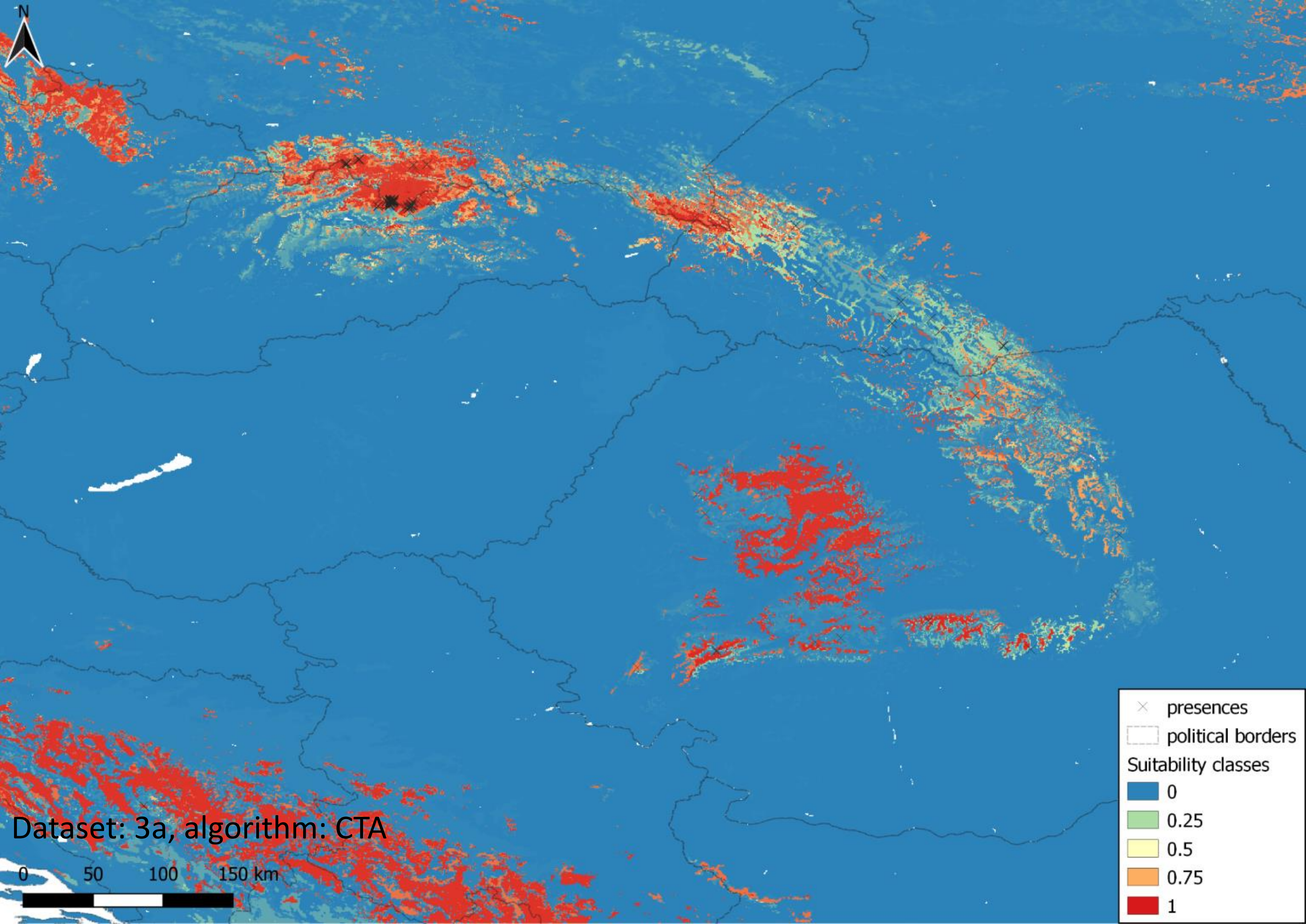
N
presences
political borders
Suitability classes
0
0.25
0.5
0.75
1
Dataset: 3a, algorithm: CTA
0
50
100
150 km

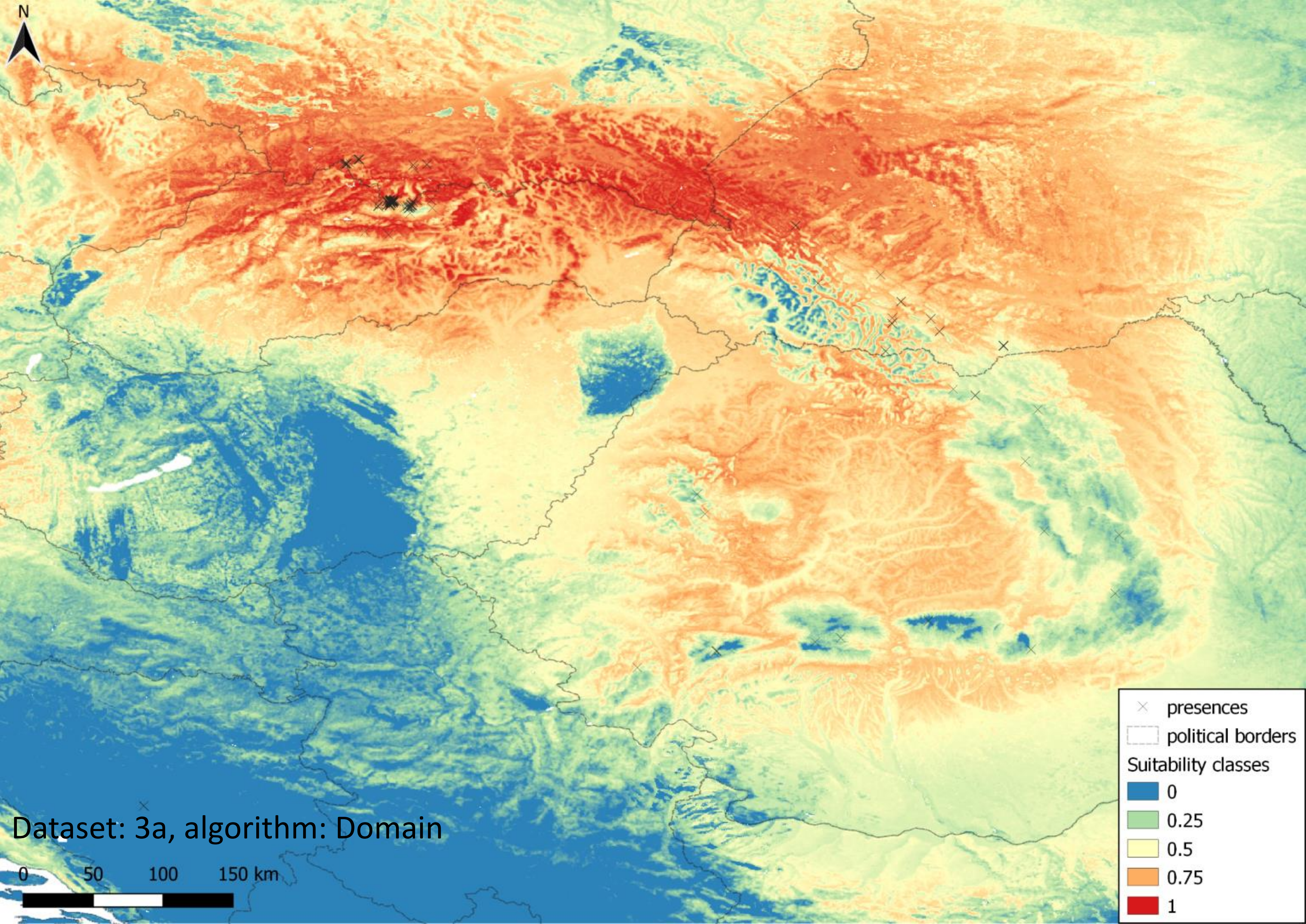
N
presences
political borders
Suitability classes
0
0.25
0.5
0.75
1
Dataset: 3a, algorithm: Domain
0
50
100
150 km

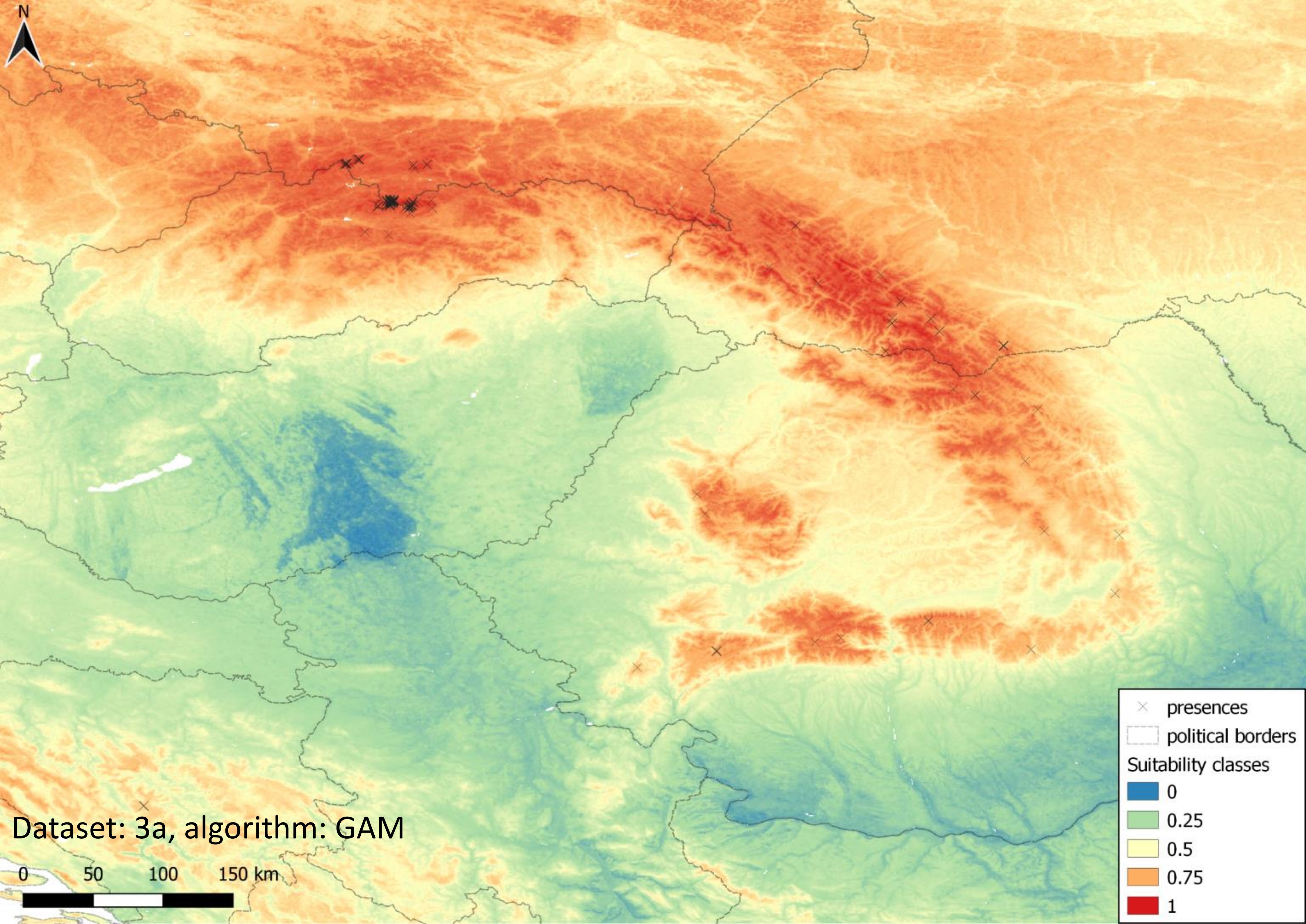
N
Dataset: 3a, algorithm: GAM
0
50
100
150 km
presences
political borders
Suitability classes
0
0.25
0.5
0.75
1

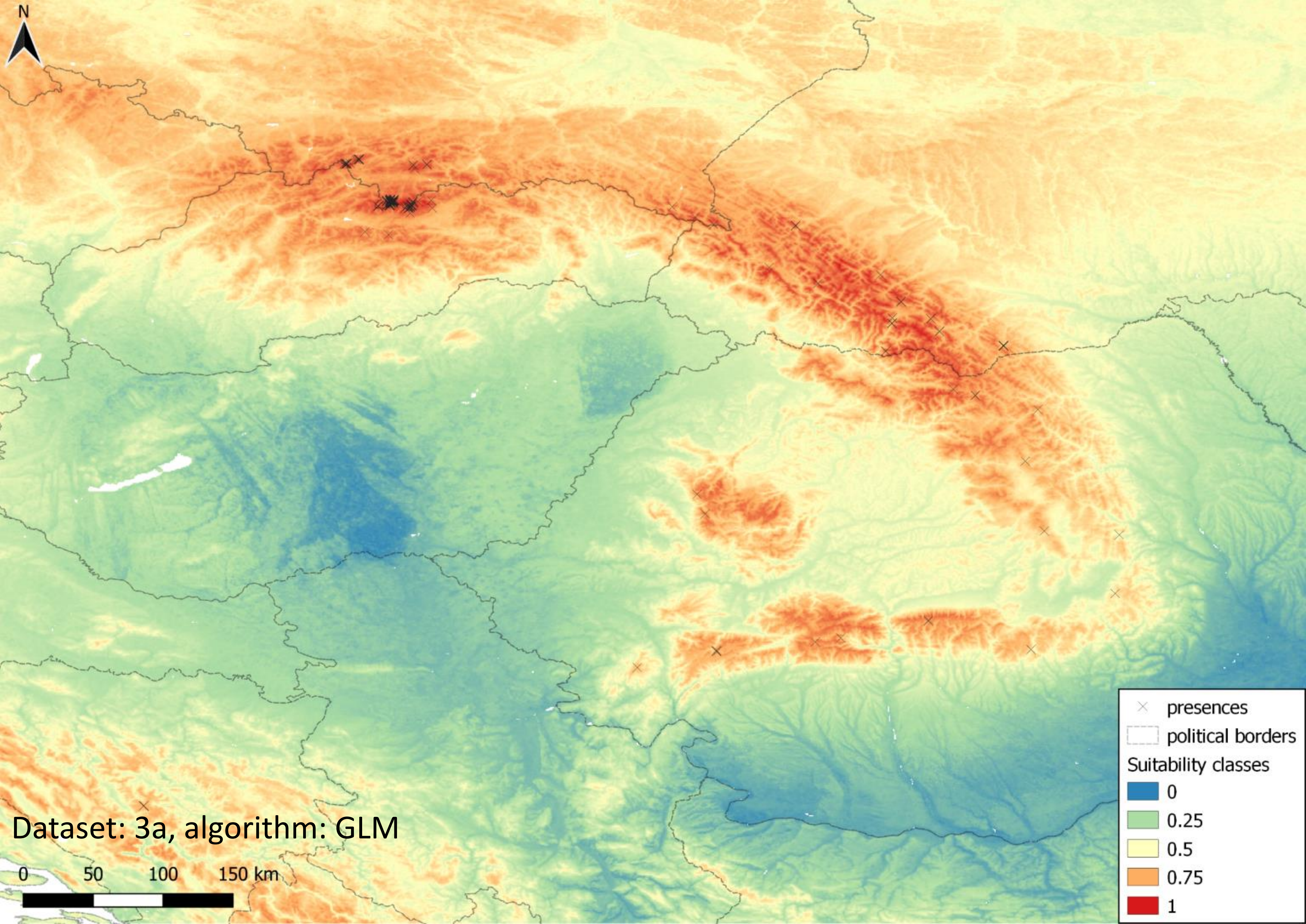
N
presences
political borders
Suitability classes
0
0.25
0.5
0.75
1
Dataset: 3a, algorithm: GLM
0
50
100
150 km

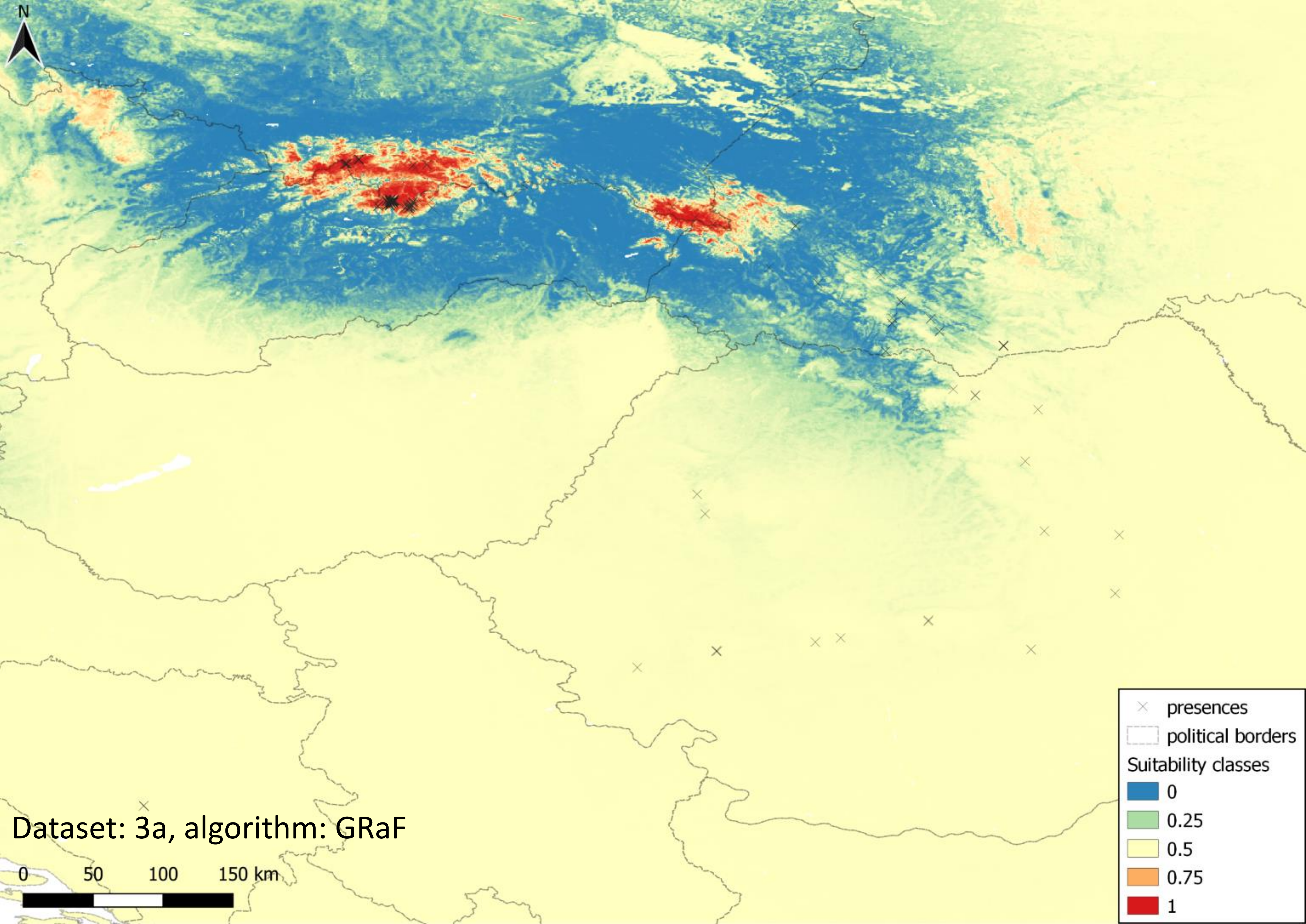
N
presences
political borders
Suitability classes
0
0.25
0.5
0.75
1
Dataset: 3a, algorithm: GRaF
0
50
100
150 km

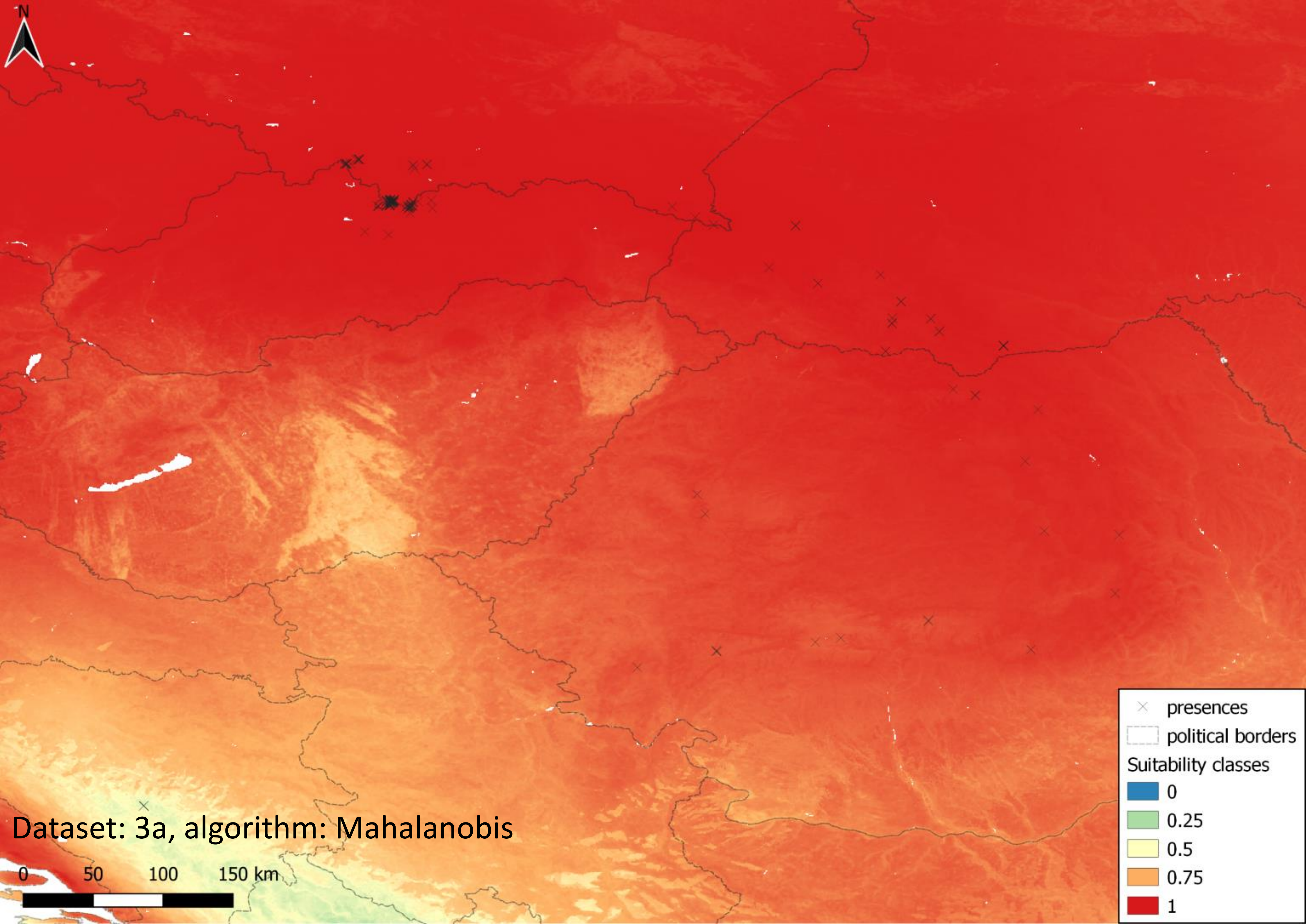
N
Dataset: 3a, algorithm: Mahalanobis
0
50
100
150 km
presences
political borders
Suitability classes
0
0.25
0.5
0.75
1

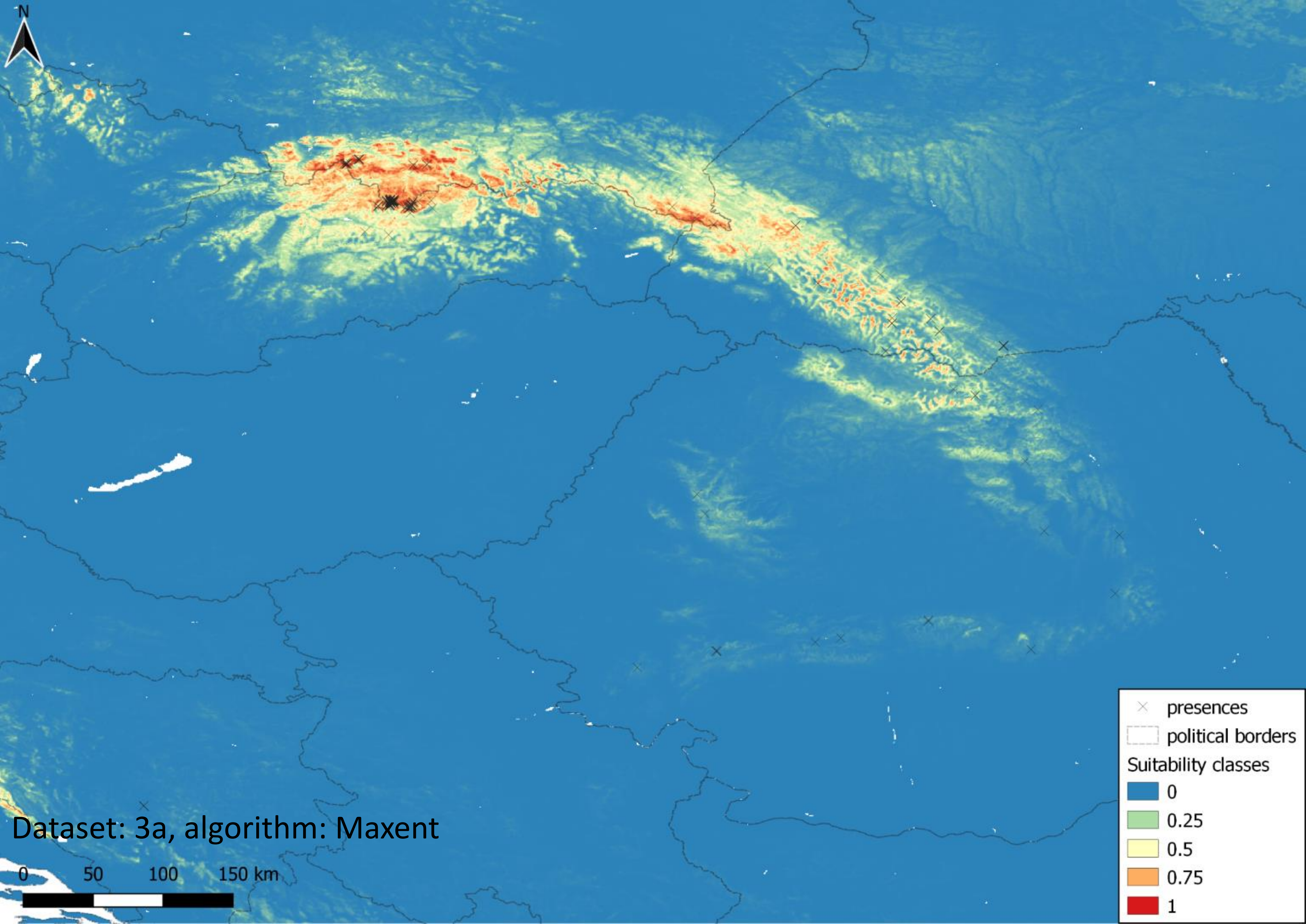
N
presences
political borders
Suitability classes
0
0.25
0.5
0.75
1
Dataset: 3a, algorithm: Maxent
0
50
100
150 km

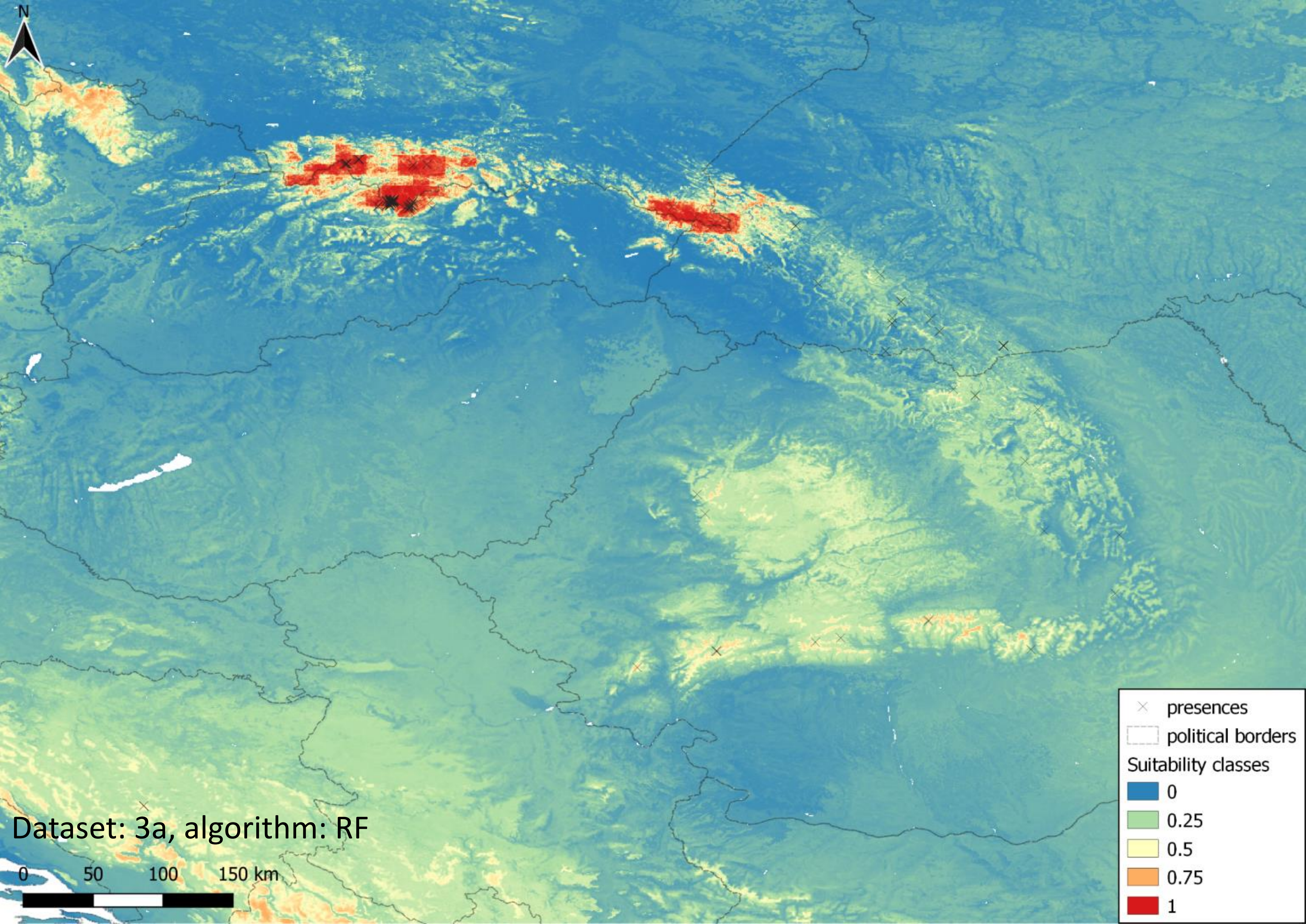
N
presences
political borders
Suitability classes
0
0.25
0.5
0.75
1
Dataset: 3a, algorithm: RF
0
50
100
150 km

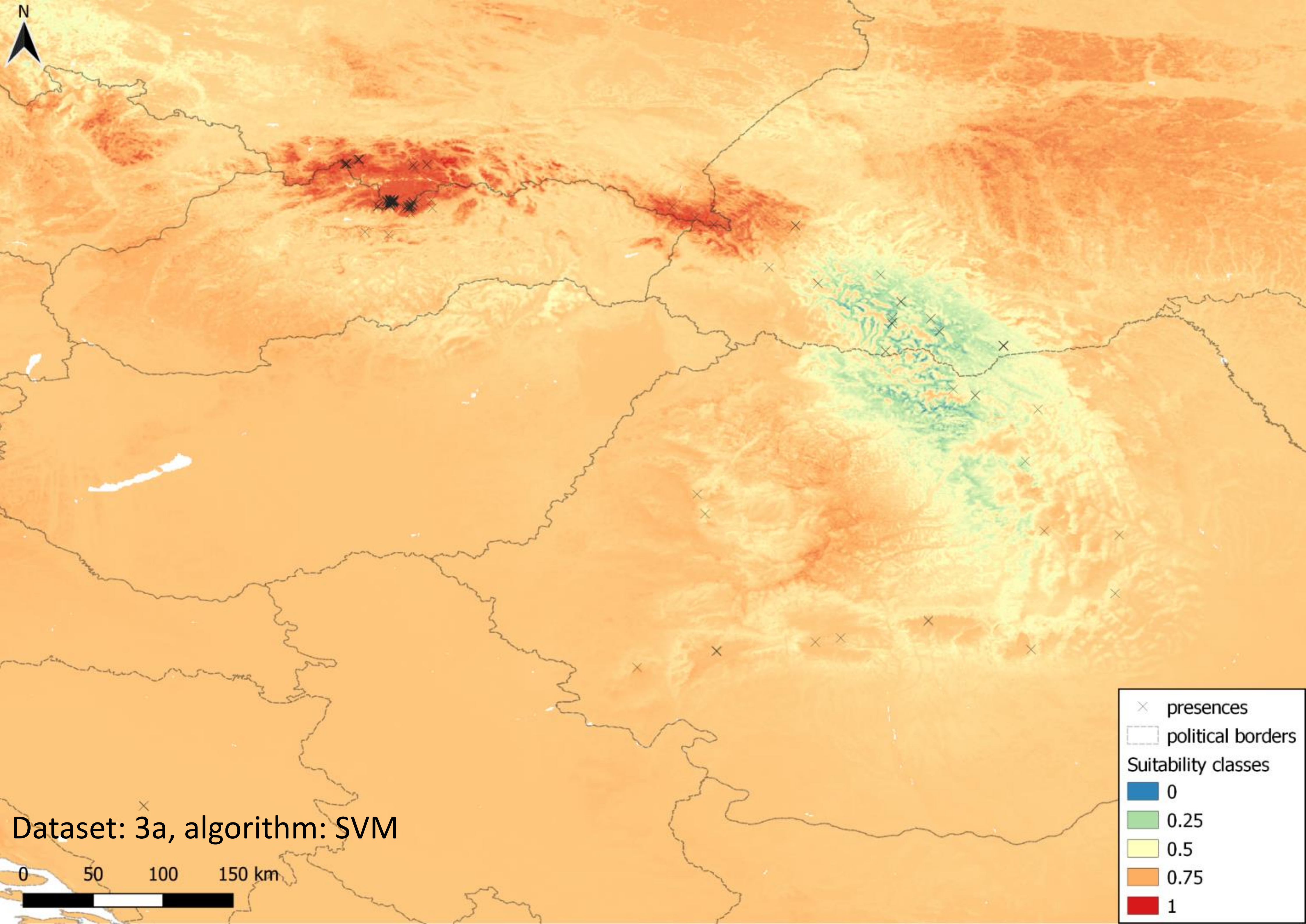
N
Dataset: 3a, algorithm: SVM
0
50
100
150 km
presences
political borders
Suitability classes
0
0.25
0.5
0.75
1

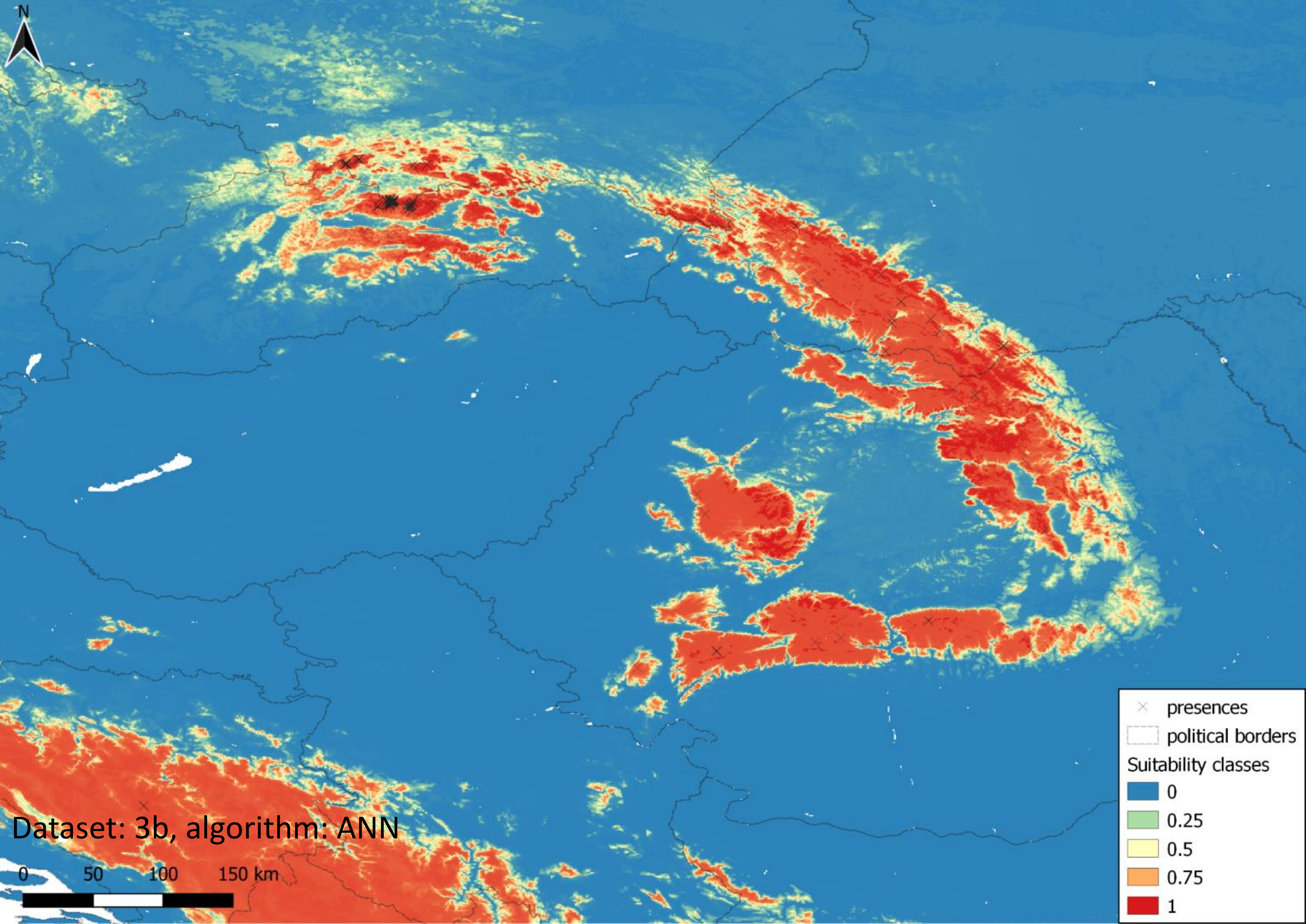
N
presences
political borders
Suitability classes
0
0.25
0.5
0.75
1
Dataset: 3b, algorithm: ANN
0
50
100
150 km

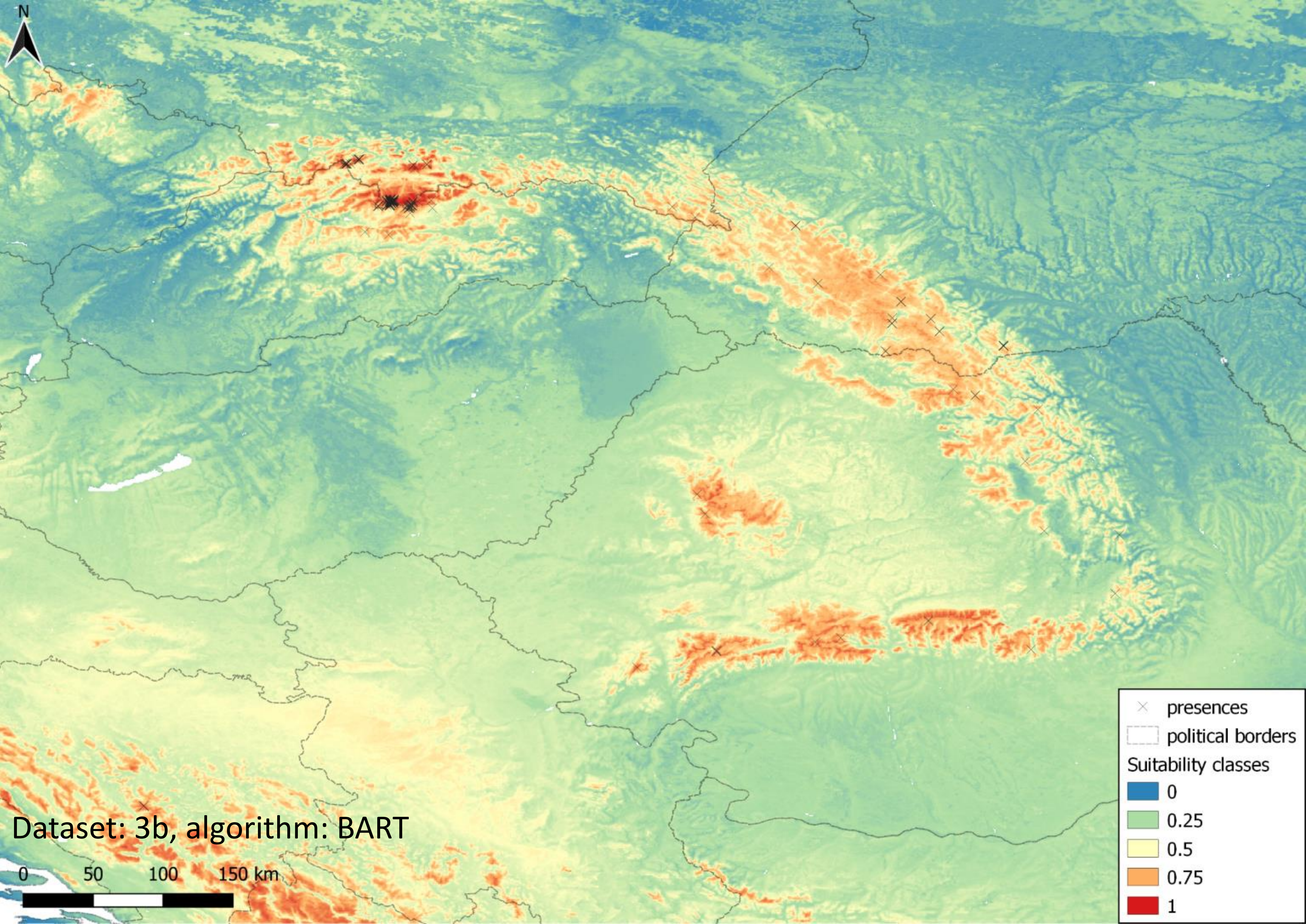
N
presences
political borders
Suitability classes
0
0.25
0.5
0.75
1
Dataset: 3b, algorithm: BART
0 50 100 150 km

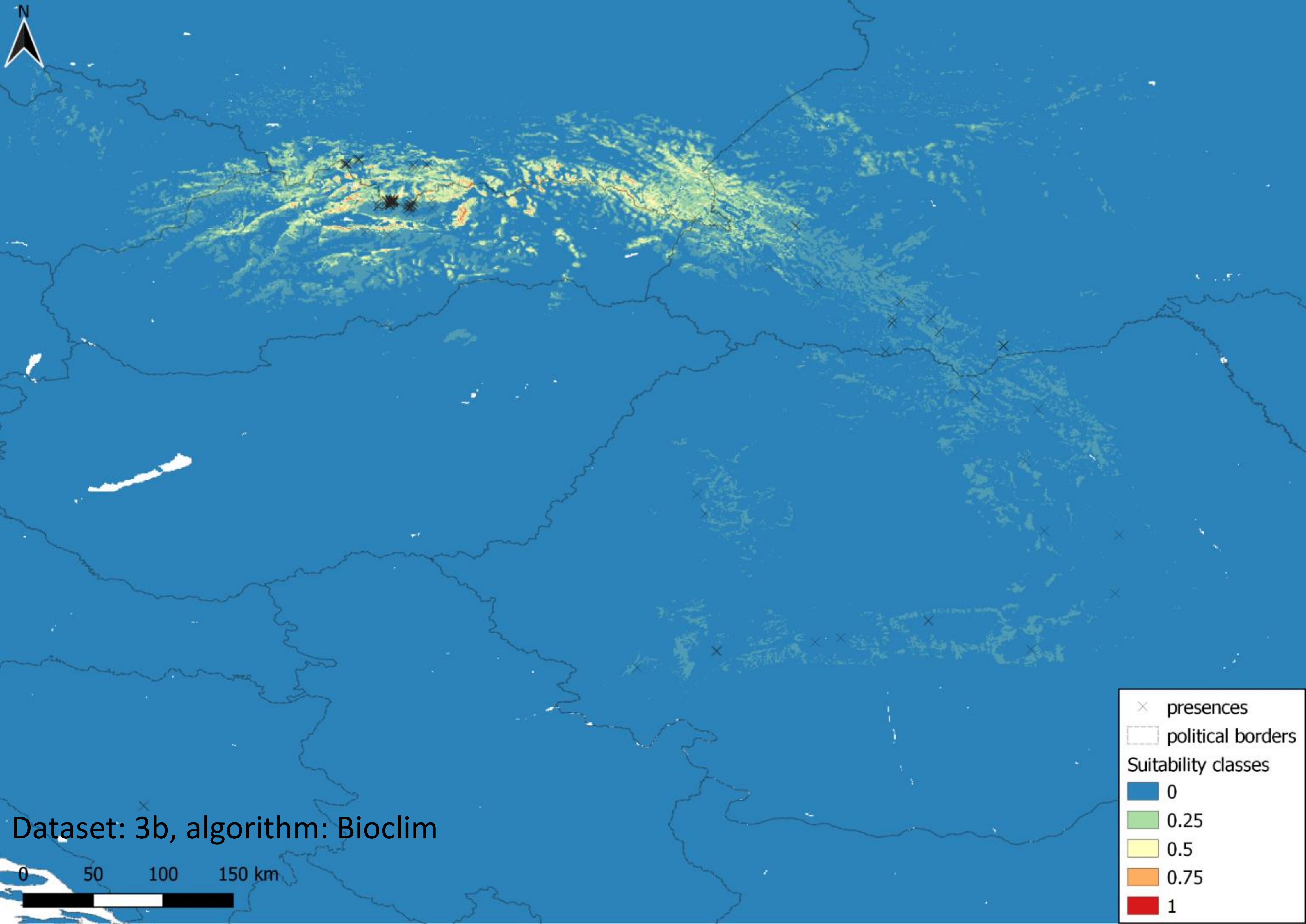
N
presences
political borders
Suitability classes
0
0.25
0.5
0.75
1
Dataset: 3b, algorithm: Bioclim
0
50
100
150 km

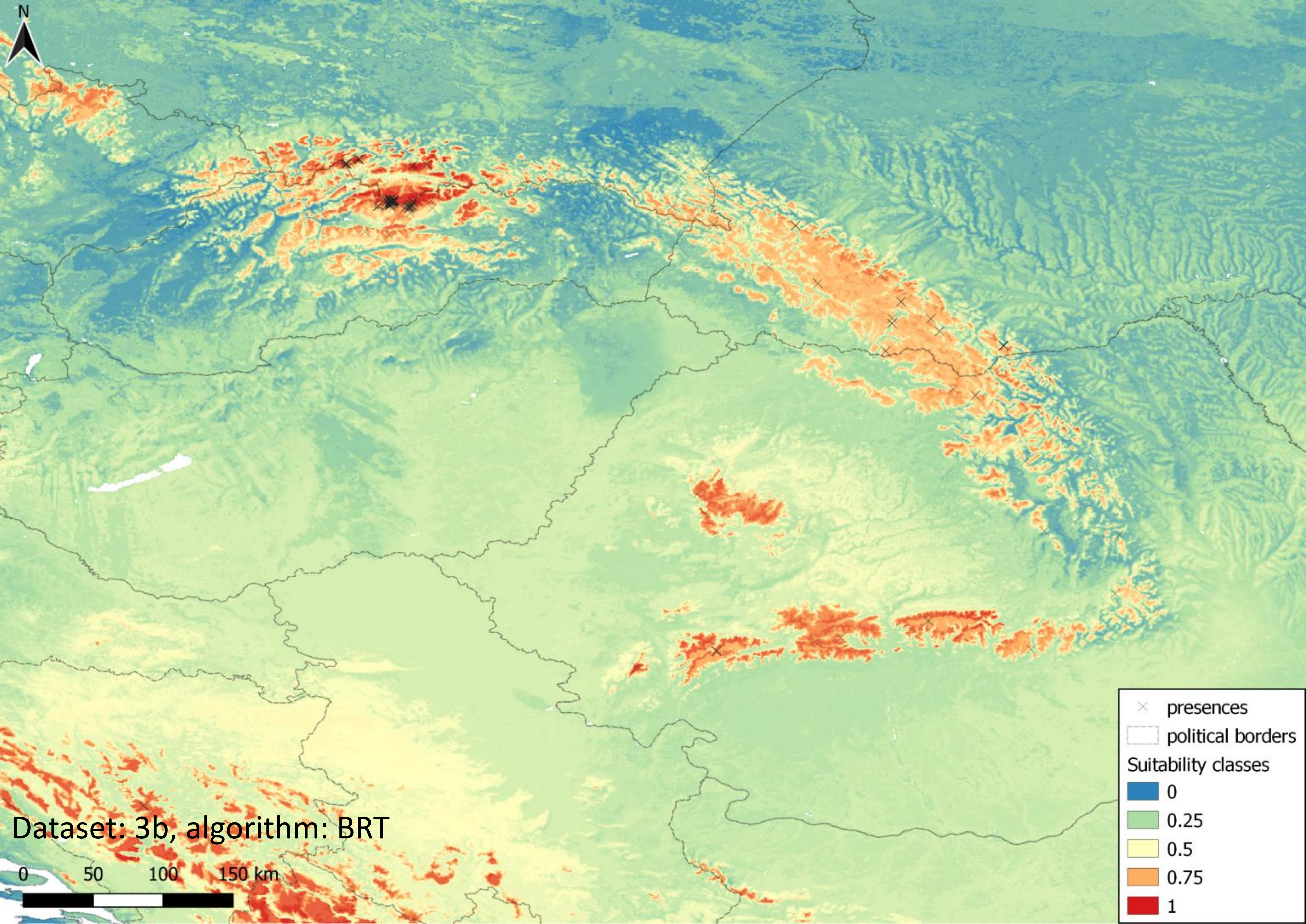

N
presences
political borders
Suitability classes
0
0.25
0.5
0.75
1
Dataset: 3b, algorithm: BRT
0
50
100
150 km

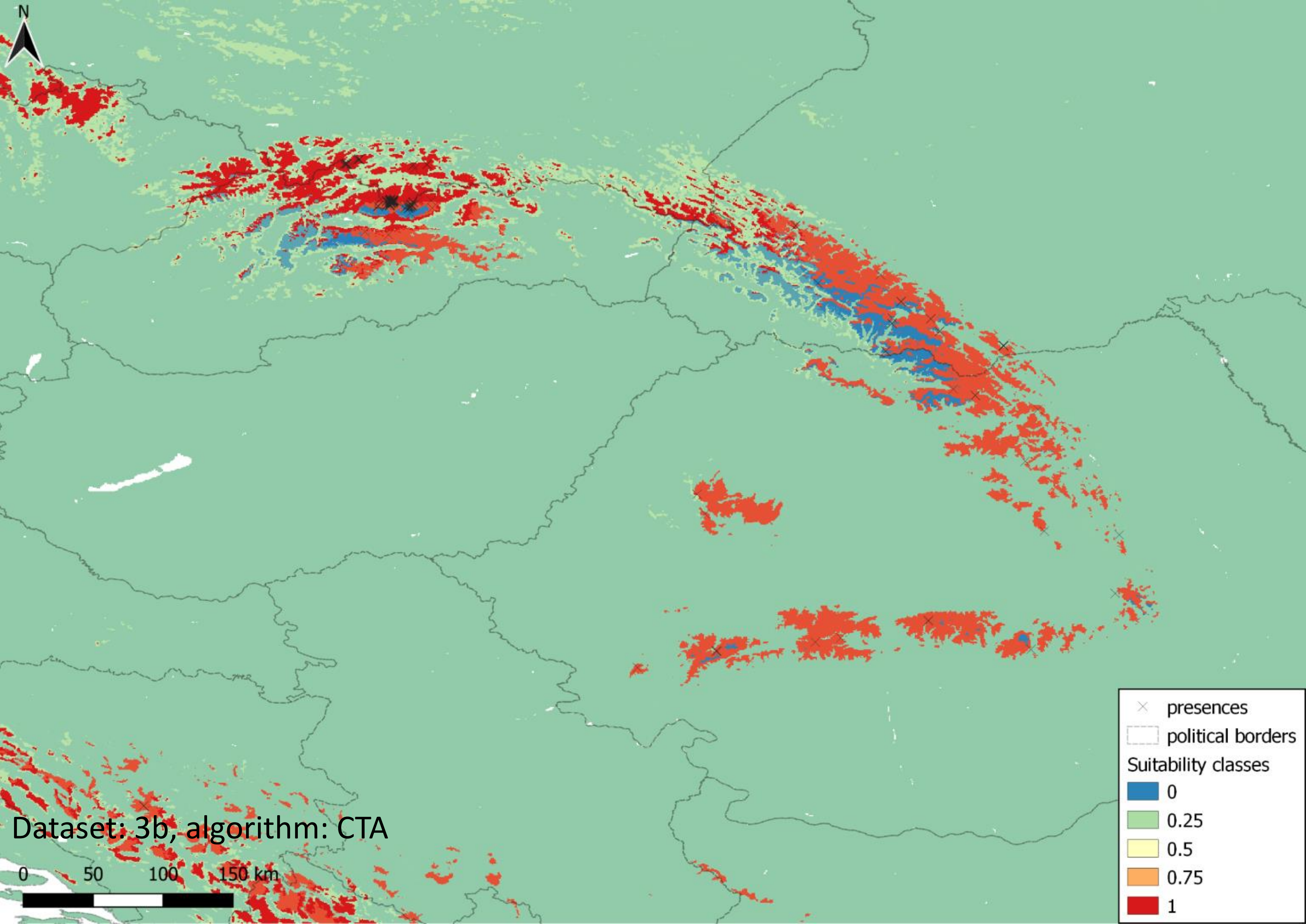
N
presences
political borders
Suitability classes
0
0.25
0.5
0.75
1
Dataset: 3b, algorithm: CTA
0
50
100
150 km

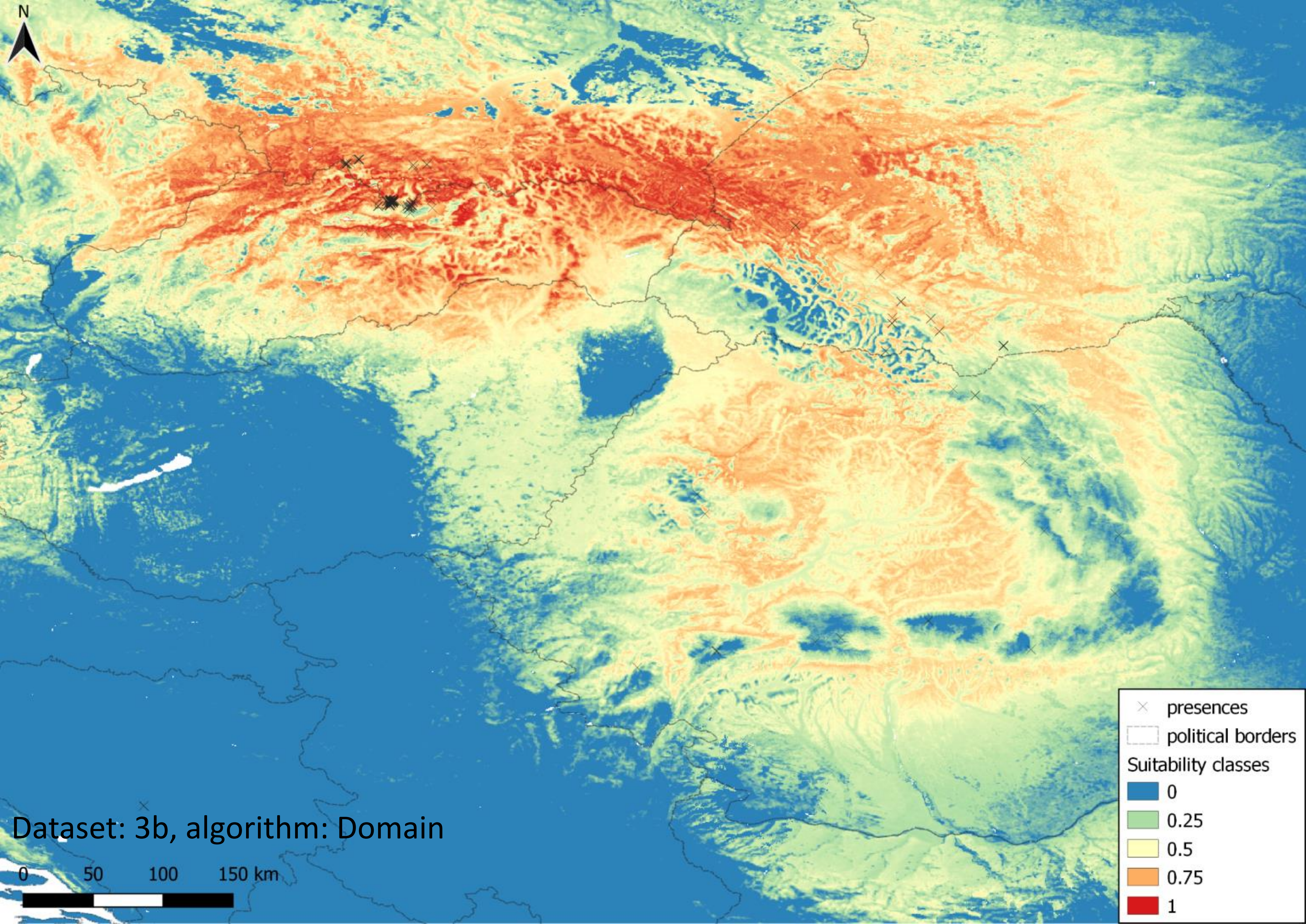
N
presences
political borders
Suitability classes
0
0.25
0.5
0.75
1
Dataset: 3b, algorithm: Domain
0
50
100
150 km

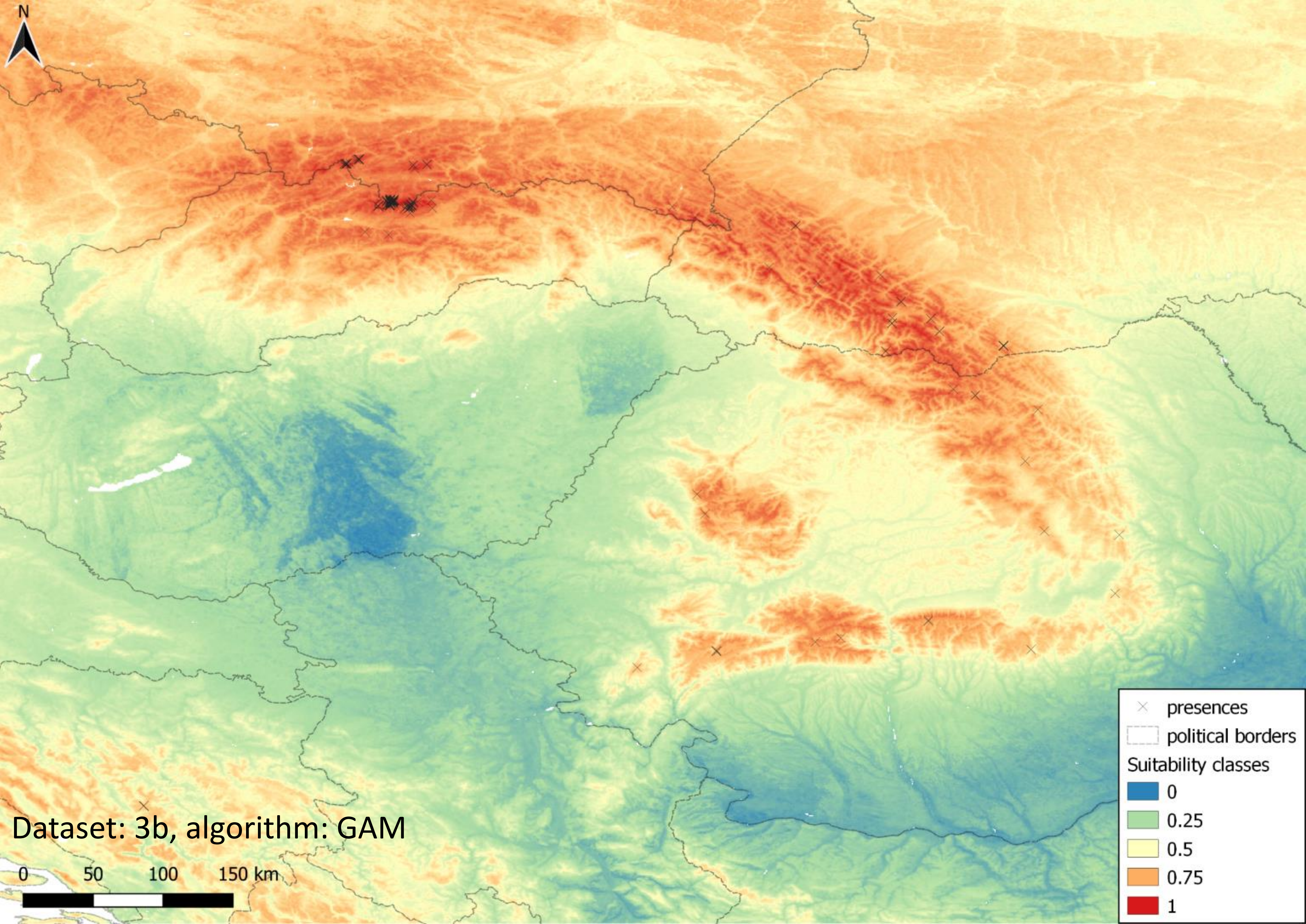
N
presences
political borders
Suitability classes
0
0.25
0.5
0.75
1
Dataset: 3b, algorithm: GAM
0
50
100
150 km

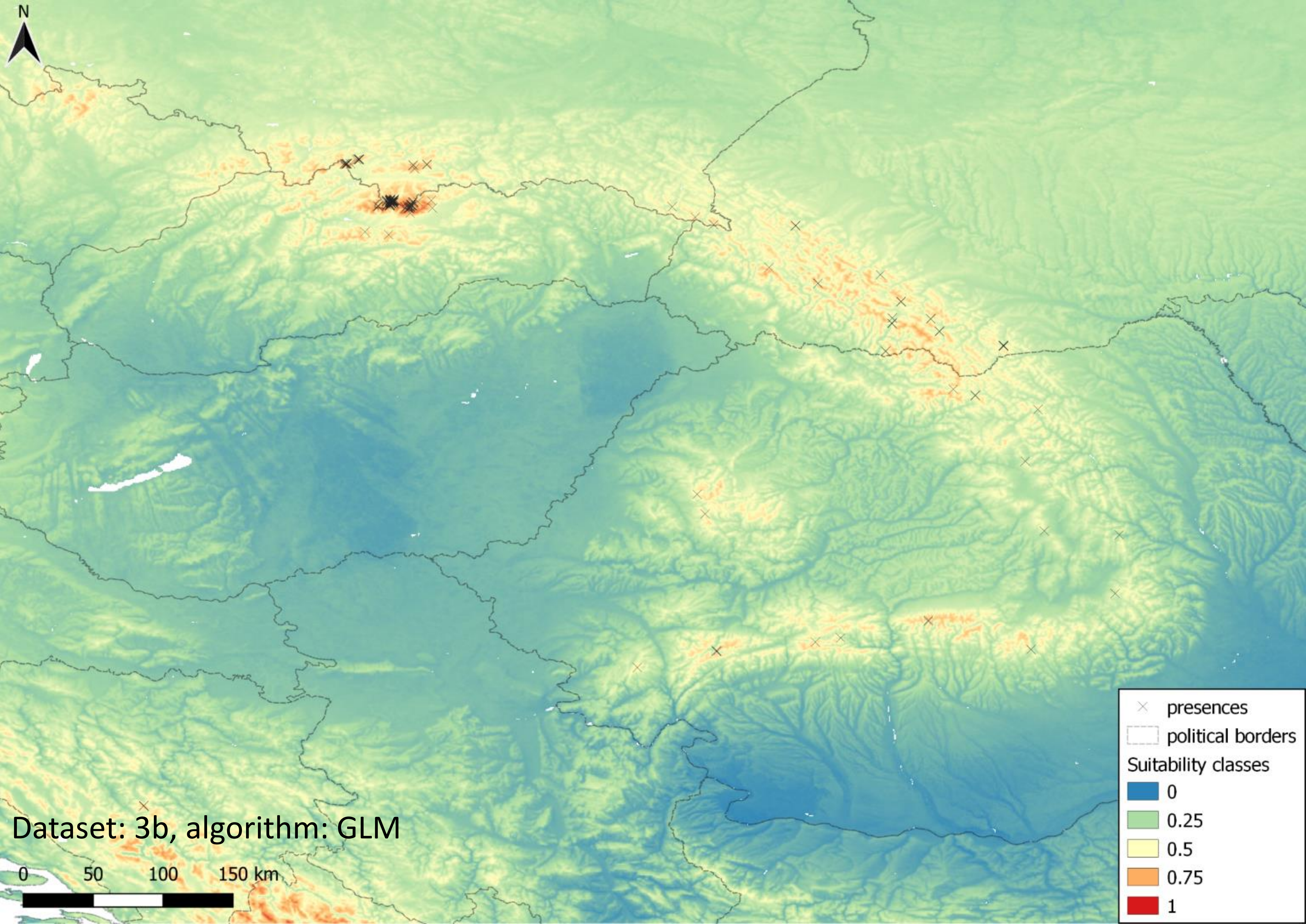
N
presences
political borders
Suitability classes
0
0.25
0.5
0.75
1
Dataset: 3b, algorithm: GLM
0
50
100
150 km

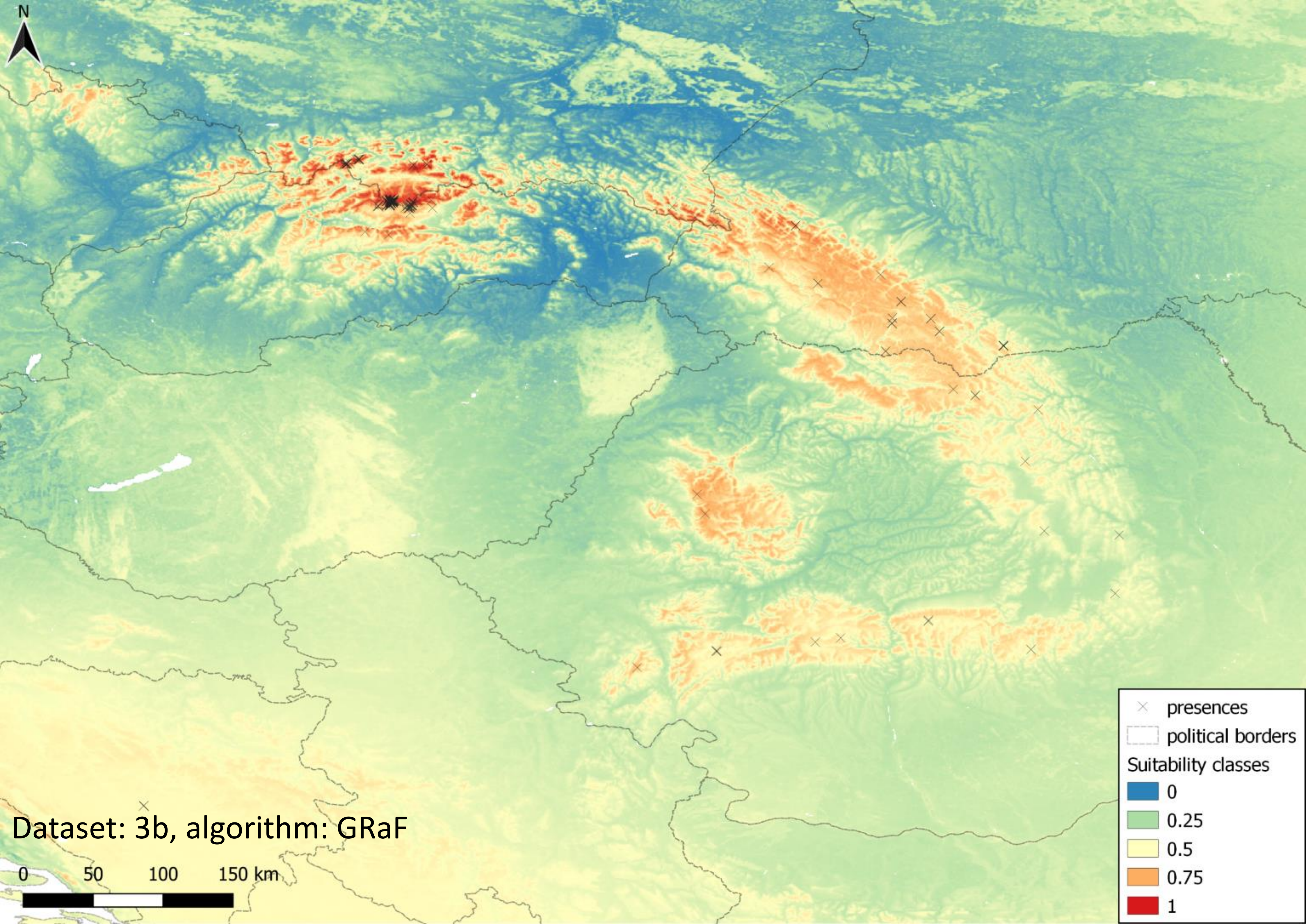
N
presences
political borders
Suitability classes
0
0.25
0.5
0.75
1
Dataset: 3b, algorithm: GRaF
0
50
100
150 km

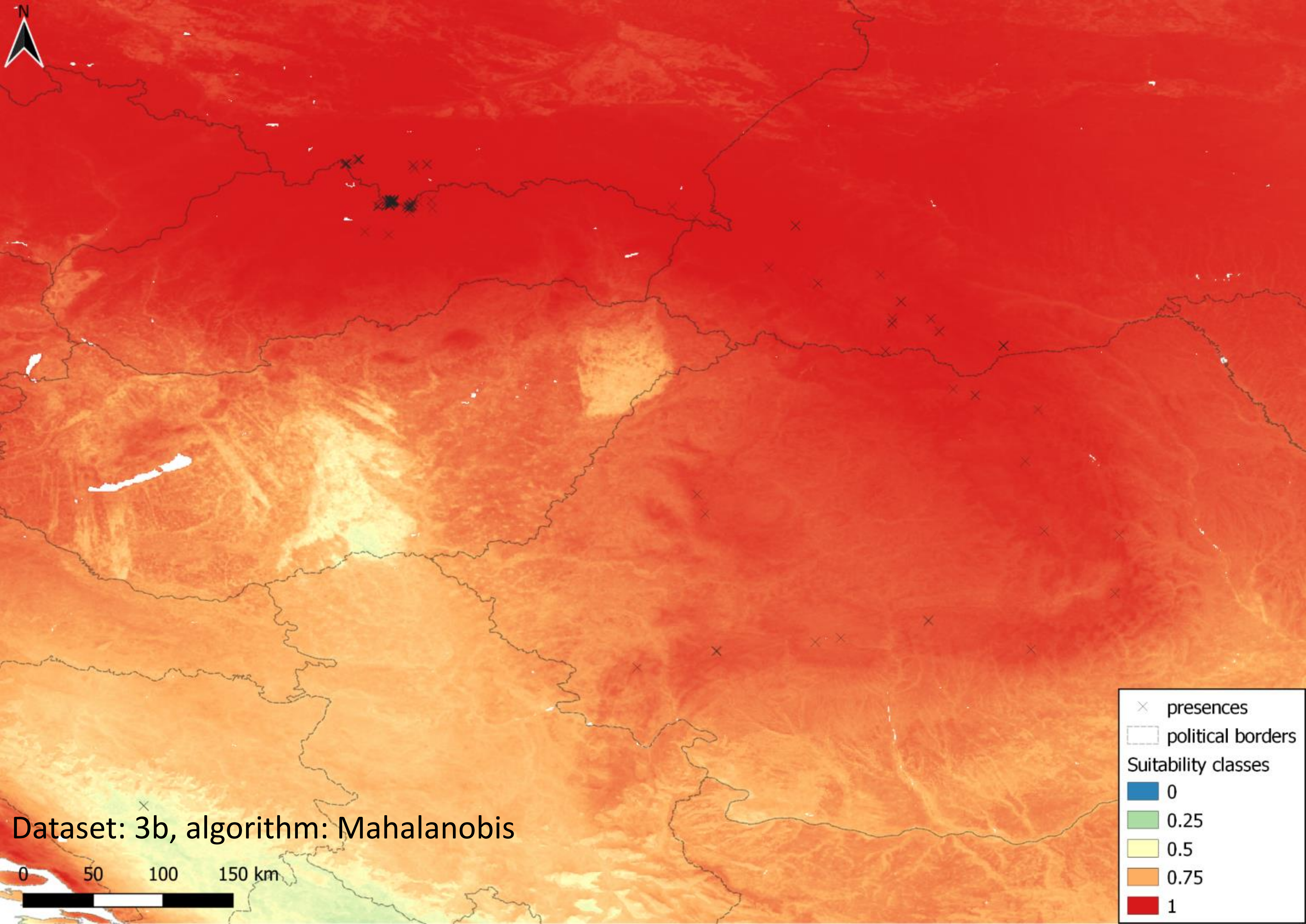
N
presences
political borders
Suitability classes
0
0.25
0.5
0.75
1
Dataset: 3b, algorithm: Mahalanobis
0
50
100
150 km

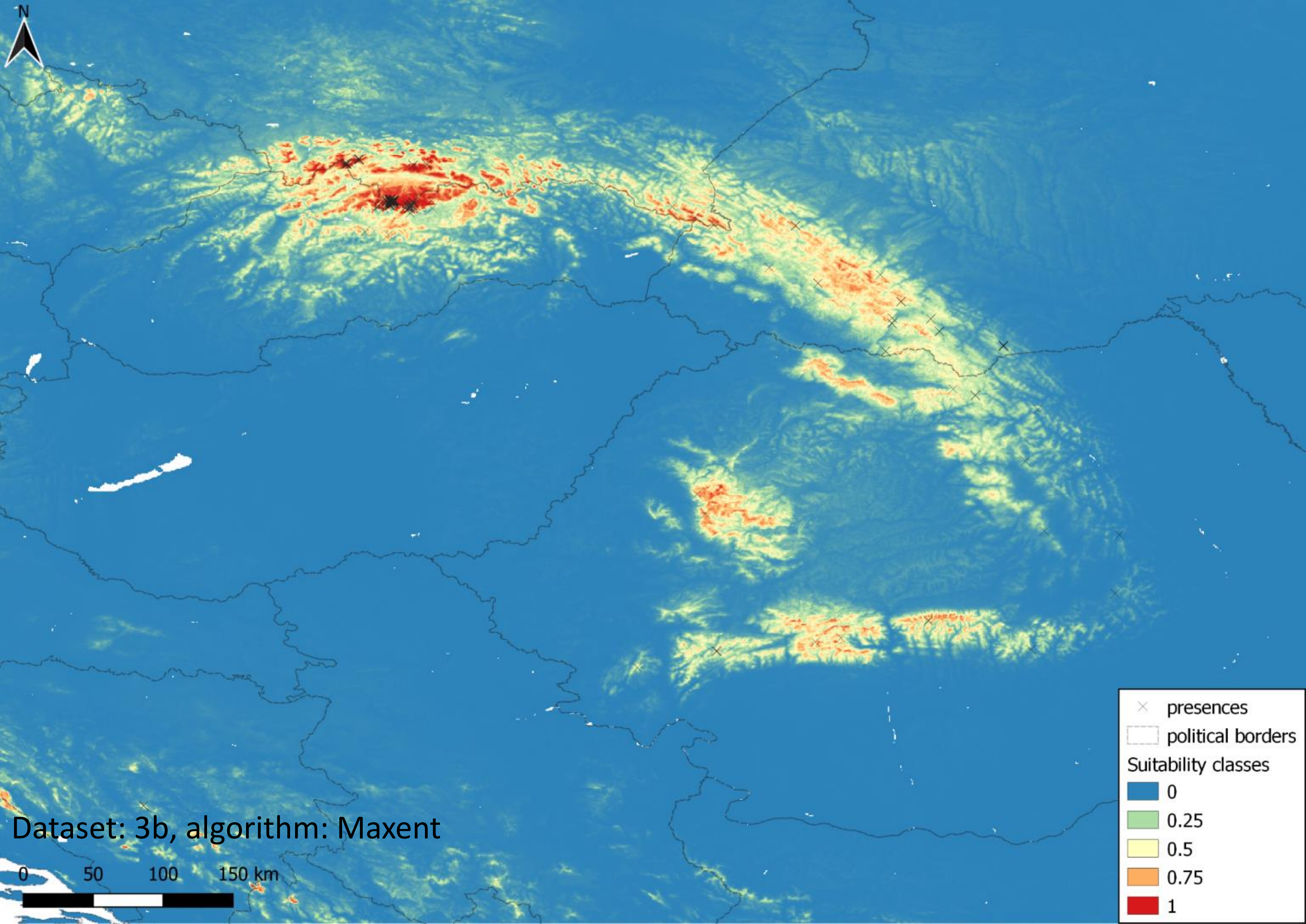
N
presences
political borders
Suitability classes
0
0.25
0.5
0.75
1
Dataset: 3b, algorithm: Maxent
0
50
100
150 km

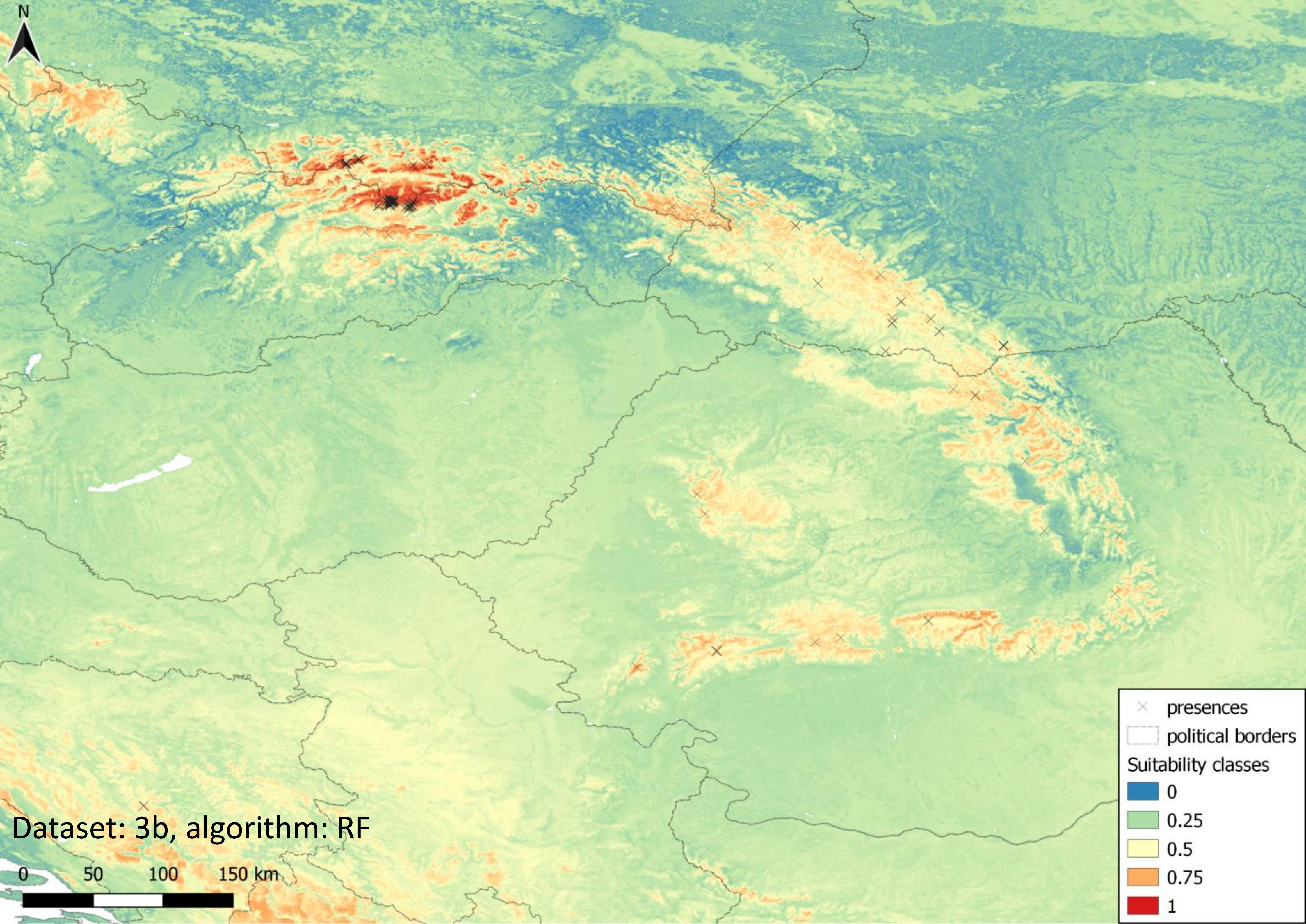
N
presences
political borders
Suitability classes
0
0.25
0.5
0.75
1
Dataset: 3b, algorithm: RF
0
50
100
150 km

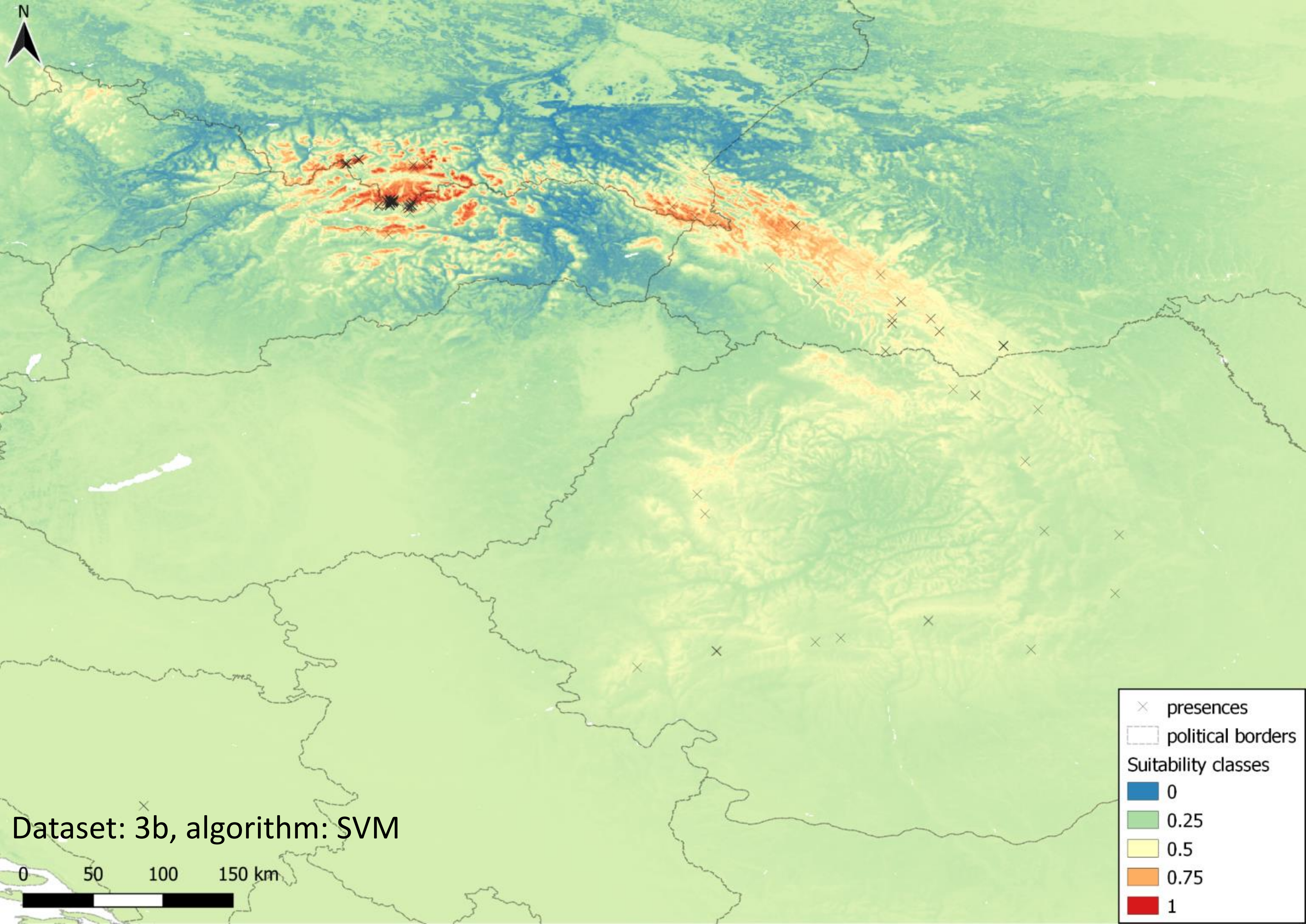
N
Dataset: 3b, algorithm: SVM
0
50
100
150 km
presences
political borders
Suitability classes
0
0.25
0.5
0.75
1

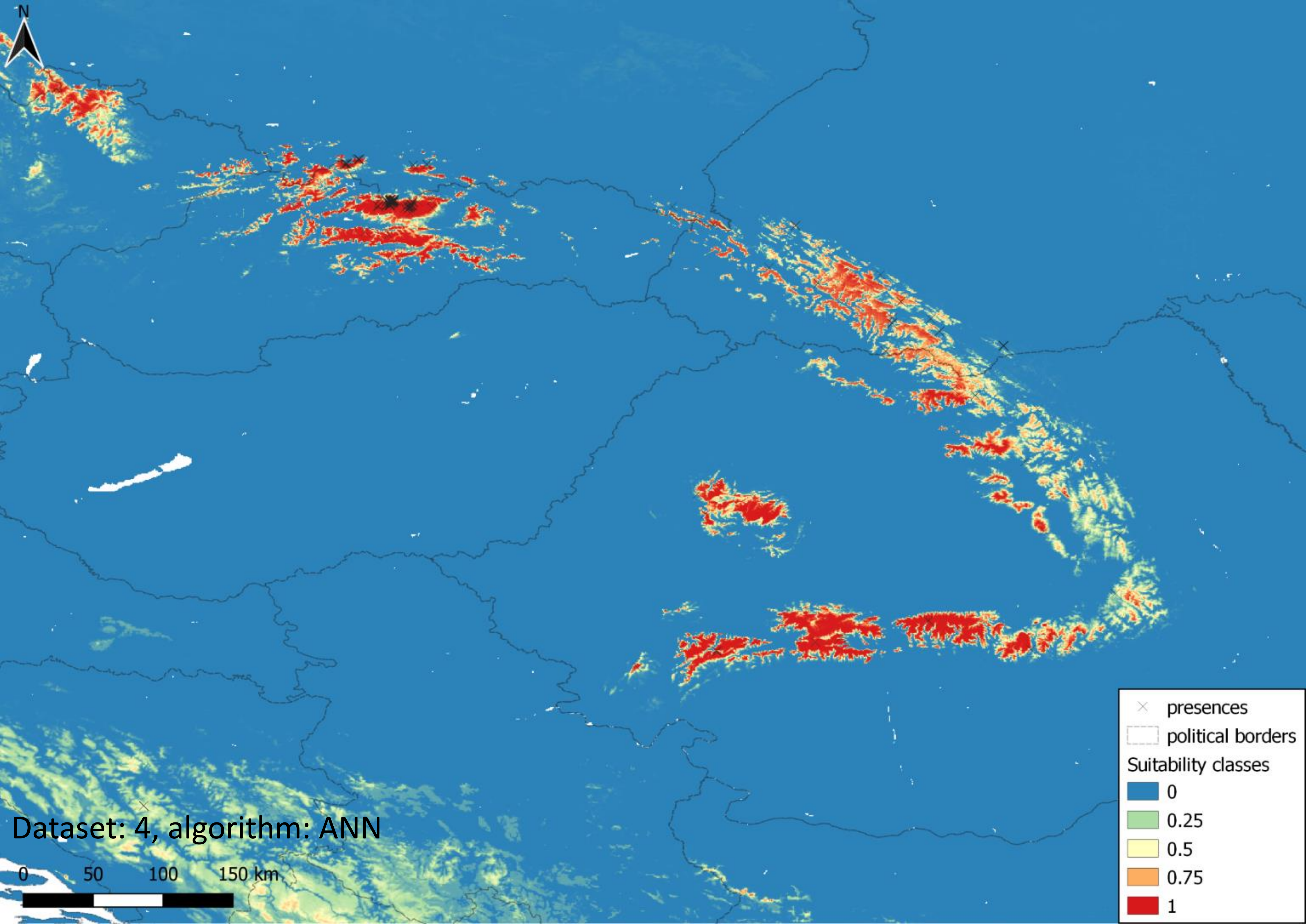
N
presences
political borders
Suitability classes
0
0.25
0.5
0.75
1
Dataset: 4, algorithm: ANN
0
50
100
150 km

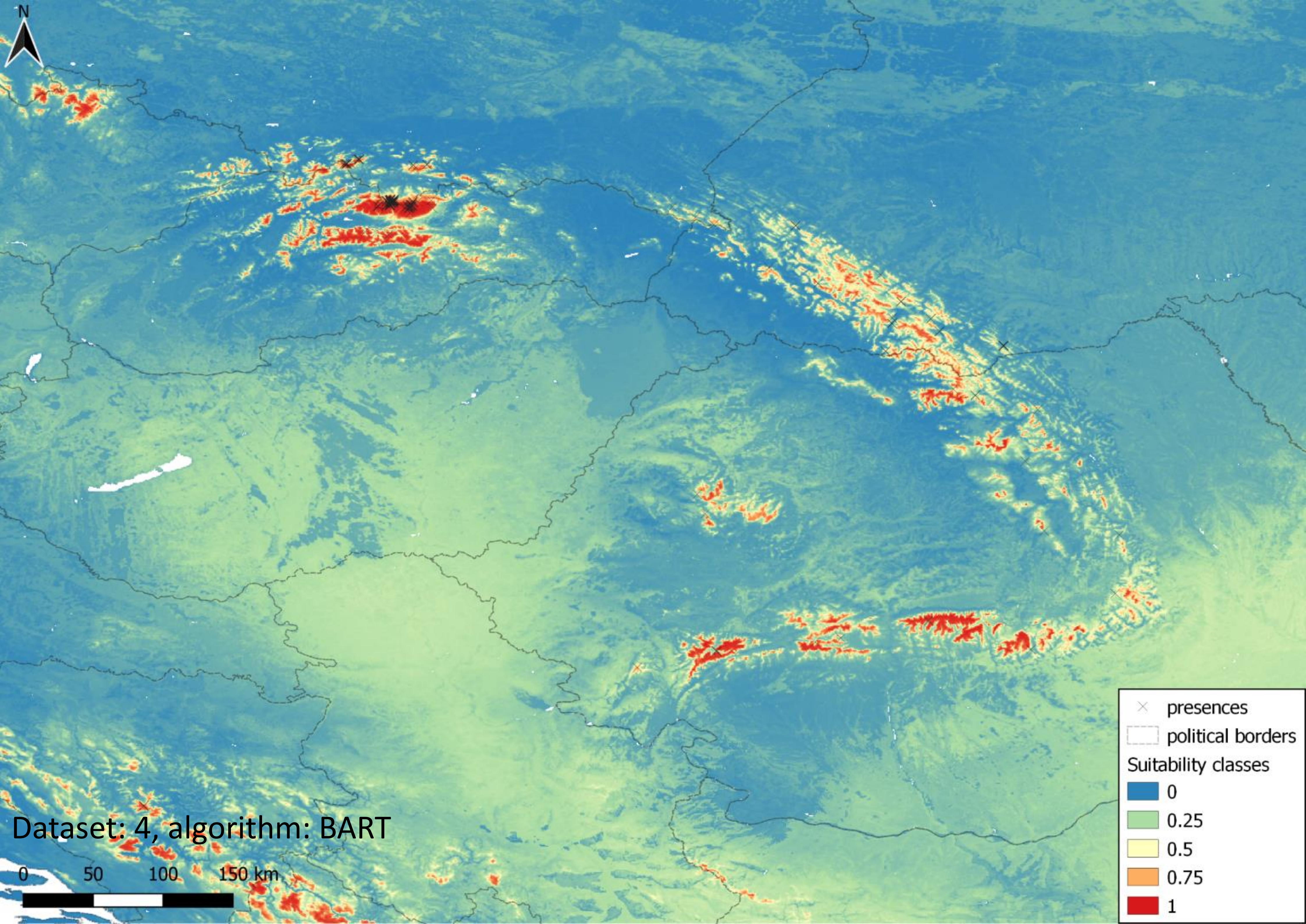
N
presences
political borders
Suitability classes
0
0.25
0.5
0.75
1
Dataset: 4, algorithm: BART
0
50
100
150 km

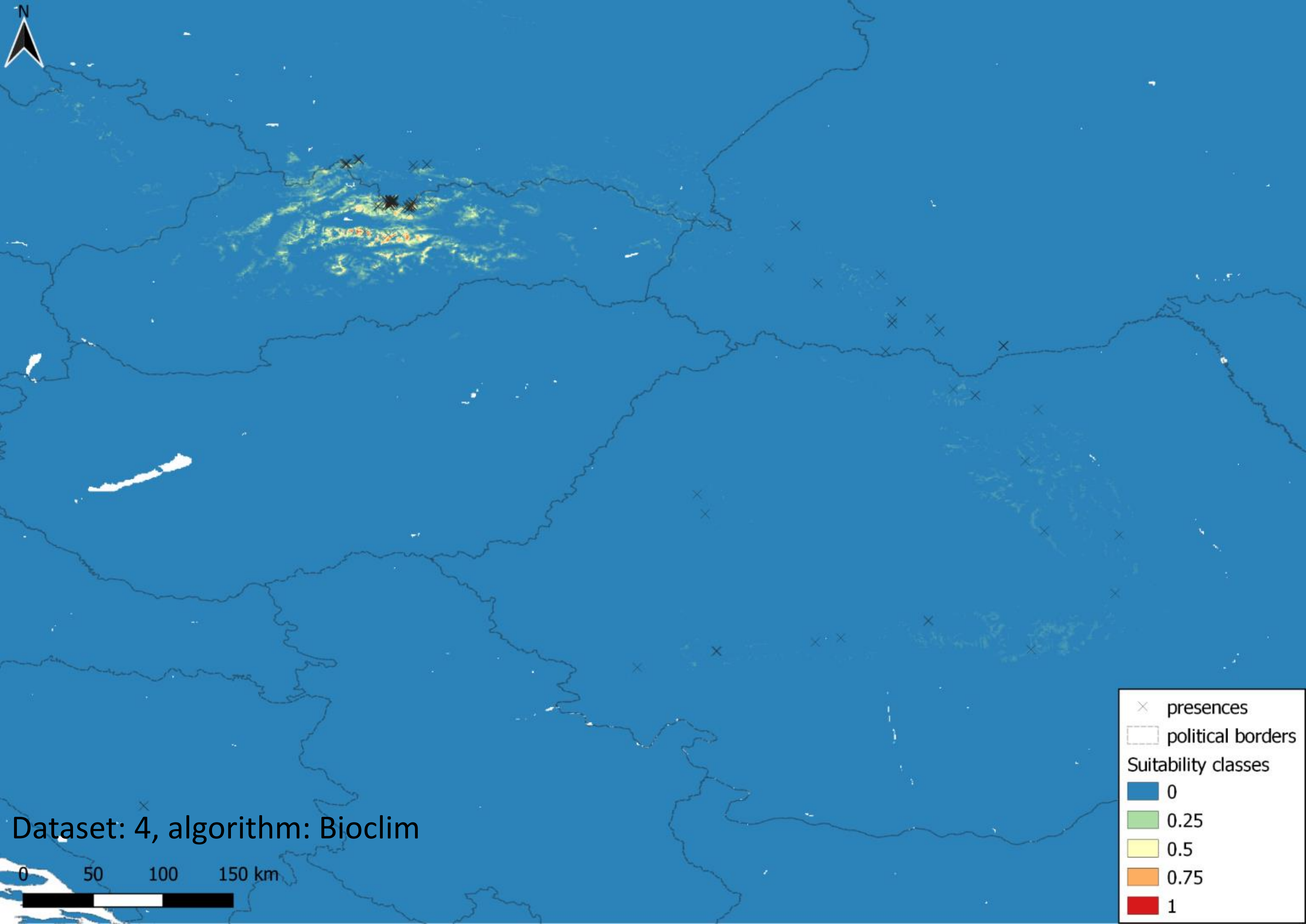
N
presences
political borders
Suitability classes
0
0.25
0.5
0.75
1
Dataset: 4, algorithm: Bioclim
0
50
100
150 km

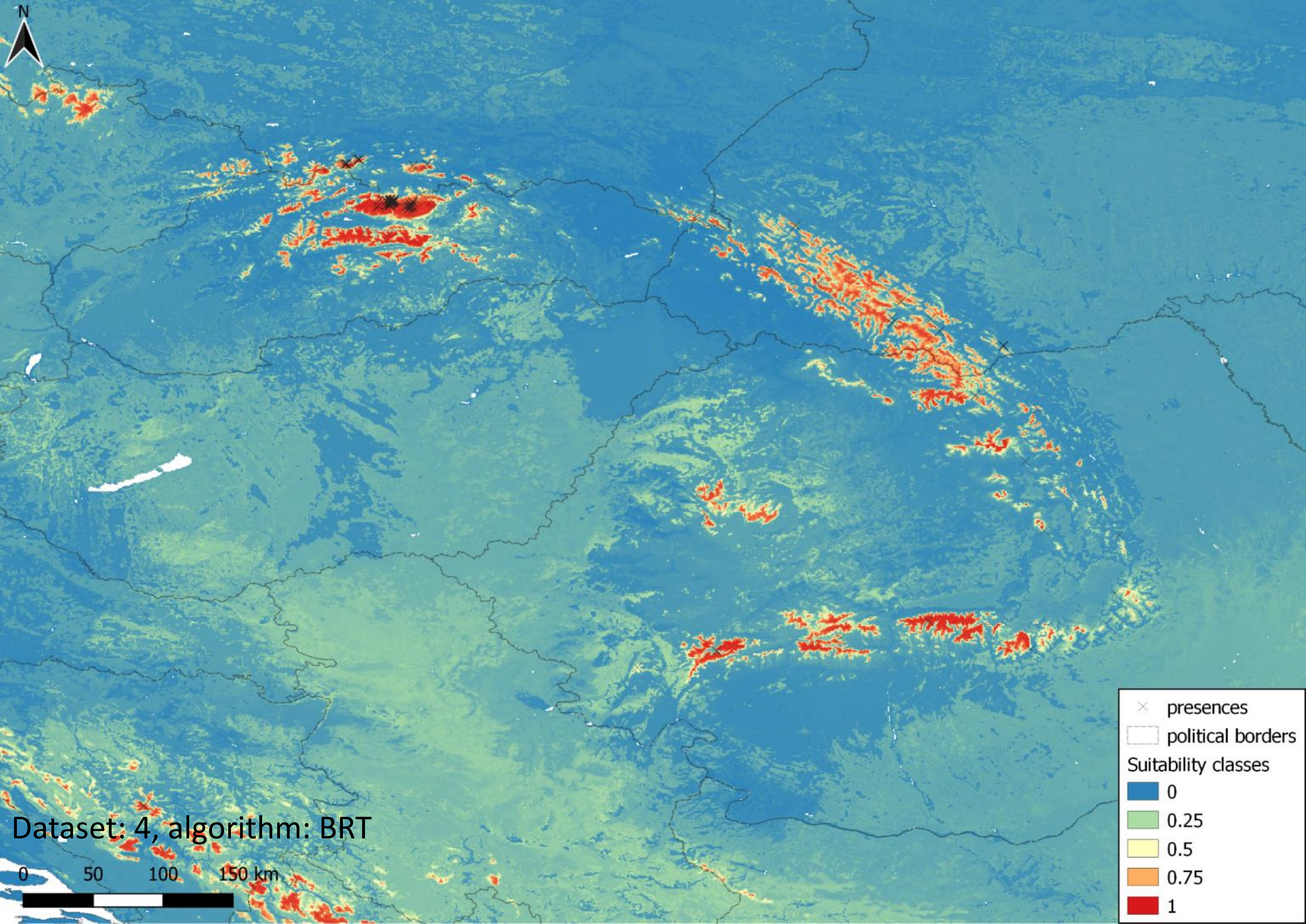
N
presences
political borders
Suitability classes
0
0.25
0.5
0.75
1
Dataset: 4, algorithm: BRT
0
50
100
150 km

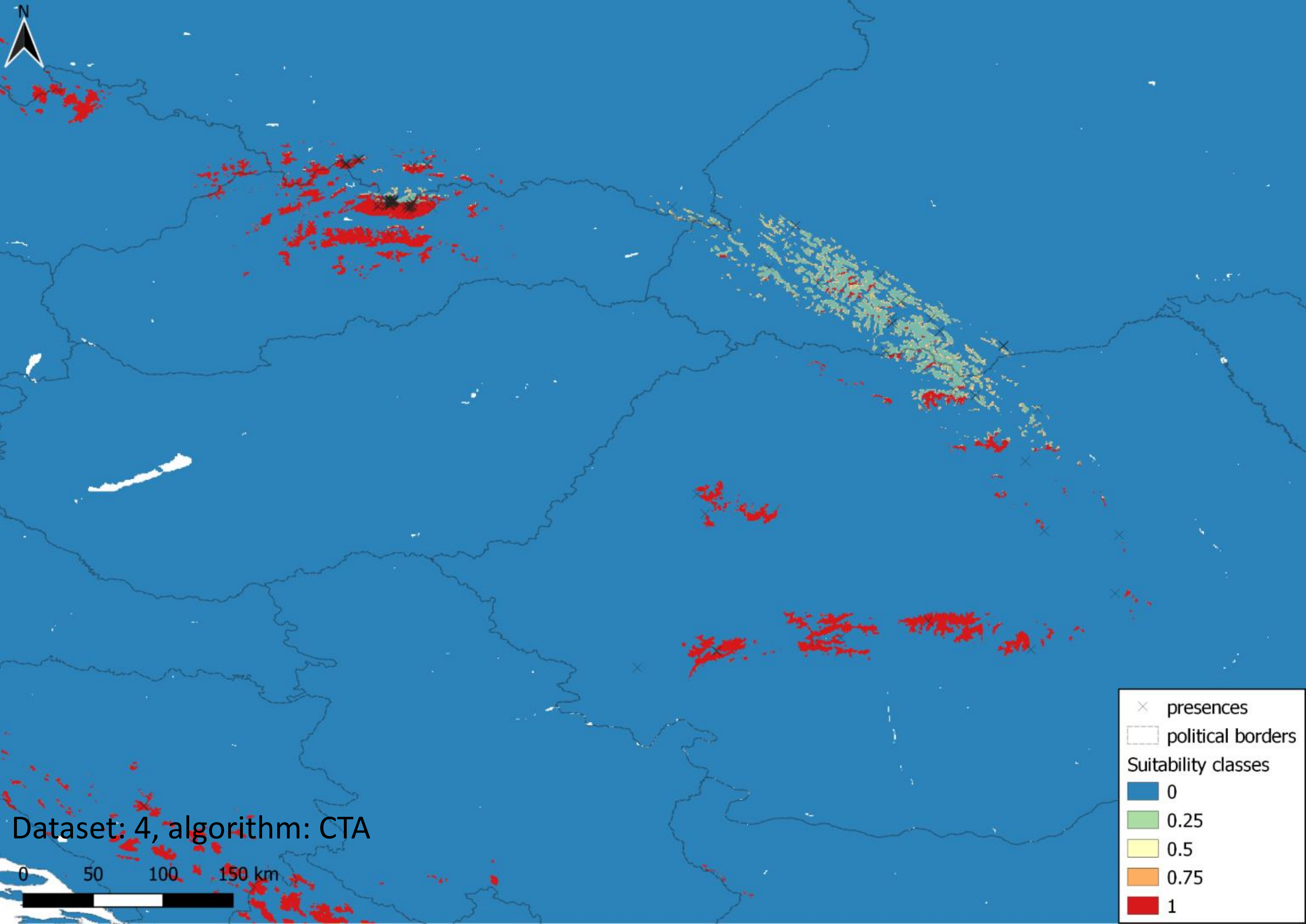
N
presences
political borders
Suitability classes
0
0.25
0.5
0.75
1
Dataset: 4, algorithm: CTA
0
50
100
150 km

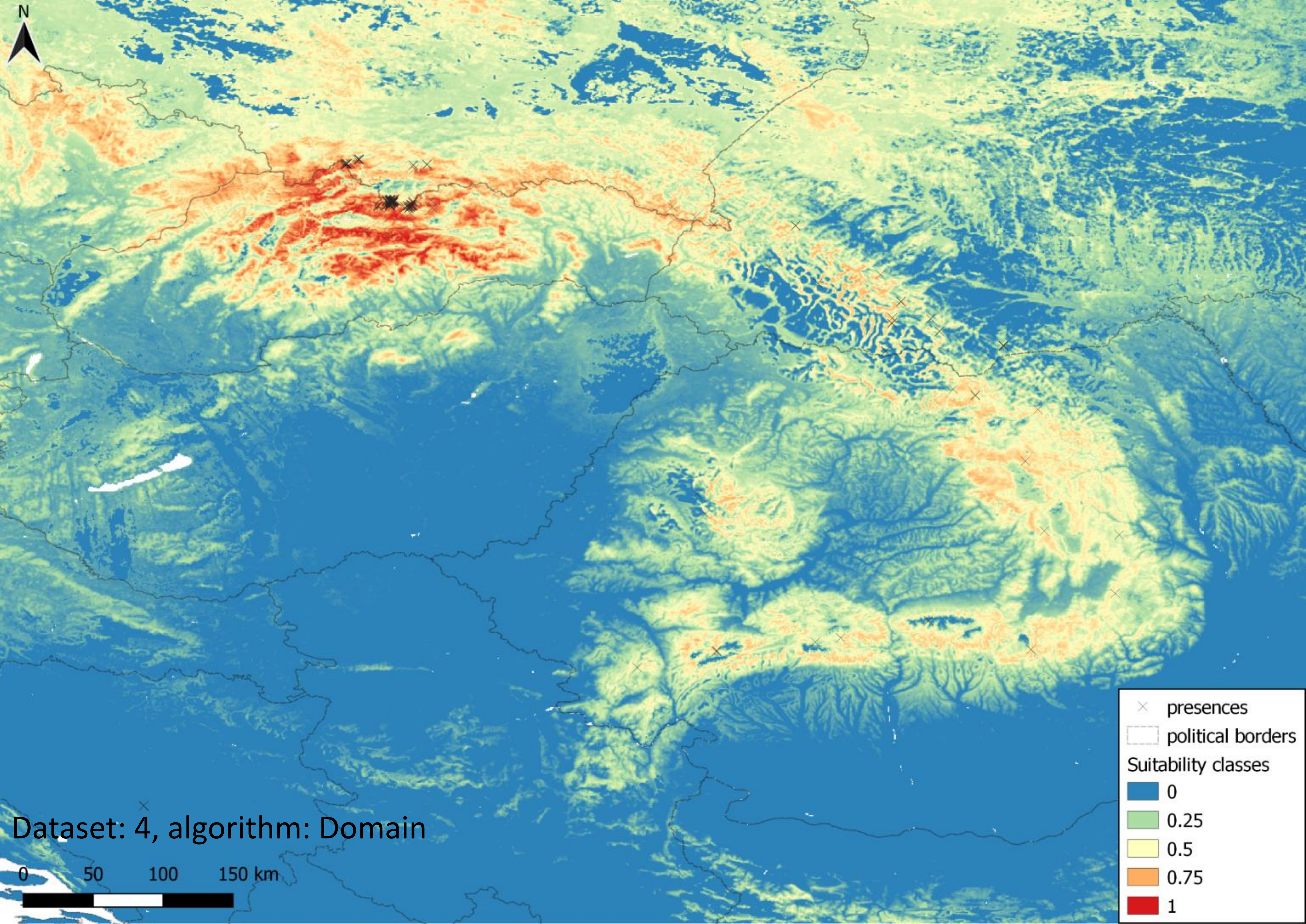
N
Dataset: 4, algorithm: Domain
0
50
100
150 km
presences
political borders
Suitability classes
0
0.25
0.5
0.75
1

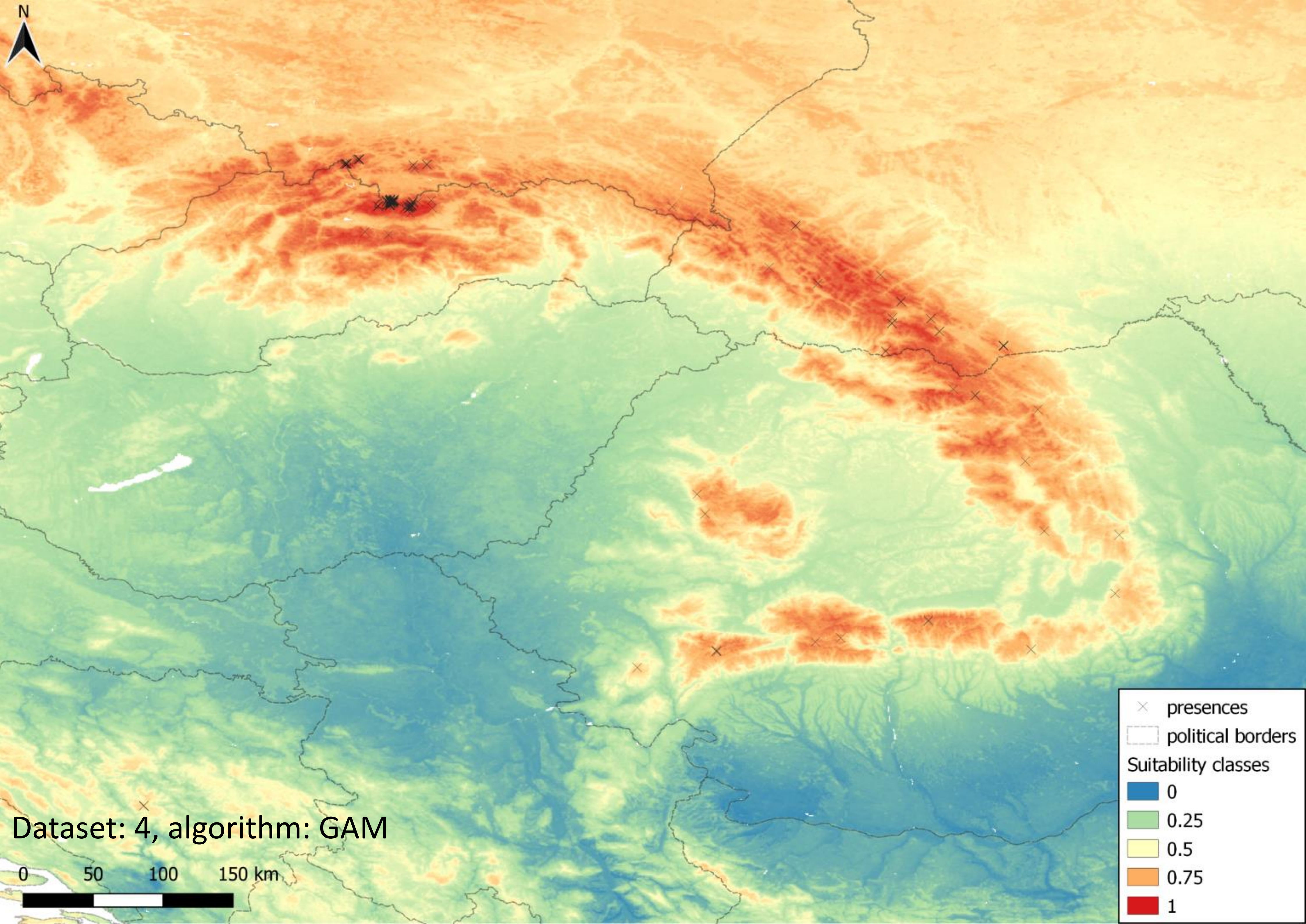
N
presences
political borders
Suitability classes
0
0.25
0.5
0.75
1
Dataset: 4, algorithm: GAM
0
50
100
150 km

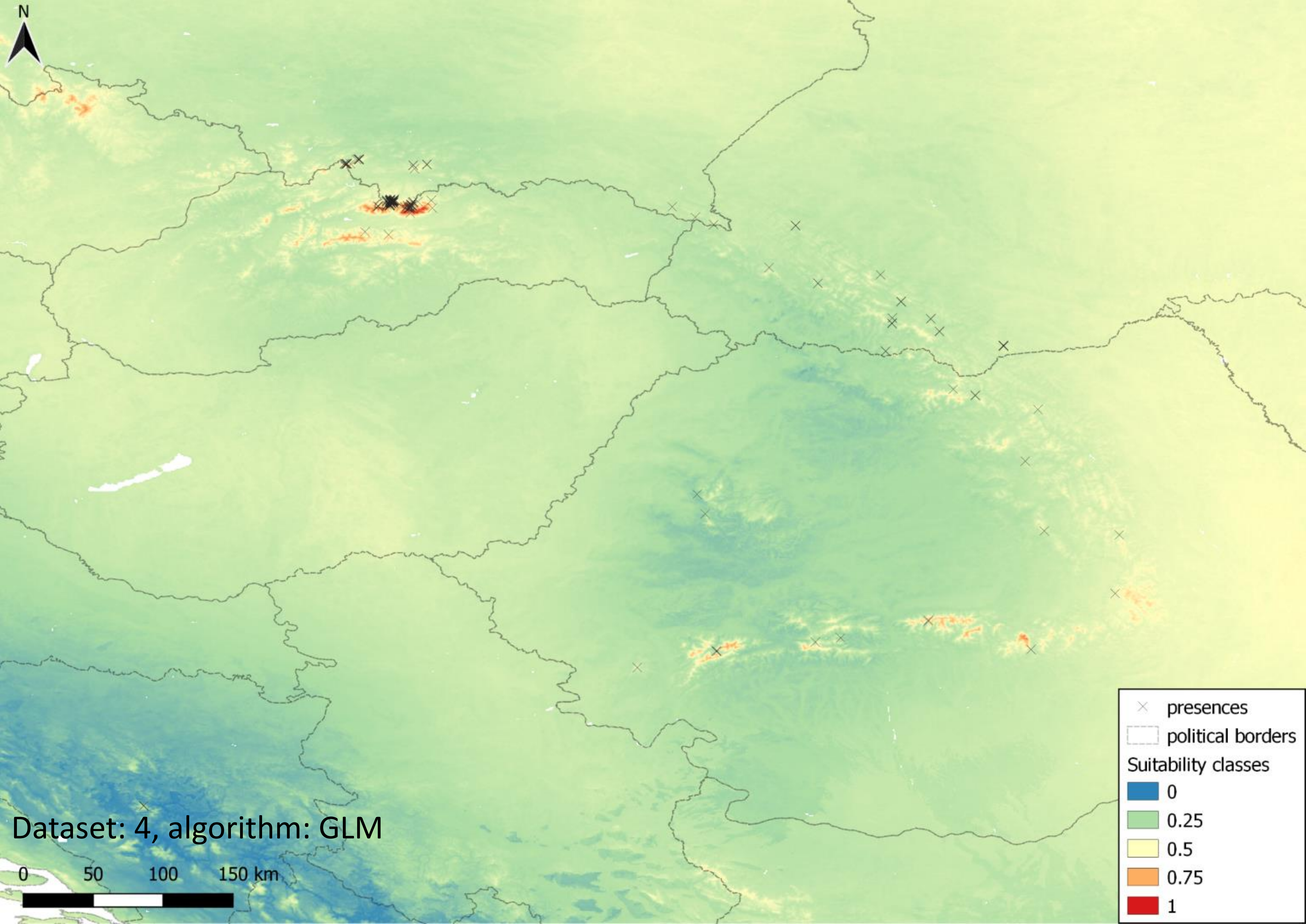
N
Dataset: 4, algorithm: GLM
0
50
100
150 km
presences
political borders
Suitability classes
0
0.25
0.5
0.75
1

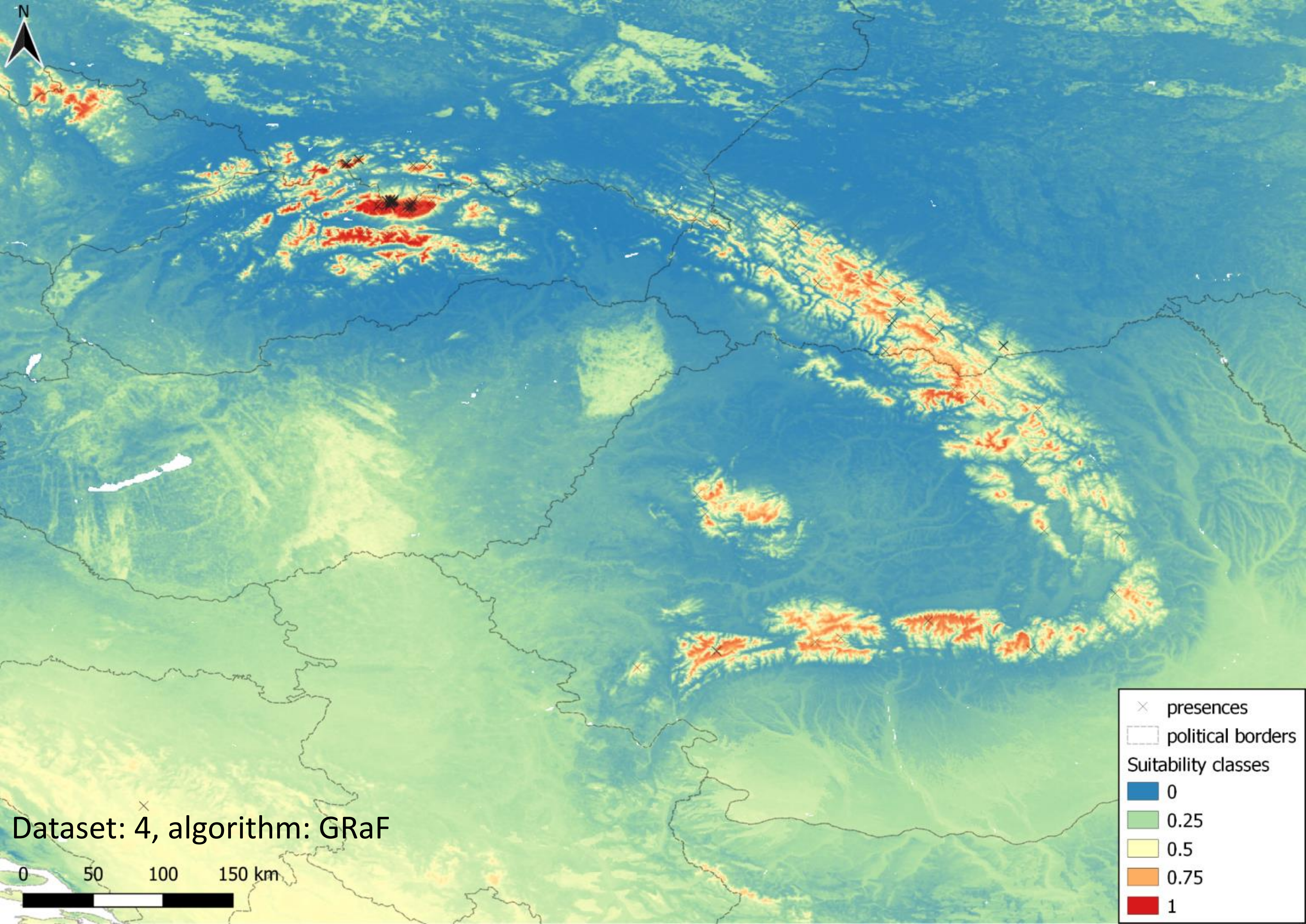
N
Dataset: 4, algorithm: GRaF
0
50
100
150 km
presences
political borders
Suitability classes
0
0.25
0.5
0.75
1

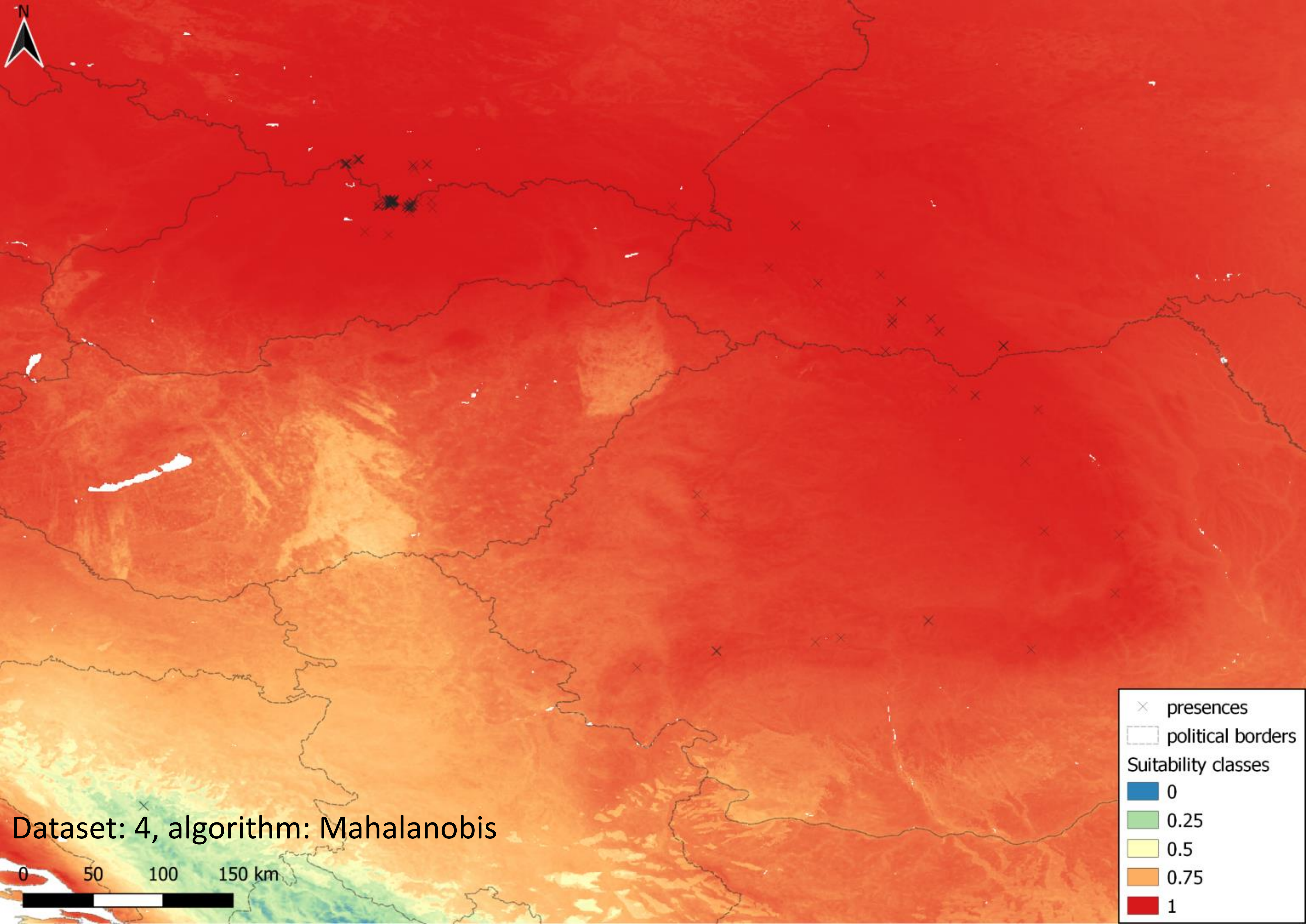
N
Dataset: 4, algorithm: Mahalanobis
0
50
100
150 km
presences
political borders
Suitability classes
0
0.25
0.5
0.75
1

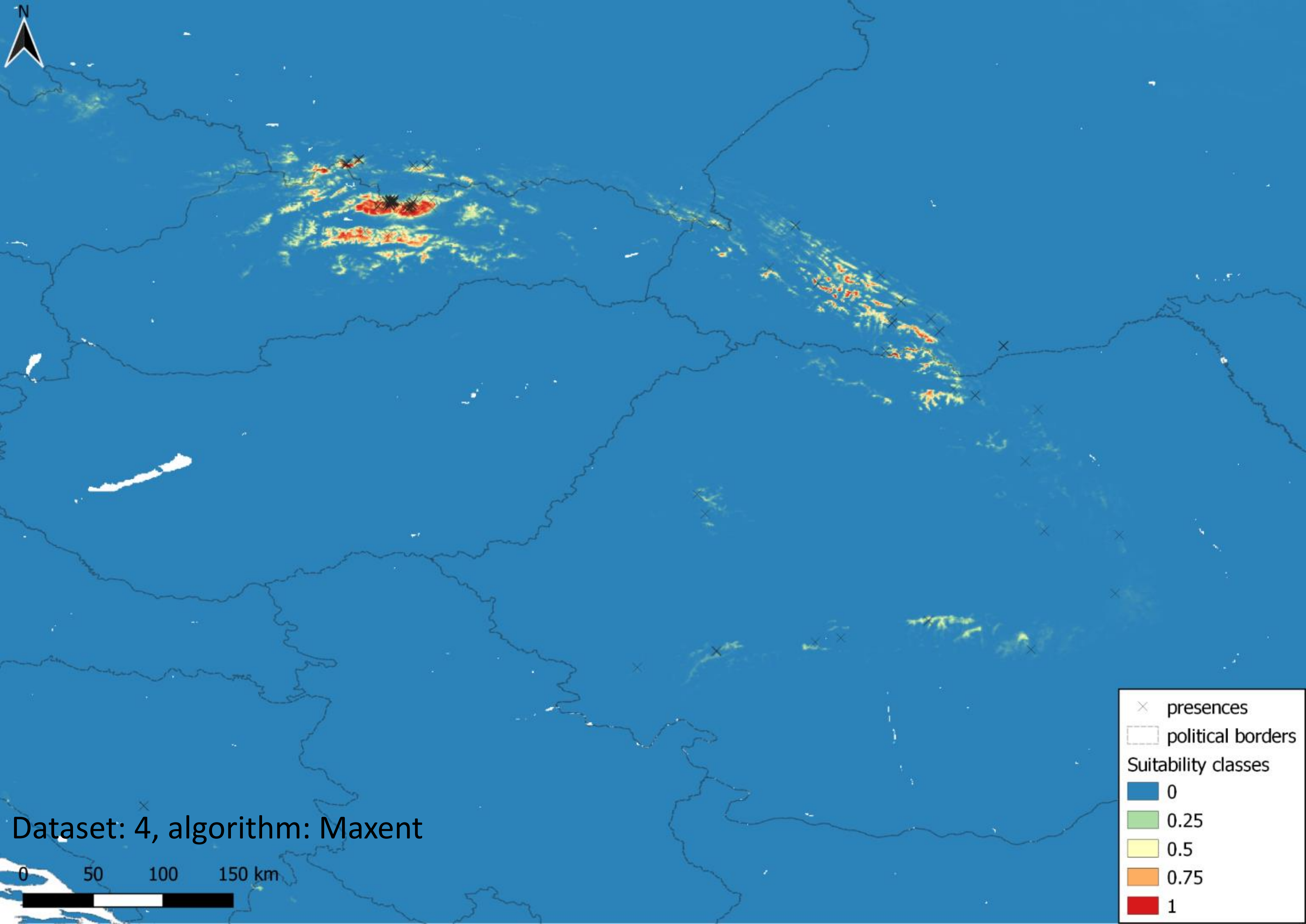
N
presences
political borders
Suitability classes
0
0.25
0.5
0.75
1
Dataset: 4, algorithm: Maxent
0
50
100
150 km

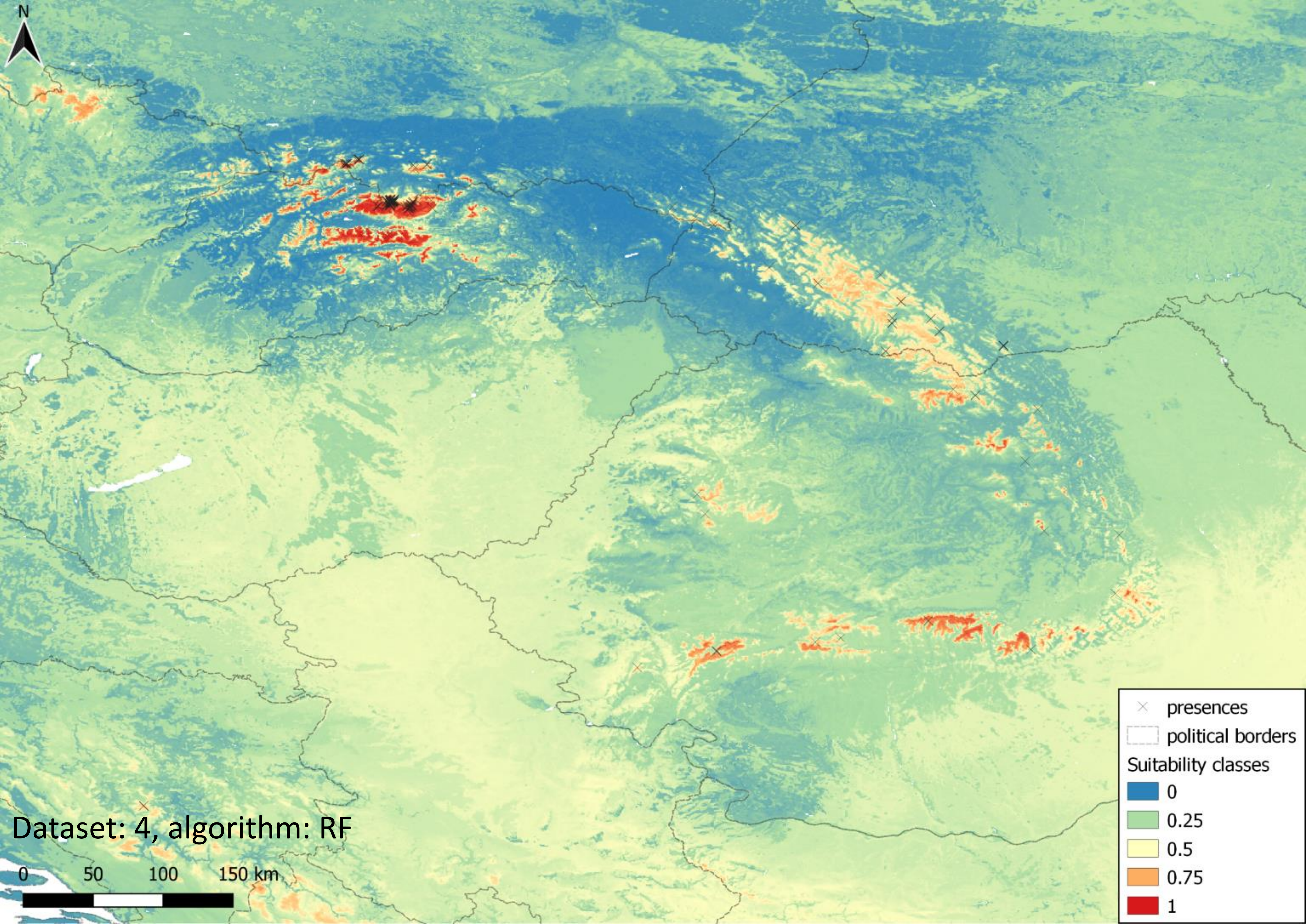
N
presences
political borders
Suitability classes
0
0.25
0.5
0.75
1
Dataset: 4, algorithm: RF
0
50
100
150 km

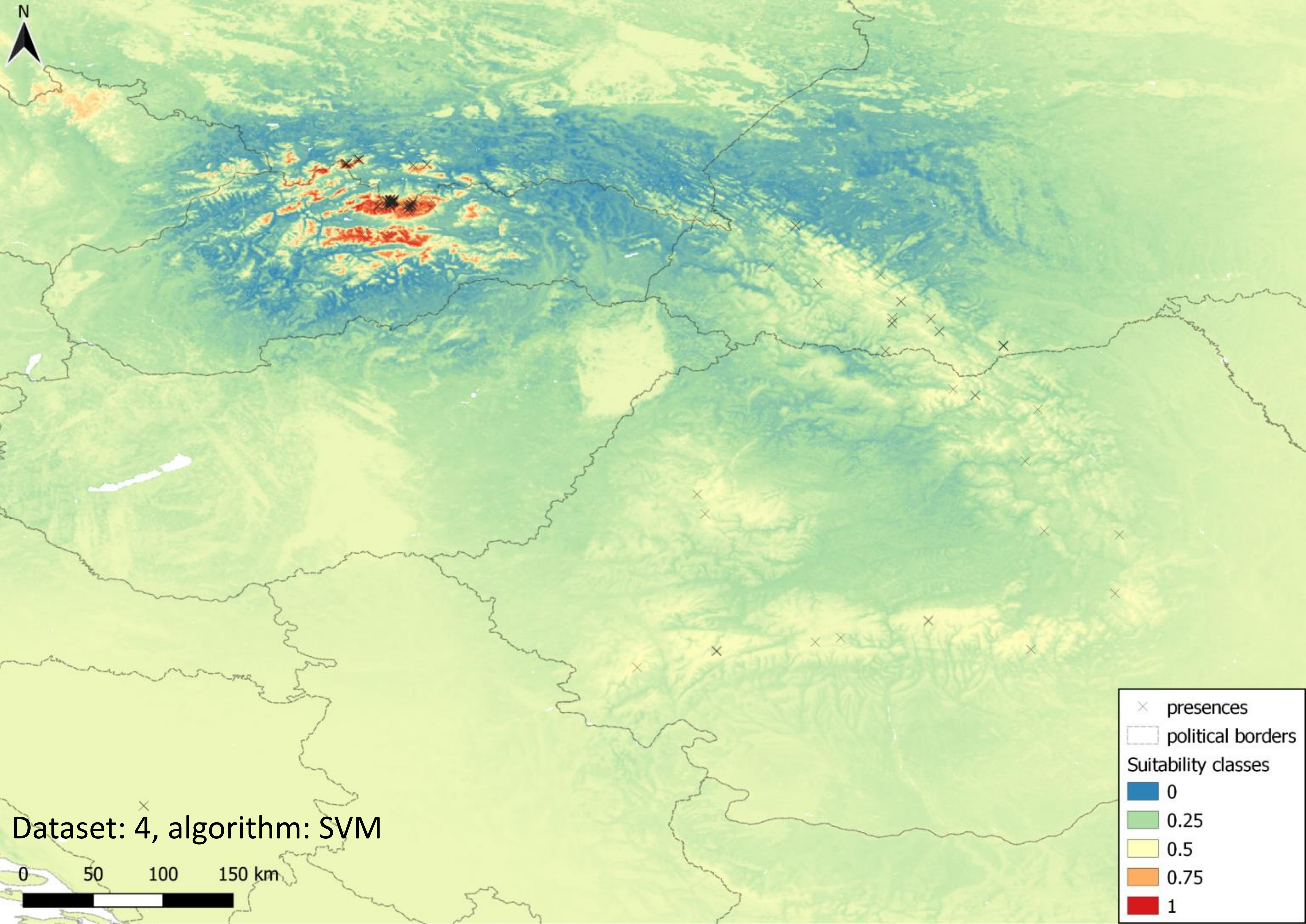
N
presences
political borders
Suitability classes
0
0.25
0.5
0.75
1
Dataset: 4, algorithm: SVM
0
50
100
150 km

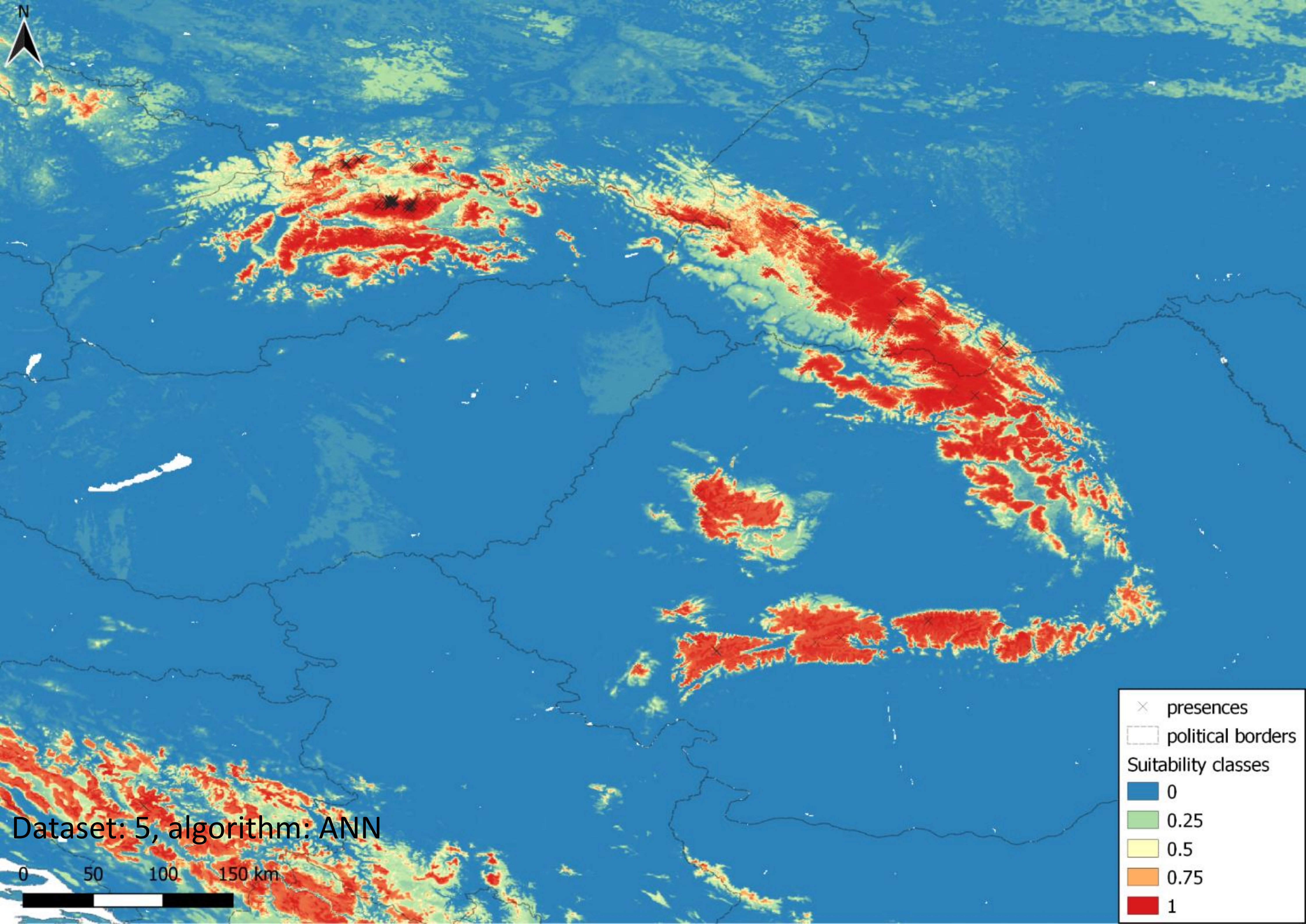
N
Dataset: 5, algorithm: ANN
0
50
100
150 km
presences
political borders
Suitability classes
0
0.25
0.5
0.75
1

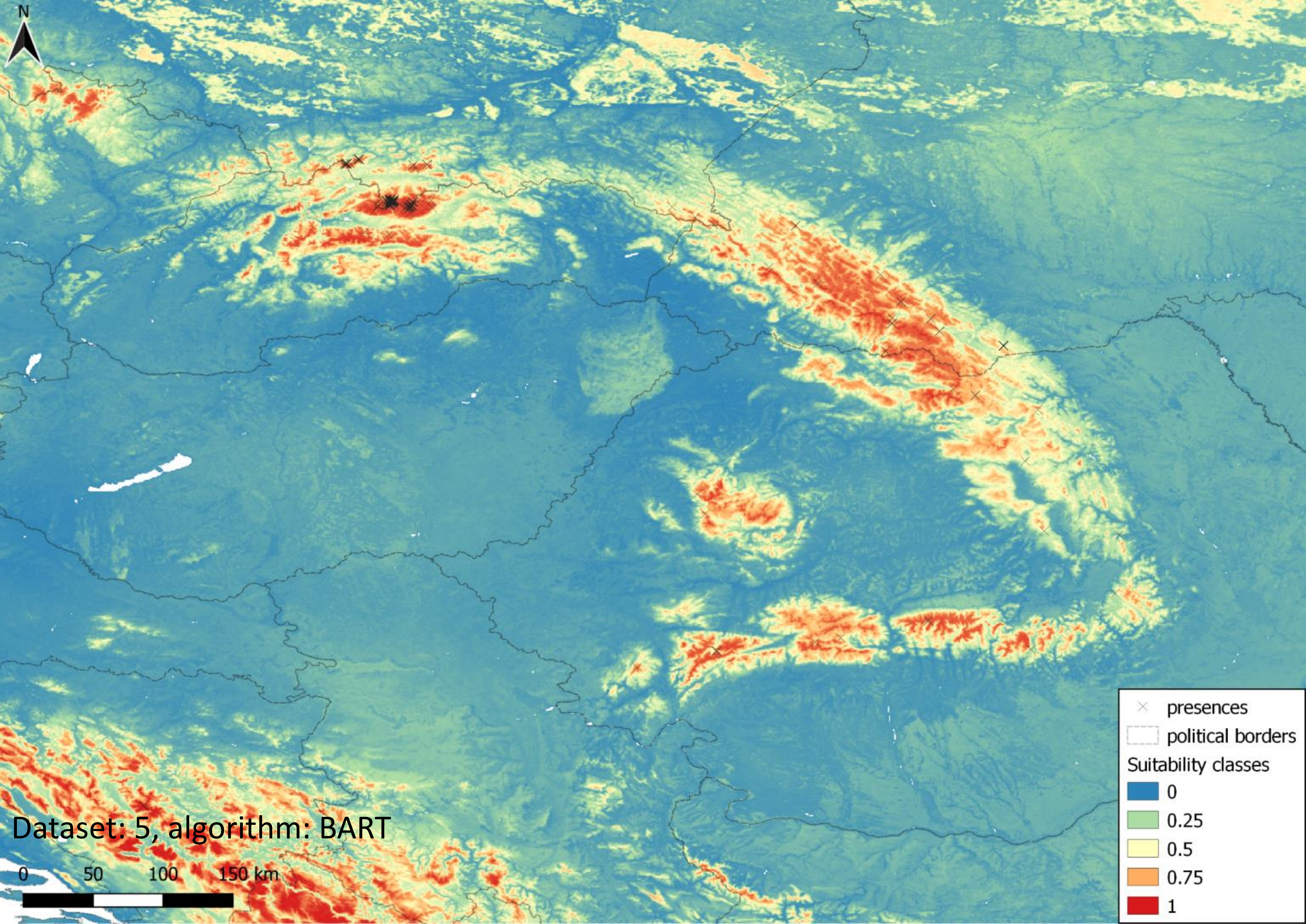
N
presences
political borders
Suitability classes
0
0.25
0.5
0.75
1
Dataset: 5, algorithm: BART
0
50
100
150 km

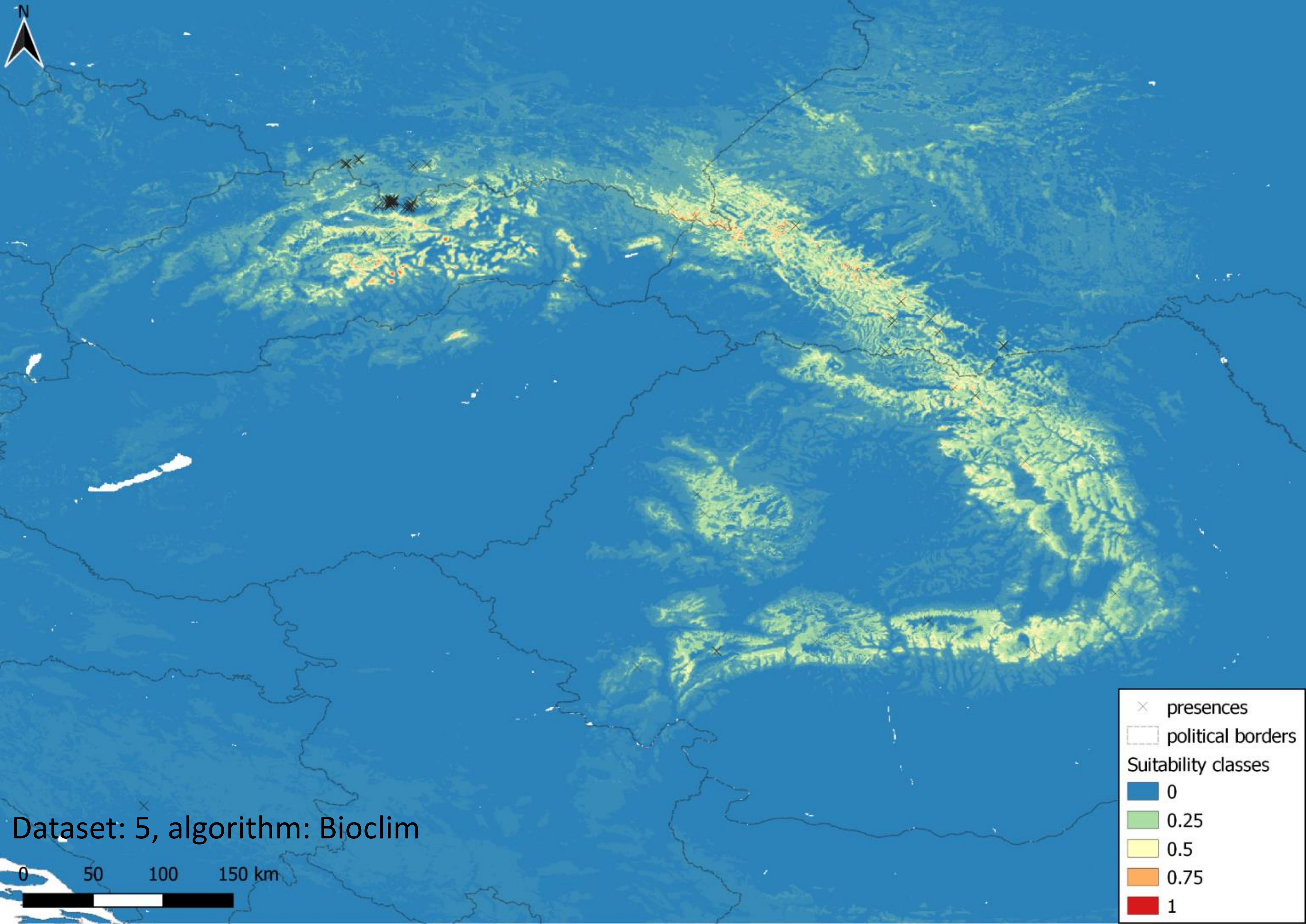
N
Dataset: 5, algorithm: Bioclim
0
50
100
150 km
presences
political borders
Suitability classes
0
0.25
0.5
0.75
1

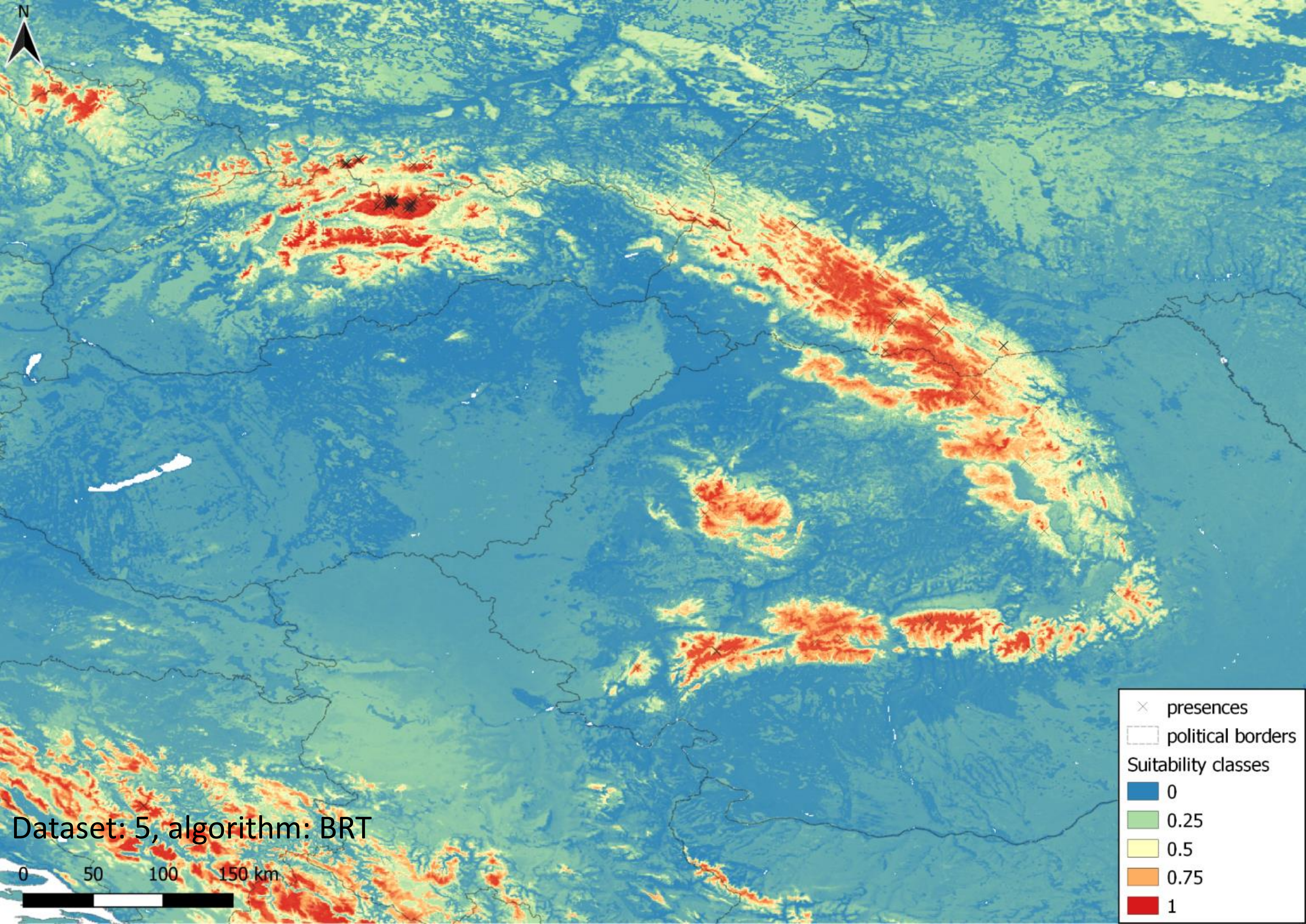
N
presences
political borders
Suitability classes
0
0.25
0.5
0.75
1
Dataset: 5, algorithm: BRT
0
50
100
150 km

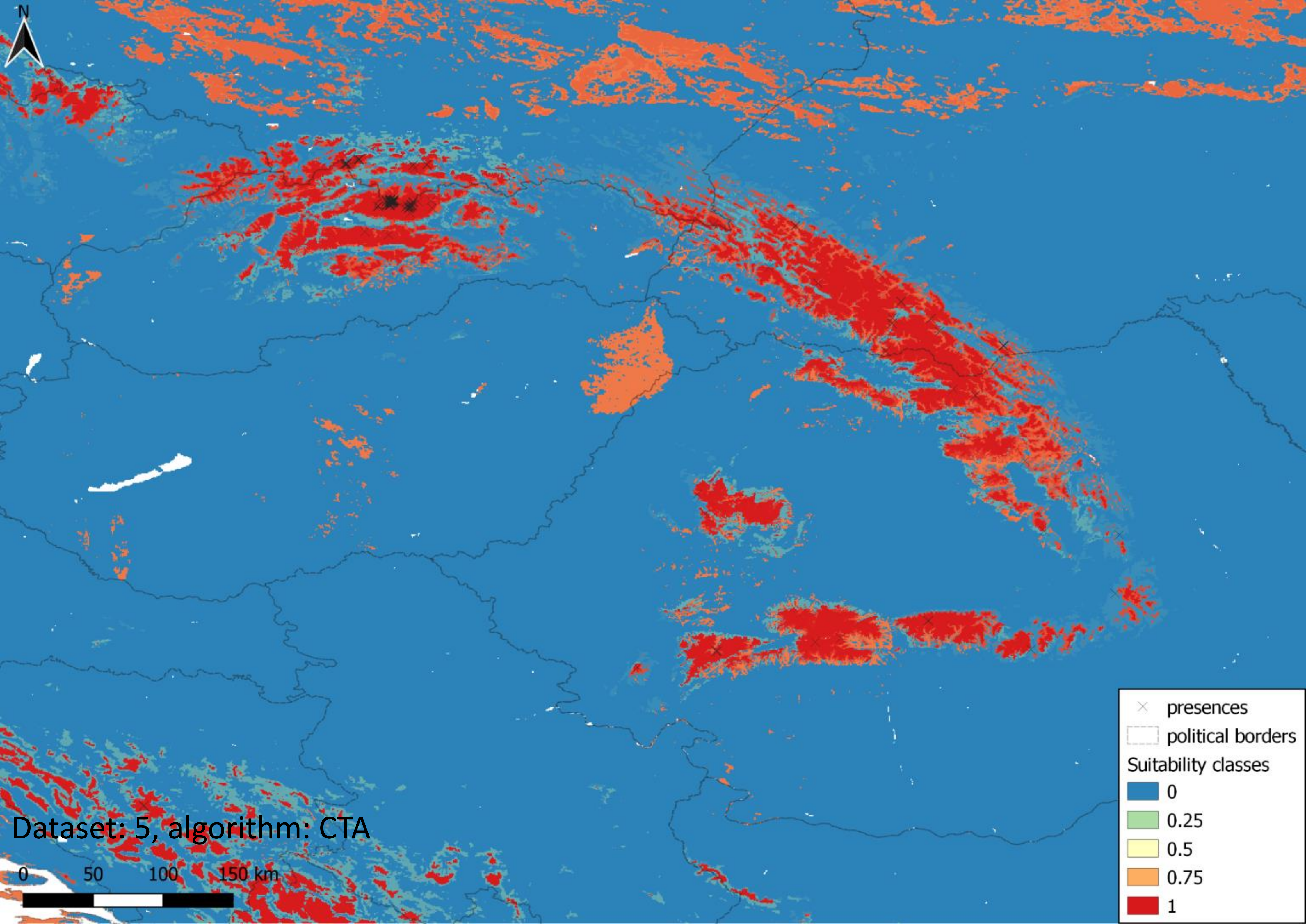
N
presences
political borders
Suitability classes
0
0.25
0.5
0.75
1
Dataset: 5, algorithm: CTA
0
50
100
150 km

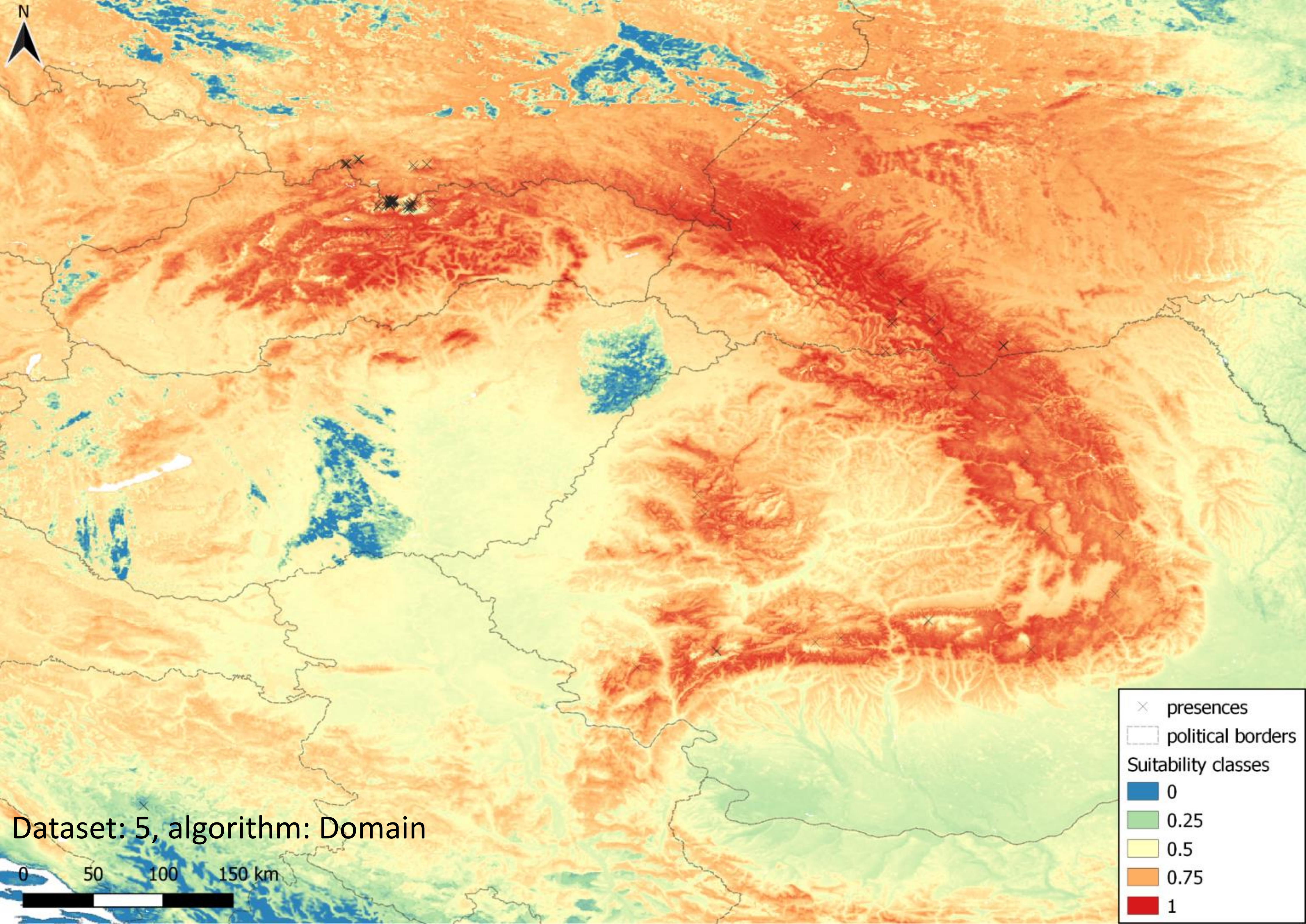
N
presences
political borders
Suitability classes
0
0.25
0.5
0.75
1
Dataset: 5, algorithm: Domain
0
50
100
150 km

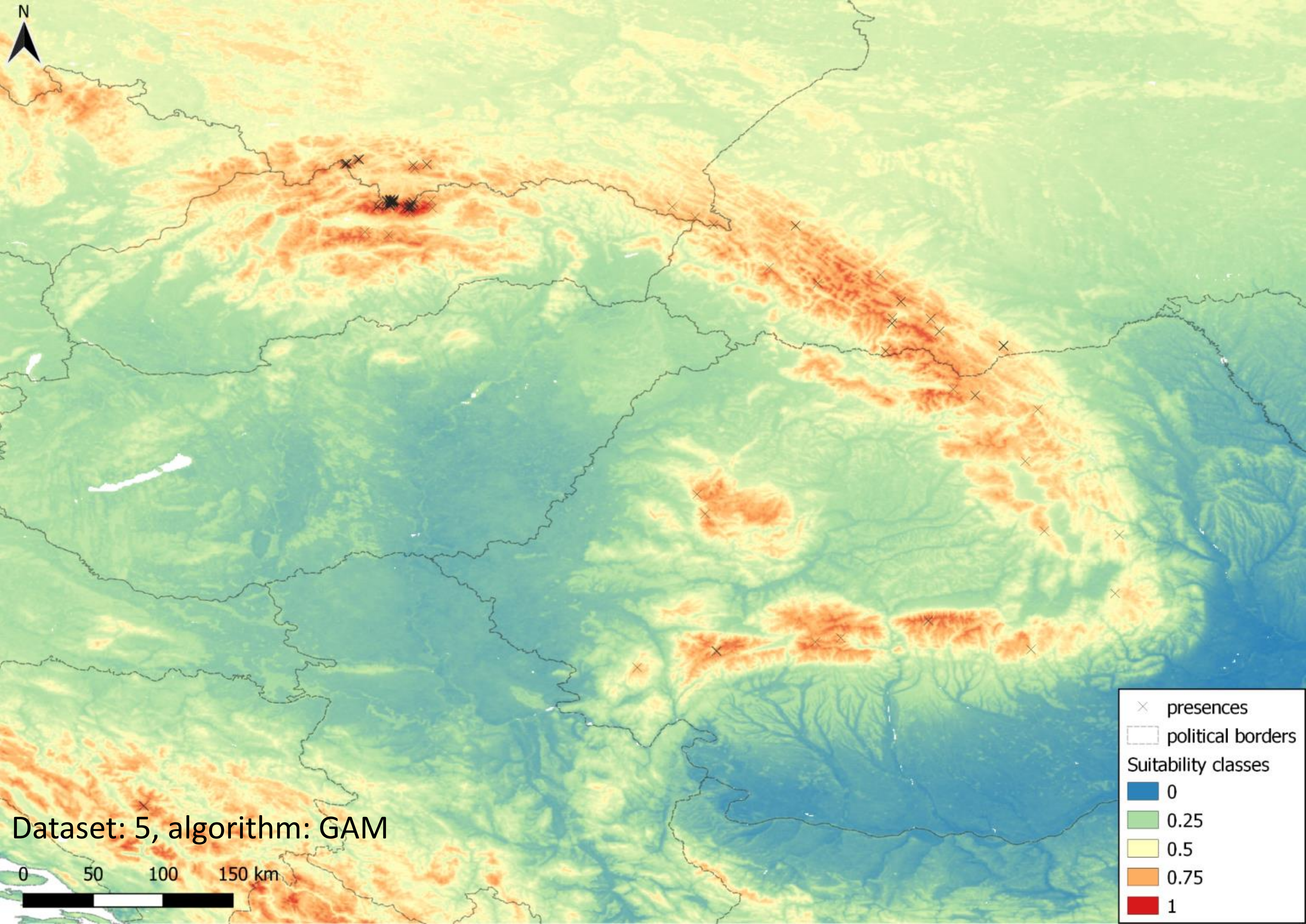
N
presences
political borders
Suitability classes
0
0.25
0.5
0.75
1
Dataset: 5, algorithm: GAM
0
50
100
150 km

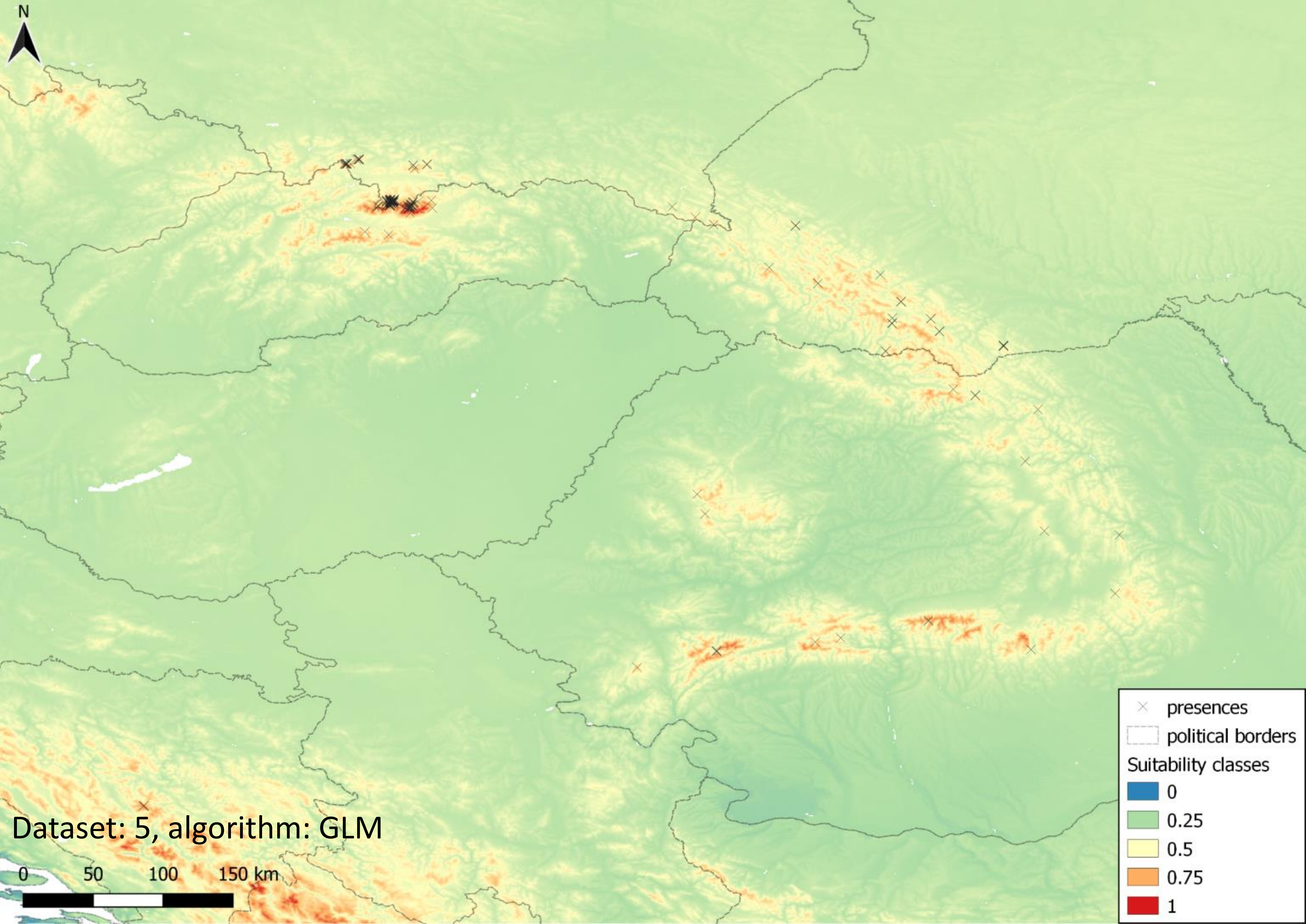
N
Dataset: 5, algorithm: GLM
0
50
100
150 km
presences
political borders
Suitability classes
0
0.25
0.5
0.75
1

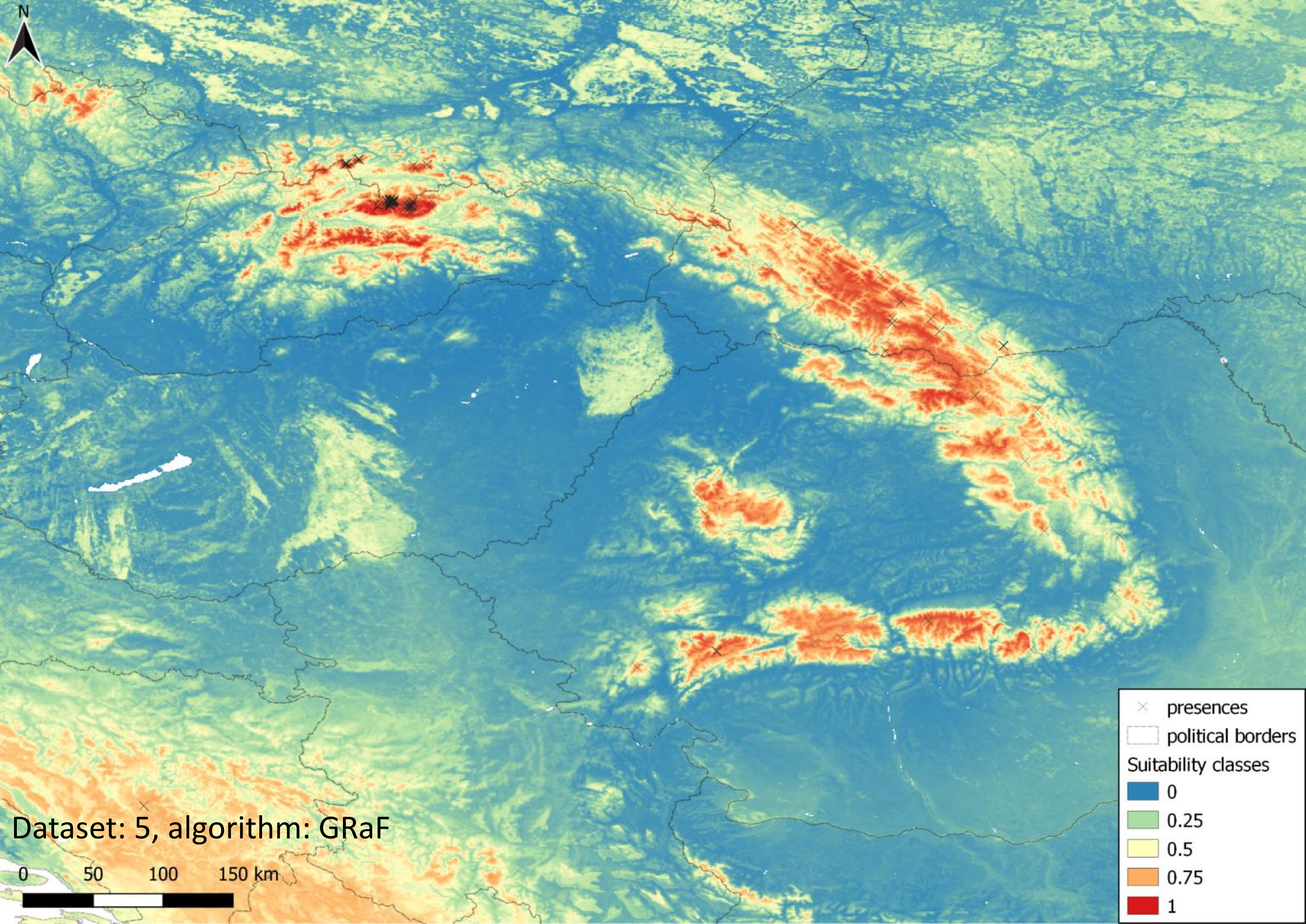

N
presences
political borders
Suitability classes
0
0.25
0.5
0.75
1
Dataset: 5, algorithm: GRaF
0
50
100
150 km

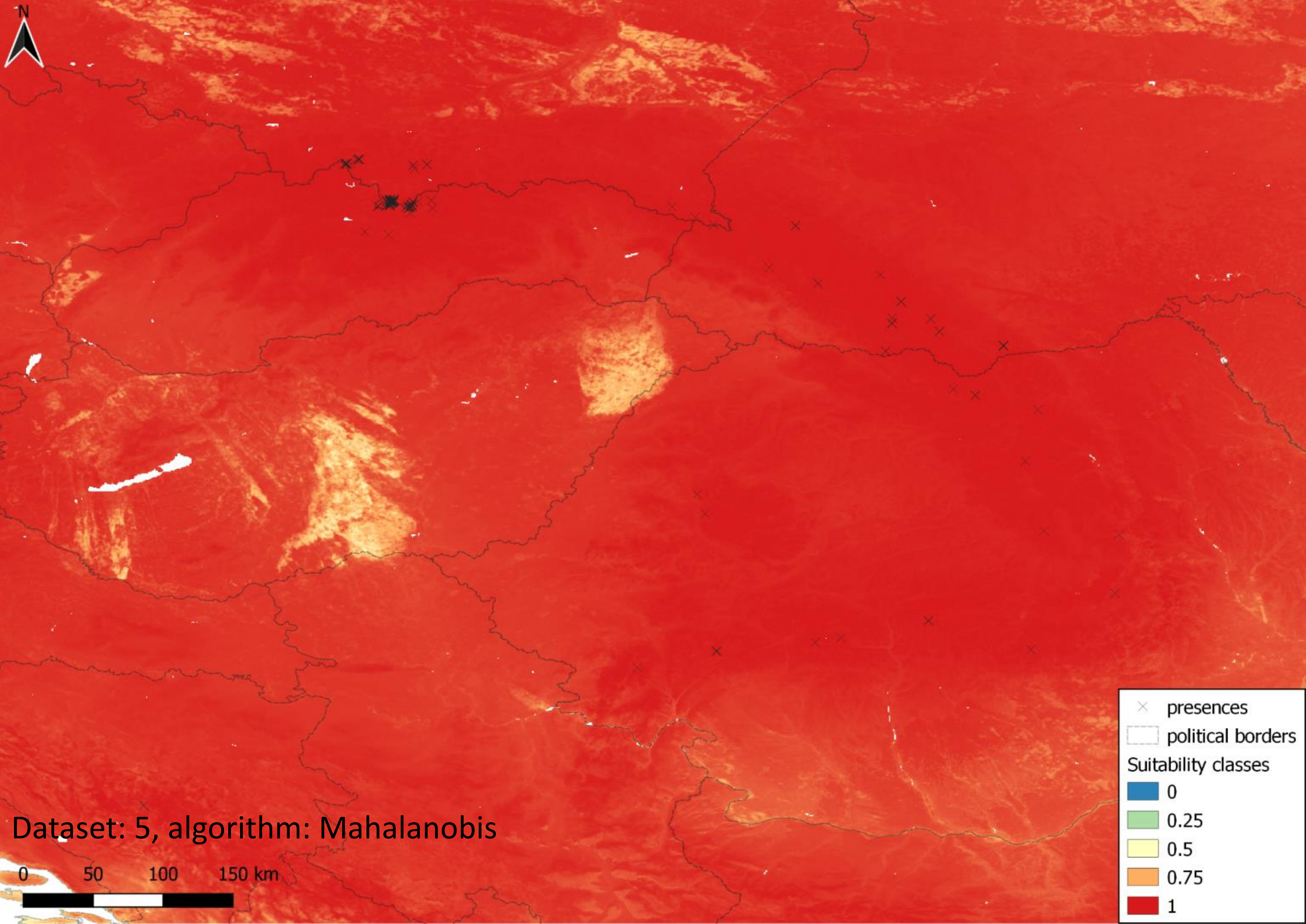
N
presences
political borders
Suitability classes
0
0.25
0.5
0.75
1
Dataset: 5, algorithm: Mahalanobis
0
50
100
150 km

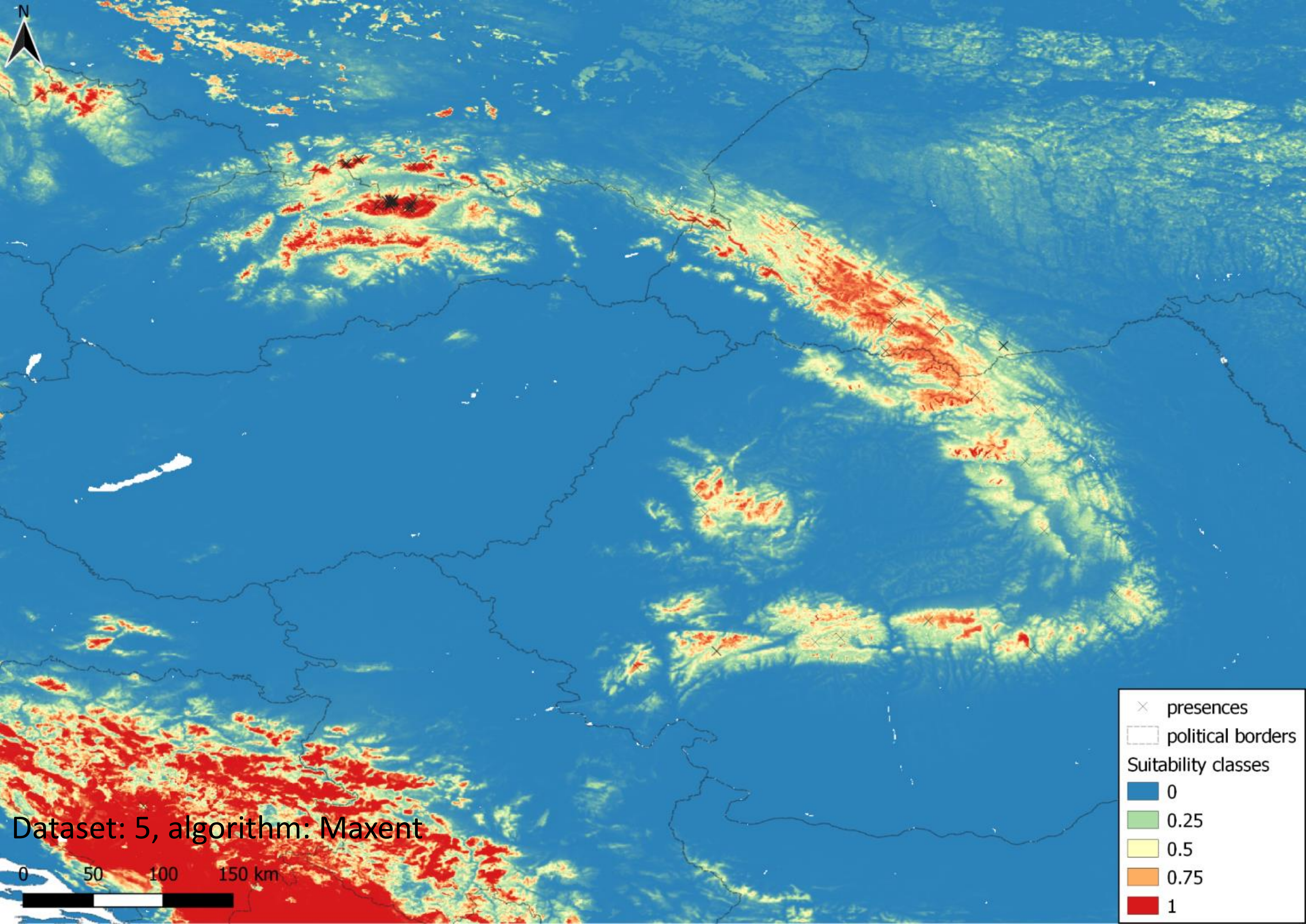
N
presences
political borders
Suitability classes
0
0.25
0.5
0.75
1
Dataset: 5, algorithm: Maxent
0
50
100
150 km

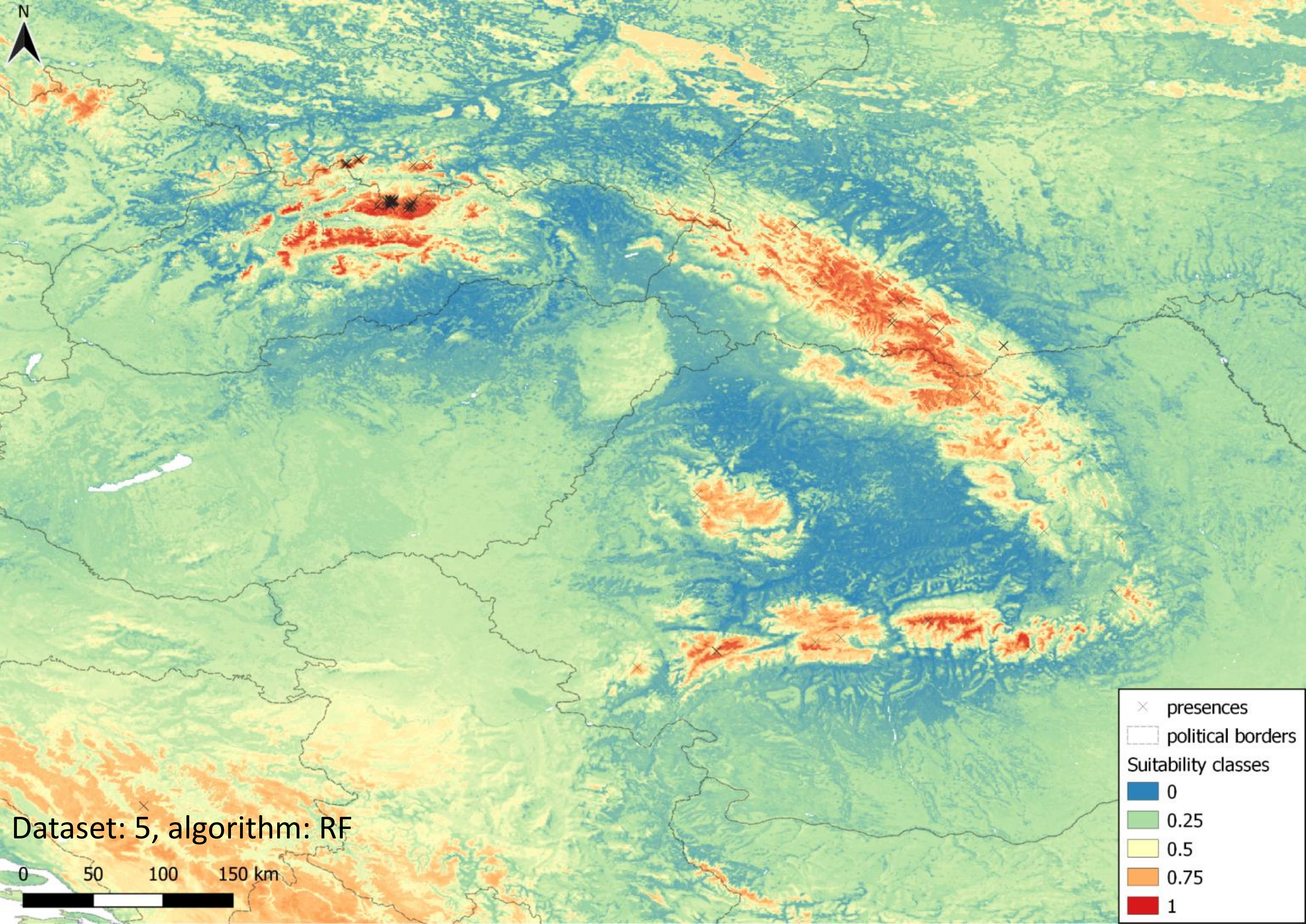
N
Dataset: 5, algorithm: RF
0
50
100
150 km
presences
political borders
Suitability classes
0
0.25
0.5
0.75
1

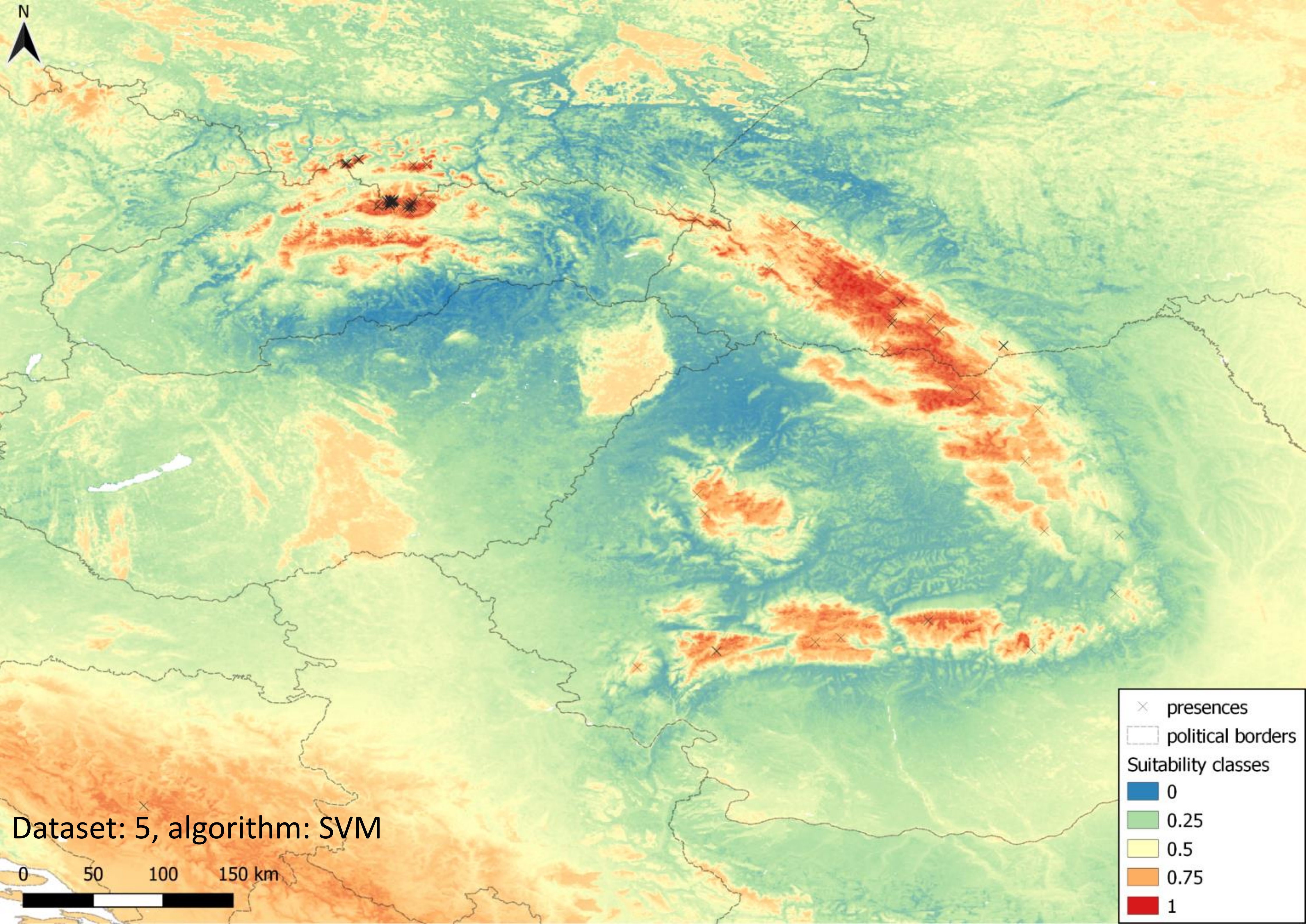
N
Dataset: 5, algorithm: SVM
0
50
100
150 km
presences
political borders
Suitability classes
0
0.25
0.5
0.75
1

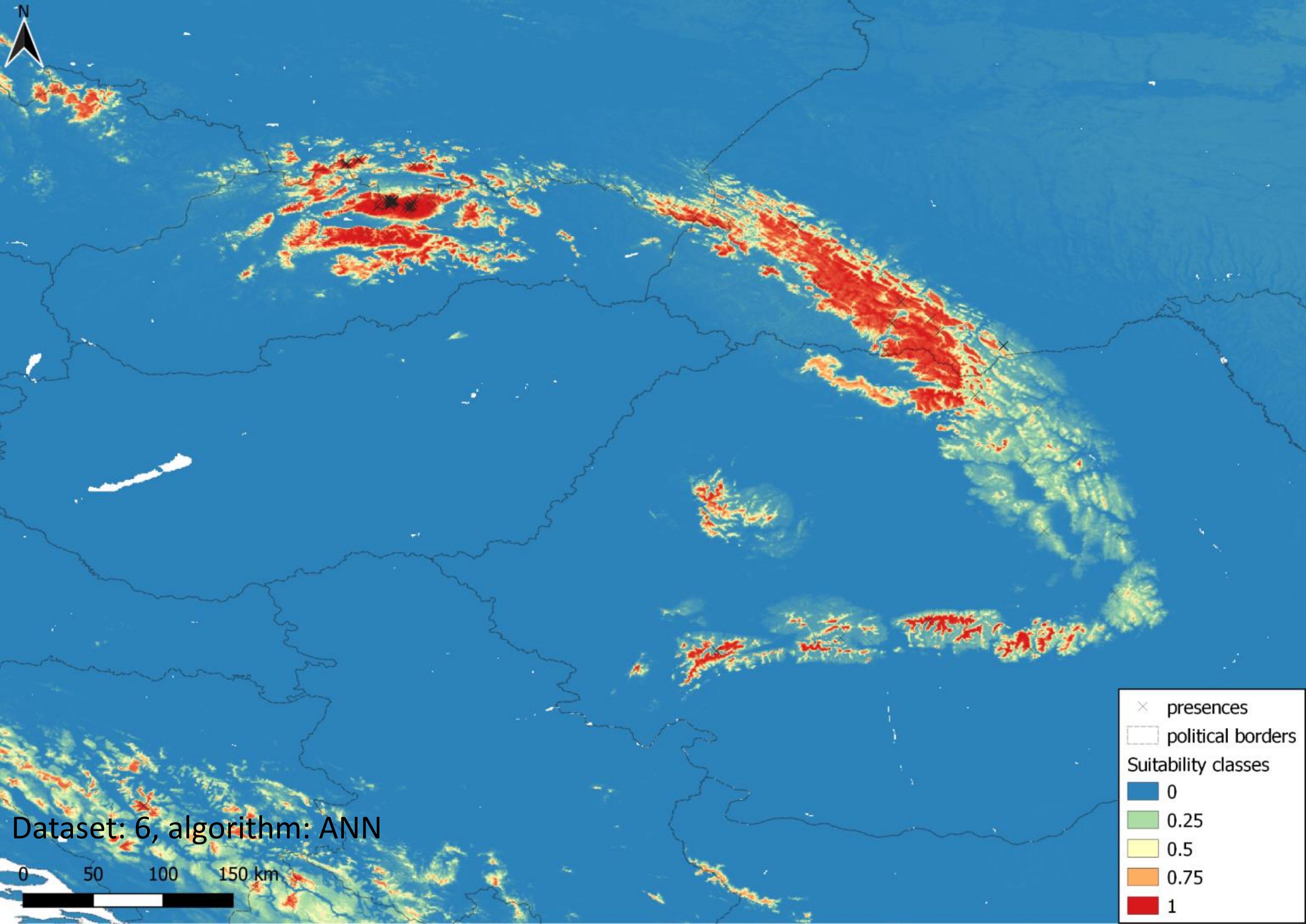
N
Dataset: 6, algorithm: ANN
0
50
100
150 km
presences
political borders
Suitability classes
0
0.25
0.5
0.75
1

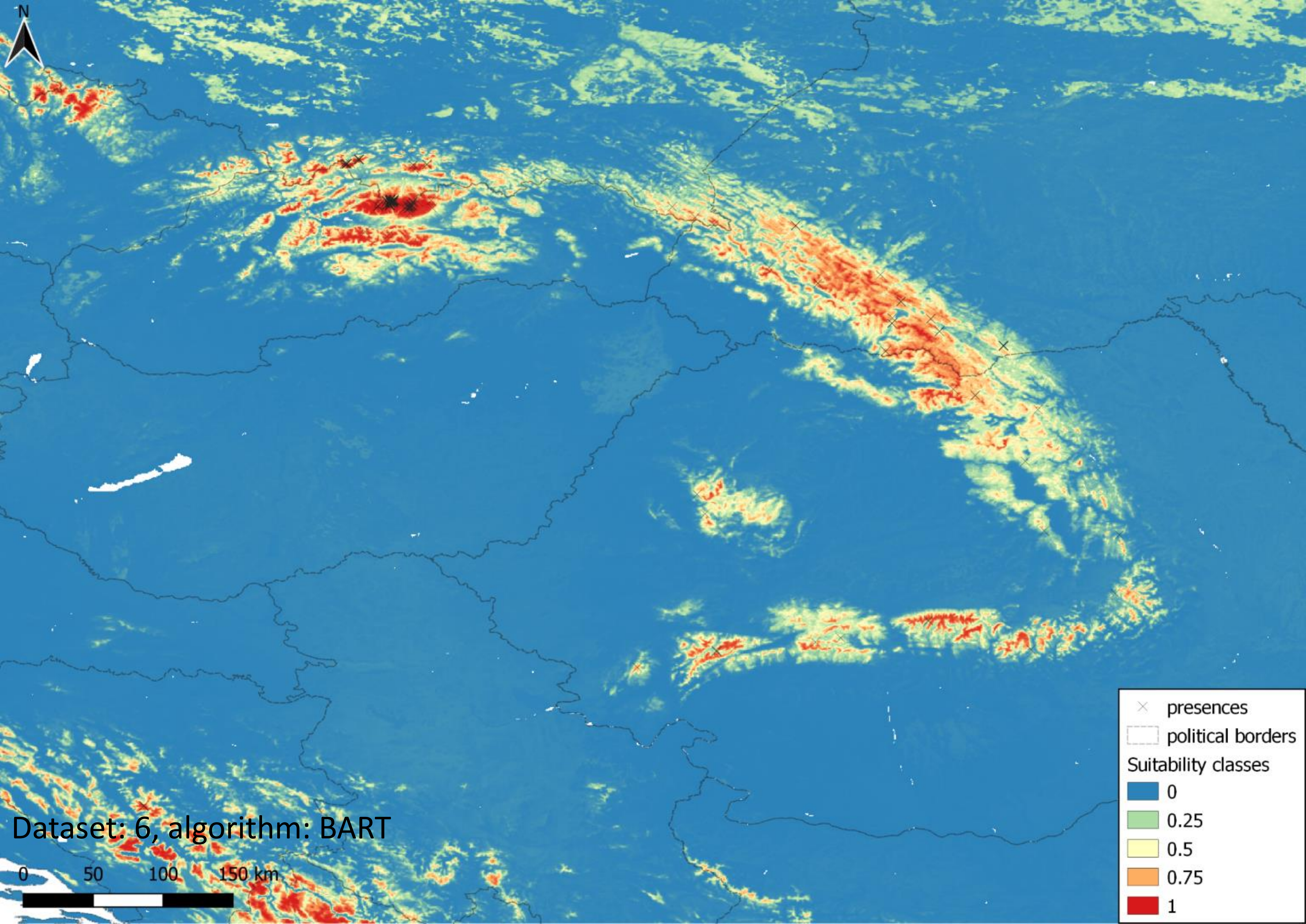
N
Dataset: 6, algorithm: BART
0
50
100
150 km
presences
political borders
Suitability classes
0
0.25
0.5
0.75
1

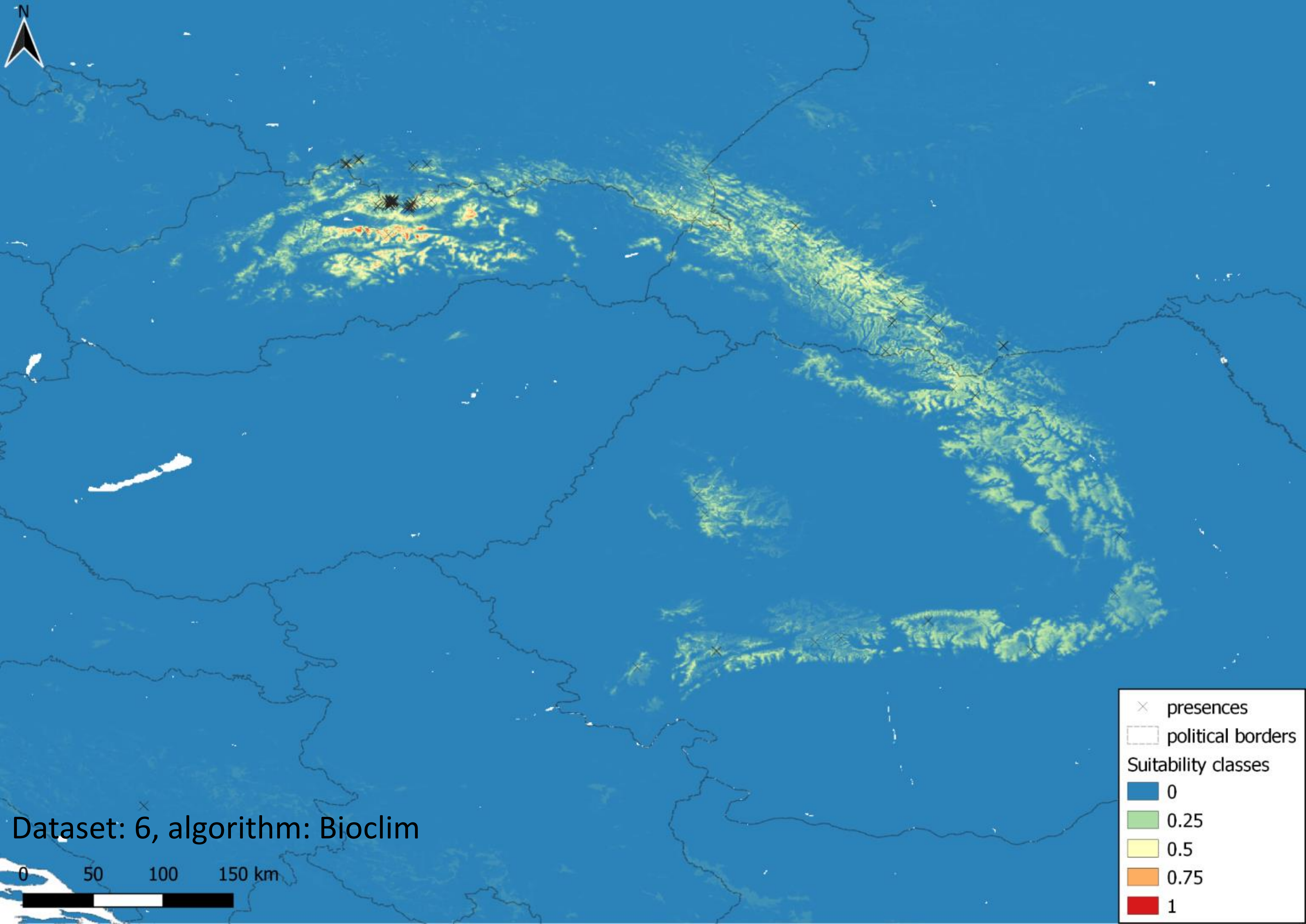
N
Dataset: 6, algorithm: Bioclim
0
50
100
150 km
presences
political borders
Suitability classes
0
0.25
0.5
0.75
1

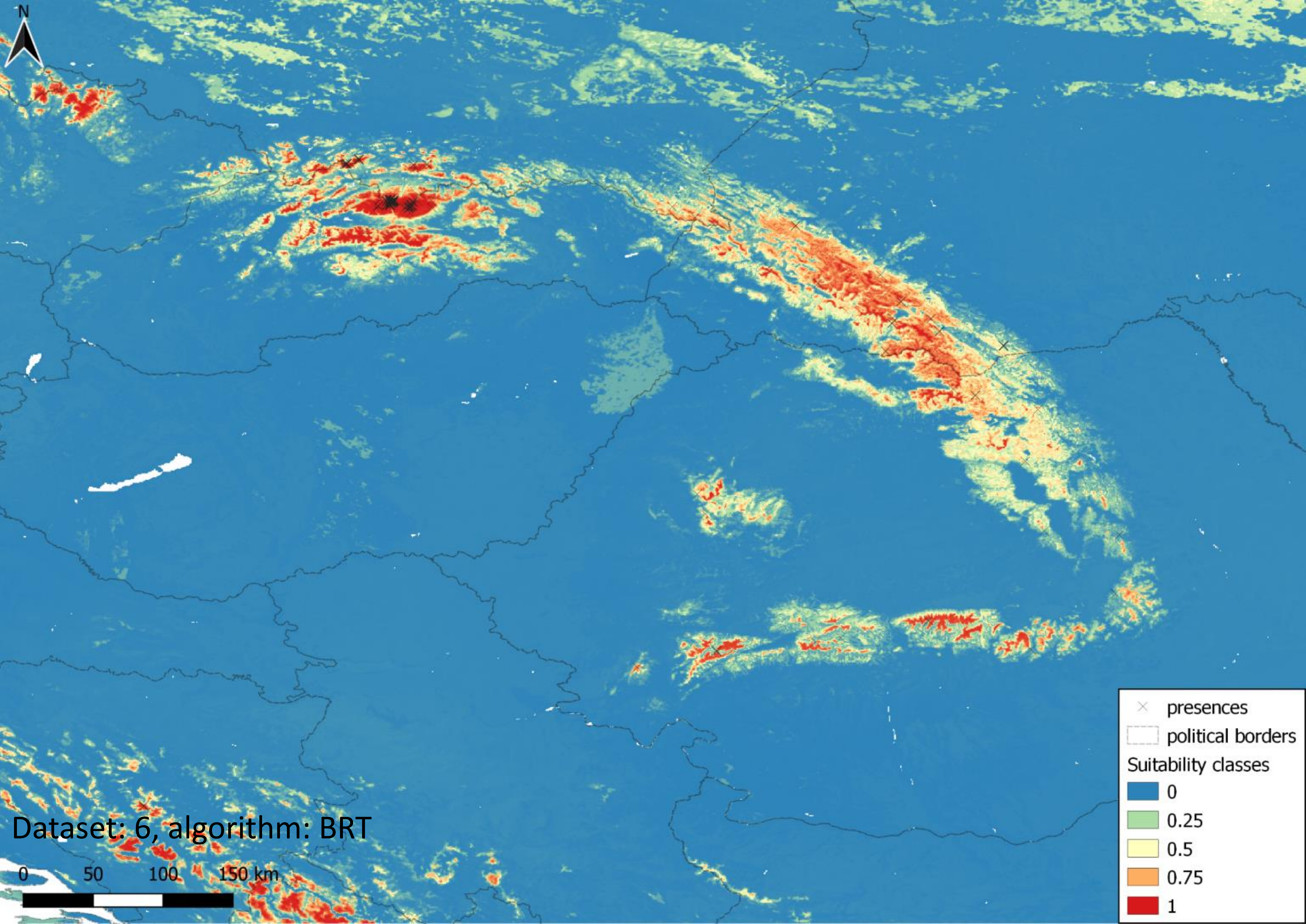
N
Dataset: 6, algorithm: BRT
0
50
100
150 km
presences
political borders
Suitability classes
0
0.25
0.5
0.75
1

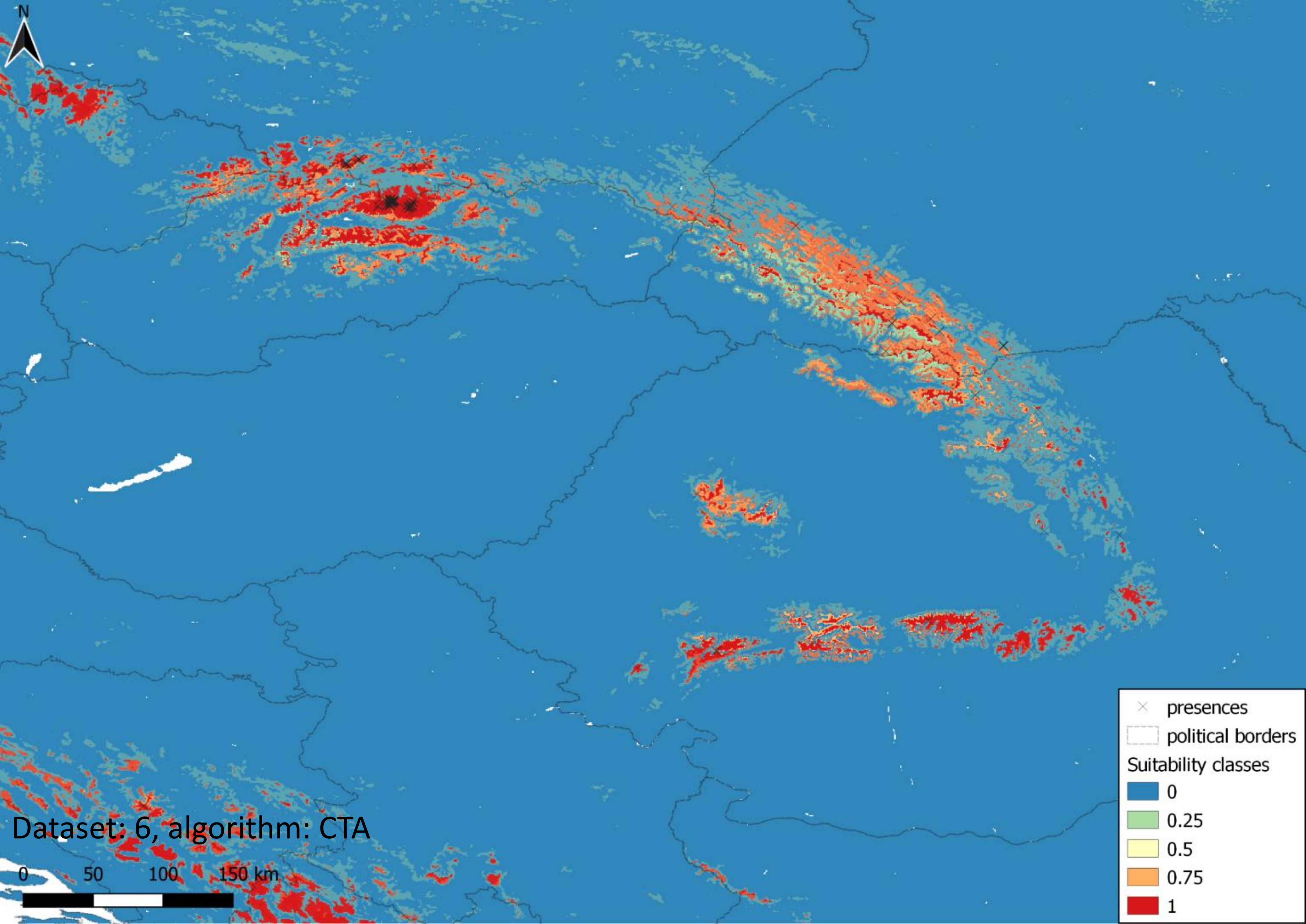
N
presences
political borders
Suitability classes
0
0.25
0.5
0.75
1
Dataset: 6, algorithm: CTA
0
50
100
150 km

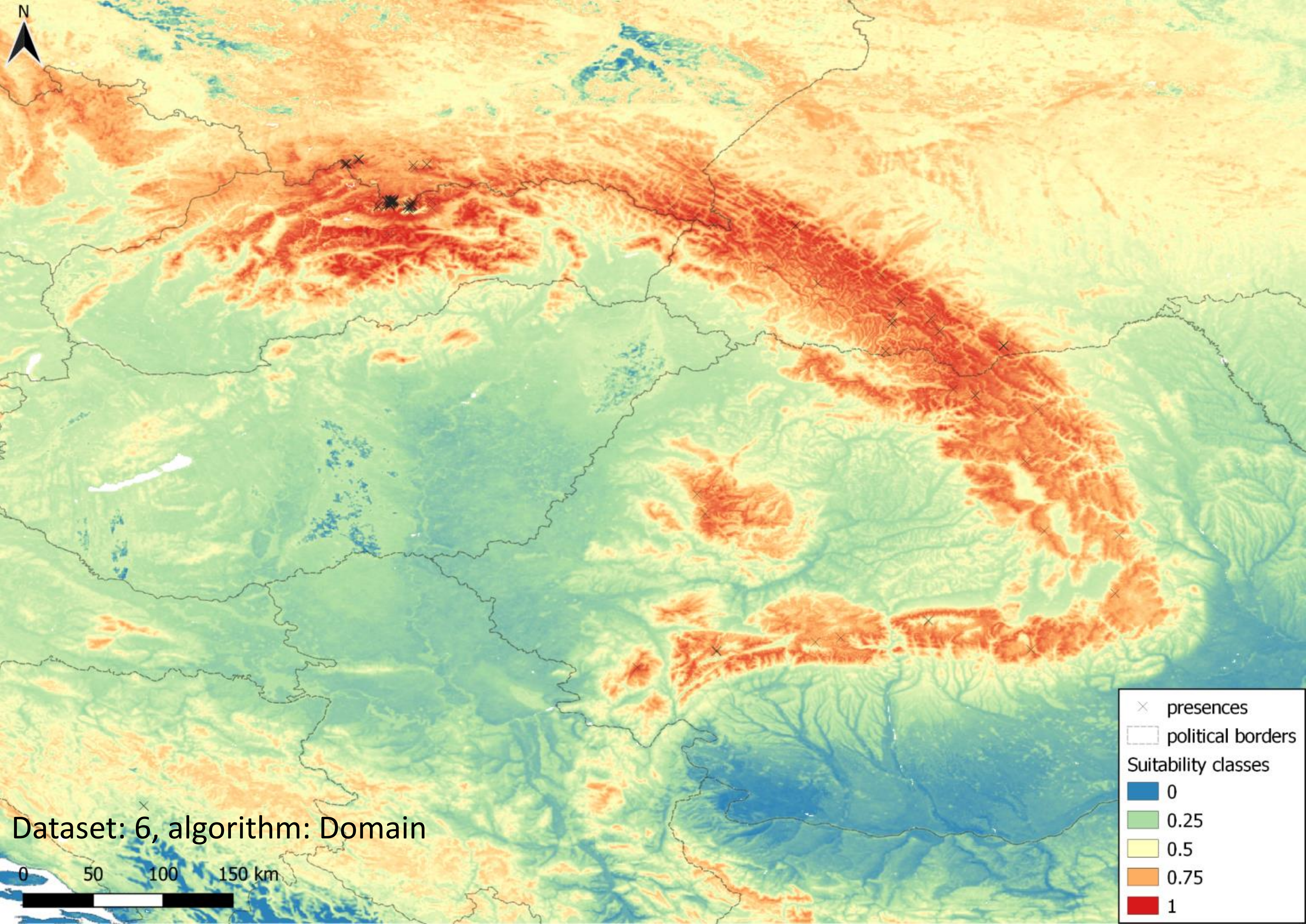
N
Dataset: 6, algorithm: Domain
0
50
100
150 km
presences
political borders
Suitability classes
0
0.25
0.5
0.75
1

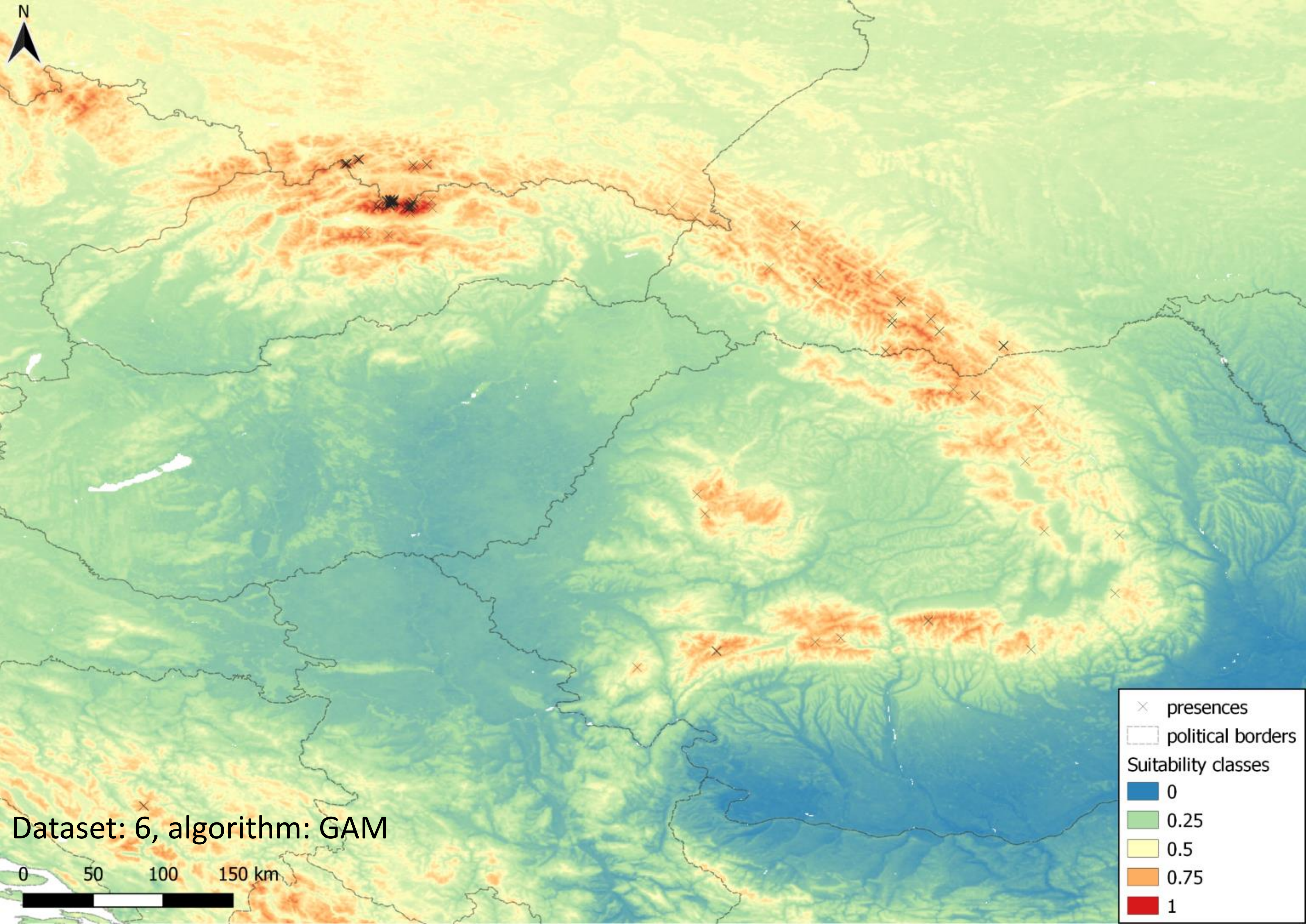
N
Dataset: 6, algorithm: GAM
0
50
100
150 km
presences
political borders
Suitability classes
0
0.25
0.5
0.75
1

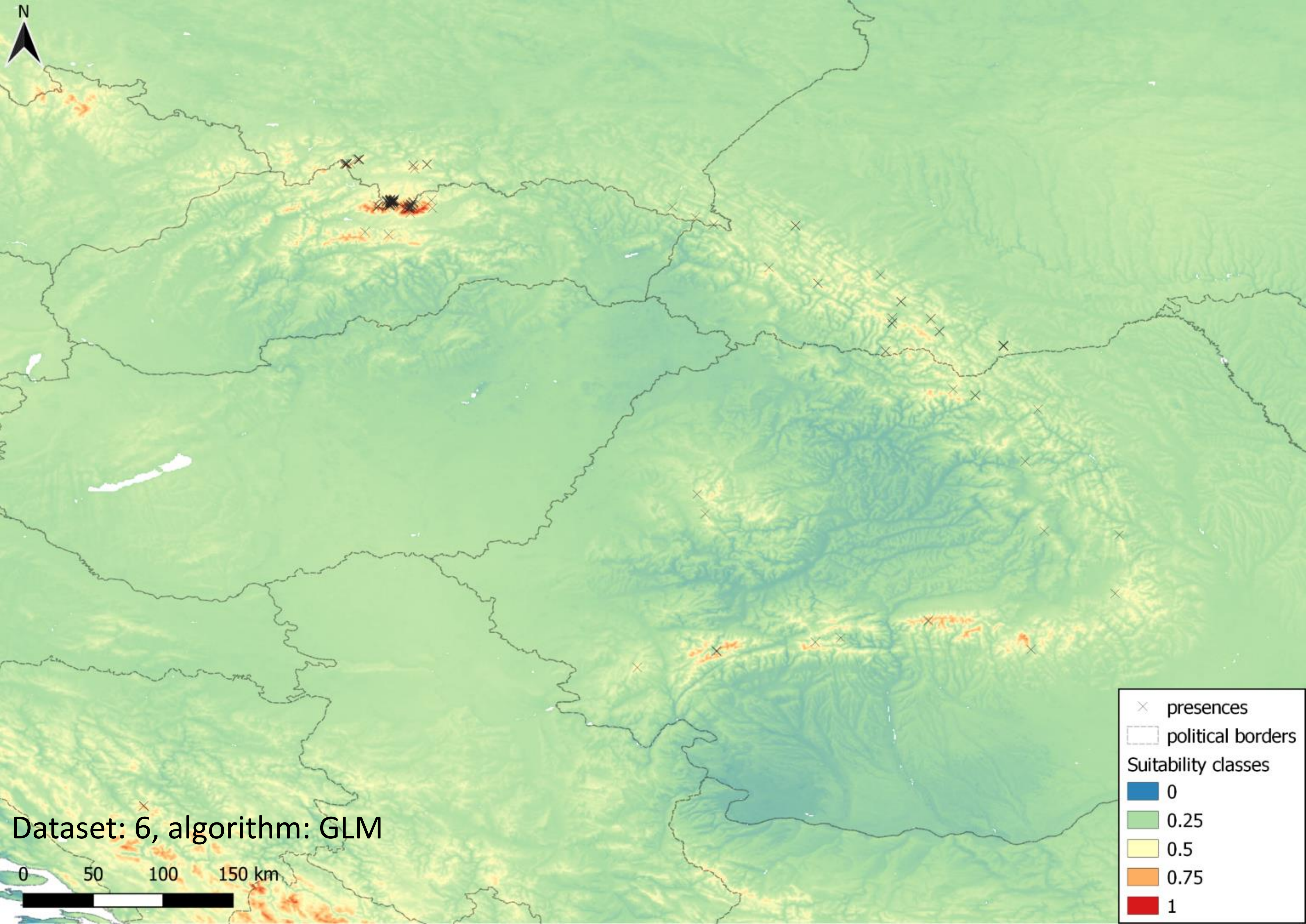
N
presences
political borders
Suitability classes
0
0.25
0.5
0.75
1
Dataset: 6, algorithm: GLM
0
50
100
150 km

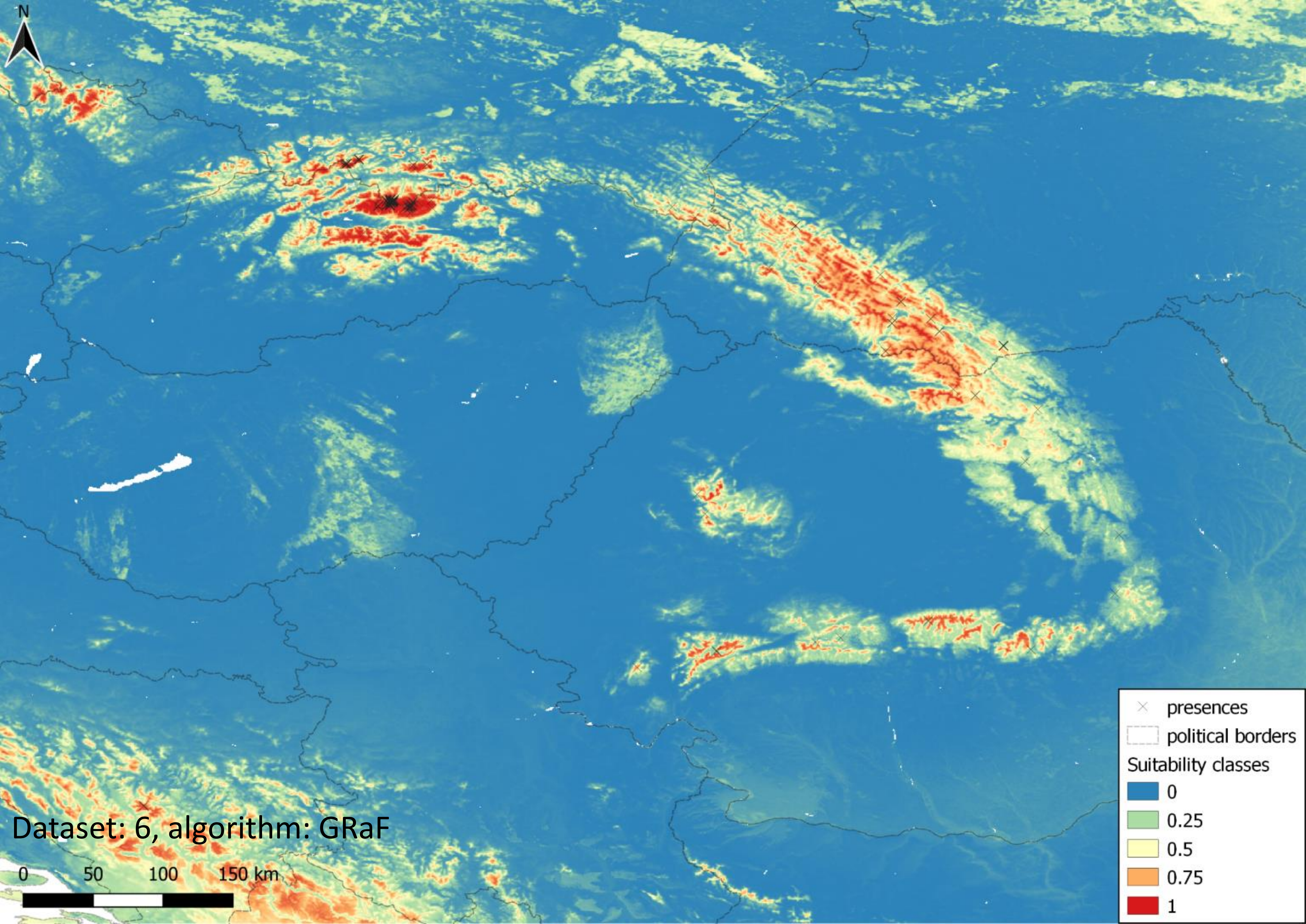
N
presences
political borders
Suitability classes
0
0.25
0.5
0.75
1
Dataset: 6, algorithm: GRaF
0
50
100
150 km

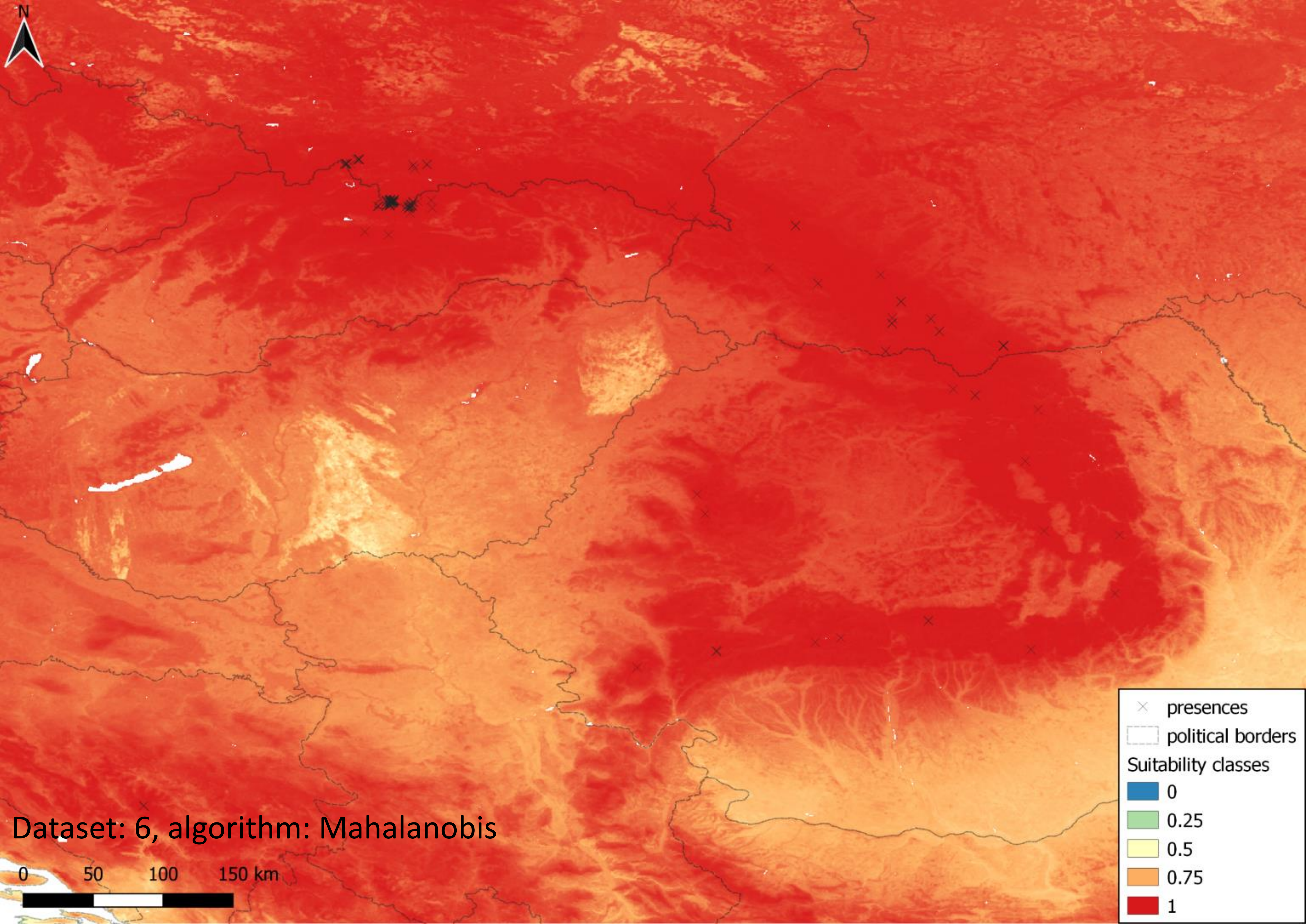
N
presences
political borders
Suitability classes
0
0.25
0.5
0.75
1
Dataset: 6, algorithm: Mahalanobis
0
50
100
150 km

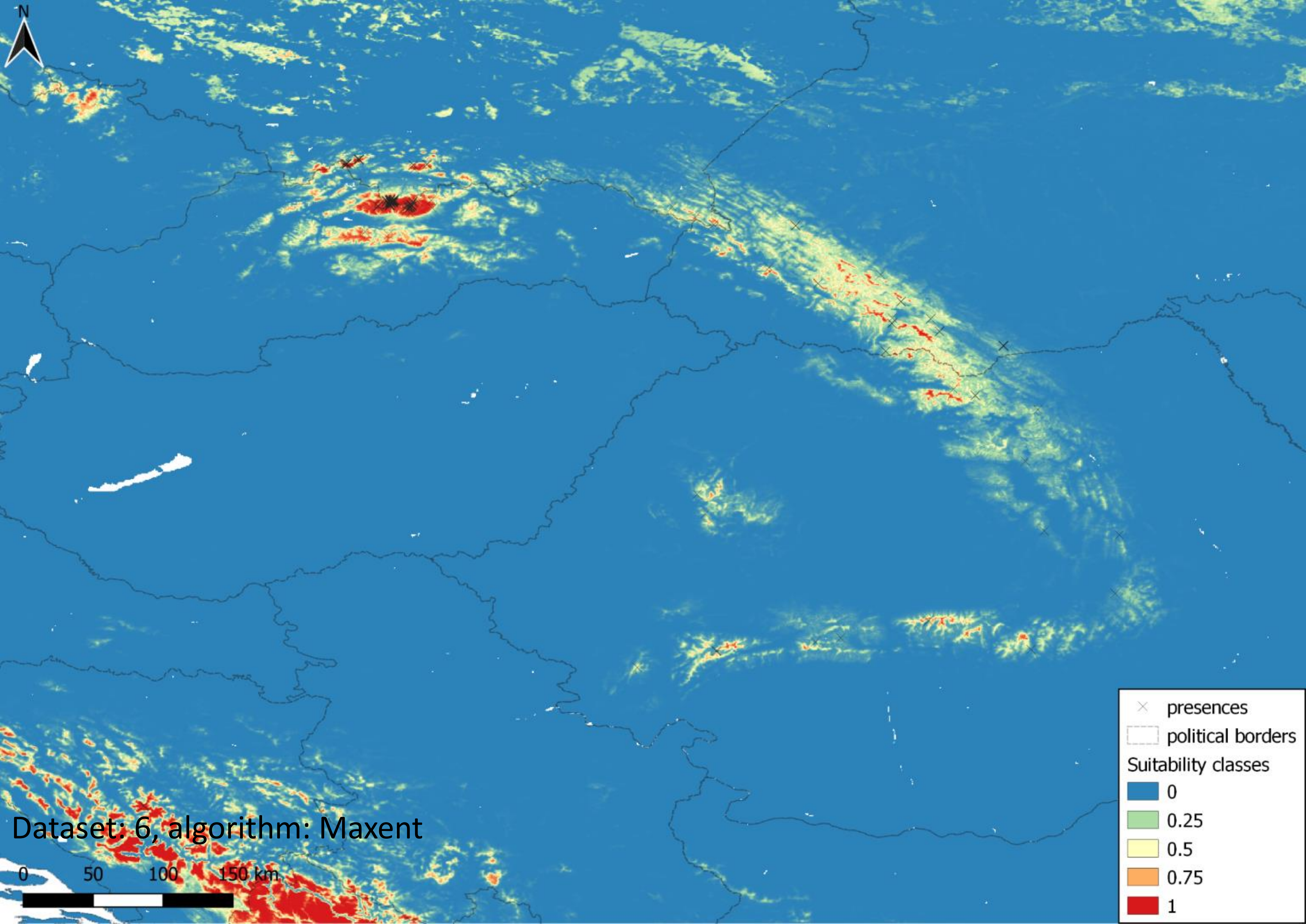
N
Dataset: 6, algorithm: Maxent
0 50 100 150 km
presences
political borders
Suitability classes
0
0.25
0.5
0.75
1

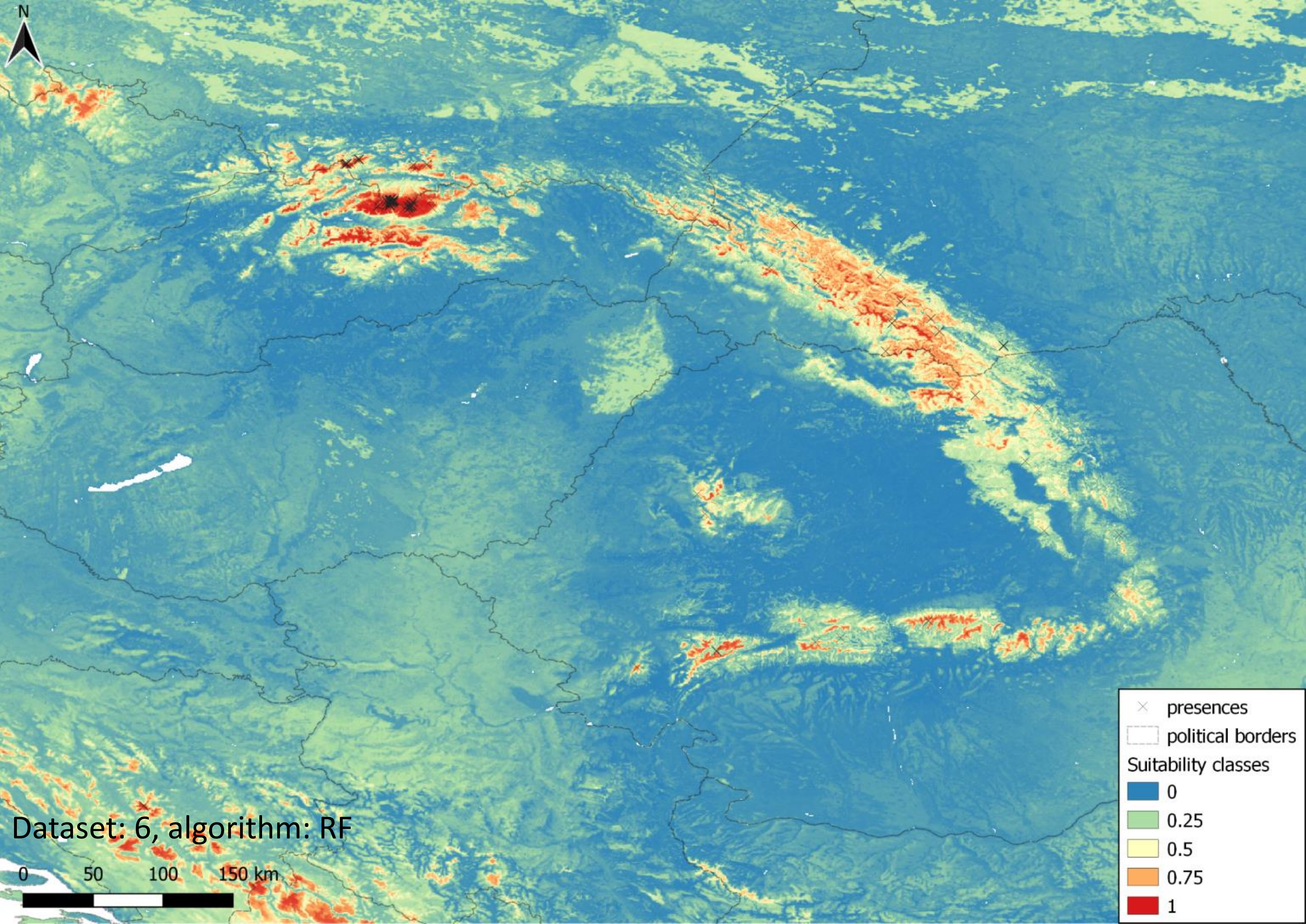
N
presences
political borders
Suitability classes
0
0.25
0.5
0.75
1
Dataset: 6, algorithm: RF
0
50
100
150 km

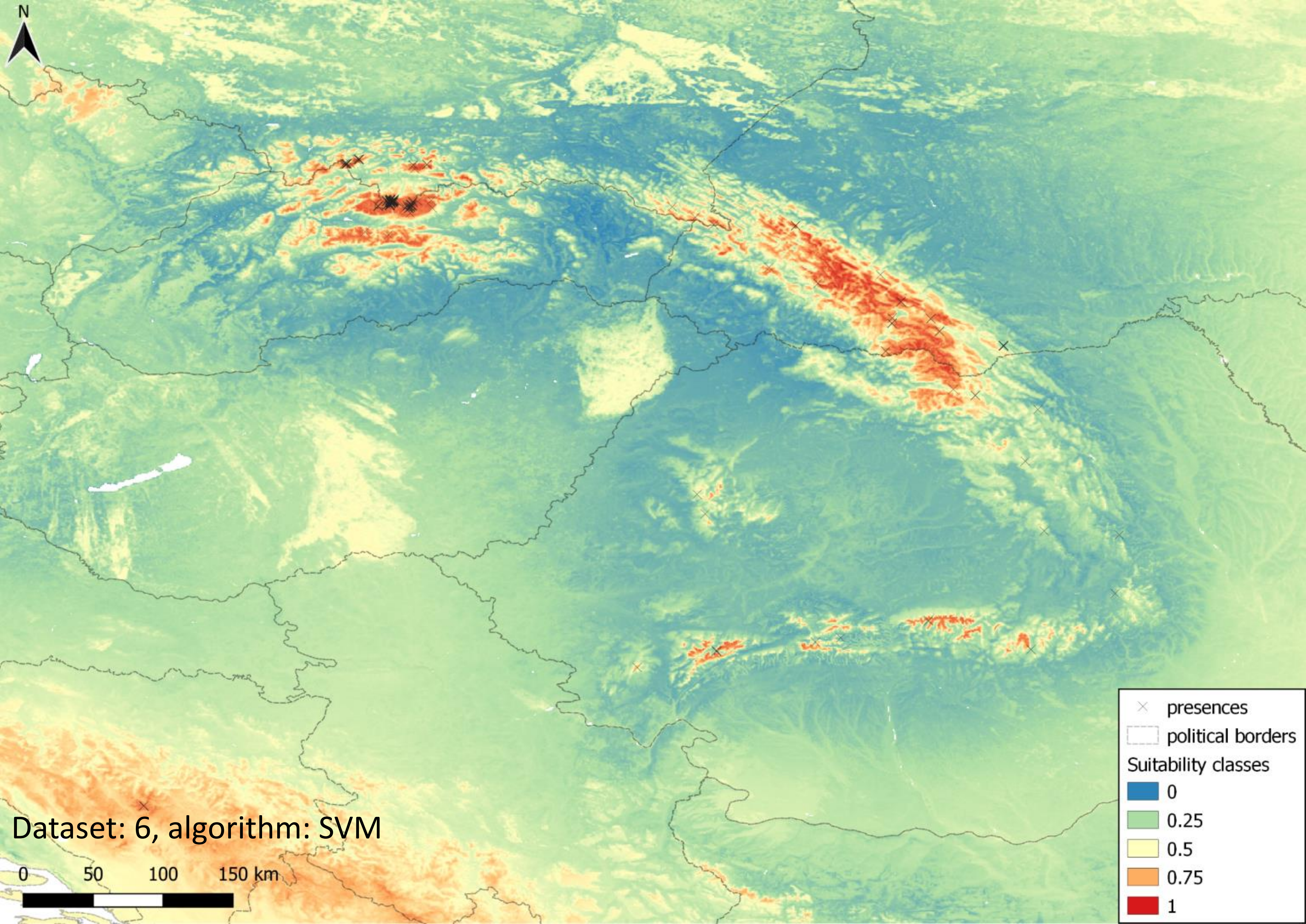
N
Dataset: 6, algorithm: SVM
0
50
100
150 km
presences
political borders
Suitability classes
0
0.25
0.5
0.75
1

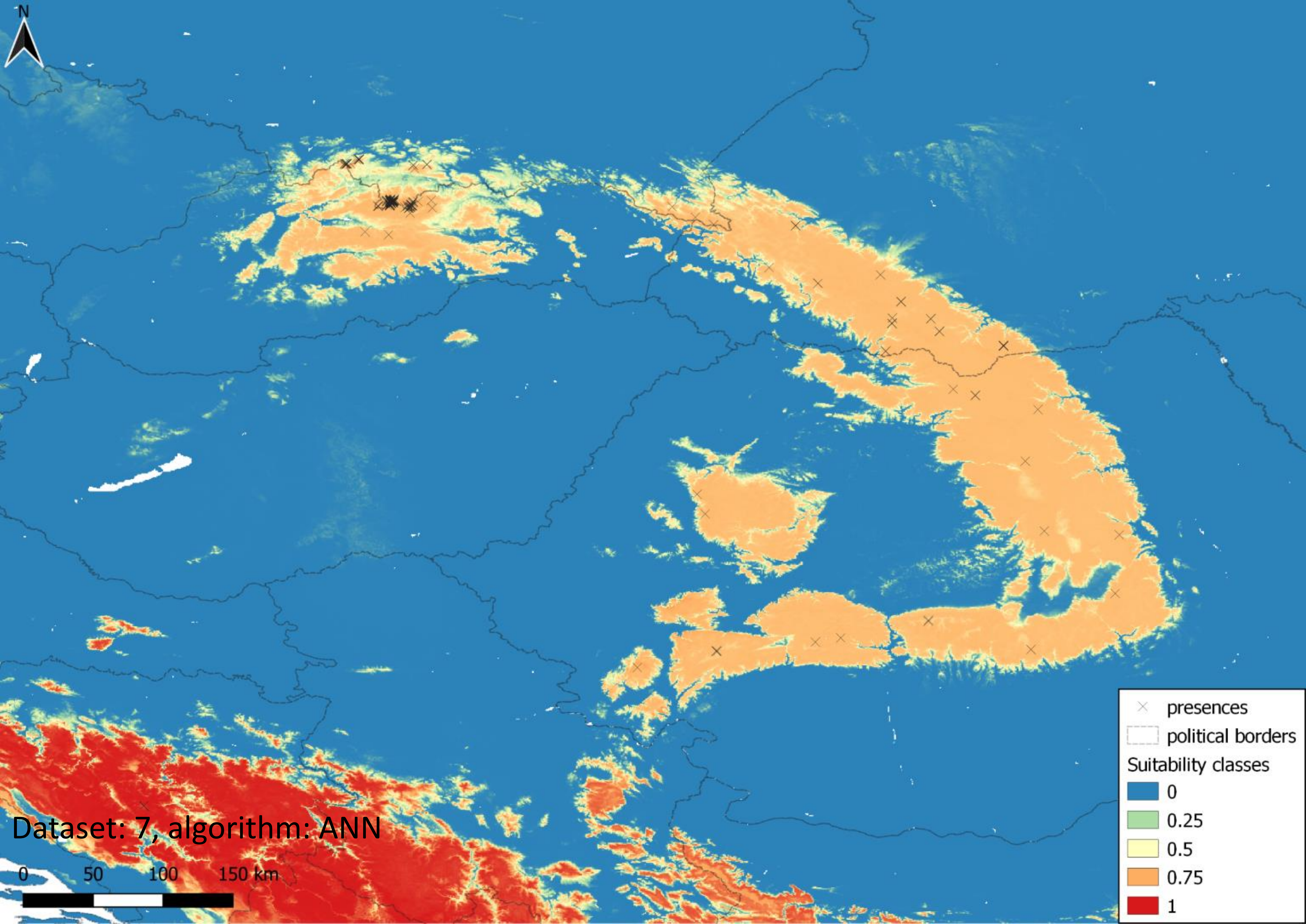
N
Dataset: 7, algorithm: ANN
0
50
100
150 km
presences
political borders
Suitability classes
0
0.25
0.5
0.75
1

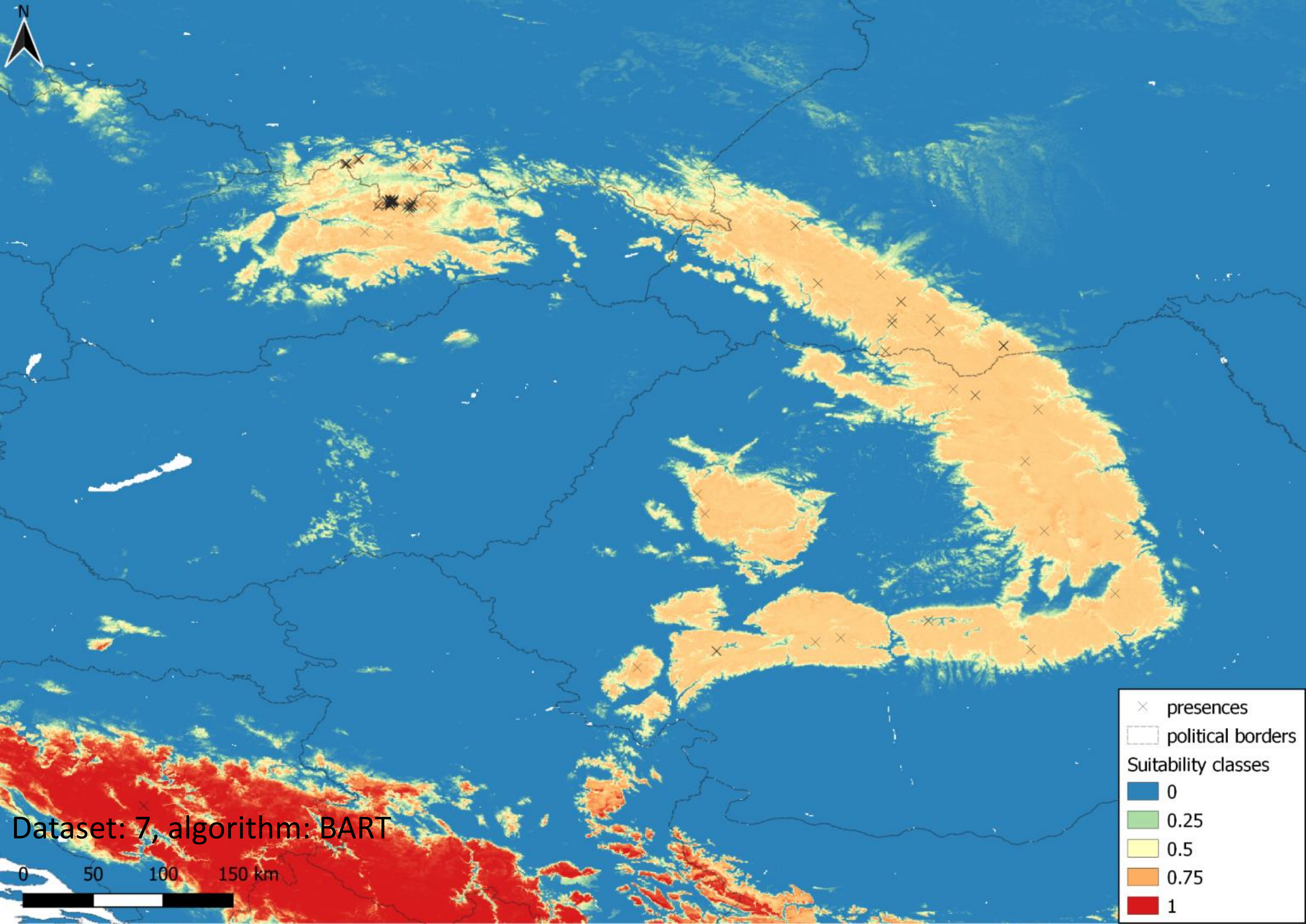
N
Dataset: 7, algorithm: BART
0
50
100
150 km
presences
political borders
Suitability classes
0
0.25
0.5
0.75
1

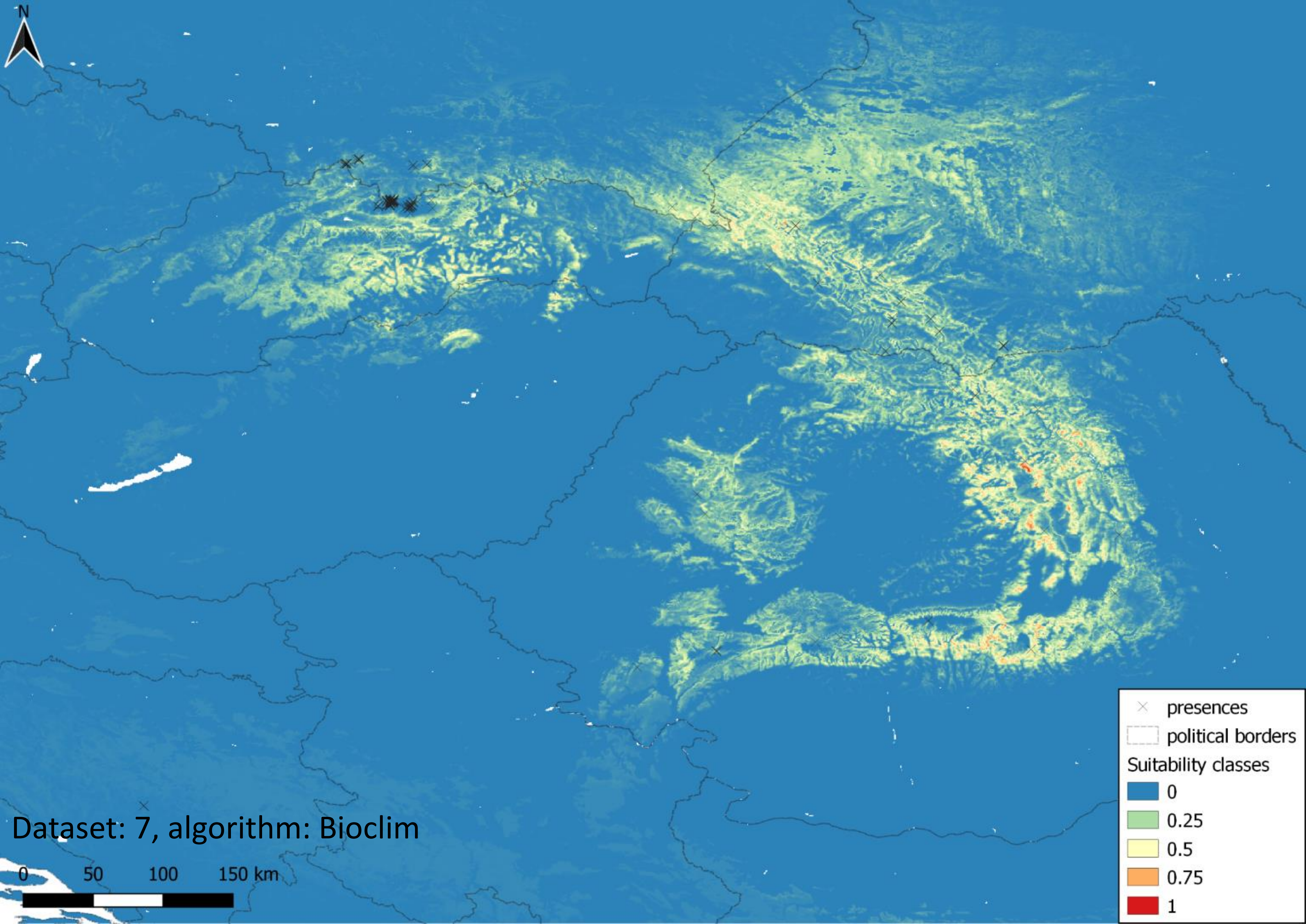
N
presences
political borders
Suitability classes
0
0.25
0.5
0.75
1
Dataset: 7, algorithm: Bioclim
0
50
100
150 km

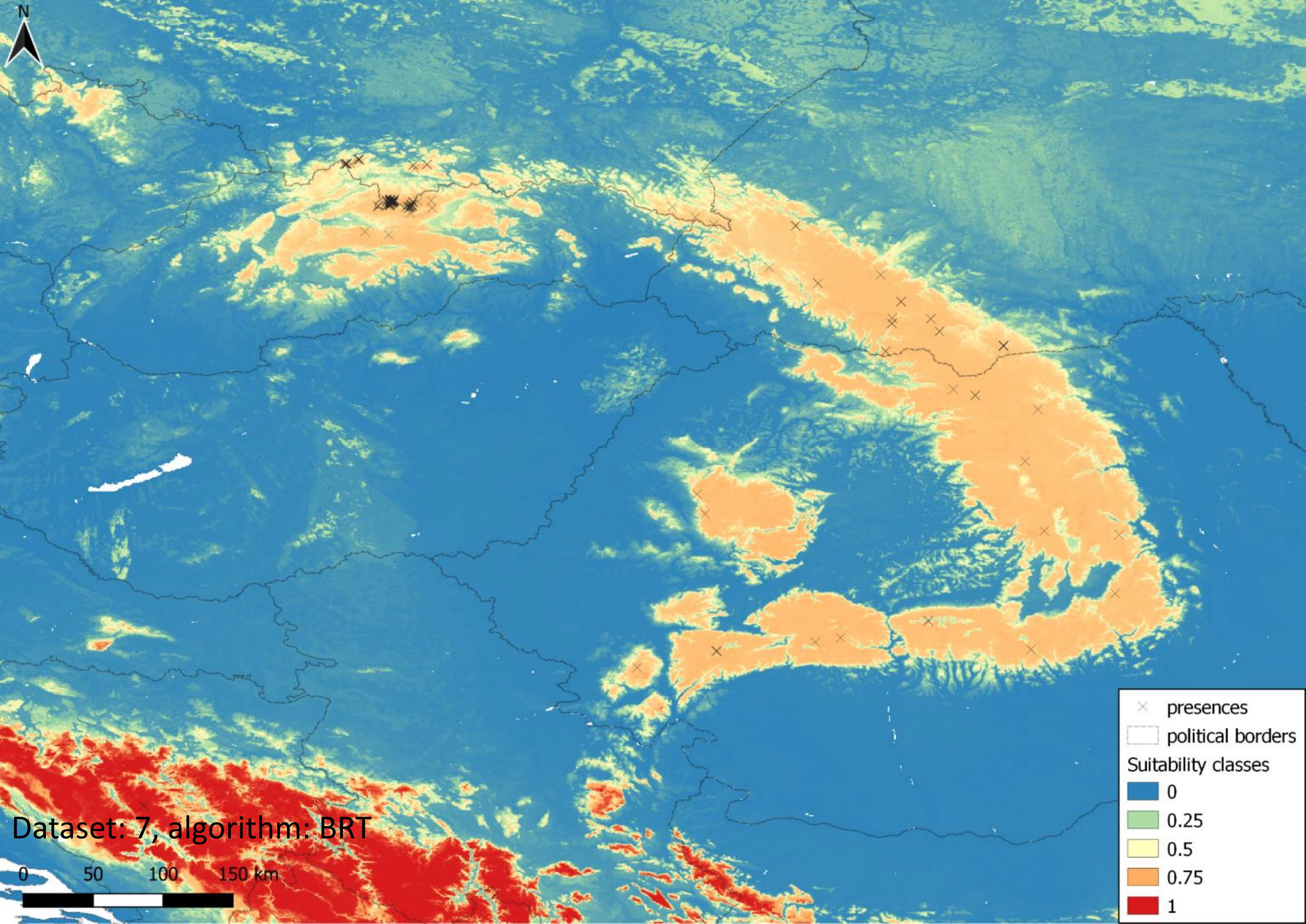
N
presences
political borders
Suitability classes
0
0.25
0.5
0.75
1
Dataset: 7, algorithm: BRT
0
50
100
150 km

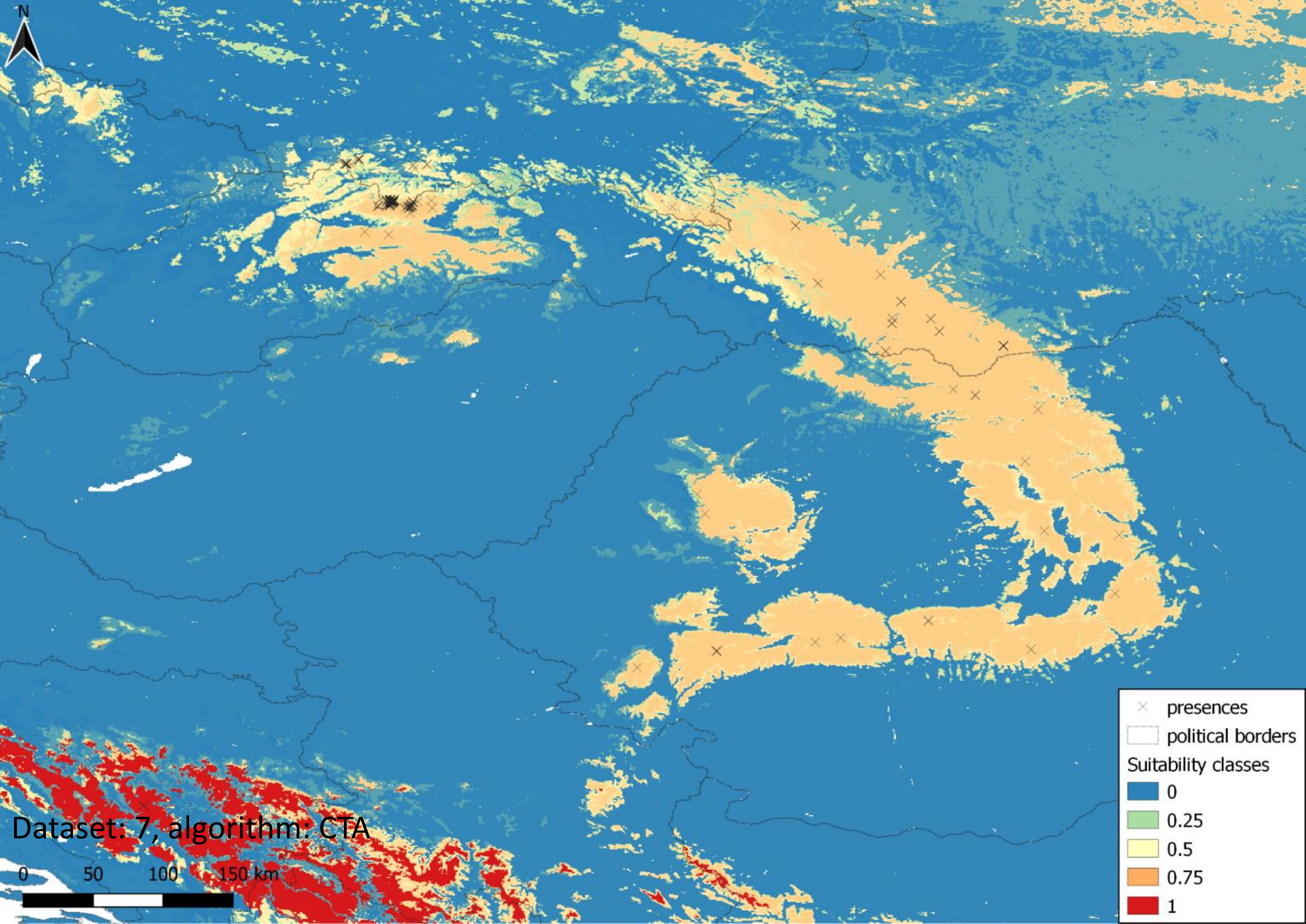
N
presences
political borders
Suitability classes
0
0.25
0.5
0.75
1
Dataset: 7, algorithm: CTA
0
50
100
150 km

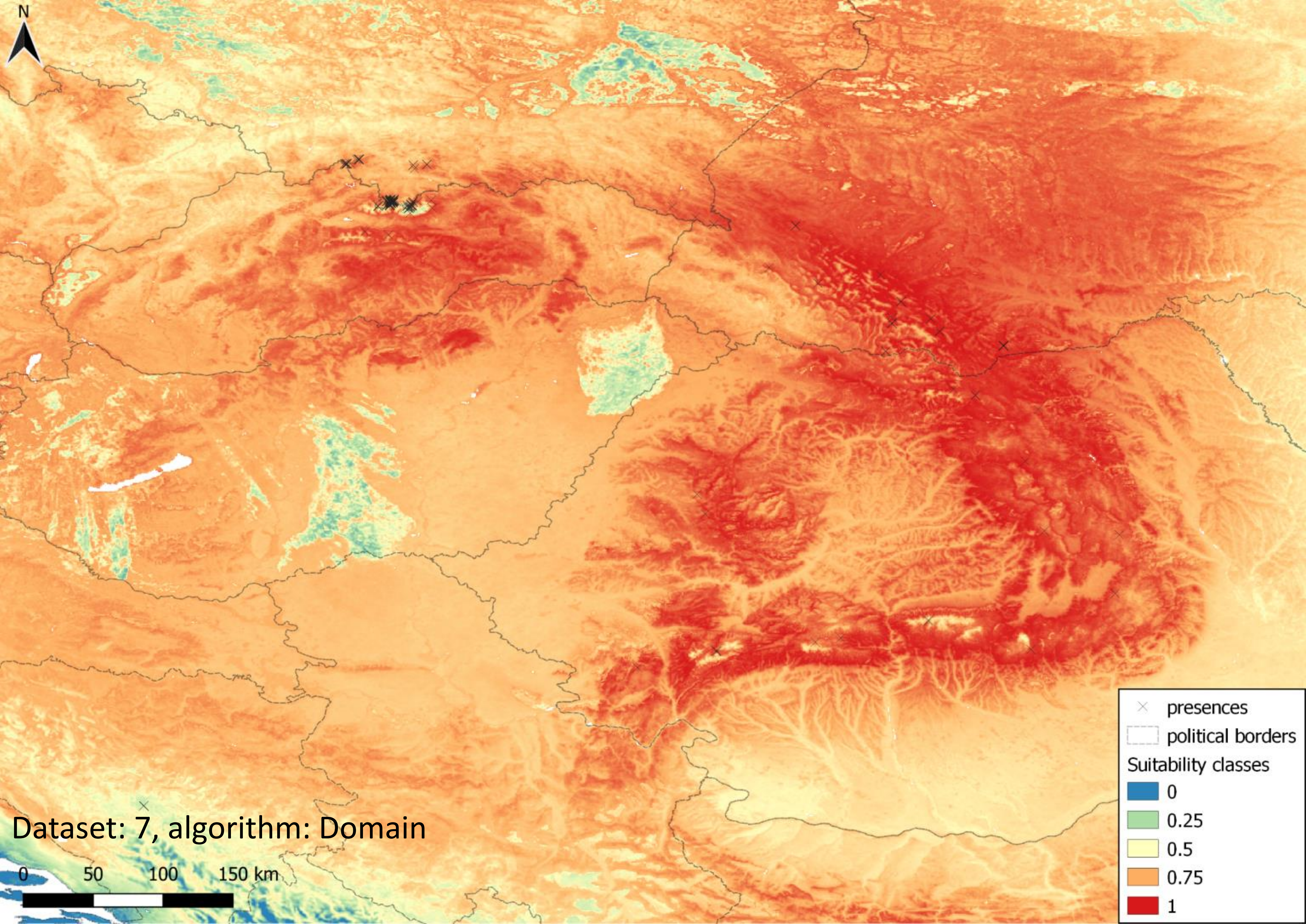

N
Dataset: 7, algorithm: Domain
0
50
100
150 km
presences
political borders
Suitability classes
0
0.25
0.5
0.75
1

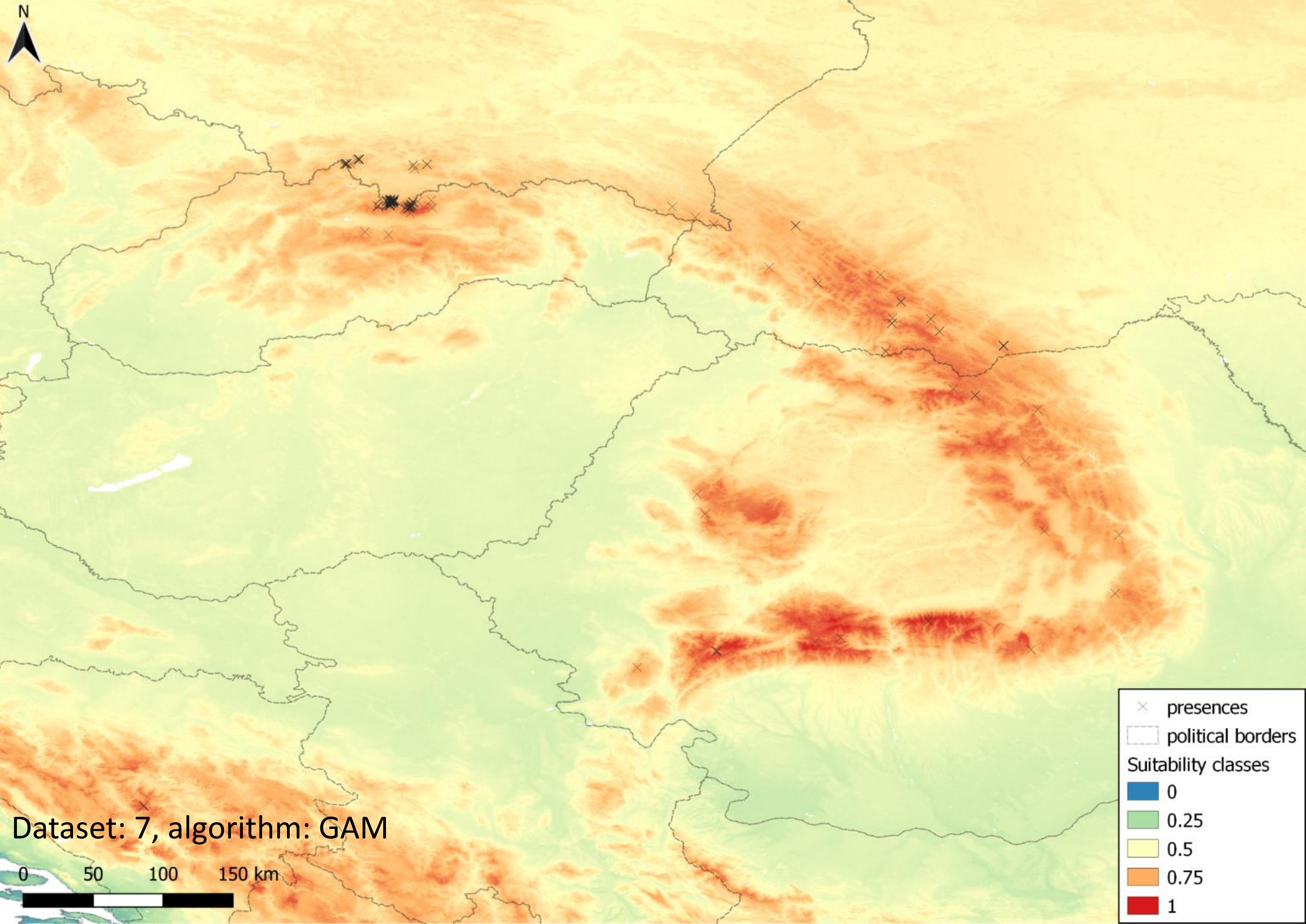
N
presences
political borders
Suitability classes
0
0.25
0.5
0.75
1
Dataset: 7, algorithm: GAM
0
50
100
150 km

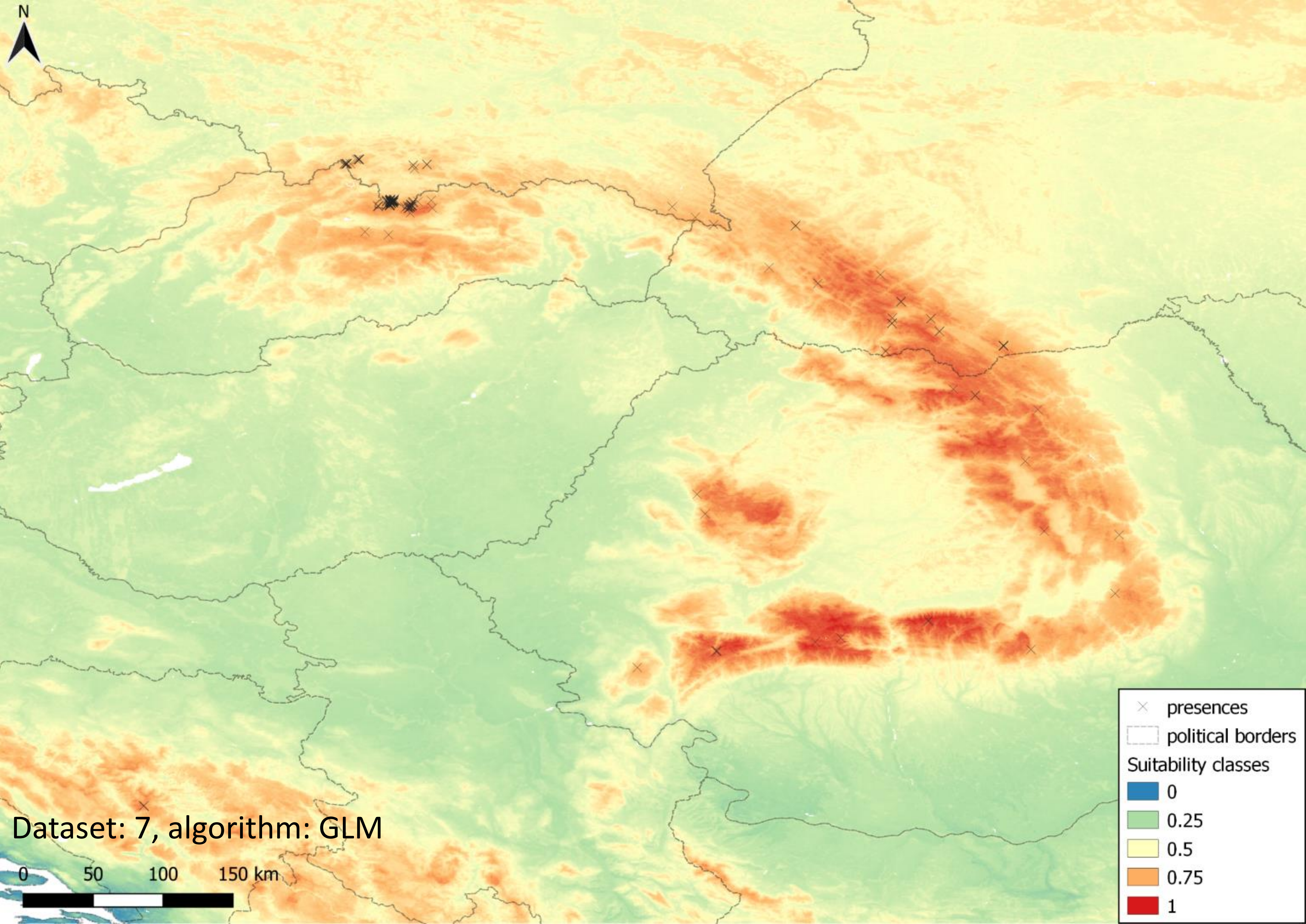
N
presences
political borders
Suitability classes
0
0.25
0.5
0.75
1
Dataset: 7, algorithm: GLM
0
50
100
150 km

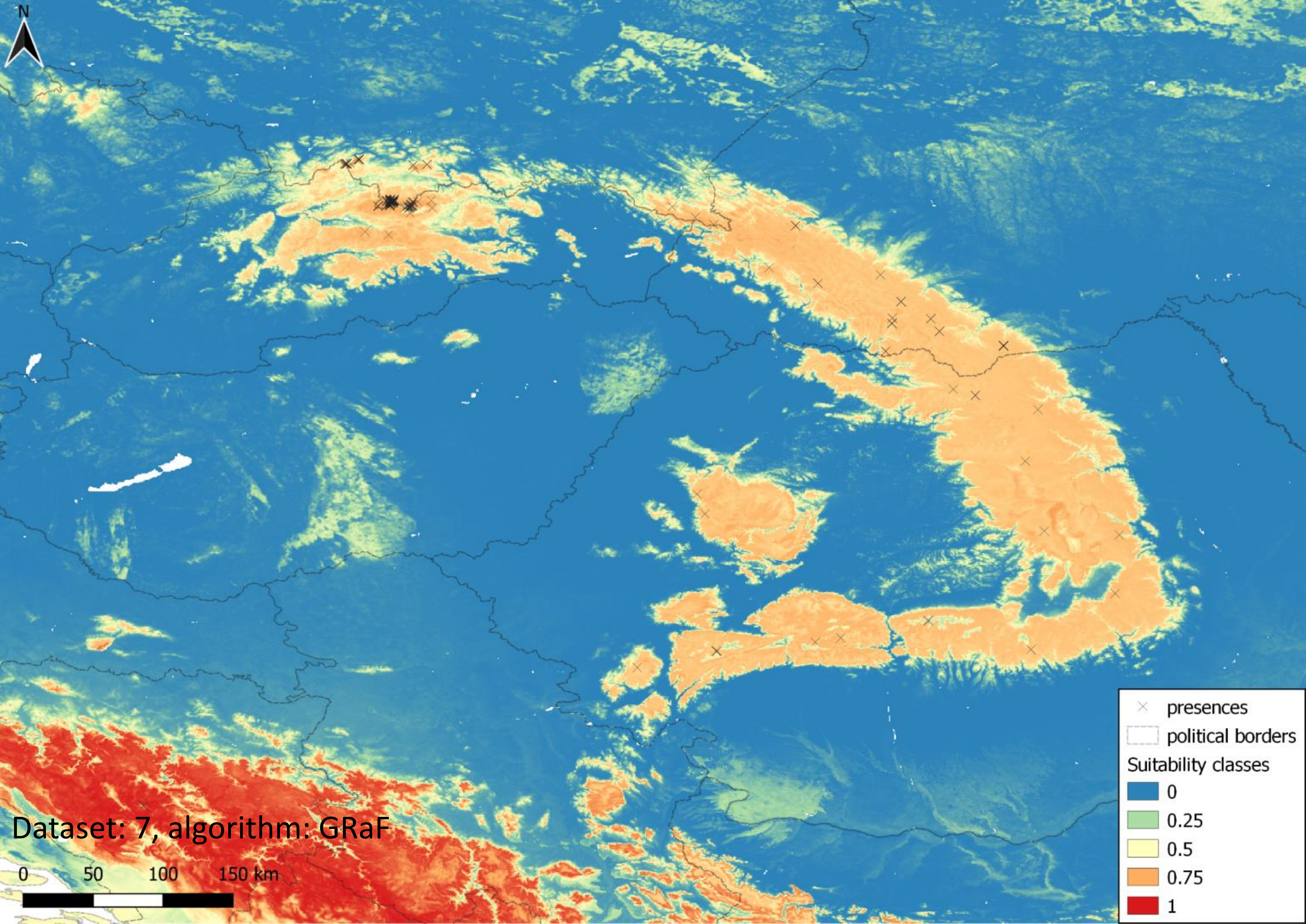
N
presences
political borders
Suitability classes
0
0.25
0.5
0.75
1
Dataset: 7, algorithm: GRaF
0
50
100
150 km

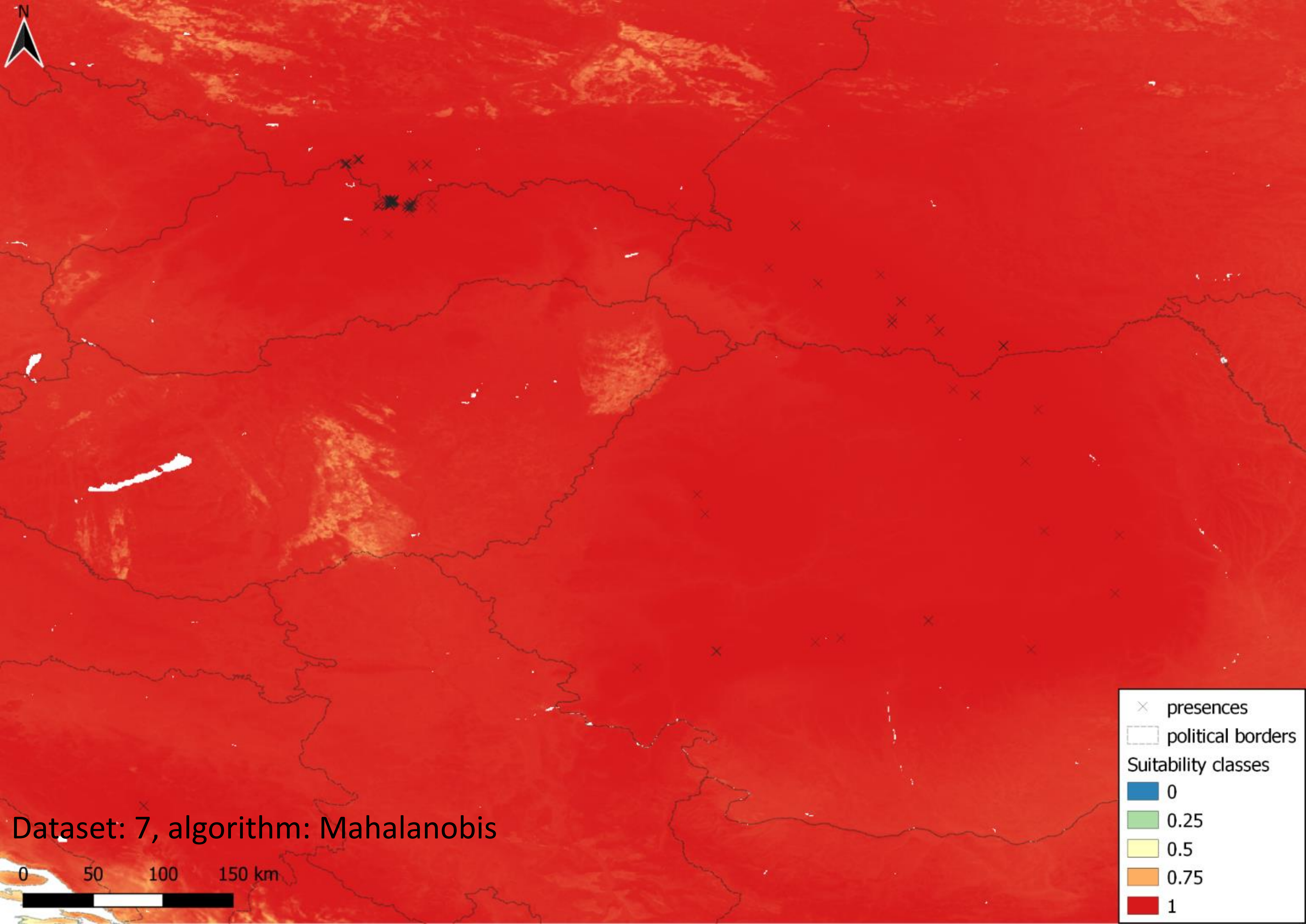
N
presences
political borders
Suitability classes
0
0.25
0.5
0.75
1
Dataset: 7, algorithm: Mahalanobis
0
50
100
150 km

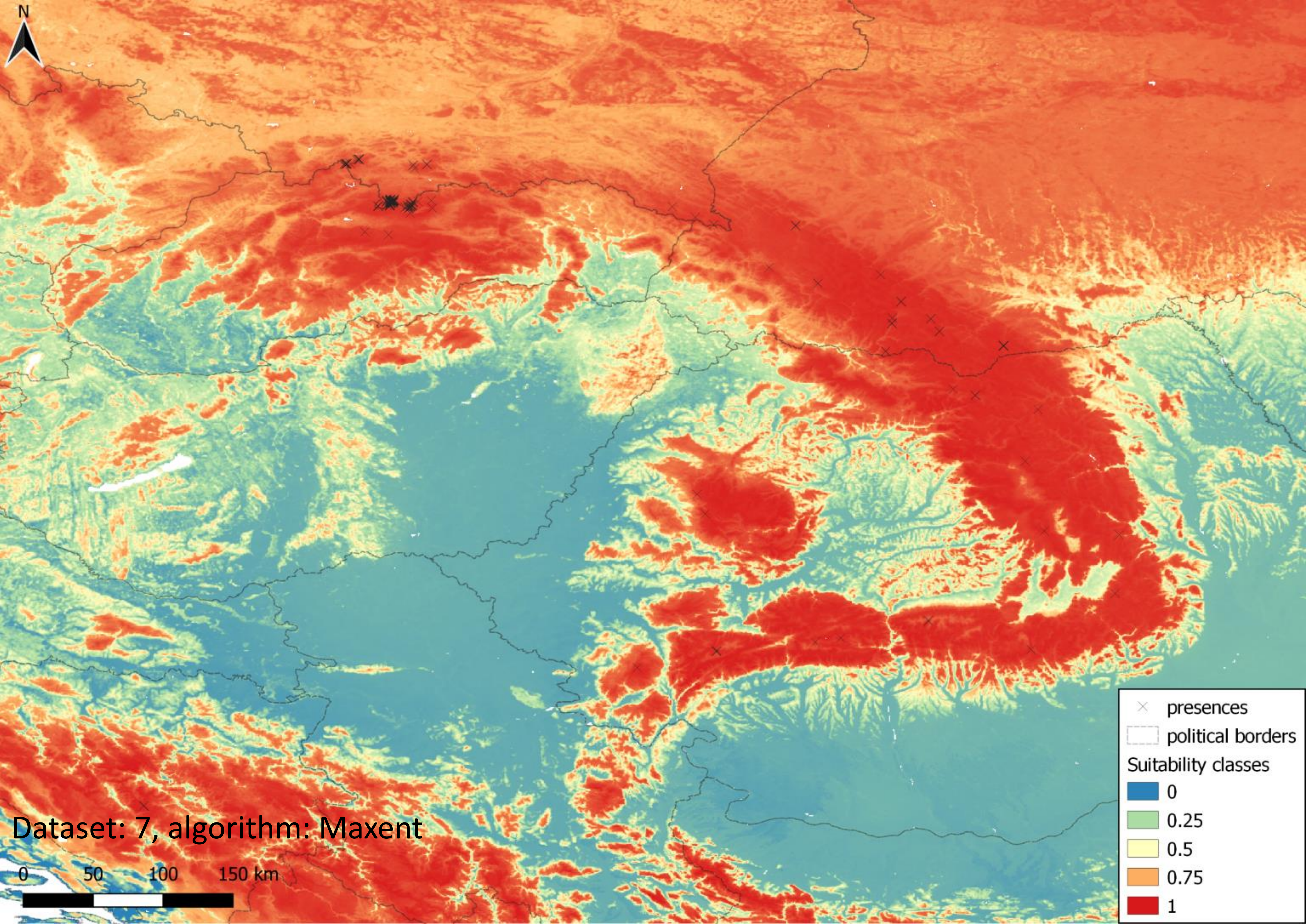
N
presences
political borders
Suitability classes
0
0.25
0.5
0.75
1
Dataset: 7, algorithm: Maxent
0
50
100
150 km

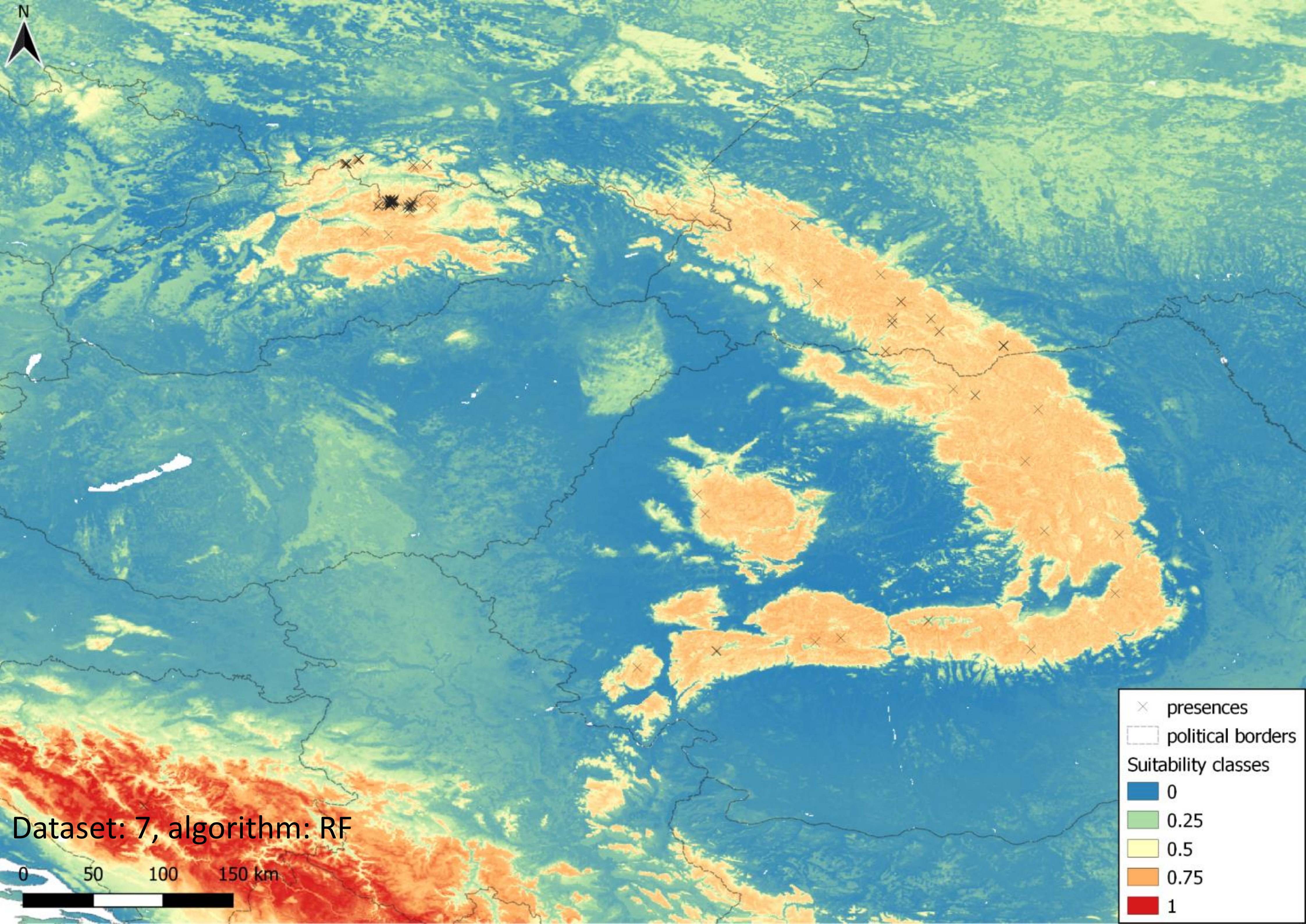
N
presences
political borders
Suitability classes
0
0.25
0.5
0.75
1
Dataset: 7, algorithm: RF
0
50
100
150 km

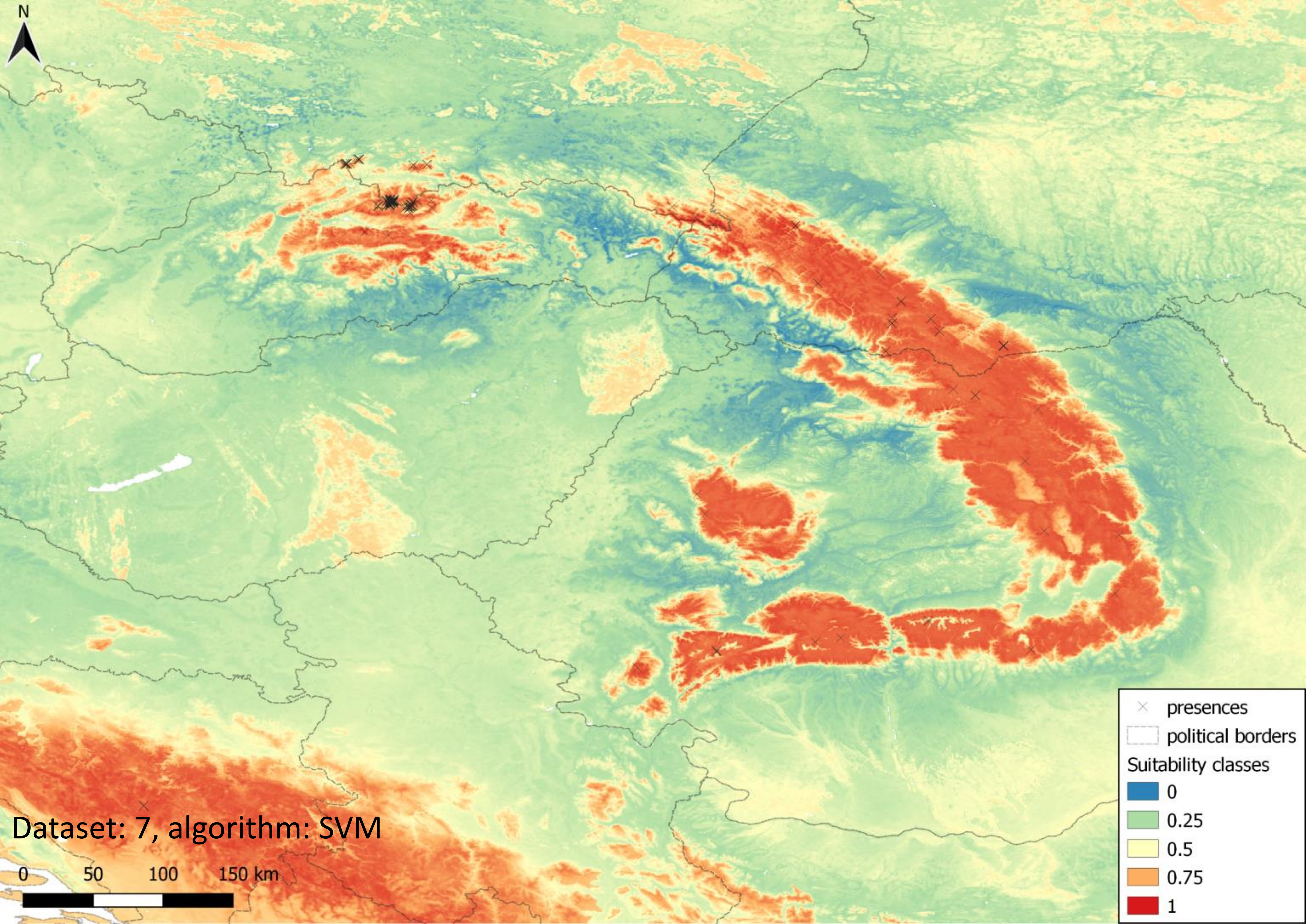
N
Dataset: 7, algorithm: SVM
0
50
100
150 km
presences
political borders
Suitability classes
0
0.25
0.5
0.75
1
